# Palladium catalyzed regioselective B–C(sp) coupling *via* direct cage B–H activation: synthesis of B(4)-alkynylated *o*-carboranes†
†Electronic supplementary information (ESI) available. CCDC 1455897 and 1455898. For ESI and crystallographic data in CIF or other electronic format see DOI: 10.1039/c6sc00901h


**DOI:** 10.1039/c6sc00901h

**Published:** 2016-05-13

**Authors:** Yangjian Quan, Cen Tang, Zuowei Xie

**Affiliations:** a Department of Chemistry , State Key Laboratory of Synthetic Chemistry , The Chinese University of Hong Kong , Shatin N. T. , Hong Kong , China . Email: zxie@cuhk.edu.hk

## Abstract

Pd-catalyzed carboxylic acid guided regioselective alkynylation of cage B(4)–H bonds in *o*-carboranes has been achieved for the first time using two different catalytic systems. In the presence of 5 mol% Pd(OAc)_2_ and 3 equiv. of AgOAc, the reaction of 1-COOH-2-R^1^-C_2_B_10_H_10_ with R_3_SiC≡CBr in ClCH_2_CH_2_Cl gives 4-(R_3_SiC≡C)-2-R^1^-*o*-C_2_B_10_H_10_ in moderate to high yields. This reaction is compatible with alkynes possessing sterically bulky silyl groups such as ^i^Pr_3_Si or ^*t*^BuMe_2_Si. Meanwhile, another catalytic system of Pd(OAc)_2_/AgOAc/K_2_HPO_4_ can catalyze the direct B(4)-alkynylation of 1-COOH-2-R^1^-C_2_B_10_H_10_ with terminal alkynes R^2^C≡CH in moderate to high yields. The latter has a broader substrate scope from bulky silyl to aromatic to carboranyl substituents. Desilylation of the resultant products affords carboranyl acetylene 4-(HC≡C)-2-R^1^-*o*-C_2_B_10_H_10_ which can undergo further transformations such as Sonogashira coupling, dimerization and click reactions. It is suggested that the above two catalytic systems may proceed *via* Pd(ii)–Pd(iv)–Pd(ii) and Pd(ii)–Pd(0)–Pd(ii) catalytic cycles, respectively. In addition, the silver salt is found to promote the decarboxylation reaction and thereby controls the mono-selectivity.

## Abstract 2

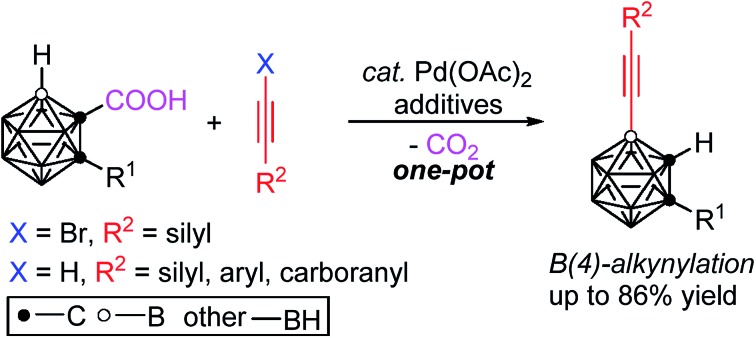
Palladium-catalyzed direct intermolecular coupling of *o*-carboranes with alkynyl bromides or terminal alkynes has been achieved, for the first time, with the help of a traceless directing group –COOH, leading to the synthesis of a series of new cage B(4)-alkynylated-*o*-carboranes in high yields with excellent regioselectivity.

## Introduction

The development of efficient synthetic methodologies to incorporate alkyne motifs has received broad interest, as they are not only important building blocks in natural products, pharmaceuticals and materials^1^ but also essential functional groups in cross-coupling, metathesis and cycloaddition reactions.^2^ Meanwhile, carboranyl acetylenes have proved to be useful basic units in molecular rods,^3^ nonlinear optical materials,^4^ supramolecular design,^5^ nanovehicles^6^ and metal–organic frameworks.^7^ As there is a lack of direct and efficient methodologies for the synthesis of B-alkynylated carboranes, the alkyne moieties in the aforementioned materials are generally connected to cage carbon atoms,^3^–^8^ which limits the application scope of the carborane derivatives.

**Scheme 1:**
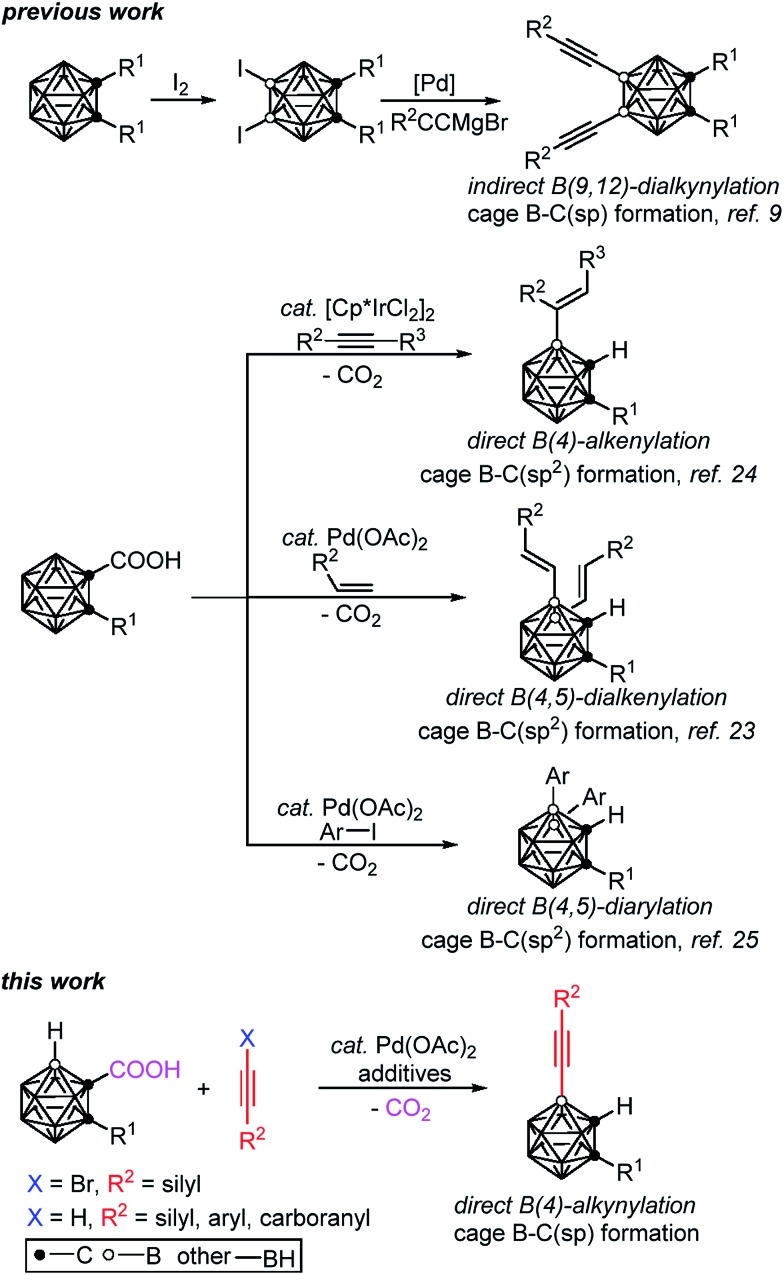
Selected examples of transition metal catalyzed formation of cage B–C(sp) and B–C(sp^2^) bonds in *o*-carboranes.

Though cage boron alkynylated carboranes can be prepared by two-step reactions, such as the selective iodination of an *o*-carborane, followed by Pd(0)-catalyzed cross-coupling with alkynyl Grignard reagents,^9^ the installation of iodo groups to specific positions on the carboranes is necessary (Scheme 1). However, the selective iodination of cage B(4,5,7,11)–H is rather challenging, if not impossible.^8^ Thus, we aim to develop new methodologies for the selective and direct alkynylation of carboranes *via* cage B–H activation.

Directing groups are essential in transition metal catalyzed C–H activation due to their ability to chelate the metal catalyst, position it for selective C–H cleavage, and reduce activation energy by stabilizing the metallacycle intermediates.^10^ Nevertheless, strategies using directing groups suffer from limitations when the directing groups are not present in the target molecules. To overcome this problem, the use of traceless directing groups is obviously an ideal method. Recently, the use of –COOH as a weak coordinating yet efficient directing group for transition metal catalyzed phenyl C–H activation has been documented, and has been found to be easily removed by decarboxylation after the reaction.10*h* Subsequently, carboxylic acid directed phenyl C–H olefination,^11^ arylation,^12^ alkylation,^13^ acylation,^14^ carboxylation,^15^ amination,^16^ hydroxylation^17^ and halogenation^18^ have been successfully developed. However, to the best of our knowledge, the direct alkynylation of C–H bonds guided by –COOH is still elusive, although nitrogen-based directing-group-guided transition-metal catalyzed phenyl C–H alkynylation has been recently documented using alkynyl halides,^19^ hypervalent iodine-alkyne reagents^20^ and terminal alkynes^21^ as the alkynylating reagents. Meanwhile, oxidative coupling of two C–H bonds for the formation of a C–C bond has received growing interest due to its benefits which include atom-economy, step-economy and less waste.^22^ Compared with the achievements of phenyl C–H bond oxidative coupling, the regioselective and direct oxidative coupling of an organic C–H bond with a cage B–H bond in *o*-carboranes is very rare.^23^

Very recently, our group has developed a transition metal catalyzed –COOH guided cage B–H alkenylation^23^,^24^ and arylation^25^ of *o*-carboranes, in which the carboxyl group is removed in a one-pot fashion. Inspired by these results and other cage B–H activation reactions,^26^–^29^ we have extended our research to investigate direct cage B–H alkynylation by alkynyl halides through a Pd(ii)–Pd(iv)–Pd(ii) catalytic cycle and by terminal alkynes *via* a Pd(ii)–Pd(0)–Pd(ii) catalytic cycle. These new findings are reported in this article (Scheme 1).

## Results and discussion

### Alkynylation using alkynyl halides

The initial reaction of 1-COOH-2-CH_3_-*o*-C_2_B_10_H_10_ (**1a**) with ^i^Pr_3_SiC≡CBr in the presence of 10 mol% Pd(OAc)_2_ and 1 equiv. of AgOAc in toluene at 90 °C for 6 h did not give any of the desired product (entry 1, Table 1). Replacement of toluene with 1,2-dichloroethane (DCE) afforded the desired coupling product 4-(^i^Pr_3_SiC≡C)-2-CH_3_-*o*-C_2_B_10_H_10_ in 40% GC yield (entry 2, Table 1). Increasing the amount of AgOAc to 3 equiv. resulted in 90% GC yield of **3a** (entry 4, Table 1). Higher or lower reaction temperatures led to decreased yields of **3a** (entries 5 and 6, Table 1). Lowering the catalyst loading to 5 mol% did not change the reaction efficiency (entry 7, Table 1). In view of the yields of **3a**, entry 7 in Table 1 was chosen as the optimal reaction conditions.

**Table 1:** Optimization of reaction conditions using alkynyl bromide^*a*^

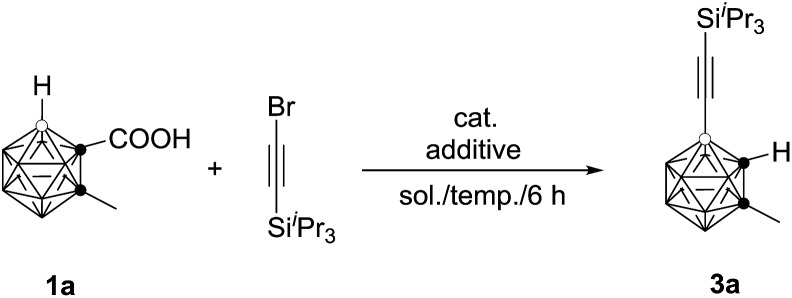					
Entry	Cat (mol%)	Additive (equiv.)	Solvent	Temp (°C)	Yield^*b*^ (%)
1	Pd(OAc)_2_ (10)	AgOAc (1)	Toluene	90	N.R.
2	Pd(OAc)_2_ (10)	AgOAc (1)	DCE	90	40
3	Pd(OAc)_2_ (10)	AgOAc (2)	DCE	90	67
4	Pd(OAc)_2_ (10)	AgOAc (3)	DCE	90	90
5	Pd(OAc)_2_ (10)	AgOAc (3)	DCE	80	70
6	Pd(OAc)_2_ (10)	AgOAc (3)	DCE	100	75
7	Pd(OAc)_2_ (5)	AgOAc (3)	DCE	90	89
8	Pd(OAc)_2_ (2.5)	AgOAc (3)	DCE	90	78
9	Pd(TFA)_2_ (5)	AgOAc (3)	DCE	90	82
10	Pd(OAc)_2_ (5)	Ag_2_CO_3_ (2)	DCE	90	75
11	Pd(OAc)_2_ (5)	Ag_2_O (2)	DCE	90	63
12	Pd(OAc)_2_ (5)	AgNO_3_ (3)	DCE	90	30

^*a*^Reactions were conducted on a 0.05 mmol scale in 0.5 mL of solvent in a closed flask for 6 h; DCE = 1,2-dichloroethane; TFA = trifluoroacetate.

^*b*^GC yields.

A variety of carborane monocarboxylic acids (**1**) were examined under the chosen optimal reaction conditions, and the results are compiled in Table 2. All alkyl, alkenyl and aryl substituents on cage C(2), regardless of electronic properties, afforded the coupling products **3** in high isolated yields (entries 1–10 and 13, Table 2). For the heteroatom containing substrate **1j**, the product **3j** was isolated in 78% yield (entry 10, Table 2), whereas that bearing a thiophenyl group (**1l**) afforded the product **3l** in 54% yield (entry 12, Table 2) probably due to the interaction of Pd with the S atom. Meanwhile, substrate **1k** with a naphthyl substituent on cage C(2) gave **3k** in only 40% isolated yield (entry 11, Table 2). For R^1^ = H, an inseparable mixture was produced (entry 14, Table 2). When R^1^ = Me_3_Si, the desilylation species **3n** was isolated in 41% yield after work up (entry 15, Table 2).

**Table 2:** Synthesis of cage B(4)-alkynylated *o*-carboranes using alkynyl bromide^*a*^

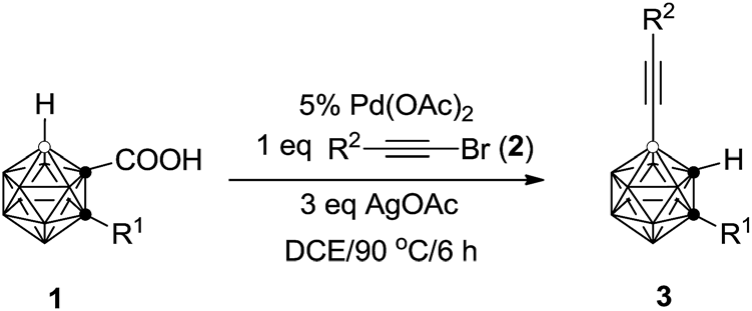			
Entry	R^1^	R^2^ (**2**)	Isolated yield (%)
1	Me (**1a**)	^i^Pr_3_Si	81 (**3a**)
2	Et (**1b**)	^i^Pr_3_Si	76 (**3b**)
3	^i^Pr (**1c**)	^i^Pr_3_Si	75 (**3c**)
4	Bn (**1d**)	^i^Pr_3_Si	73 (**3d**)
5	Ph (**1e**)	^i^Pr_3_Si	77 (**3e**)
6	4-MeC_6_H_4_ (**1f**)	^i^Pr_3_Si	82 (**3f**)
7	3,5-(CH_3_)_2_C_6_H_3_ (**1g**)	^i^Pr_3_Si	70 (**3g**)
8	4-CF_3_C_6_H_4_ (**1h**)	^i^Pr_3_Si	81 (**3h**)
9	4-ClC_6_H_4_ (**1i**)	^i^Pr_3_Si	72 (**3i**)
10	4-MeOC_6_H_4_ (**1j**)	^i^Pr_3_Si	78 (**3j**)
11	1-Naphenyl (**1k**)	^i^Pr_3_Si	40 (**3k**)
12	2-Thiophenyl (**1l**)	^i^Pr_3_Si	54 (**3l**)
13	EtCH=C(Et) (**1m**)	^i^Pr_3_Si	80 (**3m**)
14	H (**1n**)	^i^Pr_3_Si	Messy
15	Me_3_Si (**1o**)	^i^Pr_3_Si	41^*b*^ (**3n**)
16	Me (**1a**)	^*t*^BuMe_2_Si	70 (**3p**)
17	Me (**1a**)	Me_3_Si	N.R.^*c*^

^*a*^Reactions were conducted on a 0.2 mmol scale of **1** in a closed flask.

^*b*^Me_3_Si was removed after work up.

^*c*^N.R. = no reaction.

In contrast to R^1^ at cage C(2), the scope of R^2^ is highly limited in such a coupling reaction. ^*t*^BuMe_2_SiC≡CBr worked well to give **3p** in 70% isolated yield (entry 16, Table 2). However, less hindered Me_3_SiC≡CBr was not reactive, probably due to its propensity to coordinate with a Pd center *via* the π bond (entry 17, Table 2). Such a phenomenon was also observed in phenyl C–H alkynylations using R_3_SiC≡CBr as reagents.^30^ It was noted that other alkynyl bromides such as PhC≡CBr and ^*t*^BuC≡CBr were not compatible with this reaction.

### Alkynylation using terminal alkynes

As the previous method has a limited substrate scope, we wanted to develop a more atom- and step-economic method for cage B–H alkynylation using terminal alkynes as reagents. We commenced our studies by screening for a suitable base for the oxidative coupling of cage B–H in 1-COOH-2-CH_3_-*o*-C_2_B_10_H_10_ (**1a**) with ^i^Pr_3_SiC≡CH under the aforementioned optimal reaction conditions. No reaction was observed in the absence of a base (entry 1, Table 3). The addition of 2 equiv. of K_2_HPO_4_ afforded the target product **3a** in 30% GC yield with ^i^Pr_3_SiC≡C–C≡C^i^Pr_3_Si as the side product (entry 2, Table 3). To inhibit the formation of a homocoupling side product, ^i^Pr_3_SiC≡CH was added slowly *via* a syringe pump, leading to a significantly increased yield of **3a** to 56% GC yield (entry 3, Table 3). The yield was further improved to 75% if 2 equiv. of the terminal alkyne was used (entry 4, Table 3). Replacement of 1,2-dichloroethane (DCE) with toluene resulted in a slightly higher yield of **3a** (entry 5, Table 3). Decreasing the reaction temperature to 80 °C afforded **3a** in 86% GC yield (entry 6, Table 3). In view of the yields of **3a**, entry 6 in Table 3 was chosen as the optimal reaction conditions.

**Table 3:** Optimization of reaction conditions using terminal alkynes^*a*^

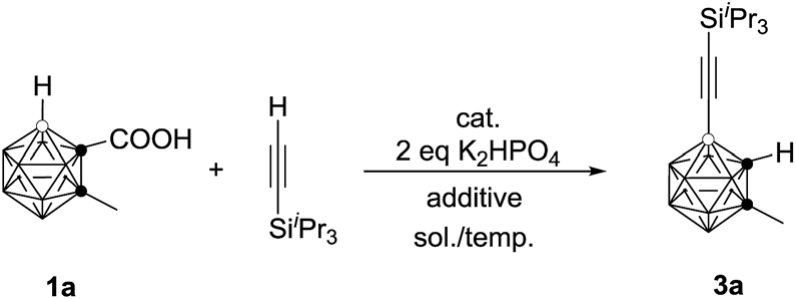					
Entry	Cat (mol%)	Additive (equiv.)	Solvent	Temp (°C)	Yield^*b*^ (%)
1^*c*^	Pd(OAc)_2_ (5)	AgOAc (3)	DCE	90	N.R.
2	Pd(OAc)_2_ (5)	AgOAc (3)	DCE	90	30
3	Pd(OAc)_2_ (5)	AgOAc (3)	DCE	90	56^*d*^
4	Pd(OAc)_2_ (5)	AgOAc (3)	DCE	90	75^*d*^ ^,^^*e*^
5	Pd(OAc)_2_ (5)	AgOAc (3)	Toluene	90	78^*d*^ ^,^^*e*^
6	Pd(OAc)_2_ (5)	AgOAc (3)	Toluene	80	86^*d*^ ^,^^*e*^
7	Pd(OAc)_2_ (5)	AgOAc (3)	Toluene	70	Trace
8	Pd(OAc)_2_ (3)	AgOAc (3)	DCE	90	18
9	Pd(TFA)_2_ (5)	AgOAc (3)	DCE	90	26
10	Pd_2_(dba)_3_ (5)	AgOAc (3)	DCE	90	21
11	Pd(OAc)_2_ (5)	Ag_2_CO_3_ (2)	DCE	90	15
12	Pd(OAc)_2_ (5)	Ag_2_O (2)	DCE	90	12
13	Pd(OAc)_2_ (5)	AgNO_3_ (3)	DCE	90	Trace

^*a*^Reactions were conducted on a 0.05 mmol scale of **1a** in 0.5 mL of solvent in the presence of 2 equiv. of K_2_HPO_4_ in a closed flask for 10 h; DCE = 1,2-dichloroethane; TFA = trifluoroacetate; dba = dibenzylideneacetone.

^*b*^GC yields.

^*c*^Without K_2_HPO_4_.

^*d*^Terminal alkyne was added dropwise by a syringe pump over a period of 10 h.

^*e*^Two equiv. of terminal alkyne was added.

This reaction has a much broader substrate scope (R^2^ = silyl, phenyl and carboranyl). The results are compiled in Table 4. For R^1^ = alkyl groups, the isolated yields of **3** are comparable to those observed in Table 2. However, if R^1^ = aryl unit such as **1g**, the isolated yield of **3g** is 30% (entry 4, Table 4), which is significantly lower than that of 70% shown in entry 7, Table 2. On the other hand, compounds **1n** (R^1^ = H) and **1o** (R^1^ = Me_3_Si) give **3n** in 35% and 74% yields, respectively (entries 5 and 6, Table 4). These yields are much higher than those found in the previous reaction (entries 14 and 15, Table 2). The reasons for this phenomenon are not clear at this stage.

**Table 4:** Synthesis of cage B(4)-alkynylated *o*-carboranes using terminal alkynes^*a*^

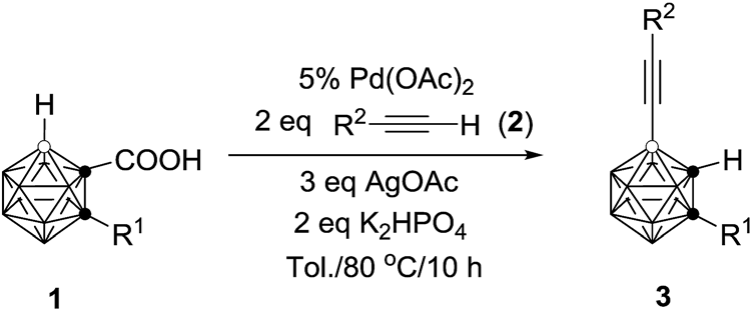			
Entry	R^1^	R^2^ (**2**)	Isolated yield (%)
1	Me (**1a**)	^i^Pr_3_Si	79 (**3a**)
2	^i^Pr (**1c**)	^i^Pr_3_Si	86 (**3c**)
3	Bn (**1d**)	^i^Pr_3_Si	70 (**3d**)
4	3,5-(CH_3_)_2_C_6_H_3_ (**1g**)	^i^Pr_3_Si	30 (**3g**)
5	H (**1n**)	^i^Pr_3_Si	35 (**3n**)
6	Me_3_Si (**1o**)	^i^Pr_3_Si	74^*b*^ (**3n**)
7	Me (**1a**)	^*t*^BuMe_2_Si	72 (**3p**)
8	Me (**1a**)	Ph	52^*c*^ (**3r**)
9	Me (**1a**)	2-CH_3_C_6_H_4_	65^*c*^ (**3s**)
10	Me (**1a**)	2,6-(CH_3_)_2_C_6_H_3_	73^*c*^ (**3t**)
11	Me (**1a**)	2-^i^PrC_6_H_4_	80^*c*^ (**3u**)
12	Me (**1a**)	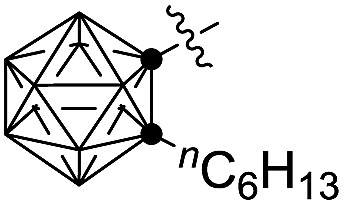	82^*c*^ (**3v**)
13	Me (**1a**)	4-CH_3_C_6_H_4_	48^*c*^ (**3w**)
14	Me (**1a**)	4-CF_3_C_6_H_4_	44^*c*^ (**3x**)

^*a*^Reactions were conducted on a 0.2 mmol scale of **1** in a closed flask.

^*b*^Me_3_Si was removed after work up.

^*c*^3 equiv. of terminal alkyne was used.

More importantly, this catalytic system is compatible with phenyl acetylene, producing the corresponding product **3r** in 52% isolated yield (entry 8, Table 4). The coupling efficiency was largely enhanced from R^2^ = Ph, 2-MeC_6_H_4_, 2,6-(Me)_2_C_6_H_3_, 2-^i^PrC_6_H_4_ to 1-^*n*^C_6_H_13_-*o*-C_2_B_10_H_10_, affording the corresponding products, **3s**, **3t**, **3u** and **3v**, in 65%, 73%, 80% and 82% isolated yields, respectively (entries 9–12, Table 4). It should be noted that *o*-carboranyl is a strong electron-withdrawing unit.4*c* In view of the isolated yields of **3w** and **3x** (entries 13 and 14, Table 4), the electronic effects on the reactions are not obvious as –CH_3_ and –CF_3_ have significantly different electronic properties. The above data (entries 8–14, Table 4) indicate strongly that bulkier substituents favor the formation of coupling products.

### Transformation of **3a**

**Scheme 2:**
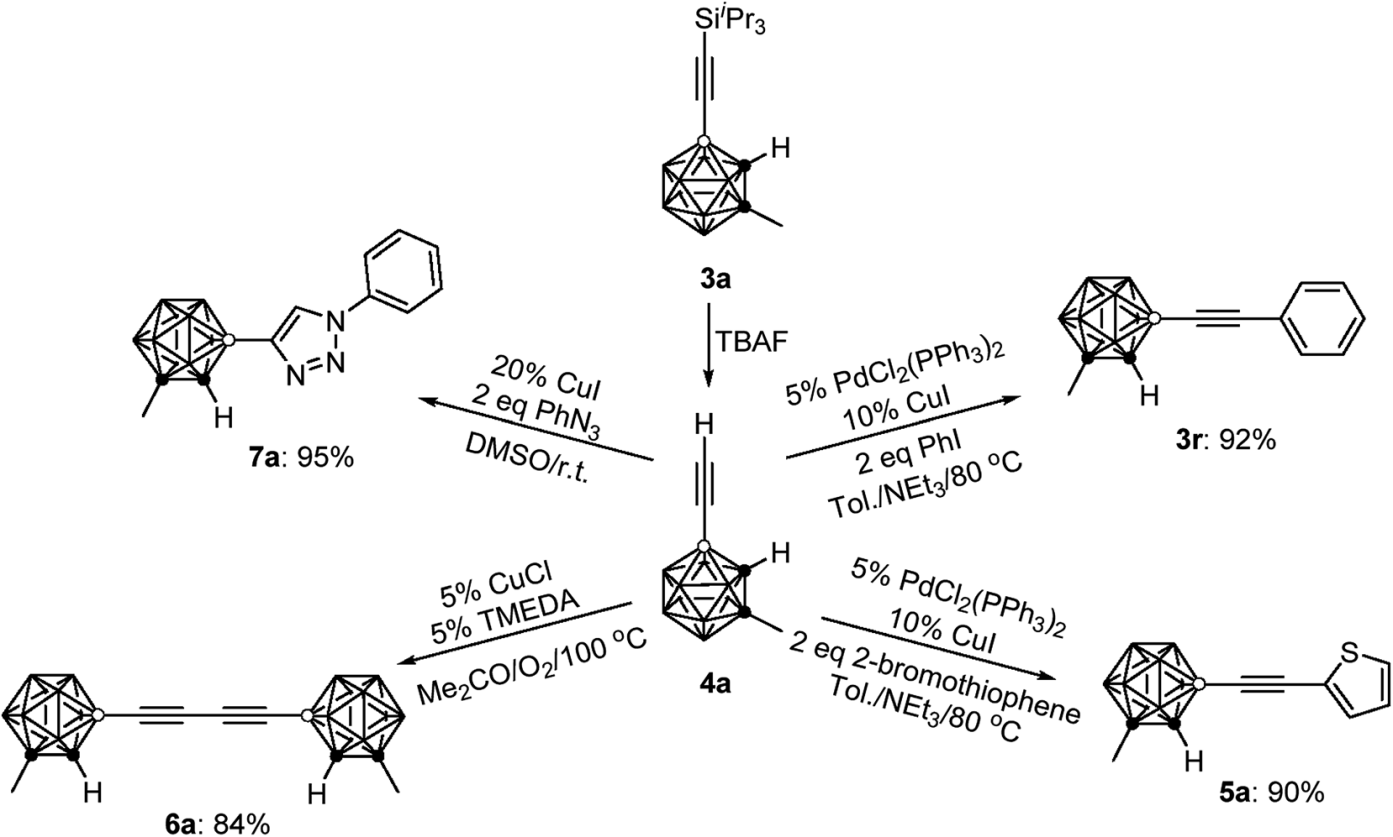
Transformations of **3a**.

To demonstrate the applications of the resultant compounds **3** as building blocks, further transformation of **3a** was carried out. The ^i^Pr_3_Si group in **3a** was readily removed by treatment with TBAF (TBAF = tetra-*n*-butylammonium fluoride) to afford quantitatively the terminal alkyne **4a** (Scheme 2). Like other terminal alkynes, compound **4a** can undergo various transformations to give different kinds of carborane-incorporated functional molecules. Sonogashira coupling of **4a** with iodobenzene or 2-bromothiophene generated **3r** or **5a** in 92% and 90% isolated yields, respectively. Glaser–Hay homocoupling of **4a** gave 1,4-dicarboranyldiacetylene (**6a**) in 84% isolated yield. A click reaction of **4a** with phenyl azide afforded carborane-functionalized 1,2,3-triazole (**7a**) in 95% isolated yield.

**Fig. 1:**
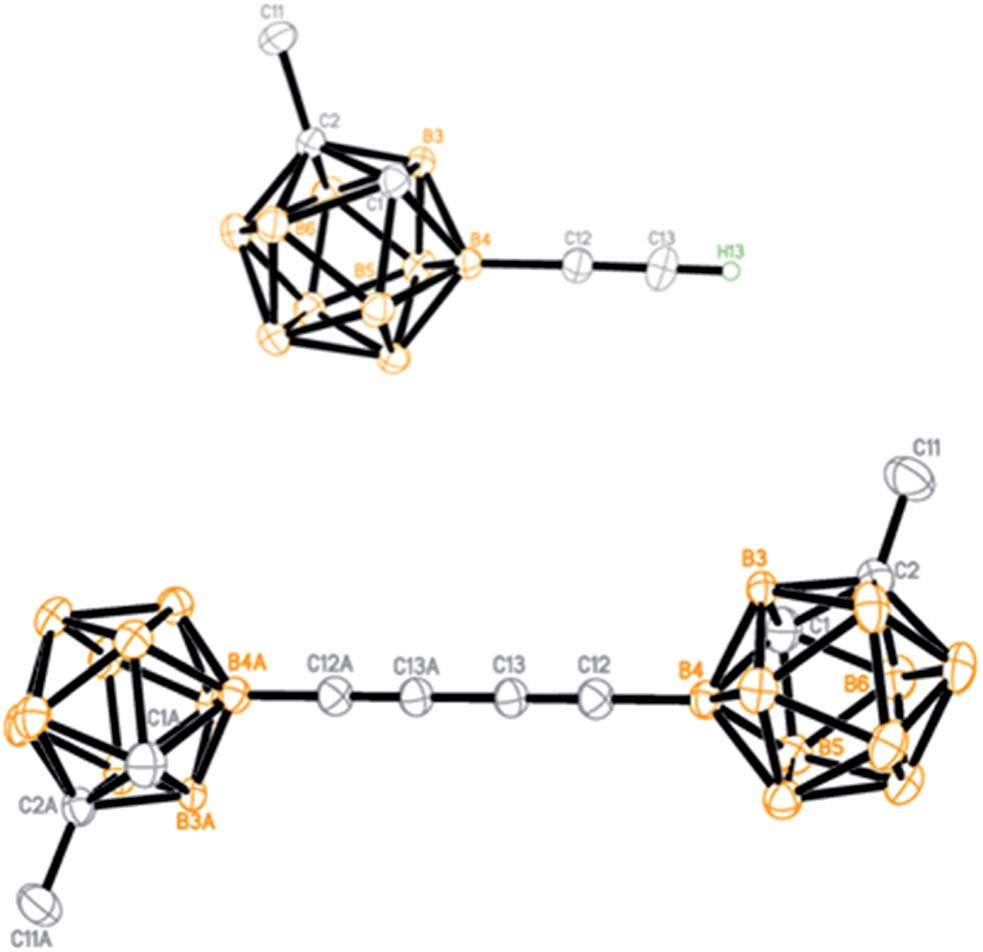
Molecular structures of **4a** (top) and **6a** (bottom) (only the terminal alkyne H atom is shown for clarity).

All new compounds **3** and **4a–7a** were fully characterized by ^1^H, ^13^C, and ^11^B NMR spectroscopy as well as high-resolution mass spectrometry (HRMS).^31^ Molecular structures of **4a** and **6a** were further confirmed by single-crystal X-ray analyses and are shown in Fig. 1. Experimental details are included in the ESI.†

### Reaction mechanism

**Scheme 3:**
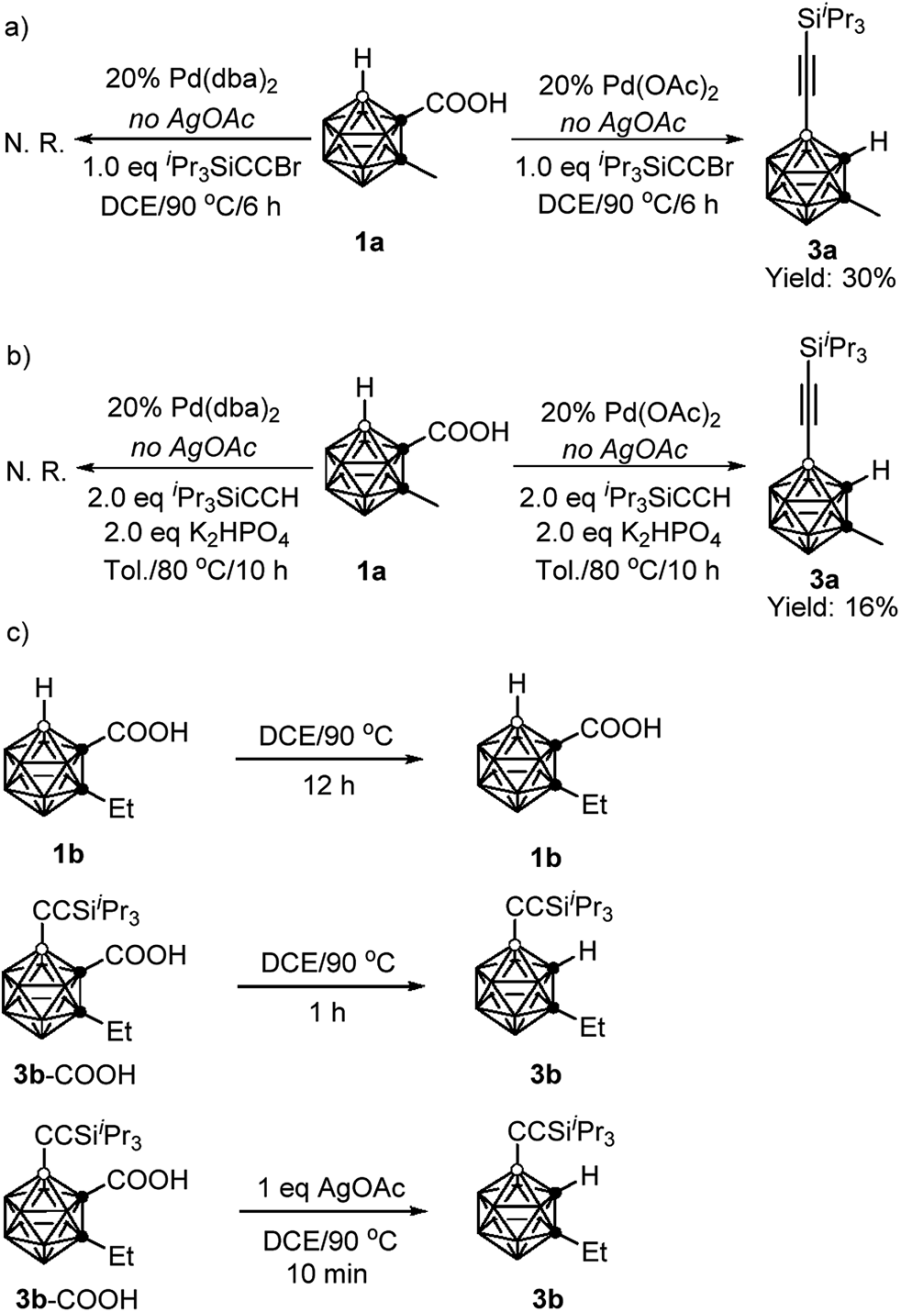
Control experiments.

To gain some insight into the reaction mechanism, the following control experiments were carried out. No reaction was observed if **1a** was treated with 1 equiv. of ^i^Pr_3_SiC≡CBr in the presence of 20 mol% Pd(dba)_2_ (dba = dibenzylideneacetone) in DCE at 90 °C for 6 h in the absence of AgOAc. On the other hand, under the same reaction conditions, replacement of Pd(dba)_2_ with Pd(OAc)_2_ gave the alkynylation product **3a** in 30% GC yield (Scheme 3a). Similarly, in the presence of 20 mol% Pd(OAc)_2_, the reaction of **1a** with 2 equiv. of ^i^Pr_3_SiC≡CH afforded **3a** in 16% GC yield without AgOAc as the oxidant. While, no **3a** was observed when 20 mol% Pd(dba)_2_ was used instead of Pd(OAc)_2_ (Scheme 3b). These results suggest that both cross-coupling reactions are initiated by Pd(ii) not Pd(0).

Decarboxylation of carboranyl carboxylic acids (**1b** and **3b**–COOH) was also examined (Scheme 3c). Compound **1b** was stable after heating at 90 °C for 12 h in DCE, whereas **3b**–COOH underwent complete decarboxylation within one hour under the same reaction conditions. Notably, it only took ten minutes to convert **3b**–COOH to **3b** in the presence of 1 equiv. of AgOAc. These results clearly indicate that the introduction of an alkynyl group at the cage B(4) site can induce the decarboxylation, and the addition of a silver salt can accelerate such decarboxylation, which is crucial for controlling the mono-selectivity.

**Scheme 4:**
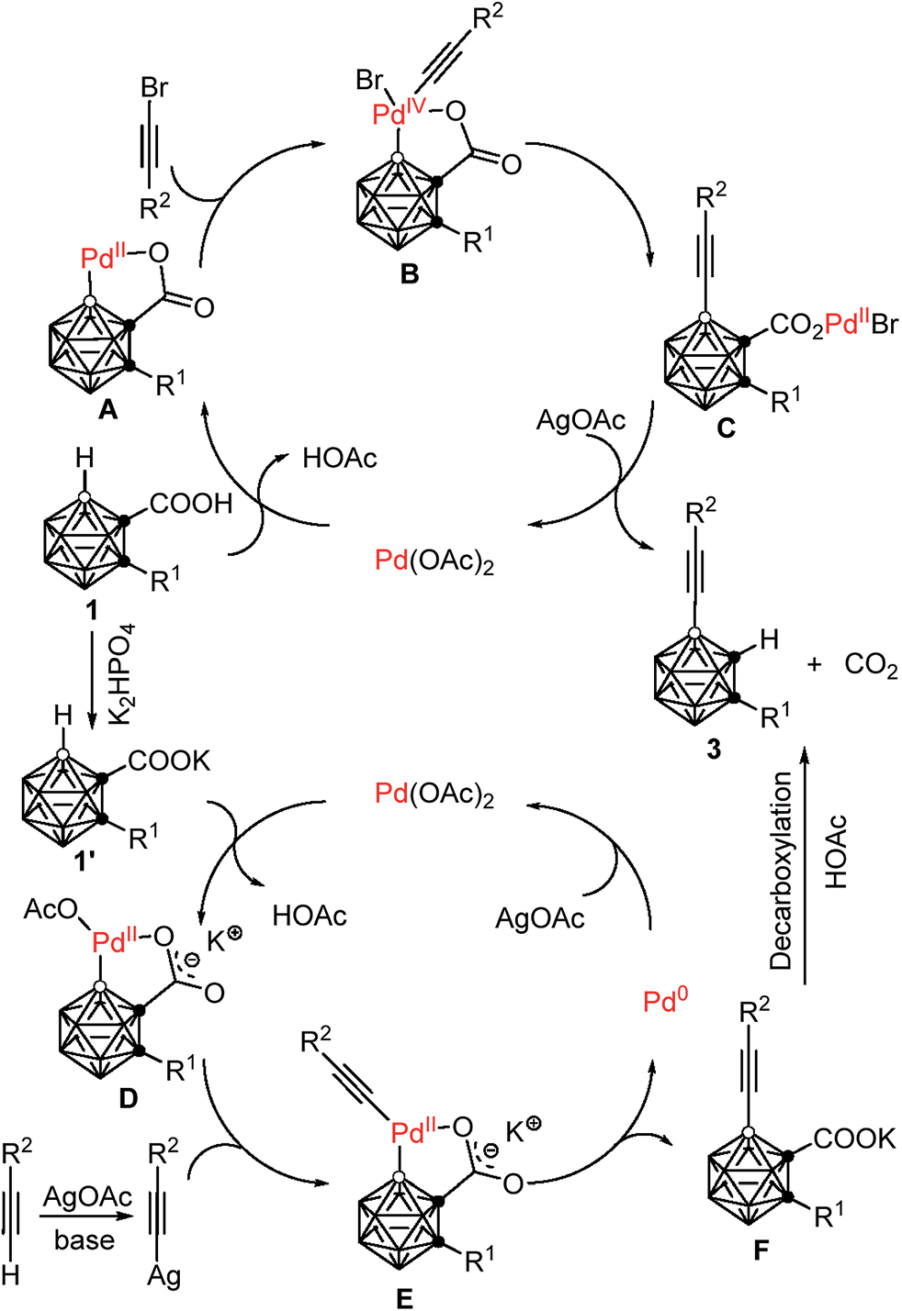
Proposed reaction mechanism.

On the basis of the aforementioned experimental data, two plausible reaction mechanisms are proposed in Scheme 4. For the Pd(ii)–Pd(iv)–Pd(ii) catalytic cycle: an exchange reaction of **1** with Pd(OAc)_2_, followed by regioselective electrophilic attack at the more electron-rich cage B(4) site yields the intermediate **A** as the charge distribution on the cage follows the trend B(9,12) > B(8,10) > B(4,5,7,11) > B(3,6).^32^ Oxidative addition of R^2^C≡CBr affords a Pd(iv) intermediate **B**.^25^,^33^ Reductive elimination produces the intermediate **C**, which undergoes a salt metathesis reaction, protonation and decarboxylation to give the final product **3** and regenerates the catalyst Pd(OAc)_2_. Meanwhile, another catalytic system involves a Pd(ii)–Pd(0)–Pd(ii) cycle. An acid–base reaction between K_2_HPO_4_ and carboranyl carboxylic acid **1** gives the potassium salt **1′**.^34^ Coordination of the oxygen atom of **1′** to the Pd(ii) center, followed by subsequent regioselective electrophilic attack at the more electron-rich cage B(4) site generates the intermediate **D**. Ligand exchange by acetylide gives a carboranyl-palladium acetylide intermediate **E**.21b,^35^ Reductive elimination affords the cage B(4)-alkynylated intermediate **F** and Pd(0). Decarboxylation of **F** results in the formation of the final product **3**, meanwhile Pd(0) is oxidized by AgOAc to regenerate Pd(OAc)_2_. It is noted that AgOAc acts as a bromide captor in the Pd(ii)–Pd(iv)–Pd(ii) catalytic cycle, but as an oxidant to regenerate Pd(ii) from Pd(0) in the Pd(ii)–Pd(0)–Pd(ii) catalytic cycle. However, in both cross-coupling reactions, AgOAc plays a crucial role in promoting decarboxylation and thereby controlling the mono-selectivity.

## Conclusion

We have developed two catalytic systems for regioselective and efficient alkynylation of cage B(4)–H bonds in *o*-carboranes using alkynyl bromides or terminal alkynes as alkynylating agents, where –COOH acts as a traceless directing group. A series of new cage B(4)-alkynylated *o*-carborane derivatives has been prepared for the first time, which could find many applications in the synthesis of carborane-based materials.^3^–^7^ This opens up a new window for the functionalization of carboranes by direct oxidative coupling of the cage B–H and organic C–H bonds. This work also offers a useful reference for selective C–H alkynylation using carboxylic acid as a traceless directing group in other aromatic systems.

On the basis of control experiments and literature work, two catalytic cycles are proposed for the above two reactions: a Pd(ii)–Pd(iv)–Pd(ii) cycle for using alkynyl bromides as coupling agents and a Pd(ii)–Pd(0)–Pd(ii) cycle for employing terminal alkynes as coupling partners. The latter has a broader substrate scope than the former. This work also gives some hints for the development of new catalytic systems for the functionalization of carboranes.

## Supplementary Material

Supplementary information

Crystal structure data
