# Supplementary material for: Palladium catalyzed regioselective B–C(sp) coupling via direct cage B–H activation: synthesis of B(4)-alkynylated o-carboranes
Source: Chem Sci. 2016 May 13;7(9):5838–45. doi: 10.1039/c6sc00901h (PMC6024241; doi:10.1039/c6sc00901h)
Supplement: Supplementary file 1 [file SC-007-C6SC00901H-s001.pdf]

Electronic Supplementary Material (ESI) for Chemical Science.
This journal is © The Royal Society of Chemistry 2016

Electronic Supplementary Information

Palladium Catalyzed Regioselective B-C(sp) Coupling via Direct Cage B–H Activation: Synthesis of B(4)-Alkynylated *o*-Carboranes

Yangjian Quan, Cen Tang, and Zuowei Xie*

Department of Chemistry and State Key Laboratory of Synthetic Chemistry, The Chinese University of Hong Kong, Shatin, New Territories, Hong Kong, China

E-mail: zxie@cuhk.edu.hk

Table of Contents

General Procedures S2

Experimental Section S2

Control Experiments S12

Transformations of **3a** S15

Crystal Data and Summary of Data Collection and Refinement S19

References S20

NMR Spectra S21

General Procedures. All reactions were carried out under an atmosphere of dry argon with the rigid exclusion of air and moisture using standard Schlenk techniques or in a glovebox unless otherwise specified. ^{1}H, ^{13}C and ^{11}B NMR spectra were recorded on a Bruker DPX 400 spectrometer at 400 MHz, 100 MHz and 128 MHz, respectively. All chemical shifts were reported in δ units with references to the residual solvent resonances of the deuterated solvents for proton and carbon chemical shifts, and to external $BF_3{\cdot}OEt_2$ (0.00 ppm) for boron chemical shifts. The data were reported as follows: chemical shift, multiplicity (s = singlet, d = doublet, t = triplet, q = quadruplet, m = multiplet or unresolved, br = broad), coupling constant(s) in Hz, integration, and assignment. Mass spectra were obtained on a Thermo Finnigan MAT 95 XL spectrometer. Compounds **1a-e**,[1] **2**,[2] and 1-Ar-*o*-carborane[3] were prepared according to literature methods. All organic solvents were freshly distilled from Na-K alloy or CaH_2 immediately prior to use. All other chemicals were purchased from either Aldrich or Acros Chemical Co. and used as received unless otherwise specified.

Preparation of *o*-Carboranyl Monocarboxylic Acid (1). A Representative Procedure. A diethyl ether solution (20 mL) of 1-R-*o*-carborane (5.0 mmol) was cooled to -78 ^{o}C, to which was slowly added $^{n}BuLi$ (5.0 mmol, 1.6 M in hexane, 3.2 mL). The resulting solution was stirred for 1 h at -78 ^{o}C. Dry ice (775 mg, 34.1 mmol) was crushed into small pieces and added immediately to the reaction mixture, which was stirred for an additional 1 h and then warmed to room temperature. After removal of ether and addition of water (10 mL), the resultant solution was extracted with hexane (10 x 2 mL) to recover the unreacted 1-R-*o*-carborane. The aqueous layer was acidified with 3 M HCl. Then the resultant solution

was extracted with hexane (10 x 3 mL). The hexane solutions were combined and dried with anhydrous Na_2SO_4. Removal of hexane gave **1** as a white crystalline solid.

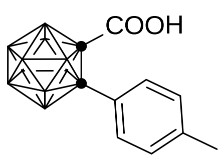

1f: Yield 90%. White solid. ^{1}H NMR (400 MHz, CD_2Cl_2): δ 8.50 (s, 1H) (COO*H*), 7.53 (d, *J* = 8.4 Hz, 2H), 7.16 (d, *J* = 8.4 Hz, 2H) (Ar), 2.35 (s, 3H) (CH_3). ^{13}C{^{1}H} NMR (100 MHz, CD_2Cl_2): δ 159.9 (*C*OOH), 142.0, 131.1, 129.7, 128.1 (Ar), 84.7, 76.1 (cage *C*), 21.2 (*C*H_3). ^{11}B{^{1}H} NMR (128 MHz, CD_2Cl_2) : δ 0.8 (1B), -2.3 (1B), -8.5 (8B). Anal. Calcd for $C_{10}H_{18}B_{10}O_2$: C, 43.15; H, 6.52. Found: C, 43.07; H, 6.73.

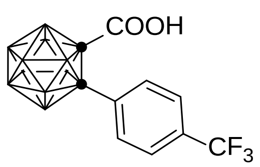

1h: Yield 85%. White solid. ^{1}H NMR (400 MHz, CD_2Cl_2): δ 7.92 (s, 1H) (COO*H*), 7.82 (d, *J* = 8.4 Hz, 2H), 7.64 (d, *J* = 8.4 Hz, 2H) (Ar). ^{13}C{^{1}H} NMR (100 MHz, CD_2Cl_2): δ 159.5 (*C*OOH), 134.7, 132.9 (q, $^{2}J_{C\text{-}F}$ = 34 Hz), 131.9, 126.0 (q, $^{3}J_{C\text{-}F}$ = 4 Hz) (Ar), 123.9 (q, $^{1}J_{C\text{-}F}$ = 270 Hz) (*C*F_3), 82.2, 75.9 (cage *C*). ^{11}B{^{1}H} NMR (128 MHz, CD_2Cl_2) : δ 0.9 (1B), -1.7 (1B), -8.1 (4B), -9.1 (2B), -9.9 (2B). Anal. Calcd for $C_{10}H_{15}B_{10}O_2F_3$: C, 36.14; H, 4.55. Found: C, 35.76; H, 4.86.

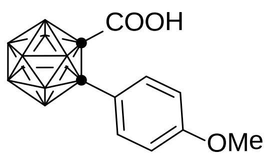

1j: Yield 88%. White solid. ^{1}H NMR (400 MHz, CD_2Cl_2): δ 8.23 (s, 1H) (COO*H*), 7.58 (d, *J* = 8.8 Hz, 2H), 6.84 (d, *J* = 8.8 Hz, 2H) (Ar), 3.81 (s, 3H) (OCH_3). ^{13}C{^{1}H} NMR (100 MHz, CD_2Cl_2): δ 162.0 (*C*OOH), 160.1, 132.8, 123.0, 114.2 (Ar), 85.0, 76.3 (cage *C*), 55.9 (O*C*H_3). ^{11}B{^{1}H} NMR (128 MHz, CD_2Cl_2) : δ 0.8 (1B), -2.7 (1B), -8.6 (8B). Anal. Calcd for $C_{10}H_{19}B_{10}O_{3.5}$ (**1j** + 0.5 H_2O): C, 39.59; H, 6.31. Found: C, 39.33; H, 6.51.

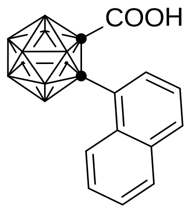

1k: Yield 80%. White solid. ^{1}H NMR (400 MHz, CD_2Cl_2): δ 8.96 (d, *J* = 8.4 Hz, 1H), 8.19 (d, *J* = 7.2 Hz, 1H), 7.97 (d, *J* = 7.6 Hz, 1H), 7.92 (d, *J* = 7.6

Hz, 1H), 7.64 (t, *J* = 8.0 Hz, 1H), 7.55 (t, *J* = 7.2 Hz, 1H), 7.42 (t, *J* = 7.2 Hz, 1H) (Ar), 6.25 (s, 1H) (COO*H*). ^{13}C{^{1}H} NMR (100 MHz, CD_2Cl_2): δ 160.2 (*C*OOH), 135.2, 134.6, 133.7, 131.7, 130.1, 127.6, 126.5, 125.9, 125.2, 124.8 (Ar), 86.5, 77.8 (cage *C*). ^{11}B{^{1}H} NMR (128 MHz, CD_2Cl_2) : δ 0.3 (1B), -0.8 (1B), -8.0 (5B), -8.9 (3B). Anal. Calcd for $C_{13}H_{18}B_{10}O_2$: C, 49.66; H, 5.77. Found: C, 49.55; H, 5.87.

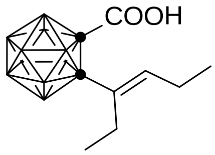

1m: Yield 88%. White solid. ^{1}H NMR (400 MHz, CD_2Cl_2): δ 9.24 (s, 1H) (COO*H*), 6.09 (t, *J* = 7.2 Hz, 1H) (olefinic), 2.26 (q, *J* = 7.6 Hz, 2H), 2.11 (m, 2H) (C*H*$_2$), 0.98 (m, 6H) (C*H*$_3$). ^{13}C{^{1}H} NMR (100 MHz, CD_2Cl_2): δ 161.7 (*C*OOH), 143.1, 132.1 (olefinic), 87.7, 76.4 (cage *C*), 25.6, 22.8 (*C*H$_2$), 14.1, 13.5 (*C*H$_3$). ^{11}B{^{1}H} NMR (128 MHz, CD_2Cl_2) : δ -0.0 (1B), -2.9 (1B), -8.7 (4B), -9.9 (4B). Anal. Calcd for $C_9H_{22}B_{10}O_2$: C, 39.98; H, 8.20. Found: C, 40.13; H, 8.14.

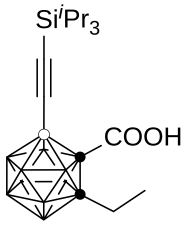

3b-COOH**:** Yield 78%. White solid. ^{1}H NMR (400 MHz, CD_2Cl_2): δ 10.09 (s, 1H) (COO*H*), 2.42 (q, *J* = 7.6 Hz, 2H), 1.15 (t, *J* = 7.6 Hz, 3H) (Et), 1.05 (m, 21H) (^{i}Pr$_3$Si). ^{13}C{^{1}H} NMR (100 MHz, CD_2Cl_2): δ 161.4 (*C*OOH), 82.9, 74.7 (cage *C*), 30.5 (*C*H$_2$), 18.7 (^{i}Pr$_3$Si), 14.2 (*C*H$_3$), 11.5 (^{i}Pr$_3$Si) (alkynyl carbons were not observed). ^{11}B{^{1}H} NMR (128 MHz, CD_2Cl_2) : δ -0.4 (1B), -2.9 (1B), -9.3 (8B). Anal. Calcd for $C_{16}H_{36}B_{10}O_2Si$: C, 48.45; H, 9.15. Found: C, 48.32; H, 9.21.

Preparation of B(4)-Alkynylated *o*-Carboranes (3). Representative Procedure (A). 1-COOH-2-R^1-*o*-carborane **1** (0.20 mmol), R^2–≡–Br **2** (0.20 mmol), $Pd(OAc)_2$ (0.01 mmol), and AgOAc (0.60 mmol) were mixed in DCE (5 mL). The resulting mixture was heated in a closed flask at 90 ^{o}C for 6 h. After hydrolysis with water (10 mL) and extraction with diethyl ether (10 mL x 3), the ether solutions were combined and concentrated to dryness in vacuo.

The residue was subjected to flash column chromatography on silica gel (230-400 mesh) using *n*-hexane as eluent to give the product **3**.

Representative Procedure (B). A toluene suspension (5 mL) of 1-COOH-2-R^1-*o*-carborane **1** (0.20 mmol), K_2HPO_4 (0.40 mmol), $Pd(OAc)_2$ (0.01 mmol) and AgOAc (0.60 mmol) was heated at 80 °C, to which was slowly added a toluene solution (5 mL) of R^2−≡−H **2** (0.40 mmol) by a syringe pump over a period of 10 h. After hydrolysis with water (10 mL) and extraction with diethyl ether (10 mL x 3), the ether solutions were combined and concentrated to dryness in vacuo. The residue was subjected to flash column chromatography on silica gel (230-400 mesh) using *n*-hexane as eluent to give the product **3**.

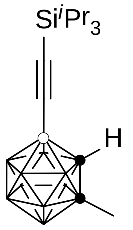

3a: Yield 81% (**A**), 79% (**B**). Colorless oil. ^{1}H NMR (400 MHz, $CDCl_3$): δ 3.76 (s, 1H) (cage *H*), 2.05 (s, 3H) (CH_3), 1.06 (m, 21H) (^{i}Pr$_3$Si). ^{13}C{^{1}H} NMR (100 MHz, $CDCl_3$): δ 70.2, 63.0 (cage *C*), 26.0 (*C*H$_3$), 18.7, 11.3 (^{i}Pr$_3$Si) (alkynyl carbons were not observed). ^{11}B{^{1}H} NMR (128 MHz, $CDCl_3$): δ -2.7 (1B), -6.4 (1B), -9.0 (1B), -10.1 (1B), -11.7 (5B), -13.2 (1B). HRMS: *m/z* calcd for $C_{14}H_{34}B_{10}Si$ $[M]^+$: 338.3436. Found: 338.3437.

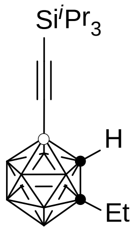

3b: Yield 76% (**A**). Colorless oil. ^{1}H NMR (400 MHz, $CDCl_3$): δ 3.74 (s, 1H) (cage *H*), 2.30 (q, *J* = 7.6 Hz, 2H) (CH_2), 1.10 (t, *J* = 7.6 Hz, 3H) (CH_3), 1.06 (m, 21H) (^{i}Pr$_3$Si). ^{13}C{^{1}H} NMR (100 MHz, $CDCl_3$): δ 76.1, 62.4 (cage *C*), 31.8 (*C*H$_2$), 18.7 (^{i}Pr$_3$Si), 13.7 (*C*H$_3$), 11.3 (^{i}Pr$_3$Si) (alkynyl carbons were not observed). ^{11}B{^{1}H} NMR (128 MHz, $CDCl_3$): δ -3.0 (1B), -5.3 (1B), -8.9 (1B), -10.1 (1B), -12.1 (5B), -14.0 (1B). HRMS: *m/z* calcd for $C_{15}H_{36}B_{10}Si$ $[M]^+$: 352.3593. Found: 352.3592.

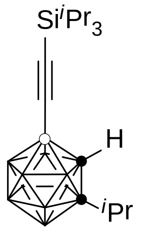

3c: Yield 75% (**A**), 86% (**B**). Colorless oil. ^{1}H NMR (400 MHz, CD_2Cl_2): δ 3.90 (s, 1H) (cage *H*), 2.52 (m, 1H) (^{i}Pr), 1.14 (d, *J* = 6.8 Hz, 6H), 1.08 (m, 21H) (^{i}Pr$_3$Si). ^{13}C{^{1}H} NMR (100 MHz, CD_2Cl_2): δ 82.0, 62.4 (cage *C*), 35.3, 23.1 (^{i}Pr), 18.8, 11.6 (^{i}Pr$_3$Si) (alkynyl carbons were not observed). ^{11}B{^{1}H} NMR (128 MHz, CD_2Cl_2): δ -4.1 (1B), -5.1 (1B), -9.3 (1B), -10.4 (1B), -12.6 (4B), -14.0 (2B). HRMS: *m*/*z* calcd for $C_{16}H_{38}B_{10}Si$ $[M]^+$: 366.3750. Found: 366.3750.

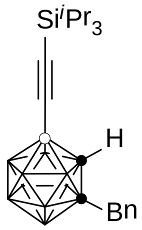

3d: Yield 73% (**A**), 70% (**B**). Colorless oil. ^{1}H NMR (400 MHz, CD_2Cl_2): δ 7.37 (m, 3H), 7.17 (m, 2H) (Ar), 3.54 (s, 3H) (C*H*$_2$ & cage *H*), 1.05 (m, 21H) (^{i}Pr$_3$Si). ^{13}C{^{1}H} NMR (100 MHz, CD_2Cl_2): δ 134.9, 130.2, 129.4, 128.9 (Ar), 75.3, 62.0 (cage *C*), 43.9 (*C*H$_2$), 18.7, 11.6 (^{i}Pr$_3$Si) (alkynyl carbons were not observed). ^{11}B{^{1}H} NMR (128 MHz, CD_2Cl_2): δ -3.6 (1B), -5.7 (1B), -9.2 (1B), -10.2 (1B), -12.3 (3B), -13.1 (3B). HRMS: *m*/*z* calcd for $C_{20}H_{38}B_{10}Si$ $[M]^+$: 415.3722. Found: 415.3723.

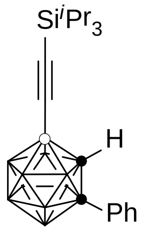

3e: Yield 77% (**A**). Colorless oil. ^{1}H NMR (400 MHz, CD_2Cl_2): δ 7.52 (d, *J* = 7.6 Hz, 2H), 7.40 (m, 3H) (Ar), 4.25 (s, 1H) (cage *H*), 1.08 (m, 21H) (^{i}Pr$_3$Si). ^{13}C{^{1}H} NMR (100 MHz, CD_2Cl_2): δ 133.5, 130.5, 129.3, 128.1 (Ar), 77.1, 62.4 (cage *C*), 18.8, 11.6 (^{i}Pr$_3$Si) (alkynyl carbons were not observed). ^{11}B{^{1}H} NMR (128 MHz, CD_2Cl_2): δ -3.2 (1B), -4.4 (1B), -9.1 (1B), -10.1 (1B), -12.1 (5B), -13.3 (1B). HRMS: *m*/*z* calcd for $C_{19}H_{36}B_{10}Si$ $[M]^+$: 401.3564. Found: 401.3568.

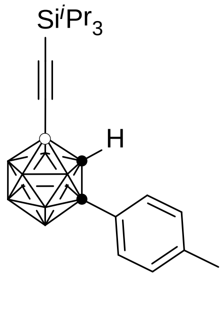

3f: Yield 82% (**A**). Colorless oil. ^{1}H NMR (400 MHz, CD_2Cl_2): δ 7.39 (d, *J* = 8.4 Hz, 2H), 7.16 (d, *J* = 8.4 Hz, 2H) (Ar), 4.20 (s, 1H) (cage *H*), 2.34 (s, 3H) (C*H*$_3$), 1.08 (m, 21H) (^{i}Pr$_3$Si). ^{13}C{^{1}H} NMR (100 MHz, CD_2Cl_2): δ 141.0, 130.6, 129.9, 128.0 (Ar), 77.3, 62.6 (cage *C*), 21.1 (*C*H$_3$), 18.8,

11.6 (^{i}Pr$_3$Si) (alkynyl carbons were not observed). ^{11}B{^{1}H} NMR (128 MHz, CD_2Cl_2): δ -3.9 (1B), -5.3 (1B), -9.9 (1B), -10.9 (1B), -12.8 (5B), -14.0 (1B). HRMS: *m*/*z* calcd for $C_{20}H_{38}B_{10}Si$ [M]$^+$: 415.3722. Found: 415.3720.

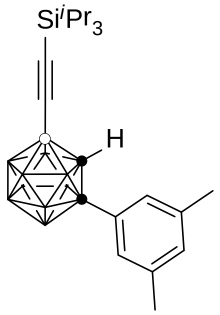

3g: Yield 70% (**A**), 30% (**B**). Colorless oil. ^{1}H NMR (400 MHz, CD_2Cl_2): δ 7.11 (s, 2H), 7.05 (s, 1H) (Ar), 4.22 (s, 1H) (cage *H*), 2.32 (s, 6H) (C*H*$_3$), 1.09 (m, 21H) (^{i}Pr$_3$Si). ^{13}C{^{1}H} NMR (100 MHz, CD_2Cl_2): δ 139.3, 133.5, 132.1, 125.9 (Ar), 77.5, 62.4 (cage *C*), 21.4 (*C*H$_3$), 18.8, 11.6 (^{i}Pr$_3$Si) (alkynyl carbons were not observed). ^{11}B{^{1}H} NMR (128 MHz, CD_2Cl_2): δ -2.9 (1B), -4.0 (1B), -8.7 (1B), -9.8 (1B), -11.8 (5B), -13.1 (1B). HRMS: *m*/*z* calcd for $C_{21}H_{40}B_{10}Si$ [M]$^+$: 429.3879. Found: 429.3875.

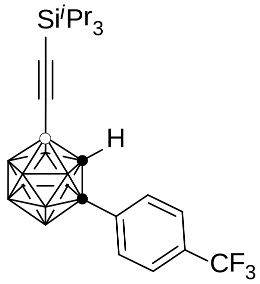

3h: Yield 81% (**A**). Colorless oil. ^{1}H NMR (400 MHz, CD_2Cl_2): δ 7.65 (m, 4H) (Ar), 4.27 (s, 1H) (cage *H*), 1.08 (m, 21H) (^{i}Pr$_3$Si). ^{13}C{^{1}H} NMR (100 MHz, $CDCl_3$): δ 131.6, 128.4 (q, $^2J_{C-F}$ = 36 Hz), 128.3, 126.2 (q, $^1J_{C-F}$ = 273 Hz) (*C*F$_3$), 126.1 (q, $^3J_{C-F}$ = 4 Hz) (Ar), 74.9, 61.4 (cage *C*), 18.7, 11.3 (^{i}Pr$_3$Si) (alkynyl carbons were not observed). ^{11}B{^{1}H} NMR (128 MHz, CD_2Cl_2): δ -3.0 (1B), -3.6 (1B), -9.4 (3B), -11.9 (4B), -13.0 (1B). HRMS: *m*/*z* calcd for $C_{20}H_{35}B_{10}SiF_3$ [M]$^+$: 469.3439. Found: 469.3435.

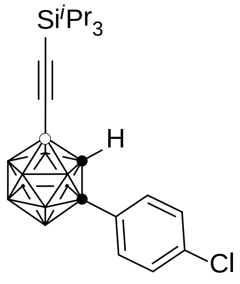

3i: Yield 72% (**A**). Colorless oil. ^{1}H NMR (400 MHz, CD_2Cl_2): δ 7.47 (d, *J* = 8.8 Hz, 2H), 7.35 (d, *J* = 8.8 Hz, 2H) (Ar), 4.20 (s, 1H) (cage *H*), 1.07 (m, 21H) (^{i}Pr$_3$Si). ^{13}C{^{1}H} NMR (100 MHz, CD_2Cl_2): δ 136.9, 132.0, 129.7, 129.4 (Ar), 76.1, 62.5 (cage *C*), 18.8, 11.6 (^{i}Pr$_3$Si) (alkynyl carbons were not observed). ^{11}B{^{1}H} NMR (128 MHz, CD_2Cl_2): δ -2.7 (1B), -3.8 (1B), -8.6

(1B), -9.6 (1B), -11.6 (6B). HRMS: *m*/*z* calcd for $C_{19}H_{35}B_{10}SiCl$ $[M]^+$: 434.3200. Found: 434.3202.

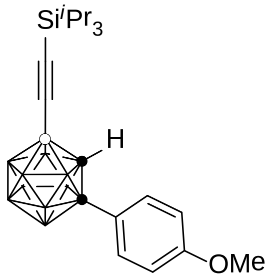

3j: Yield 78% (**A**). Colorless oil. ^{1}H NMR (400 MHz, CD_2Cl_2): δ 7.45 (d, *J* = 8.8 Hz, 2H), 6.85 (d, *J* = 8.8 Hz, 2H) (Ar), 4.16 (s, 1H) (cage *H*), 3.80 (s, 3H) (OC*H*$_3$), 1.08 (m, 21H) (^{i}Pr$_3$Si). ^{13}C{^{1}H} NMR (100 MHz, CD_2Cl_2): δ 161.4, 129.8, 125.4, 114.4 (Ar), 77.5, 63.2 (cage *C*), 55.9 (O*C*H$_3$), 18.8, 11.6 (^{i}Pr$_3$Si) (alkynyl carbons were not observed). ^{11}B{^{1}H} NMR (128 MHz, CD_2Cl_2): δ -3.0 (1B), -4.6 (1B), -9.1 (1B), -10.2 (1B), -12.0 (6B). HRMS: *m*/*z* calcd for $C_{20}H_{38}B_{10}SiO$ $[M]^+$: 431.3671. Found: 431.3664.

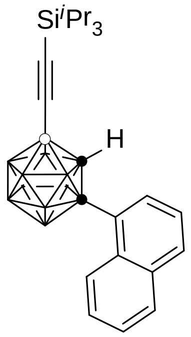

3k: Yield 40% (**A**). Colorless oil. ^{1}H NMR (400 MHz, CD_2Cl_2): δ 8.71 (d, *J* = 8.8 Hz, 1H), 7.93 (t, *J* = 7.2 Hz, 2H), 7.86 (d, *J* = 7.6 Hz, 1H), 7.61 (t, *J* = 7.6 Hz, 1H), 7.55 (t, *J* = 7.2 Hz, 1H), 7.45 (t, *J* = 7.6 Hz, 1H) (Ar), 4.90 (s, 1H) (cage *H*), 1.10 (m, 21H) (^{i}Pr$_3$Si). ^{13}C{^{1}H} NMR (100 MHz, CD_2Cl_2): δ 135.2, 132.4, 130.2, 129.1, 127.7, 126.5, 125.0, 124.6 (Ar), 78.0, 63.5 (cage *C*), 18.8, 11.6 (^{i}Pr$_3$Si) (alkynyl carbons were not observed). ^{11}B{^{1}H} NMR (128 MHz, CD_2Cl_2): δ -2.6 (2B), -9.6 (5B), -12.2 (3B). HRMS: *m*/*z* calcd for $C_{23}H_{38}B_{10}Si$ $[M]^+$: 451.3724. Found: 451.3723.

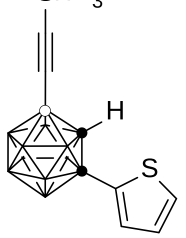

3l: Yield 54% (**A**). Colorless oil. ^{1}H NMR (400 MHz, CD_2Cl_2): δ 7.32 (d, *J* = 5.2 Hz, 1H), 7.24 (d, *J* = 3.6 Hz, 1H), 6.94 (t, *J* = 4.4 Hz, 1H) (Ar), 4.15 (s, 1H) (cage *H*), 1.08 (m, 21H) (^{i}Pr$_3$Si). ^{13}C{^{1}H} NMR (100 MHz, CD_2Cl_2): δ 136.8, 130.7, 128.7, 127.8 (Ar), 72.5, 65.3 (cage *C*), 18.8, 11.6 (^{i}Pr$_3$Si) (alkynyl carbons were not observed). ^{11}B{^{1}H} NMR (128 MHz, CD_2Cl_2): δ -2.4 (1B), -4.4

(1B), -9.2(1B), -10.4 (1B), -10.8 (2B), -11.9 (4B). HRMS: *m/z* calcd for $C_{17}H_{34}B_{10}SiS$ $[M]^+$: 407.3128. Found: 407.3129.

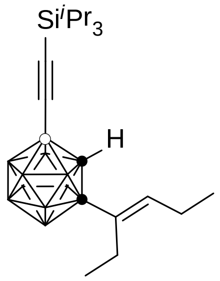

3m: Yield 80% (**A**). Colorless oil. ^{1}H NMR (400 MHz, $CDCl_3$): δ 5.72 (t, *J* = 7.2 Hz, 1H) (olefinic), 3.89 (s, 1H) (cage *H*), 2.20 (q, *J* = 7.6 Hz, 2H), 2.07 (m, 2H) (CH_2), 1.07 (m, 21H) (^{i}Pr$_3$Si), 1.01 (t, *J* = 7.6 Hz, 6H) (CH_3). ^{13}C{^{1}H} NMR (100 MHz, $CDCl_3$): δ 136.2, 134.0 (olefinic), 78.8, 61.7 (cage *C*), 24.2, 22.0 (*C*H_2), 18.7 (^{i}Pr$_3$Si), 13.9, 13.7 (*C*H_3), 11.3 (^{i}Pr$_3$Si) (alkynyl carbons were not observed). ^{11}B{^{1}H} NMR (128 MHz, $CDCl_3$): δ -3.4 (1B), -4.2 (1B), -9.0 (1B), -10.1 (1B), -12.4 (6B). HRMS: *m/z* calcd for $C_{19}H_{42}B_{10}Si$ $[M]^+$: 407.4034. Found: 407.4030.

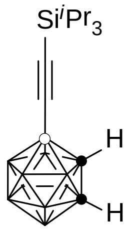

3o: Yield 41% (**A**), 74% (**B**). Colorless oil. ^{1}H NMR (400 MHz, CD_2Cl_2): δ 3.85 (s, 1H), 3.70 (s, 1H) (cage *H*), 1.07 (m, 21H) (^{i}Pr$_3$Si). ^{13}C{^{1}H} NMR (100 MHz, CD_2Cl_2): δ 56.7, 55.0 (cage *C*), 18.8, 11.6 (^{i}Pr$_3$Si) (alkynyl carbons were not observed). ^{11}B{^{1}H} NMR (128 MHz, CD_2Cl_2): δ -2.3 (1B), -3.5 (1B), -9.1(1B), -10.3 (1B), -12.7 (1B), -13.3 (1B), -14.3 (2B), -15.3 (1B), -16.4 (1B). HRMS: *m/z* calcd for $C_{13}H_{32}B_{10}Si$ $[M]^+$: 324.3276. Found: 324.3280.

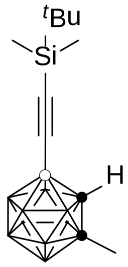

3p: Yield 70% (**A**), 72% (**B**). Colorless oil. ^{1}H NMR (400 MHz, $CDCl_3$): δ 3.77 (s, 1H) (cage *H*), 2.05 (s, 3H) (CH_3), 0.93 (s, 9H) (^{t}Bu), 0.10 (s, 6H) (CH_3). ^{13}C{^{1}H} NMR (100 MHz, $CDCl_3$): δ 70.3, 63.0 (cage *C*), 26.2 (^{t}Bu), 26.0, 16.8 (*C*H_3) (alkynyl carbons were not observed). ^{11}B{^{1}H} NMR (128 MHz, $CDCl_3$): δ -2.4 (1B), -6.2 (1B), -8.7 (1B), -9.8 (1B), -11.7 (6B). HRMS: *m/z* calcd for $C_{11}H_{28}B_{10}Si$ $[M]^+$: 296.2965. Found: 296.2961.

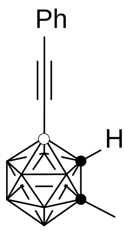

3r: Yield 52% (**B**). Colorless oil. ^{1}H NMR (400 MHz, $CDCl_3$): δ 7.46 (d, *J* = 7.6 Hz, 2H), 7.30 (m, 3H) (Ar), 3.86 (s, 1H) (cage *H*), 2.07 (s, 3H) (C*H*$_3$). ^{13}C{^{1}H} NMR (100 MHz, $CDCl_3$): δ 132.2, 128.7, 128.4, 123.0 (Ar), 70.3, 62.9 (cage *C*), 26.1 (*C*H$_3$) (alkynyl carbons were not observed). ^{11}B{^{1}H} NMR (128 MHz, $CDCl_3$): δ -0.7 (1B), -4.6 (1B), -7.2 (1B), -8.2 (1B), -10.1 (5B), -11.4 (1B). HRMS: *m*/*z* calcd for $C_{11}H_{18}B_{10}$ $[M]^+$: 258.2412. Found: 258.2413.

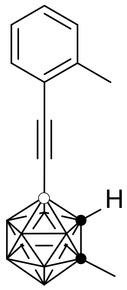

3s: Yield 65% (**B**). Colorless oil. ^{1}H NMR (400 MHz, $CDCl_3$): δ 7.41 (d, *J* = 7.6 Hz, 1H), 7.19 (m, 2H), 7.11 (t, *J* = 7.6 Hz, 1H) (Ar), 3.85 (s, 1H) (cage *H*), 2.43 (s, 3H), 2.07 (s, 3H) (C*H*$_3$). ^{13}C{^{1}H} NMR (100 MHz, $CDCl_3$): δ 141.1, 132.2, 129.5, 128.7, 125.6, 122.8 (Ar), 70.4, 62.9 (cage *C*), 26.1, 20.8 (*C*H$_3$) (alkynyl carbons were not observed). ^{11}B{^{1}H} NMR (128 MHz, $CDCl_3$): δ -2.7 (1B), -6.4 (1B), -8.9 (1B), -10.0 (1B), -11.9 (4B), -13.4 (2B). HRMS: *m*/*z* calcd for $C_{12}H_{20}B_{10}$ $[M]^+$: 272.2568. Found: 272.2566.

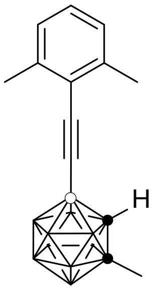

3t: Yield 73% (**B**). Colorless oil. ^{1}H NMR (400 MHz, $CDCl_3$): δ 7.10 (t, *J* = 7.6 Hz, 1H), 7.01 (d, *J* = 7.6 Hz, 2H) (Ar), 3.84 (s, 1H) (cage *H*), 2.42 (s, 6H), 2.08 (s, 3H) (C*H*$_3$). ^{13}C{^{1}H} NMR (100 MHz, $CDCl_3$): δ 141.0, 128.1, 126.7, 122.8 (Ar), 70.3, 62.9 (cage *C*), 26.1, 21.1 (*C*H$_3$) (alkynyl carbons were not observed). ^{11}B{^{1}H} NMR (128 MHz, $CDCl_3$): δ -1.3 (1B), -5.1 (1B), -7.7 (1B), -8.9 (1B), -10.2 (4B), -12.2 (2B). HRMS: *m*/*z* calcd for $C_{13}H_{22}B_{10}$ $[M]^+$: 286.2725. Found: 286.2730.

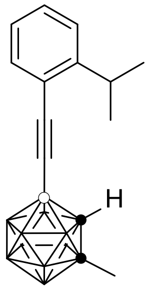

3u: Yield 80% (**B**). Colorless oil. ^{1}H NMR (400 MHz, $CDCl_3$): δ 7.42 (d, *J* = 7.2 Hz, 1H), 7.29 (m, 2H), 7.13 (t, *J* = 7.6 Hz, 1H) (Ar), 3.84 (s, 1H) (cage *H*), 3.42 (m, 1H) (^{i}Pr), 2.08 (s, 3H) (C*H*$_3$), 1.27 (d, *J* = 7.2 Hz, 6H) (^{i}Pr). ^{13}C{^{1}H}

NMR (100 MHz, $CDCl_3$): δ 151.4, 132.7, 129.1, 125.5, 125.0, 121.8 (Ar), 70.4, 62.8 (cage *C*), 31.8, 26.1, 23.0 (alkyl *C*) (alkynyl carbons were not observed). $^{11}B\{^1H\}$ NMR (128 MHz, $CDCl_3$): δ -2.6 (1B), -6.4 (1B), -9.0 (1B), -10.1 (1B), -11.5 (5B), -13.4 (1B). HRMS: *m*/*z* calcd for $C_{14}H_{24}B_{10}$ $[M]^+$: 300.2881. Found: 300.2882.

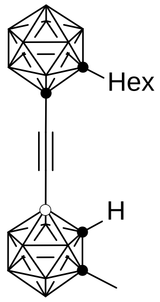

3v: Yield 82% (**B**). Colorless oil. ^1H NMR (400 MHz, $CDCl_3$): δ 3.81 (s, 1H) (cage *H*), 2.32 (m, 2H) (Hex), 2.06 (s, 3H) (CH_3), 1.53 (m, 2H), 1.30 (m, 6H), 0.89 (t, J = 6.8 Hz, 3H) (Hex). $^{13}C\{^1H\}$ NMR (100 MHz, $CDCl_3$): δ 82.3, 70.8, 66.8, 62.6 (cage *C*), 36.2, 31.5, 29.7, 29.0, 26.0, 22.7, 14.1 (alkyl *C*) (alkynyl carbons were not observed). $^{11}B\{^1H\}$ NMR (128 MHz, $CDCl_3$): δ -2.1 (1B), -3.2 (1B), -6.3 (2B), -9.8 (8B), -11.9 (7B), -13.5 (1B). HRMS: *m*/*z* calcd for $C_{13}H_{36}B_{20}$ $[M]^+$: 408.4823. Found: 408.4826.

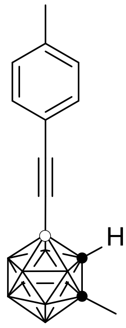

3w: Yield 48% (**B**). Colorless oil. ^1H NMR (400 MHz, $CDCl_3$): δ 7.35 (d, J = 7.6 Hz, 2H), 7.10 (d, J = 8.0 Hz, 2H) (Ar), 3.85 (s, 1H) (cage *H*), 2.34 (s, 3H), 2.07 (s, 3H) (CH_3). $^{13}C\{^1H\}$ NMR (100 MHz, $CDCl_3$): δ 138.9, 132.1, 129.1, 119.9 (Ar), 70.3, 62.9 (cage *C*), 26.1, 21.7 (CH_3) (alkynyl carbons were not observed). $^{11}B\{^1H\}$ NMR (128 MHz, $CDCl_3$): δ -2.3 (1B), -6.2 (1B), -8.8 (1B), -9.9 (1B), -11.6 (6B). HRMS: *m*/*z* calcd for $C_{12}H_{20}B_{10}$ $[M]^+$: 272.2568. Found: 272.2571.

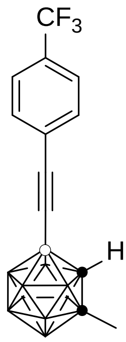

3x: Yield 44% (**B**). Colorless oil. ^1H NMR (400 MHz, $CDCl_3$): δ 7.56 (m, 4H) (Ar), 3.87 (s, 1H) (cage *H*), 2.08 (s, 3H) (CH_3). $^{13}C\{^1H\}$ NMR (100 MHz, $CDCl_3$): δ 132.4, 130.4 (q, $^1J_{C\text{-}F}$ = 272 Hz) (CF_3), 130.0 (q, $^2J_{C\text{-}F}$ = 33 Hz), 126.9, 125.3 (q, $^3J_{C\text{-}F}$ = 4 Hz) (Ar), 70.5, 62.8 (cage *C*), 26.1 (CH_3) (alkynyl carbons were not observed). $^{11}B\{^1H\}$ NMR (128 MHz, $CDCl_3$): δ -2.3 (1B), -6.3 (1B),

-8.9 (1B), -9.9 (1B), -11.9 (6B). HRMS: *m*/*z* calcd for $C_{12}H_{17}B_{10}F_3$ $[M]^+$: 327.2249. Found: 327.2250.

Control Experiments.

Reaction of 1a with 2 in the presence of Pd(dba)$_2$ without AgOAc. Method A: A DCE solution (5 mL) of **1a** (40.4 mg, 0.20 mmol), ^{i}Pr$_3$SiC≡CBr (52.3 mg, 0.20 mmol) and Pd(dba)$_2$ (23.0 mg, 0.04 mmol) was stirred at 90 ^{o}C for 6 h. **Method B**: To a toluene solution (5 mL) of **1a** (40.4 mg, 0.20 mmol), K_2HPO_4 (69.6 mg, 0.40 mmol), and Pd(dba)$_2$ (23.0 mg, 0.04 mmol) was slowly added ^{i}Pr$_3$SiC≡CH (72.8 mg, 0.40 mmol in 5 mL toluene) by a syringe pump over a period of 10 h at 80 ^{o}C. After hydrolysis with water, the organic portion was analyzed by ^{11}B NMR and GC-MS. The ^{11}B NMR showed that **1a** remained unchanged. GC-MS analyses indicated that only decarboxylation product 1-Me-*o*-carborane was present. Noted that **1a** decomposed into 1-Me-*o*-carborane under GC conditions, which was confirmed by an authenticated sample.

Reaction of 1a with 2 in the presence of Pd(OAc)$_2$ without AgOAc. Method A: A DCE solution (5 mL) of **1a** (40.4 mg, 0.20 mmol), ^{i}Pr$_3$SiC≡CBr (52.3 mg, 0.20 mmol) and Pd(OAc)$_2$ (9.0 mg, 0.04 mmol) was heated at 90 ^{o}C for 6 h. **Method B**: To a toluene solution (5 mL) of **1a** (40.4 mg, 0.20 mmol), K_2HPO_4 (69.6 mg, 0.40 mmol), and Pd(OAc)$_2$ (9.0 mg, 0.04 mmol) was slowly added ^{i}Pr$_3$SiC≡CH (72.8 mg, 0.40 mmol in 5 mL toluene) by a syringe pump over a period of 10 h at 80 ^{o}C. After hydrolysis with water, the organic portion was analyzed by ^{11}B NMR and GC-MS. The ^{11}B NMR showed that **3a** was formed in 25% (by **Method A**) and 14% (by **Method B**) NMR yield. GC-MS analyses indicated that **3a** was generated in 30% (by **Method A**) and 16% (by **Method B**) GC yield.

Decarboxylation Reactions. A DCE solution (0.5 mL) of carboranyl carboxylic acid (0.01 mmol) and additive was heated in a closed NMR tube at 90 °C, which was monitored by ^{11}B NMR spectra. The time-dependent ^{11}B NMR spectra were compiled below.

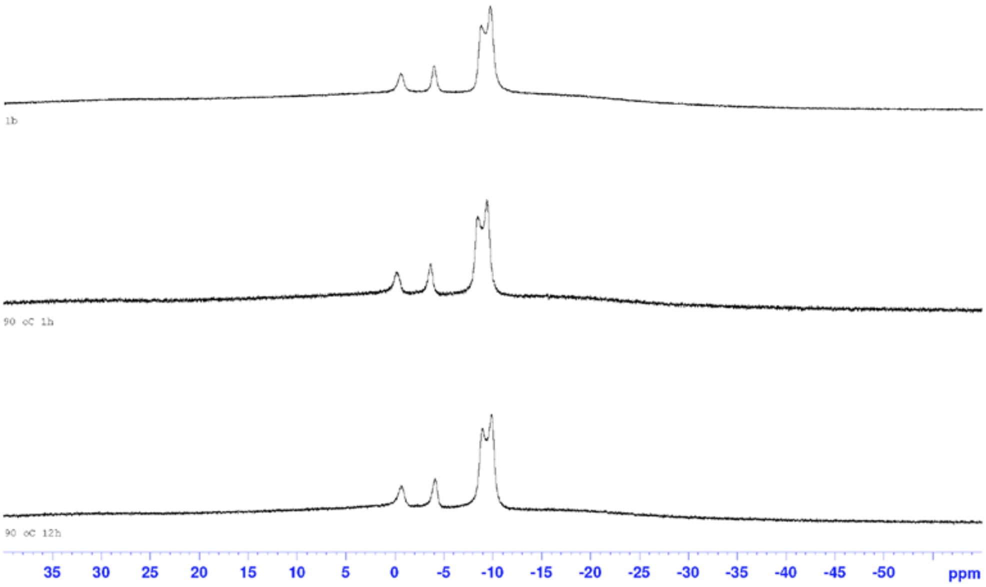

Figure S1. Time-Dependent ^{11}B NMR Spectra for Decarboxylation of **1b**

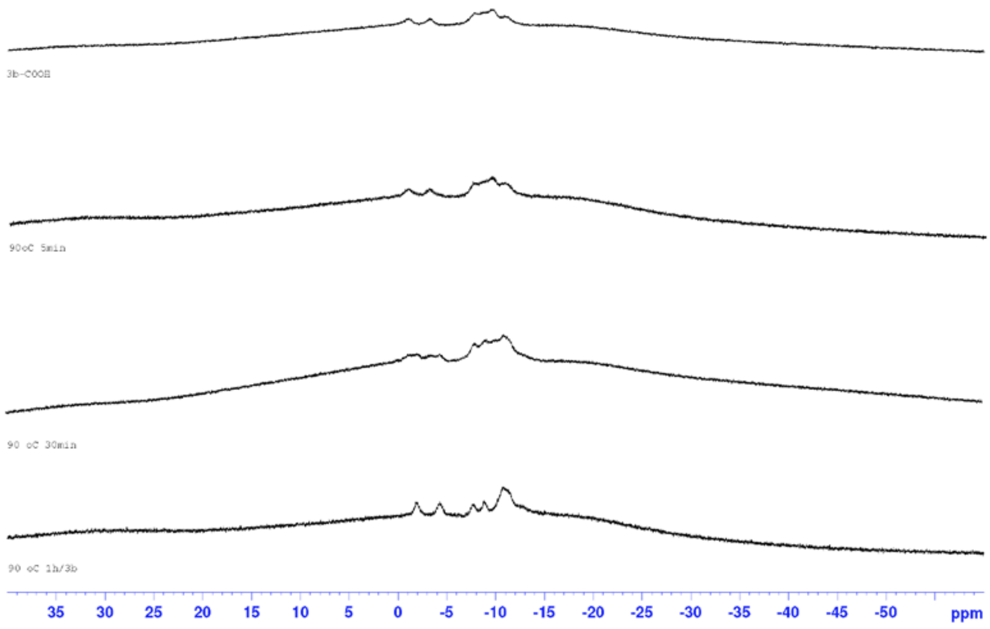

Figure S2. Time-Dependent ^{11}B NMR Spectra for Decarboxylation of **3b**-COOH

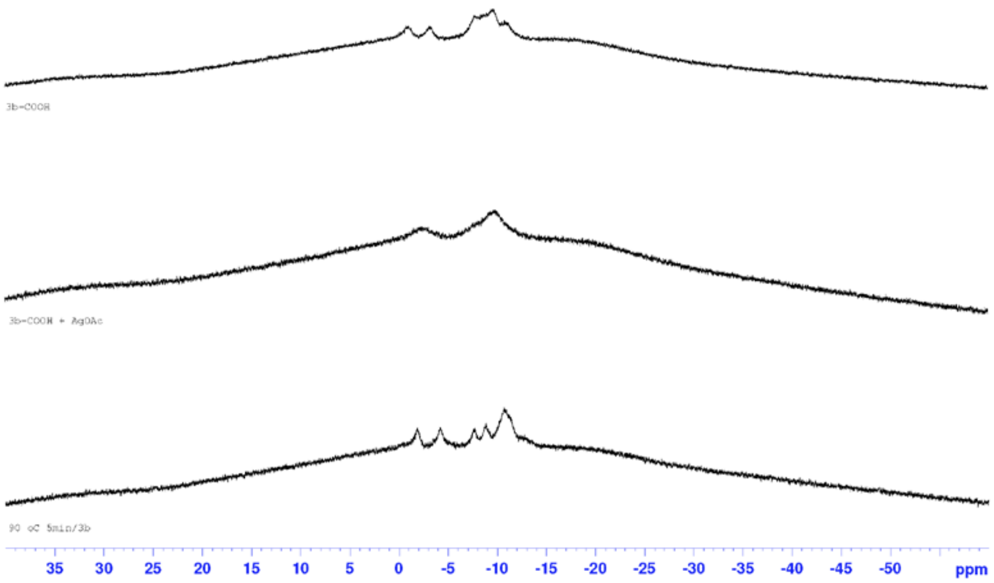

Figure S3. Time-Dependent ^{11}B NMR Spectra for Decarboxylation of **3b**-COOH in the presence of AgOAc

Transformations of 3a.

Desilylation of 3a. Compound **3a** (67.6 mg, 0.20 mmol) and TBAF (0.2 mL, 1M in THF) were mixed in THF (5 mL). The resulting mixture was stirred at 0 °C for 1 min. After hydrolysis with water (10 mL) and extraction with diethyl ether (10 mL x 3), the ether solutions were combined and concentrated to dryness in vacuo. The residue was subjected to flash column chromatography to give the product **4a** as colorless crystals (34.6 mg, 95%).

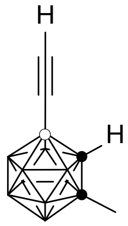

4a. ^{1}H NMR (400 MHz, $CDCl_3$): δ 3.81 (s, 1H) (cage *H*), 2.20 (s, 1H) (alkynyl), 2.06 (s, 3H) (C*H*$_3$). ^{13}C{^{1}H} NMR (100 MHz, $CDCl_3$): δ 85.4 (alkynyl), 70.5, 63.0 (cage *C*), 26.0 (*C*H$_3$) (one alkynyl carbons was observed, the other was not). ^{11}B{^{1}H} NMR (128 MHz, $CDCl_3$): δ -0.2 (1B), -4.2 (1B), -6.8 (1B), -7.8 (1B), -9.0 (2B), -9.8 (2B), -10.8 (2B). HRMS: *m*/*z* calcd for $C_5H_{14}B_{10}$ $[M]^+$: 182.2096. Found: 182.2094.

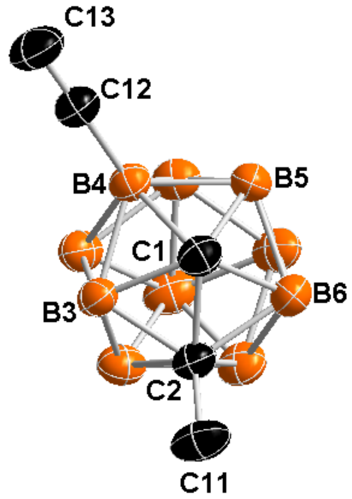

Figure S4. Molecular Structure of **4a**

Reaction of 4a with PhI. Compound **4a** (36.4 mg, 0.20 mmol), PhI (81.6 mg, 0.40 mmol), $PdCl_2(PPh_3)_2$ (7.0 mg, 0.01 mmol), CuI (3.8 mg, 0.02 mmol) and NEt_3 (1.0 mL) were mixed in toluene (4 mL). The resulting mixture was heated at 80 °C for 12 h. After hydrolysis with water (10 mL) and extraction with diethyl ether (10 mL x 3), the ether solutions were

combined and concentrated to dryness in vacuo. The residue was subjected to flash column chromatography to give the product **3r** as colorless oil (47.5 mg, 92%).

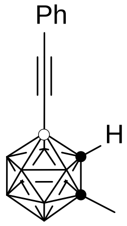

3r. ^{1}H NMR (400 MHz, $CDCl_3$): δ 7.46 (d, *J* = 7.6 Hz, 2H), 7.30 (m, 3H) (Ar), 3.86 (s, 1H) (cage *H*), 2.07 (s, 3H) (C*H*$_3$). ^{13}C{^{1}H} NMR (100 MHz, $CDCl_3$): δ 132.2, 128.7, 128.4, 123.0 (Ar), 70.3, 62.9 (cage *C*), 26.1 (*C*H$_3$) (alkynyl carbons were not observed). ^{11}B{^{1}H} NMR (128 MHz, $CDCl_3$): δ -0.7 (1B), -4.6 (1B), -7.2 (1B), -8.2 (1B), -10.1 (5B), -11.4 (1B). HRMS: *m*/*z* calcd for $C_{11}H_{18}B_{10}$ $[M]^+$: 258.2412. Found: 258.2413.

Reaction of 4a with 2-Bromothiophene. Compound **4a** (36.4 mg, 0.20 mmol), 2-bromothiophene (65.2 mg, 0.40 mmol), $PdCl_2(PPh_3)_2$ (7.0 mg, 0.01 mmol), CuI (3.8 mg, 0.02 mmol) and NEt_3 (1.0 mL) were mixed in toluene (4 mL). The resulting mixture was heated at 80 ^{o}C for 12 h. After hydrolysis with water (10 mL) and extraction with diethyl ether (10 mL x 3), the ether solutions were combined and concentrated to dryness in vacuo. The residue was subjected to flash column chromatography to give the product **5a** as colorless oil (47.6 mg, 90%).

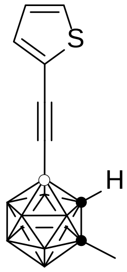

5a. ^{1}H NMR (400 MHz, $CDCl_3$): δ 7.28 (d, *J* = 4.8 Hz, 2H), 6.98 (t, *J* = 4.4 Hz, 1H) (Ar), 3.87 (s, 1H) (cage *H*), 2.09 (s, 3H) (C*H*$_3$). ^{13}C{^{1}H} NMR (100 MHz, $CDCl_3$): δ 133.1, 127.6, 127.0, 123.1 (Ar), 70.4, 62.8 (cage *C*), 26.0 (*C*H$_3$) (alkynyl carbons were not observed). ^{11}B{^{1}H} NMR (128 MHz, $CDCl_3$): δ -0.7 (1B), -4.6 (1B), -7.2 (1B), -8.2 (1B), -10.1 (6B). HRMS: *m*/*z* calcd for $C_9H_{16}B_{10}S$ $[M]^+$: 264.1975. Found: 264.1971.

Homocoupling Reaction of 4a. Compound **4a** (36.4 mg, 0.20 mmol), CuCl (1.0 mg, 0.01 mmol) and TMEDA (1.2 mg, 0.01 mmol) were mixed in acetone (5 mL). The resulting mixture was heated at 100 °C for 24 h in a closed flask. After hydrolysis with water (10 mL) and extraction with diethyl ether (10 mL x 3), the ether solutions were combined and concentrated to dryness in vacuo. The residue was subjected to flash column chromatography to give the product **6a** as colorless crystals (30.4 mg, 84%).

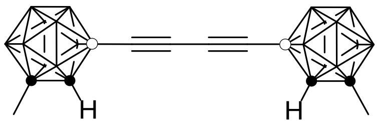

6a. ^{1}H NMR (400 MHz, $CDCl_3$): δ 3.77 (s, 2H) (cage *H*), 2.04 (s, 6H) (C*H*$_3$). ^{13}C{^{1}H} NMR (100 MHz, $CDCl_3$): δ 70.6, 62.8 (cage *C*), 26.0 (*C*H$_3$) (alkynyl carbons were not observed). ^{11}B{^{1}H} NMR (128 MHz, $CDCl_3$): δ -0.4 (1B), -4.4 (1B), -7.1 (1B), -7.9 (1B), -9.3 (2B), -10.0 (2B), -11.1 (2B). HRMS: *m*/*z* calcd for $C_{10}H_{26}B_{20}$ $[M]^+$: 362.4041. Found: 362.4041.

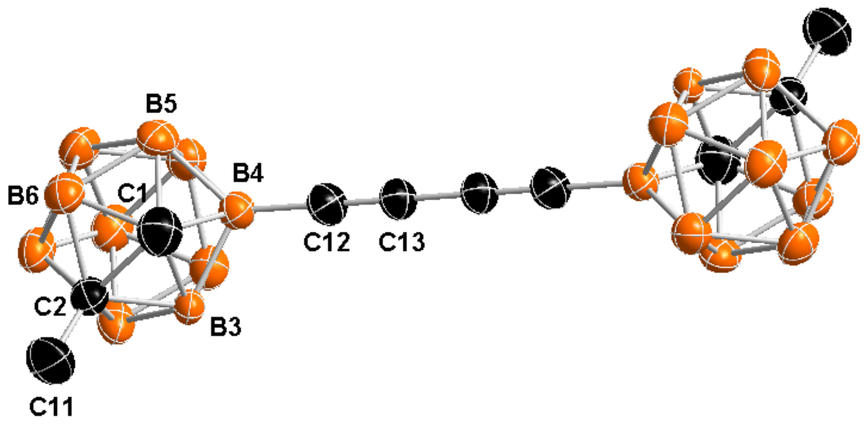

Figure S5. Molecular Structure of **6a**

Reaction of 4a with PhN_3. Compound **4a** (36.4 mg, 0.20 mmol), PhN_3 (47.6 mg, 0.40 mmol) and CuI (7.6 mg, 0.04 mmol) were mixed in DMSO (5 mL). The resulting mixture was stirred at room temperature for 36 h. After hydrolysis with water (10 mL) and extraction

with diethyl ether (10 mL x 3), the ether solutions were combined and dried with anhydrous Na_2SO_4. Removal of solvent gave **7a** as brown oil. (57.2 mg, 95%).

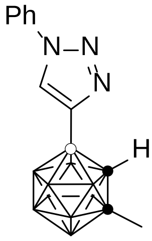

7a. ^{1}H NMR (400 MHz, $CDCl_3$): δ 8.03 (s, 1H), 7.73 (d, *J* = 7.6 Hz, 2H), 7.53 (t, *J* = 7.6 Hz, 2H), 7.44 (t, *J* = 7.2 Hz, 1H) (Ar), 4.49 (s, 1H) (cage *H*), 2.13 (s, 3H) (CH_3). ^{13}C{^{1}H} NMR (100 MHz, $CDCl_3$): δ 136.9, 129.9, 129.0, 120.9 (Ar), 71.1, 62.4 (cage *C*), 26.0 (*C*H_3). ^{11}B{^{1}H} NMR (128 MHz, $CDCl_3$): δ -0.3 (1B), -4.6 (2B), -7.3 (2B), -9.3 (5B). HRMS: *m*/*z* calcd for $C_{11}H_{19}B_{10}N_3$ $[M+H]^+$: 302.2660. Found: 302.2661.

X-ray Structure Determination. X-ray data of **4a** and **6a** were collected at 293 K on a Bruker SMART 1000 CCD diffractometer using Mo-Kα radiation. An empirical absorption correction was applied using the SADABS program.[4] All structures were solved by direct methods and subsequent Fourier difference techniques and refined anisotropically for all non-hydrogen atoms by full-matrix least squares calculations on F^2 using the SHELXTL program package.[5] All hydrogen atoms were geometrically fixed using the riding model. Crystal data and details of data collection and structure refinements are given in Table S1. Details of the crystal structures were deposited in the Cambridge Crystallographic Data Centre with CCDC 1455897-1455898 for **4a** and **6a**.

Table S1. Crystal Data and Summary of Data Collection and Refinement for **4a** and **6a**

compd	**4a**	**6a**
formula	$C_5H_{14}B_{10}$	$C_{10}H_{26}B_{20}$
cryst size (mm)	0.50x0.40x0.30	0.50x0.40x0.30
fw	182.26	362.51
cryst syst	monoclinic	monoclinic
space group	$P2_1/n$	$P2_1/n$
a, Å	12.173(1)	6.818(1)
b, Å	7.921(1)	13.857(1)
c, Å	12.723(1)	11.619(1)
β, deg	112.68(1)	91.19(1)
V, Å^3	1131.89(15)	1097.54(19)
Z	4	2
D_{calcd}, Mg/m^3	1.070	1.097
radiation (λ), Å	0.71073	0.71073
2θ range, deg	3.9 to 50.5	4.6 to 50.5
μ, mm^{-1}	0.047	0.048
F(000)	376	372
no. of obsd reflns	2050	1973
no. of params refnd	136	136
goodness of fit	1.679	1.050
R1	0.119	0.084
wR2	0.398	0.240

References

1 R. A. Kasar, G. M. Knudsen, S. B. Kahl, *Inorg. Chem.*, 1999, **38**, 2936-2940.

2 R. Frei, J. Waser, *J. Am. Chem. Soc.*, 2013, **135**, 9620-9623.

3 C. Tang, Z. Xie, *Angew. Chem. Int. Ed.*, 2015, **54**, 7662-7665.

4 G. M. Sheldrick, SADABS: Program for Empirical Absorption Correction of Area Detector Data. University of Göttingen: Germany, 1996.

5 G. M. Sheldrick, SHELXTL 5.10 for Windows NT: Structure Determination Software Programs. Bruker Analytical X-ray Systems, Inc., Madison, Wisconsin, USA, 1997.

qyj-H-pMe-CD2Cl2

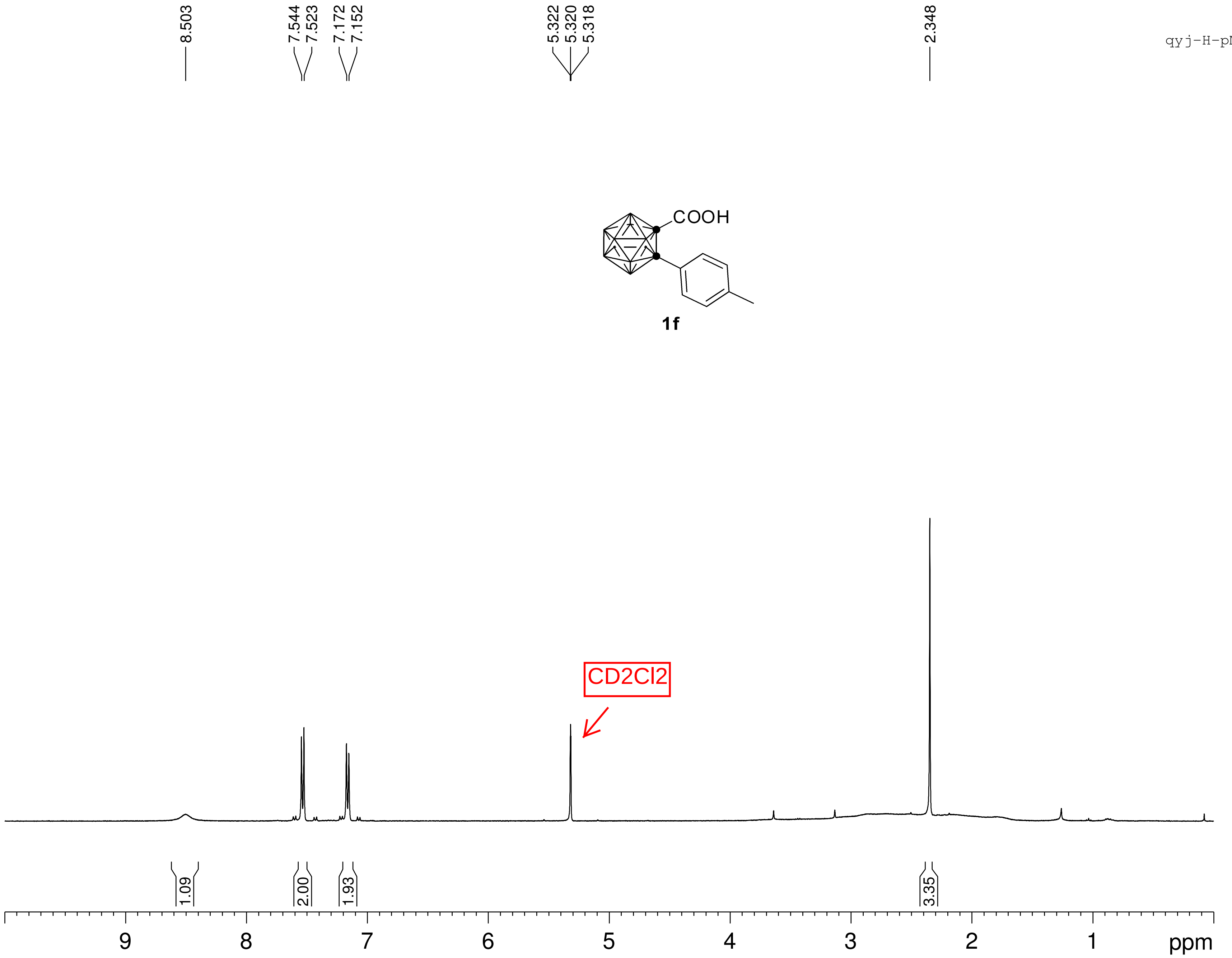

Current Data Parameters
NAME qyj-H-pMe-CD2Cl2
EXPNO 1
PROCNO 1

F2 - Acquisition Parameters
Date_ 20150624
Time 12.19 h
INSTRUM spect
PROBHD Z108618_0257 (
PULPROG zg30
TD 65536
SOLVENT CD2Cl2
NS 8
DS 2
SWH 8012.820 Hz
FIDRES 0.122266 Hz
AQ 4.0894465 sec
RG 144
DW 62.400 usec
DE 6.50 usec
TE 295.2 K
D1 1.00000000 sec
TD0 1
SFO1 400.2324714 MHz
NUC1 1H
P1 12.80 usec
PLW1 13.56000042 W

F2 - Processing parameters
SI 65536
SF 400.2300159 MHz
WDW EM
SSB 0
LB 0.30 Hz
GB 0
PC 1.00

qyj-C-pMe-CD2Cl2

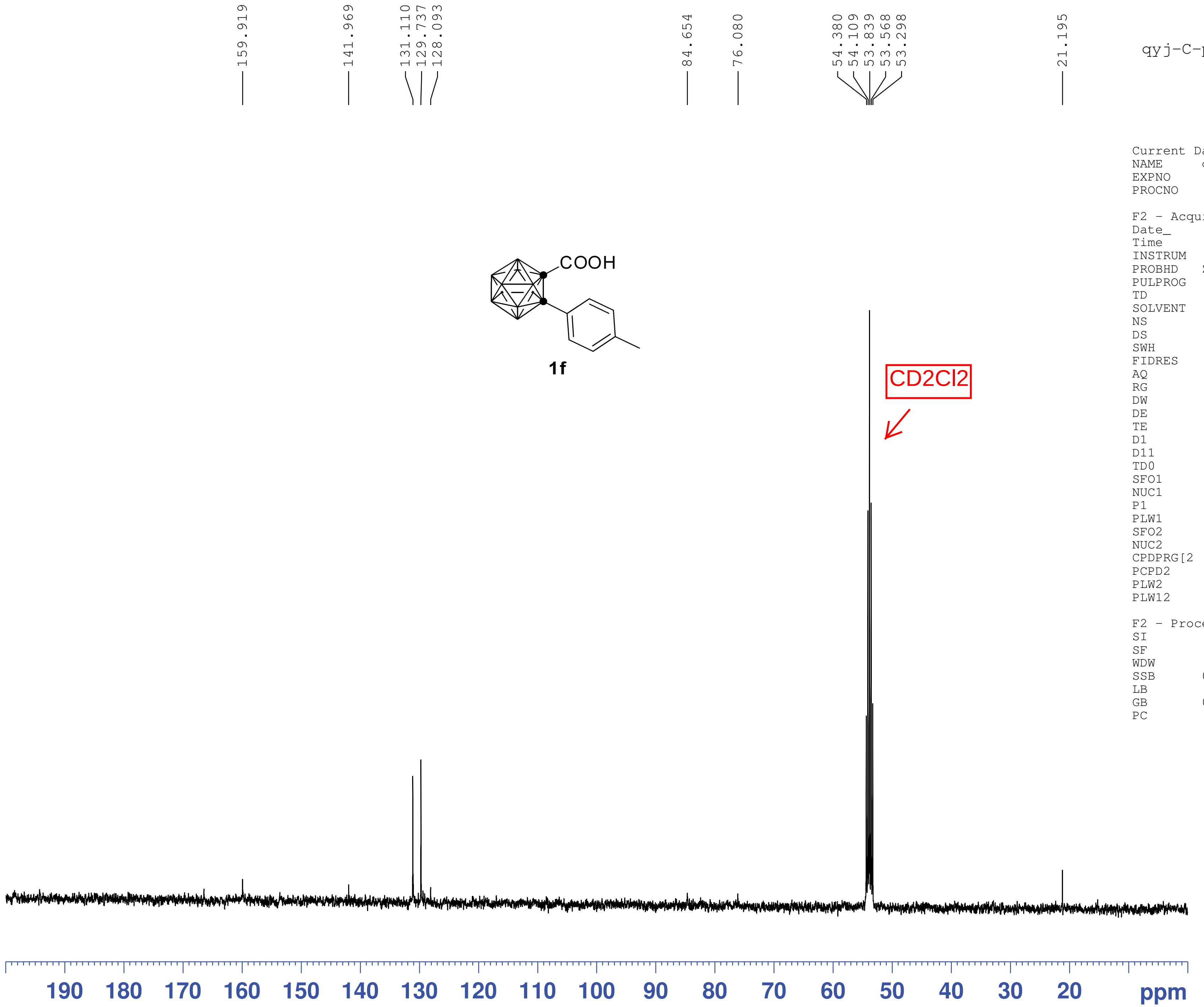

Current Data Parameters
NAME qyj-C-pMe-CD2Cl2
EXPNO 1
PROCNO 1

F2 - Acquisition Parameters
Date_ 20150625
Time 8.57 h
INSTRUM spect
PROBHD Z824601_0021 (
PULPROG zgdc
TD 131072
SOLVENT CD2Cl2
NS 1156
DS 0
SWH 25252.525 Hz
FIDRES 0.192661 Hz
AQ 2.5952256 sec
RG 203
DW 19.800 usec
DE 6.50 usec
TE 295.3 K
D1 1.00000000 sec
D11 0.03000000 sec
TD0 1
SFO1 100.6227690 MHz
NUC1 13C
P1 9.50 usec
PLW1 41.25000000 W
SFO2 400.1320007 MHz
NUC2 1H
CPDPRG[2 waltz16
PCPD2 90.00 usec
PLW2 8.31000042 W
PLW12 0.23083000 W

F2 - Processing parameters
SI 131072
SF 100.6127265 MHz
WDW EM
SSB 0
LB 3.00 Hz
GB 0
PC 1.40

qyj-B-pMe-CDCl3

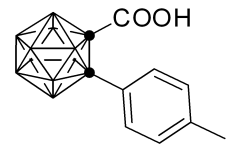

1f

Current Data Parameters
NAME qyj-B-pMe-CDCl3
EXPNO 1
PROCNO 1

F2 - Acquisition Parameters
Date_ 20150624
Time 12.56 h
INSTRUM spect
PROBHD Z108618_0257 (
PULPROG zgpg30
TD 65536
SOLVENT CD2Cl2
NS 64
DS 4
SWH 29761.904 Hz
FIDRES 0.454131 Hz
AQ 1.1010048 sec
RG 575
DW 16.800 usec
DE 6.50 usec
TE 295.6 K
D1 2.00000000 sec
D11 0.03000000 sec
TD0 1
SFO1 128.4096890 MHz
NUC1 11B
P1 7.50 usec
PLW1 55.09999847 W
SFO2 400.2316008 MHz
NUC2 1H
CPDPRG[2 waltz16
PCPD2 90.00 usec
PLW2 13.56000042 W
PLW12 0.27428001 W
PLW13 0.13796000 W

F2 - Processing parameters
SI 32768
SF 128.4095347 MHz
WDW EM
SSB 0
LB 1.00 Hz
GB 0
PC 1.40

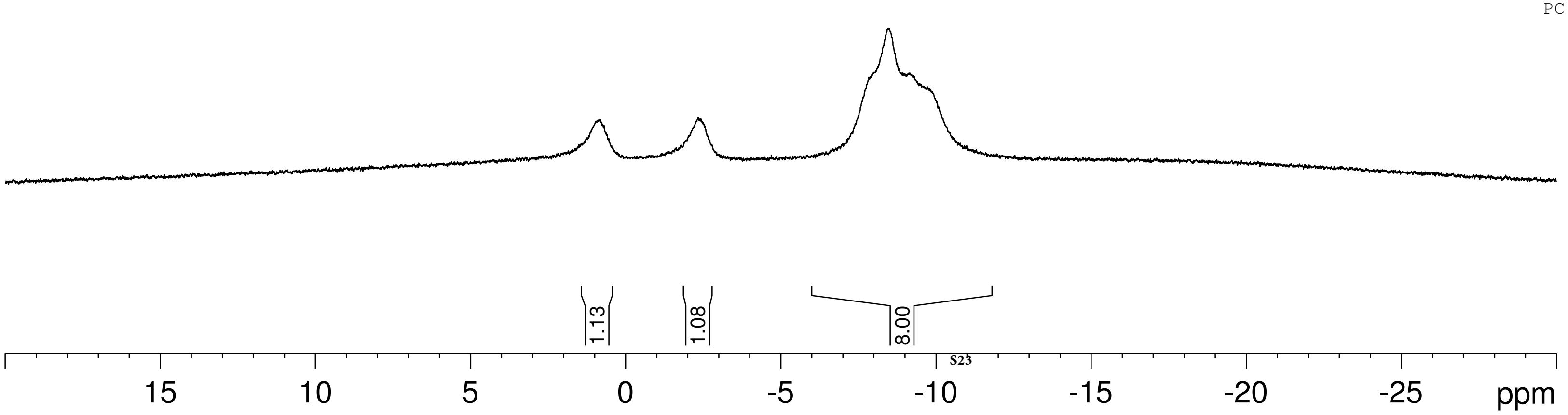

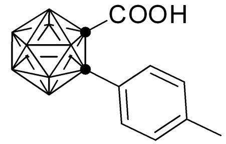

1f

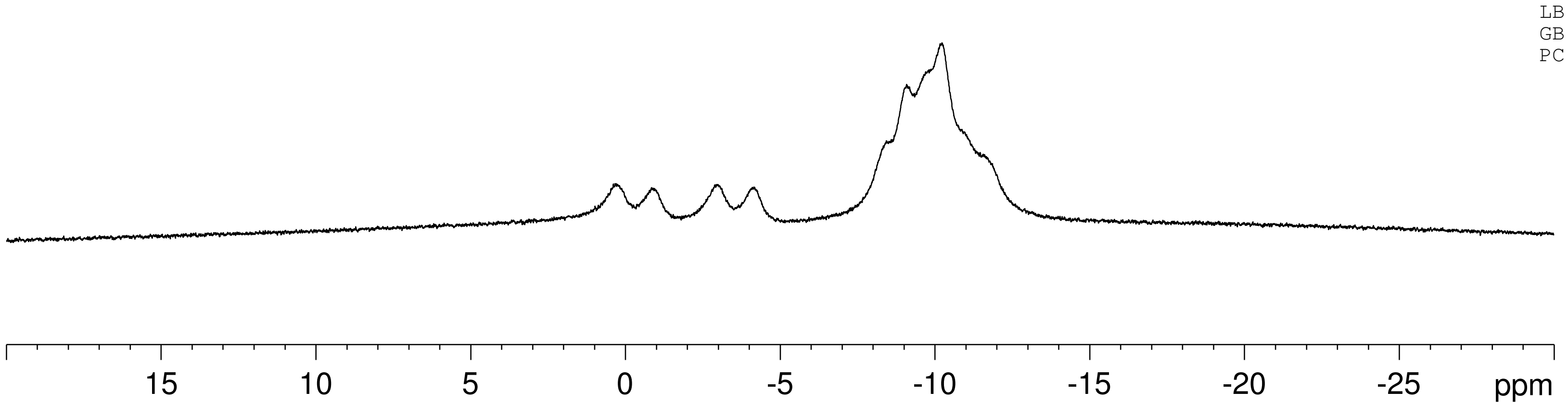

qyj-B-pMe-CDCl3(C)

```
Current Data Parameters
NAME      qyj-B-pMe-CDCl3(C)
EXPNO                  1
PROCNO                 1

F2 - Acquisition Parameters
Date_             20150624
Time                 13.00 h
INSTRUM              spect
PROBHD    Z108618_0257 (
PULPROG                 zg
TD                   65536
SOLVENT             CD2Cl2
NS                      40
DS                       4
SWH              25510.203 Hz
FIDRES            0.389255 Hz
AQ               1.2845056 sec
RG                     322
DW                  19.600 usec
DE                    6.50 usec
TE                   295.4 K
D1              1.00000000 sec
TD0                      1
SFO1           128.4096891 MHz
NUC1                   11B
P1                    7.50 usec
PLW1           55.09999847 W

F2 - Processing parameters
SI                   32768
SF             128.4096891 MHz
WDW                     EM
SSB      0
LB                    1.00 Hz
GB       0
PC                    1.40
```

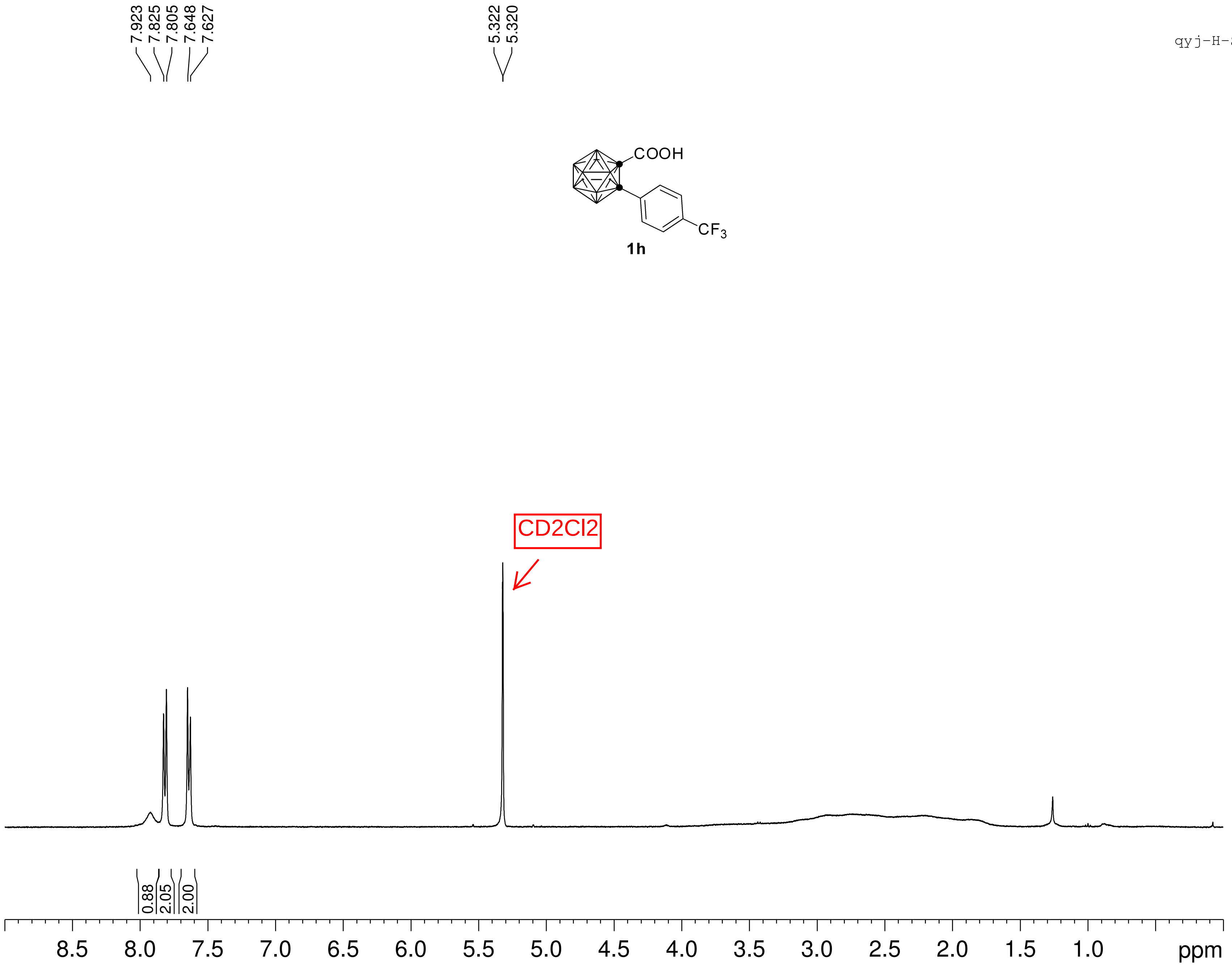

qyj-H-SM-pCF3-CD2Cl2

Current Data Parameters

NAME	qyj-H-SM-pCF3-CD2Cl2
EXPNO	1
PROCNO	1

F2 - Acquisition Parameters

Date_	20150625
Time	17.37 h
INSTRUM	spect
PROBHD	Z108618_0257 (
PULPROG	zg30
TD	65536
SOLVENT	CD2Cl2
NS	8
DS	2
SWH	8012.820 Hz
FIDRES	0.122266 Hz
AQ	4.0894465 sec
RG	144
DW	62.400 usec
DE	6.50 usec
TE	295.4 K
D1	1.00000000 sec
TD0	1
SFO1	400.2324714 MHz
NUC1	1H
P1	12.80 usec
PLW1	13.56000042 W

F2 - Processing parameters

SI	65536
SF	400.2300155 MHz
WDW	EM
SSB	0
LB	0.30 Hz
GB	0
PC	1.00

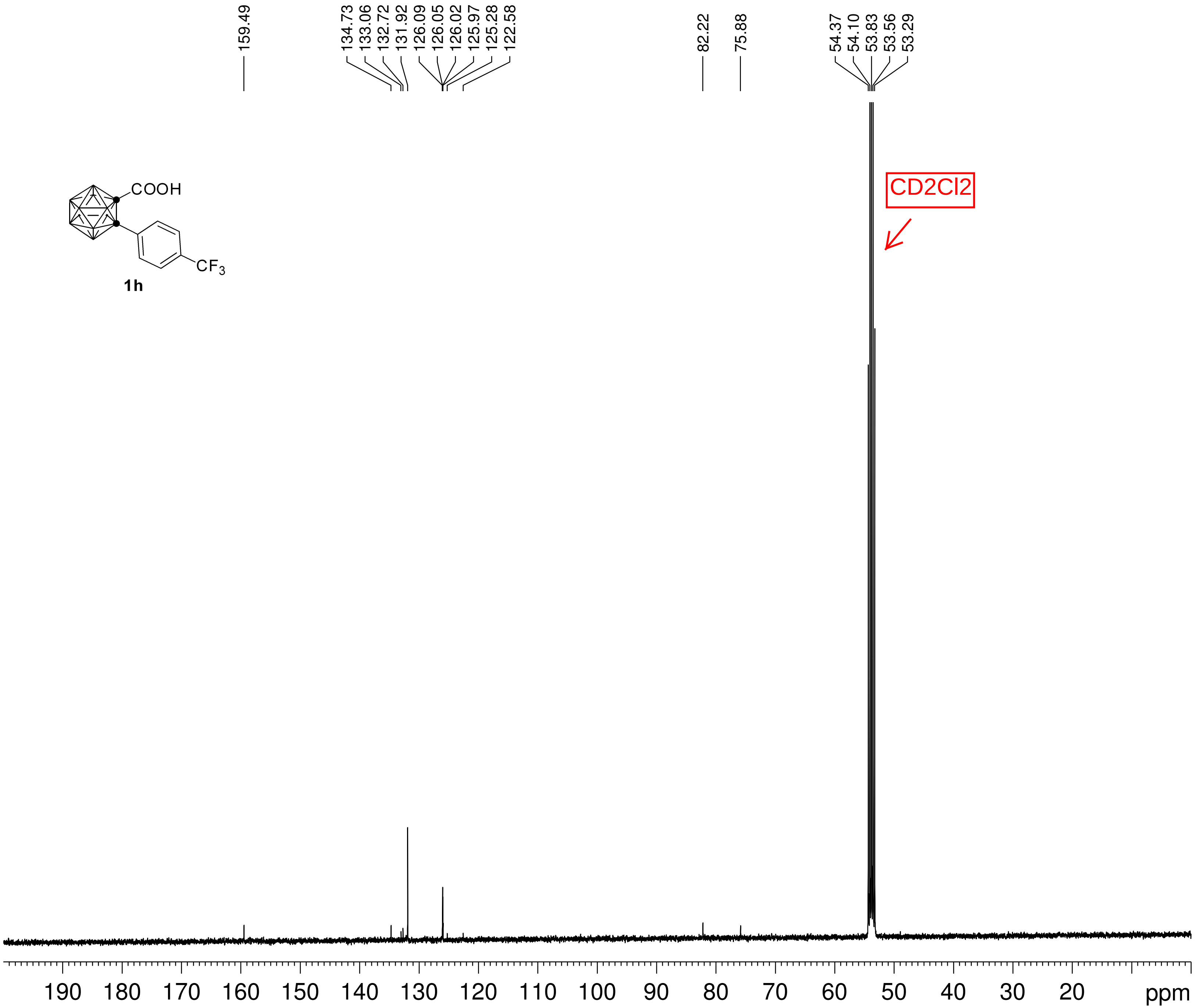

qyj-C-SM-pCF3-CD2C

Current Data Parameters
NAME qyj-C-SM-pCF3-CD2Cl2
EXPNO 1
PROCNO 1

F2 - Acquisition Parameters
Date_ 20150628
Time 13.39 h
INSTRUM spect
PROBHD Z108618_0257 (
PULPROG zgpg30
TD 65536
SOLVENT CD2Cl2
NS 5143
DS 4
SWH 29761.904 Hz
FIDRES 0.454131 Hz
AQ 1.1010048 sec
RG 128
DW 16.800 usec
DE 6.50 usec
TE 295.6 K
D1 2.00000000 sec
D11 0.03000000 sec
TD0 1
SFO1 100.6479773 MHz
NUC1 13C
P1 9.50 usec
PLW1 55.34000015 W
SFO2 400.2316009 MHz
NUC2 1H
CPDPRG[2 waltz16
PCPD2 90.00 usec
PLW2 13.56000042 W
PLW12 0.27428001 W
PLW13 0.13796000 W

F2 - Processing parameters
SI 32768
SF 100.6378733 MHz
WDW EM
SSB 0
LB 1.00 Hz
GB 0
PC 1.40

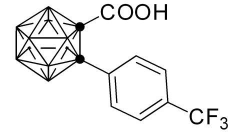

1h

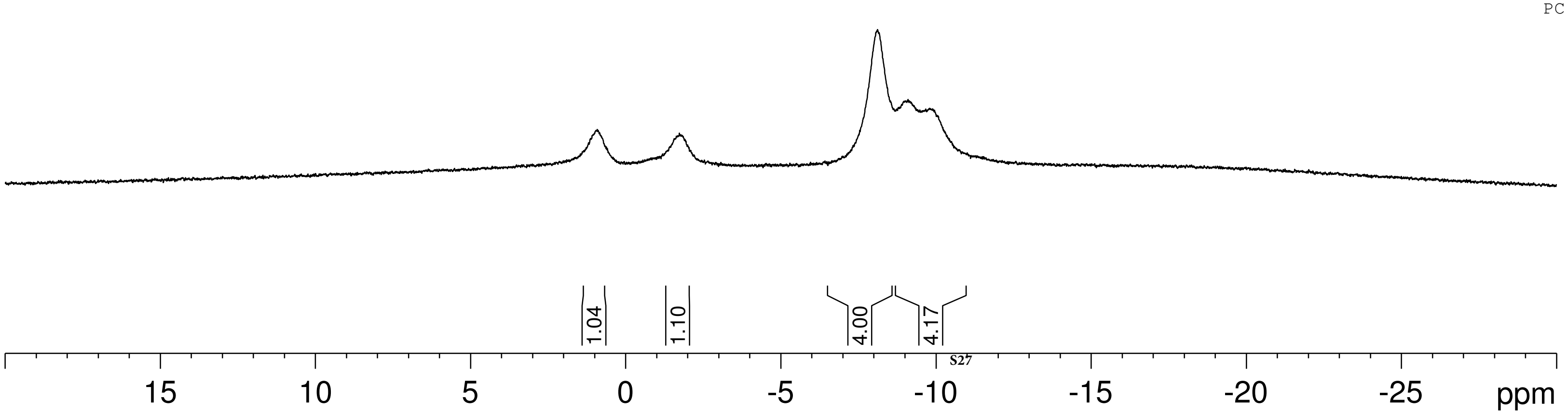

qyj-B-SM-pCF3-CD2Cl2

Current Data Parameters
NAME qyj-B-SM-pCF3-CD2Cl2
EXPNO 1
PROCNO 1

F2 - Acquisition Parameters
Date_ 20150625
Time 17.27 h
INSTRUM spect
PROBHD Z108618_0257 (
PULPROG zgpg30
TD 65536
SOLVENT DMSO
NS 78
DS 4
SWH 29761.904 Hz
FIDRES 0.454131 Hz
AQ 1.1010048 sec
RG 456
DW 16.800 usec
DE 6.50 usec
TE 295.8 K
D1 2.00000000 sec
D11 0.03000000 sec
TD0 1
SFO1 128.4096890 MHz
NUC1 11B
P1 7.50 usec
PLW1 55.09999847 W
SFO2 400.2316008 MHz
NUC2 1H
CPDPRG[2 waltz16
PCPD2 90.00 usec
PLW2 13.56000042 W
PLW12 0.27428001 W
PLW13 0.13796000 W

F2 - Processing parameters
SI 32768
SF 128.4095347 MHz
WDW EM
SSB 0
LB 1.00 Hz
GB 0
PC 1.40

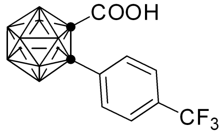

1h

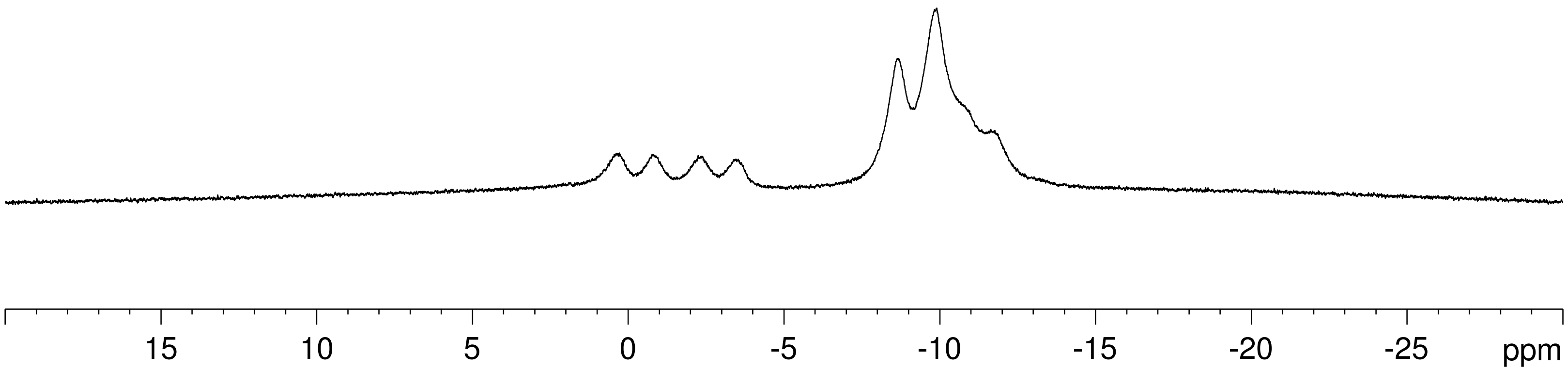

qyj-B-SM-pCF3-CD2Cl2(C)

Current Data Parameters
NAME qyj-B-SM-pCF3-CD2Cl2(C)
EXPNO 1
PROCNO 1

F2 - Acquisition Parameters
Date_ 20150625
Time 17.32 h
INSTRUM spect
PROBHD Z108618_0257 (
PULPROG zg
TD 65536
SOLVENT DMSO
NS 44
DS 4
SWH 25510.203 Hz
FIDRES 0.389255 Hz
AQ 1.2845056 sec
RG 456
DW 19.600 usec
DE 6.50 usec
TE 295.5 K
D1 1.00000000 sec
TD0 1
SFO1 128.4096891 MHz
NUC1 11B
P1 7.50 usec
PLW1 55.09999847 W

F2 - Processing parameters
SI 32768
SF 128.4096891 MHz
WDW EM
SSB 0
LB 1.00 Hz
GB 0
PC 1.40

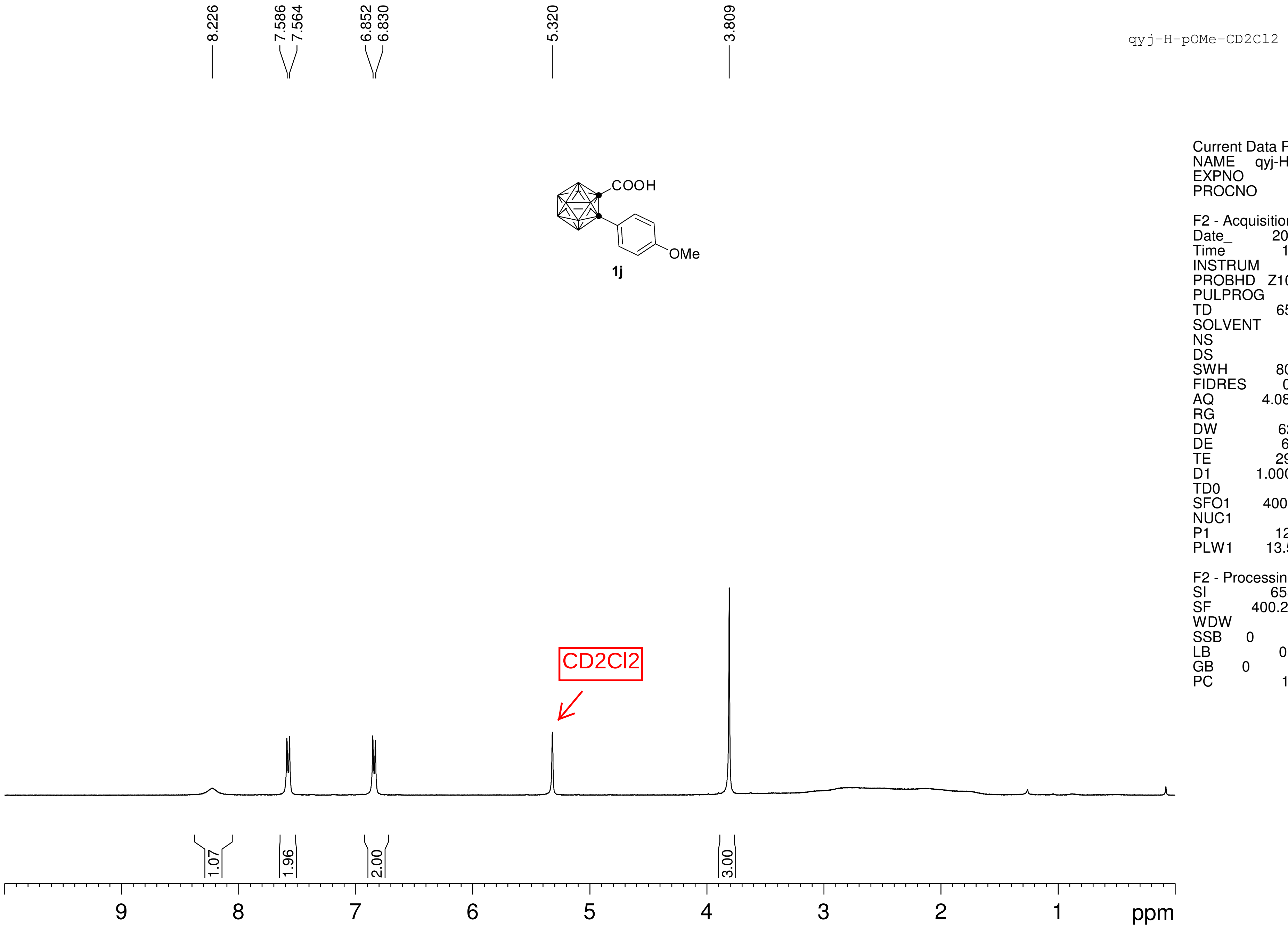

Current Data Parameters
NAME qyj-H-pOMe-CD2Cl2
EXPNO 1
PROCNO 1

F2 - Acquisition Parameters
Date_ 20150624
Time 12.26 h
INSTRUM spect
PROBHD Z108618_0257 (
PULPROG zg30
TD 65536
SOLVENT CD2Cl2
NS 8
DS 2
SWH 8012.820 Hz
FIDRES 0.122266 Hz
AQ 4.0894465 sec
RG 144
DW 62.400 usec
DE 6.50 usec
TE 295.3 K
D1 1.00000000 sec
TD0 1
SFO1 400.2324714 MHz
NUC1 1H
P1 12.80 usec
PLW1 13.56000042 W

F2 - Processing parameters
SI 65536
SF 400.2300162 MHz
WDW EM
SSB 0
LB 0.30 Hz
GB 0
PC 1.00

qyj-C-pOMe-CDCl3

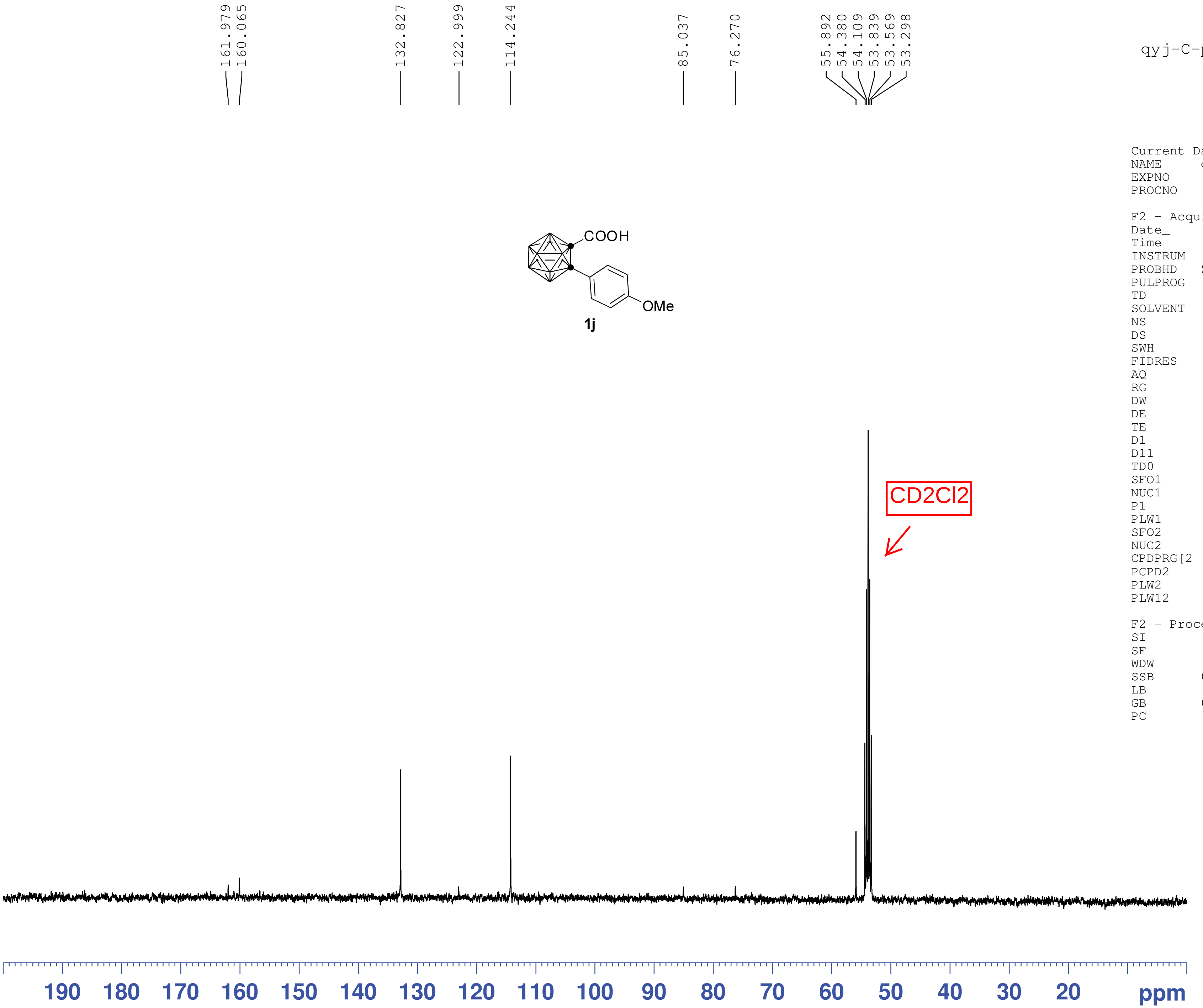

Current Data Parameters
NAME qyj-C-pOMe-CDCl3
EXPNO 1
PROCNO 1

F2 - Acquisition Parameters
Date_ 20150624
Time 19.33 h
INSTRUM spect
PROBHD Z824601_0021 (
PULPROG zgdc
TD 131072
SOLVENT CD2Cl2
NS 1360
DS 0
SWH 25252.525 Hz
FIDRES 0.192661 Hz
AQ 2.5952256 sec
RG 203
DW 19.800 usec
DE 6.50 usec
TE 295.1 K
D1 1.00000000 sec
D11 0.03000000 sec
TD0 1
SFO1 100.6227690 MHz
NUC1 13C
P1 9.50 usec
PLW1 41.25000000 W
SFO2 400.1320007 MHz
NUC2 1H
CPDPRG[2 waltz16
PCPD2 90.00 usec
PLW2 8.31000042 W
PLW12 0.23083000 W

F2 - Processing parameters
SI 131072
SF 100.6127265 MHz
WDW EM
SSB 0
LB 3.00 Hz
GB 0
PC 1.40

qyj-B-pOMe-CDCl3

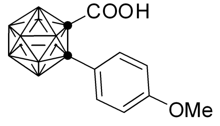

1j

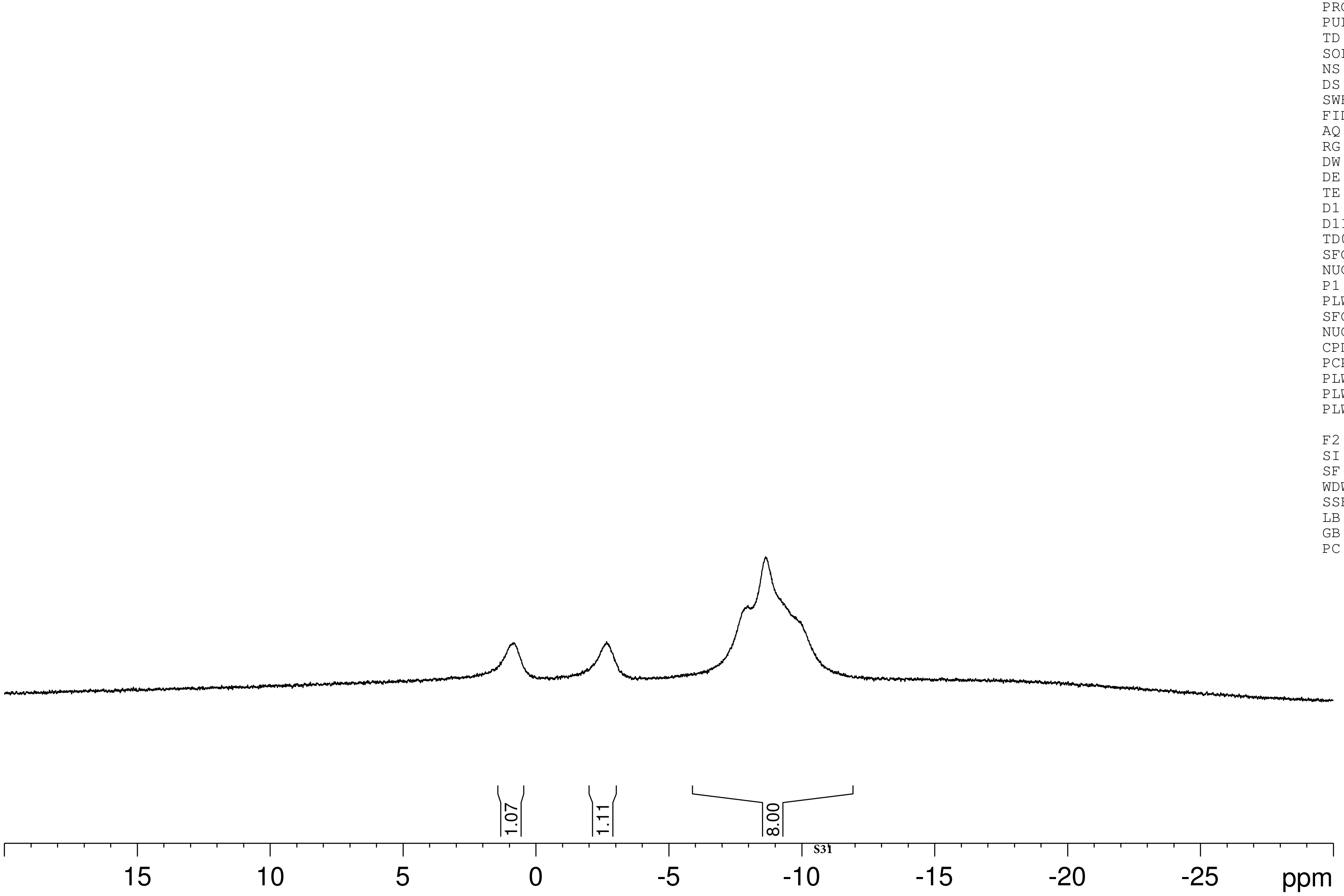


```
Current Data Parameters
NAME      qyj-B-pOMe-CDCl3
EXPNO                    1
PROCNO                   1

F2 - Acquisition Parameters
Date_            20150624
Time                12.50 h
INSTRUM             spect
PROBHD    Z108618_0257 (
PULPROG           zgpg30
TD                 65536
SOLVENT           CD2Cl2
NS                    64
DS                     4
SWH            29761.904 Hz
FIDRES          0.454131 Hz
AQ             1.1010048 sec
RG                   575
DW                16.800 usec
DE                  6.50 usec
TE                 295.6 K
D1            2.00000000 sec
D11           0.03000000 sec
TD0                    1
SFO1         128.4096890 MHz
NUC1                 11B
P1                  7.50 usec
PLW1         55.09999847 W
SFO2         400.2316008 MHz
NUC2                  1H
CPDPRG[2          waltz16
PCPD2              90.00 usec
PLW2         13.56000042 W
PLW12         0.27428001 W
PLW13         0.13796000 W

F2 - Processing parameters
SI                 32768
SF           128.4095347 MHz
WDW                   EM
SSB       0
LB                  1.00 Hz
GB        0
PC                  1.40
```

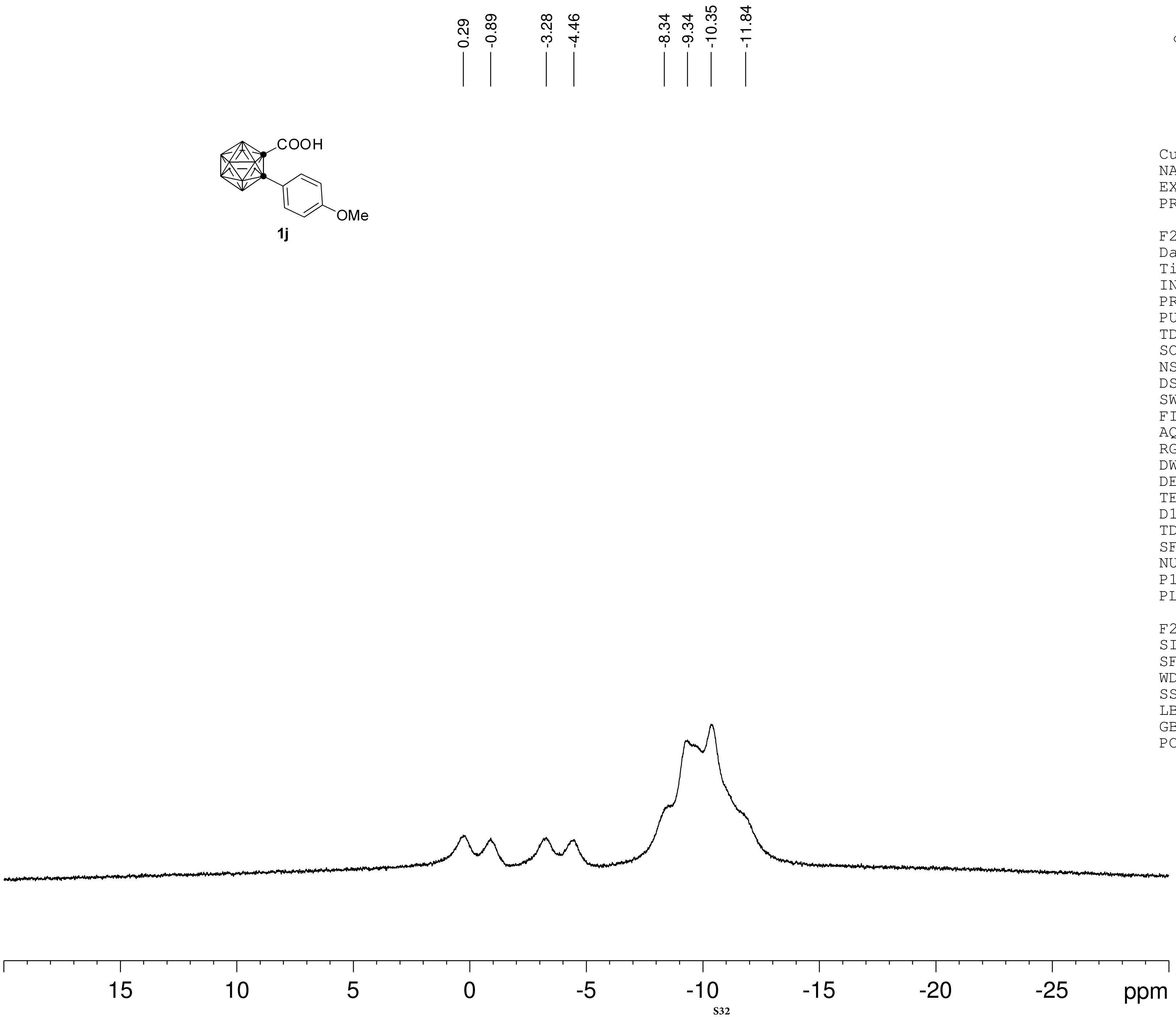

qyj-B-pOMe-CDCl3(C)

Current Data Parameters
NAME qyj-B-pOMe-CDCl3(C)
EXPNO 1
PROCNO 1

F2 - Acquisition Parameters
Date_ 20150624
Time 12.54 h
INSTRUM spect
PROBHD Z108618_0257 (
PULPROG zg
TD 65536
SOLVENT CD2Cl2
NS 32
DS 4
SWH 25510.203 Hz
FIDRES 0.389255 Hz
AQ 1.2845056 sec
RG 322
DW 19.600 usec
DE 6.50 usec
TE 295.4 K
D1 1.00000000 sec
TD0 1
SFO1 128.4096891 MHz
NUC1 11B
P1 7.50 usec
PLW1 55.09999847 W

F2 - Processing parameters
SI 32768
SF 128.4096891 MHz
WDW EM
SSB 0
LB 1.00 Hz
GB 0
PC 1.40

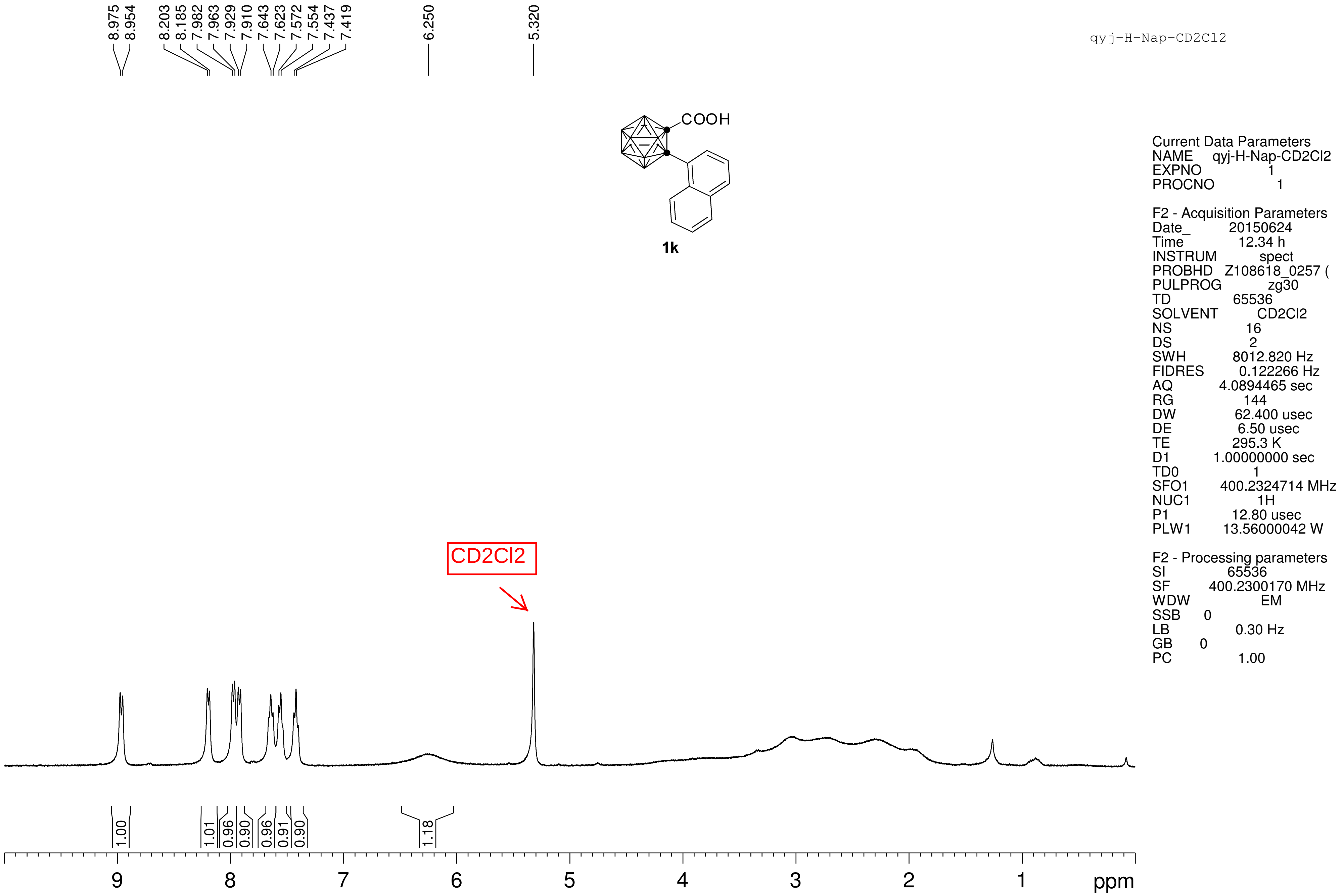
8.975
8.954
8.203
8.185
7.982
7.963
7.929
7.910
7.643
7.623
7.572
7.554
7.437
7.419
6.250
5.320
qyj-H-Nap-CD2Cl2
COOH
1k
Current Data Parameters
NAME qyj-H-Nap-CD2Cl2
EXPNO 1
PROCNO 1
F2 - Acquisition Parameters
Date_ 20150624
Time 12.34 h
INSTRUM spect
PROBHD Z108618_0257 (
PULPROG zg30
TD 65536
SOLVENT CD2Cl2
NS 16
DS 2
SWH 8012.820 Hz
FIDRES 0.122266 Hz
AQ 4.0894465 sec
RG 144
DW 62.400 usec
DE 6.50 usec
TE 295.3 K
D1 1.00000000 sec
TD0 1
SFO1 400.2324714 MHz
NUC1 1H
P1 12.80 usec
PLW1 13.56000042 W
F2 - Processing parameters
SI 65536
SF 400.2300170 MHz
WDW EM
SSB 0
LB 0.30 Hz
GB 0
PC 1.00
CD2Cl2
1.00
1.01
0.96
0.90
0.96
0.91
0.90
1.18
9
8
7
6
5
4
3
2
1
ppm

qyj-C-Nap-CDCl3

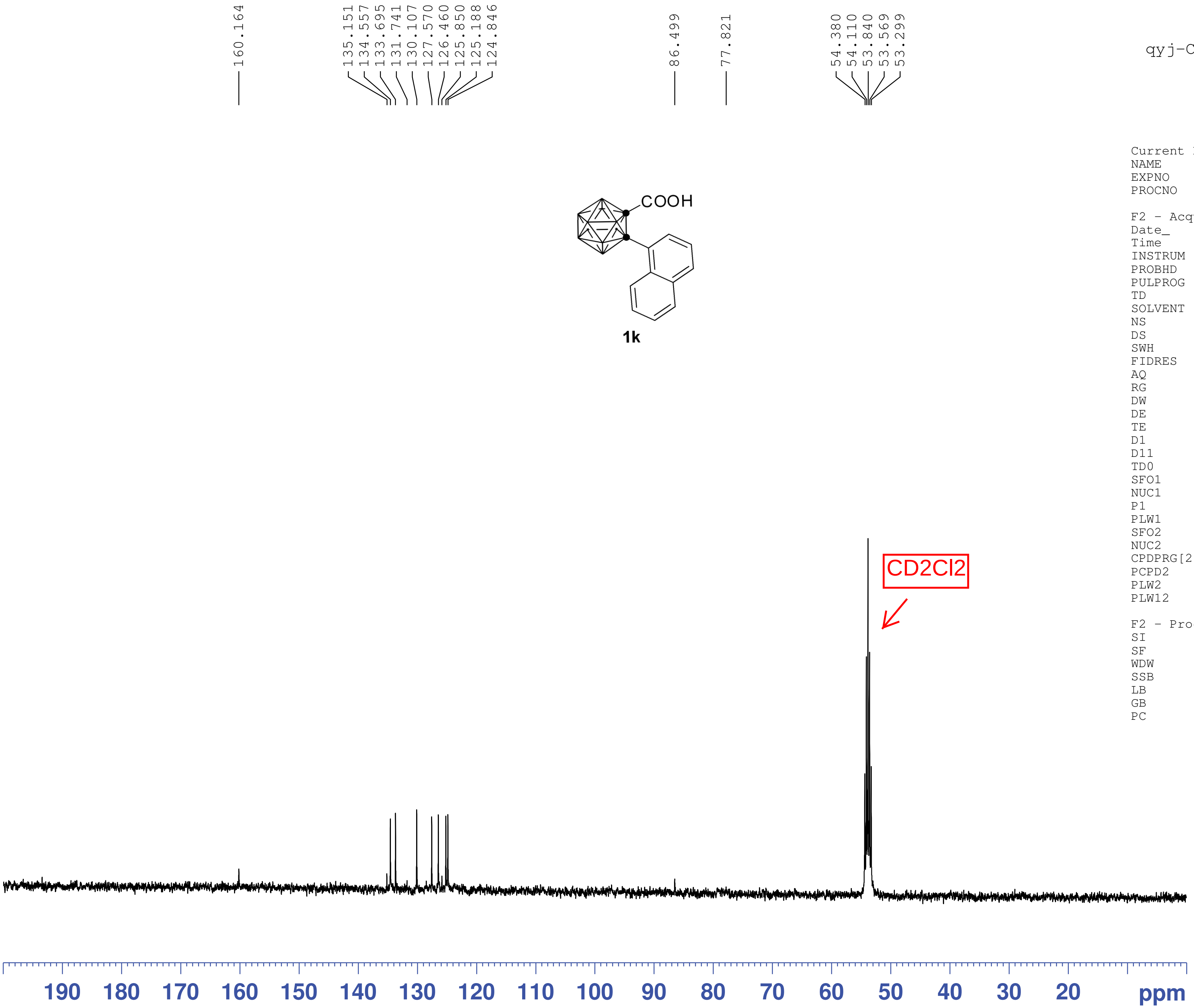

Current Data Parameters
NAME qyj-C-Nap-CDCl3
EXPNO 1
PROCNO 1

F2 - Acquisition Parameters
Date_ 20150625
Time 11.56 h
INSTRUM spect
PROBHD Z824601_0021 (
PULPROG zgdc
TD 131072
SOLVENT CD2Cl2
NS 1348
DS 0
SWH 25252.525 Hz
FIDRES 0.192661 Hz
AQ 2.5952256 sec
RG 203
DW 19.800 usec
DE 6.50 usec
TE 296.8 K
D1 1.00000000 sec
D11 0.03000000 sec
TD0 1
SFO1 100.6227690 MHz
NUC1 13C
P1 9.50 usec
PLW1 41.25000000 W
SFO2 400.1320007 MHz
NUC2 1H
CPDPRG[2 waltz16
PCPD2 90.00 usec
PLW2 8.31000042 W
PLW12 0.23083000 W

F2 - Processing parameters
SI 131072
SF 100.6127254 MHz
WDW EM
SSB 0
LB 3.00 Hz
GB 0
PC 1.40

qyj-B-Nap-CDCl3

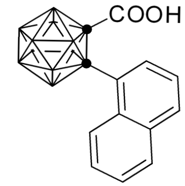

1k

Current Data Parameters
NAME qyj-B-Nap-CDCl3
EXPNO 1
PROCNO 1

F2 - Acquisition Parameters
Date_ 20150624
Time 12.41 h
INSTRUM spect
PROBHD Z108618_0257 (
PULPROG zgpg30
TD 65536
SOLVENT CD2Cl2
NS 100
DS 4
SWH 29761.904 Hz
FIDRES 0.454131 Hz
AQ 1.1010048 sec
RG 1
DW 16.800 usec
DE 6.50 usec
TE 295.6 K
D1 2.00000000 sec
D11 0.03000000 sec
TD0 1
SFO1 128.4096890 MHz
NUC1 11B
P1 7.50 usec
PLW1 55.09999847 W
SFO2 400.2316008 MHz
NUC2 1H
CPDPRG[2 waltz16
PCPD2 90.00 usec
PLW2 13.56000042 W
PLW12 0.27428001 W
PLW13 0.13796000 W

F2 - Processing parameters
SI 32768
SF 128.4095347 MHz
WDW EM
SSB 0
LB 1.00 Hz
GB 0
PC 1.40

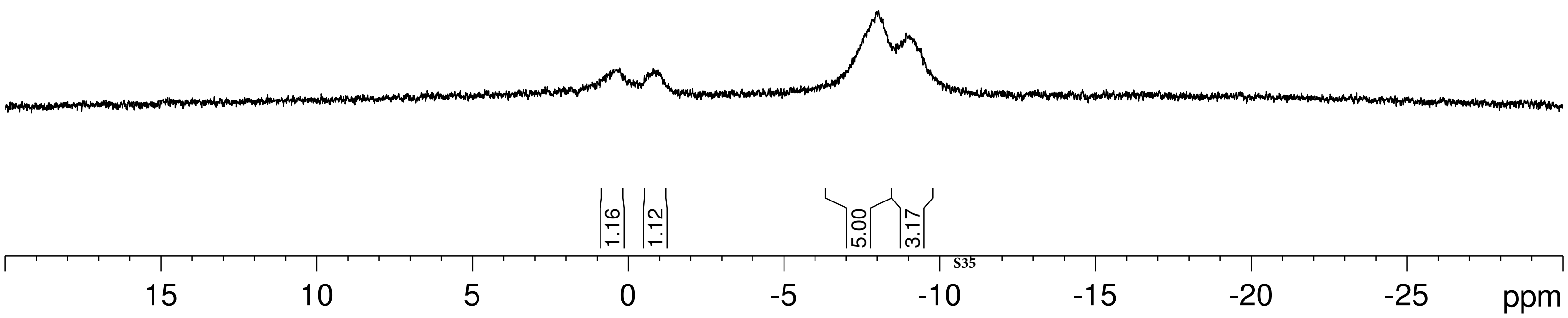

qyj-B-Nap-CDCl3(C)

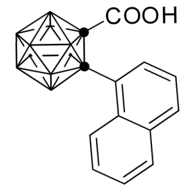

1k

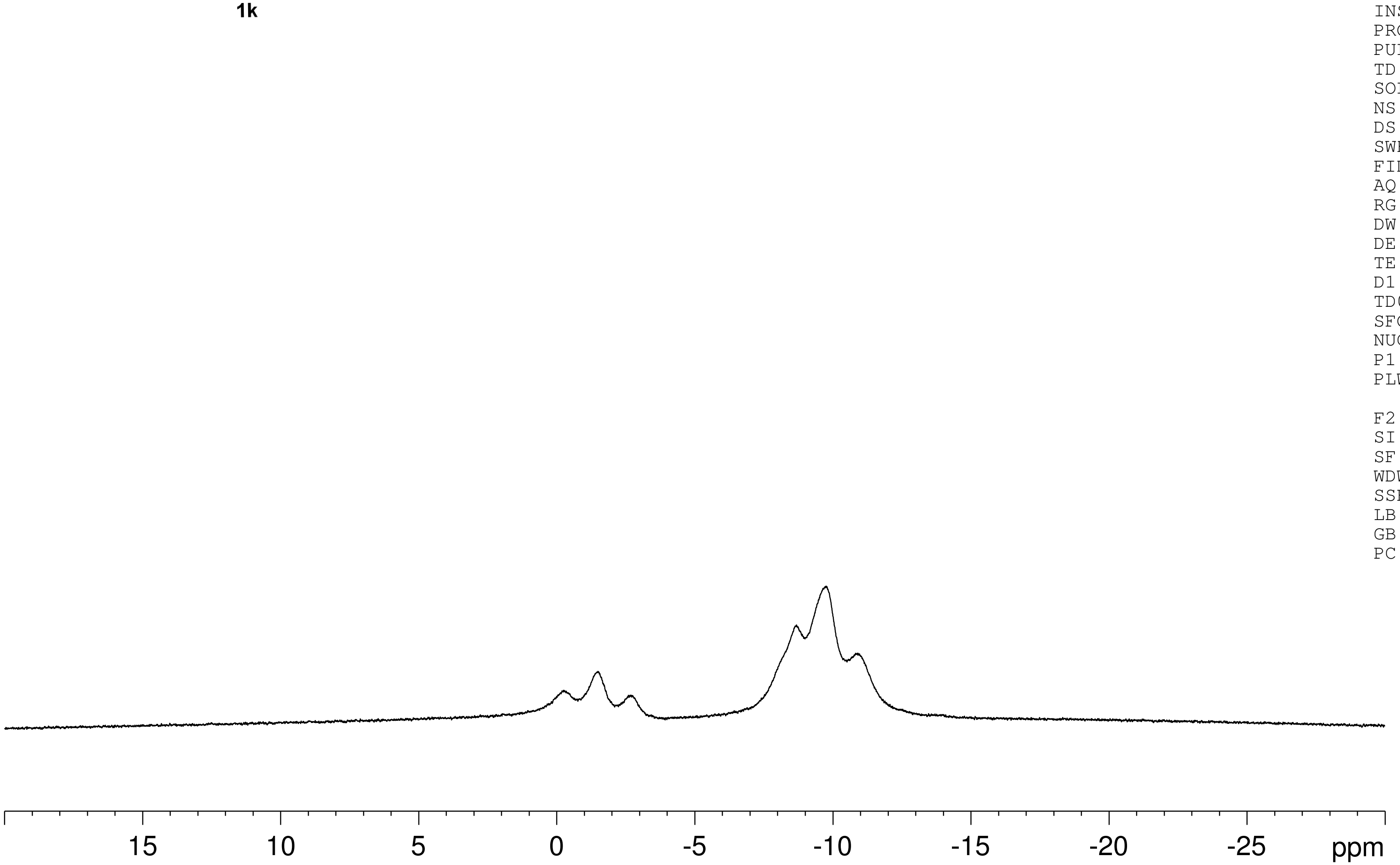


```
Current Data Parameters
NAME       qyj-B-Nap-CDCl3(C)
EXPNO                       1
PROCNO                      1

F2 - Acquisition Parameters
Date_              20150624
Time                  12.48 h
INSTRUM               spect
PROBHD     Z108618_0257 (
PULPROG                  zg
TD                    65536
SOLVENT              CD2Cl2
NS                       37
DS                        4
SWH               25510.203 Hz
FIDRES             0.389255 Hz
AQ                1.2845056 sec
RG                      322
DW                   19.600 usec
DE                     6.50 usec
TE                    295.4 K
D1               1.00000000 sec
TD0                       1
SFO1            128.4096891 MHz
NUC1                    11B
P1                     7.50 usec
PLW1            55.09999847 W

F2 - Processing parameters
SI                    32768
SF              128.4096891 MHz
WDW                      EM
SSB        0
LB                     1.00 Hz
GB         0
PC                     1.40
```

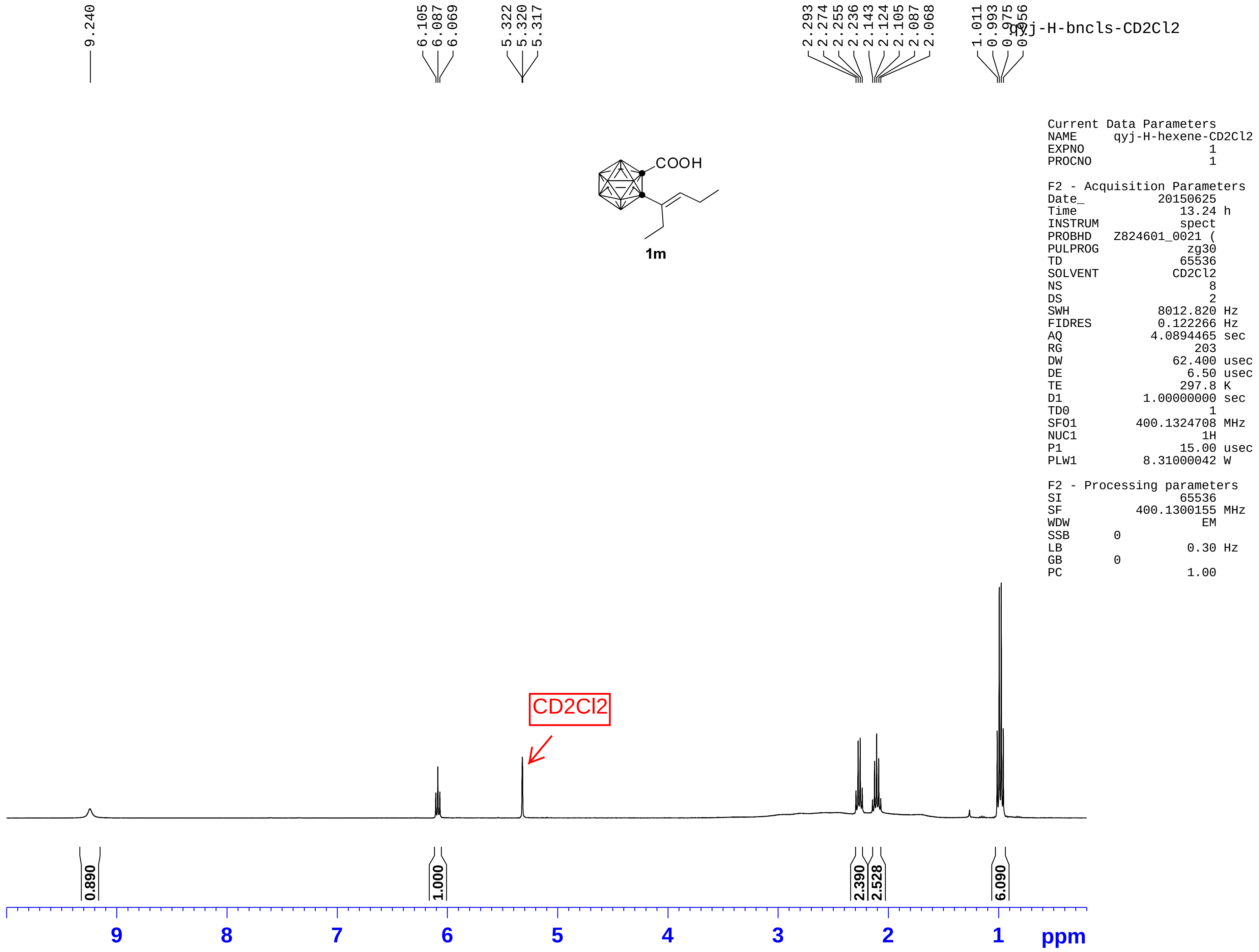
9.240
6.105
6.087
6.069
5.322
5.320
5.317
2.293
2.274
2.255
2.236
2.143
2.124
2.105
2.087
2.068
1.011
0.993
0.975
0.956
qyj-H-bncls-CD2Cl2
COOH
1m
Current Data Parameters
NAME qyj-H-hexene-CD2Cl2
EXPNO 1
PROCNO 1
F2 - Acquisition Parameters
Date_ 20150625
Time 13.24 h
INSTRUM spect
PROBHD Z824601_0021 (
PULPROG zg30
TD 65536
SOLVENT CD2Cl2
NS 8
DS 2
SWH 8012.820 Hz
FIDRES 0.122266 Hz
AQ 4.0894465 sec
RG 203
DW 62.400 usec
DE 6.50 usec
TE 297.8 K
D1 1.00000000 sec
TD0 1
SFO1 400.1324708 MHz
NUC1 1H
P1 15.00 usec
PLW1 8.31000042 W
F2 - Processing parameters
SI 65536
SF 400.1300155 MHz
WDW EM
SSB 0
LB 0.30 Hz
GB 0
PC 1.00
CD2Cl2
0.890
1.000
2.390
2.528
6.090
9
8
7
6
5
4
3
2
1
ppm

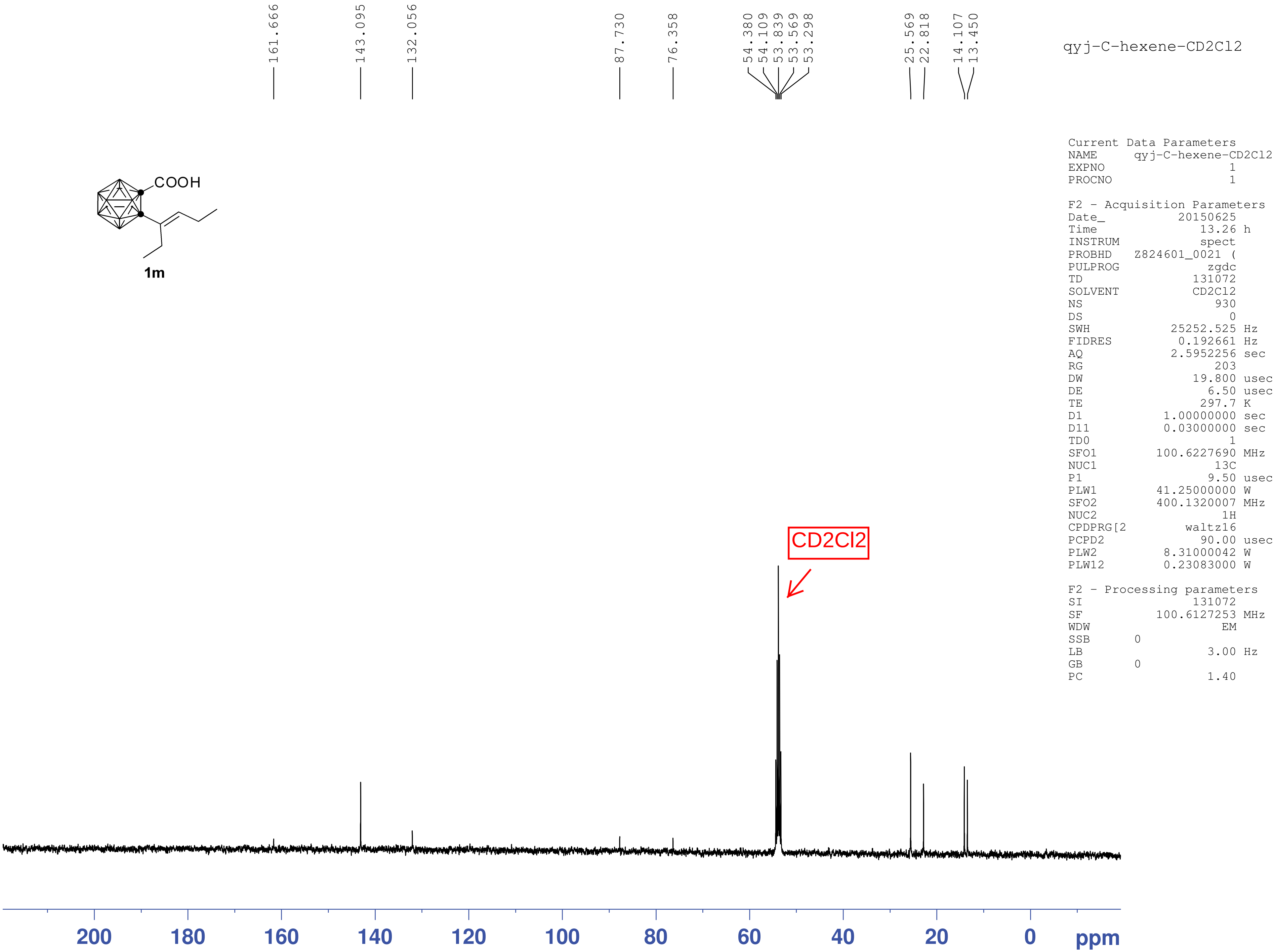

161.666
143.095
132.056
87.730
76.358
54.380
54.109
53.839
53.569
53.298
25.569
22.818
14.107
13.450
qyj-C-hexene-CD2Cl2
COOH
1m
CD2Cl2
Current Data Parameters
NAME qyj-C-hexene-CD2Cl2
EXPNO 1
PROCNO 1
F2 - Acquisition Parameters
Date_ 20150625
Time 13.26 h
INSTRUM spect
PROBHD Z824601_0021 (
PULPROG zgdc
TD 131072
SOLVENT CD2Cl2
NS 930
DS 0
SWH 25252.525 Hz
FIDRES 0.192661 Hz
AQ 2.5952256 sec
RG 203
DW 19.800 usec
DE 6.50 usec
TE 297.7 K
D1 1.00000000 sec
D11 0.03000000 sec
TD0 1
SFO1 100.6227690 MHz
NUC1 13C
P1 9.50 usec
PLW1 41.25000000 W
SFO2 400.1320007 MHz
NUC2 1H
CPDPRG[2 waltz16
PCPD2 90.00 usec
PLW2 8.31000042 W
PLW12 0.23083000 W
F2 - Processing parameters
SI 131072
SF 100.6127253 MHz
WDW EM
SSB 0
LB 3.00 Hz
GB 0
PC 1.40
200
180
160
140
120
100
80
60
40
20
0
ppm

qyj-B-SM-alkene-CD2Cl2

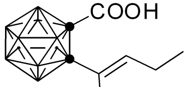

1m

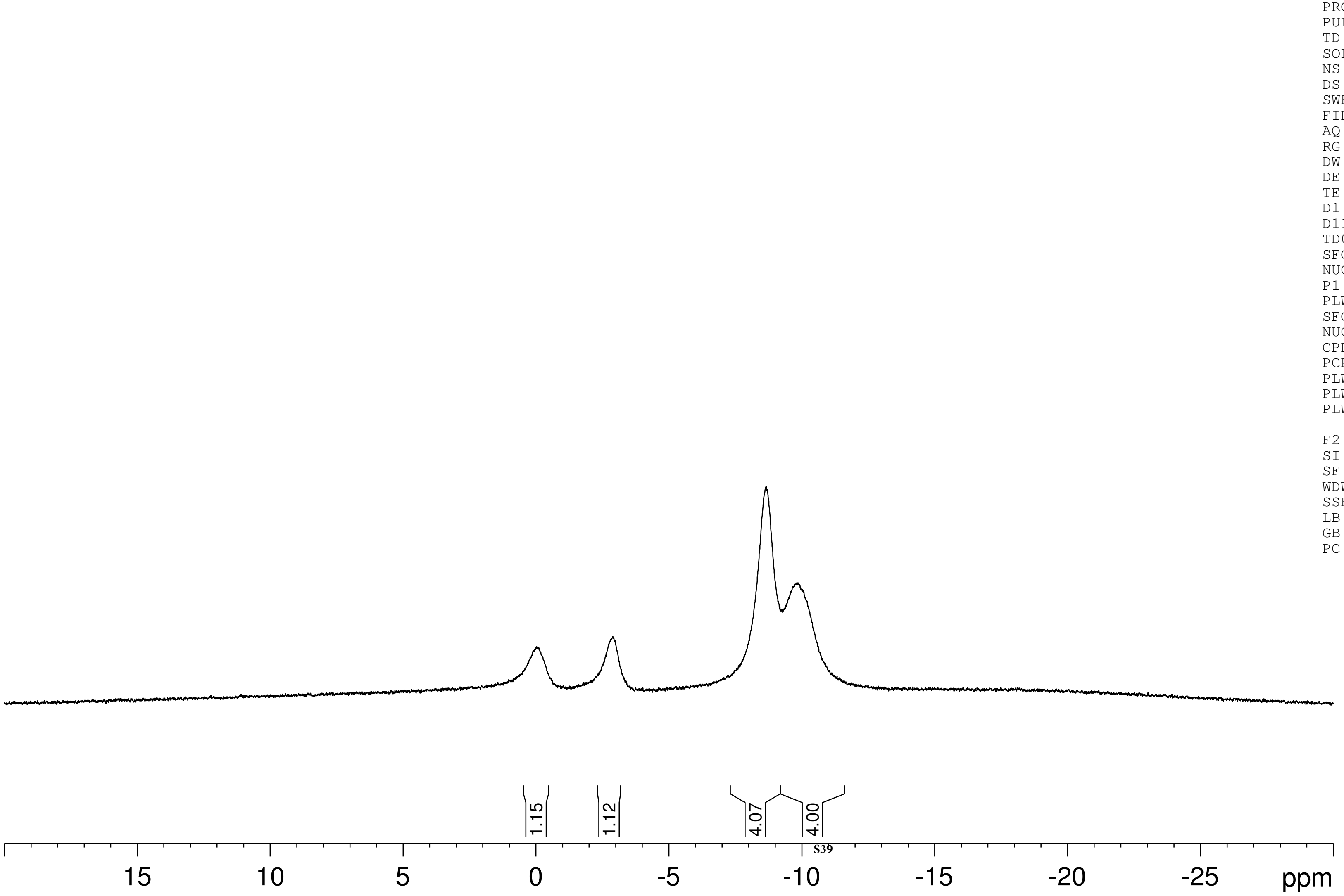


```
Current Data Parameters
NAME     qyj-B-SM-alkene-CD2Cl2
EXPNO                1
PROCNO               1

F2 - Acquisition Parameters
Date_          20150625
Time              17.18 h
INSTRUM           spect
PROBHD   Z108618_0257 (
PULPROG          zgpg30
TD                65536
SOLVENT            DMSO
NS                   40
DS                    4
SWH           29761.904 Hz
FIDRES         0.454131 Hz
AQ            1.1010048 sec
RG                  456
DW               16.800 usec
DE                 6.50 usec
TE                295.6 K
D1           2.00000000 sec
D11          0.03000000 sec
TD0                   1
SFO1        128.4096890 MHz
NUC1                11B
P1                 7.50 usec
PLW1        55.09999847 W
SFO2        400.2316008 MHz
NUC2                 1H
CPDPRG[2        waltz16
PCPD2             90.00 usec
PLW2        13.56000042 W
PLW12        0.27428001 W
PLW13        0.13796000 W

F2 - Processing parameters
SI                32768
SF          128.4095347 MHz
WDW                  EM
SSB      0
LB                 1.00 Hz
GB       0
PC                 1.40
```

qyj-B-SM-alkene-CD2Cl2(C)

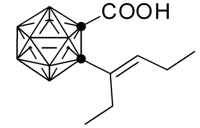

1m

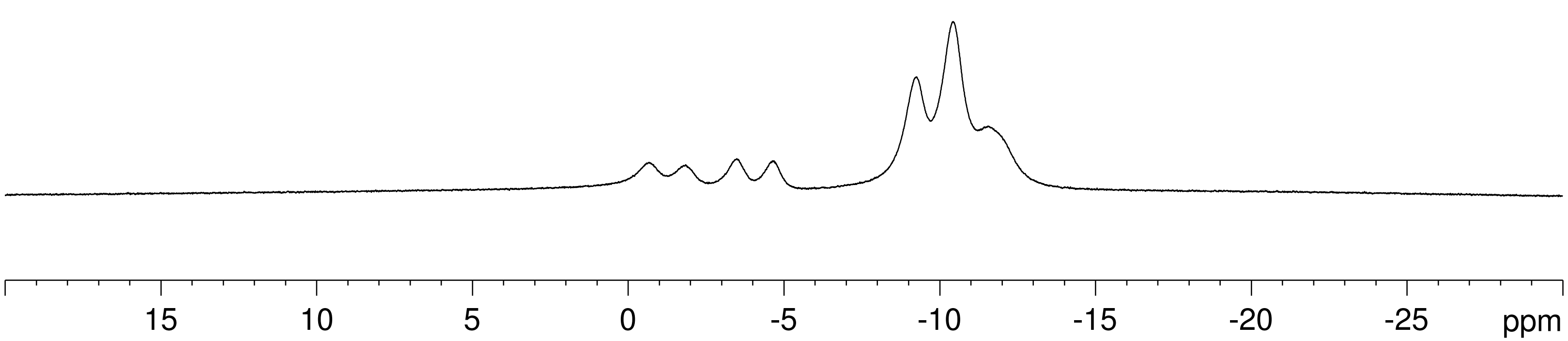


```
Current Data Parameters
NAME     qyj-B-SM-alkene-CD2Cl2(C)
EXPNO                    1
PROCNO                   1

F2 - Acquisition Parameters
Date_            20150625
Time                17.21 h
INSTRUM             spect
PROBHD   Z108618_0257 (
PULPROG                zg
TD                  65536
SOLVENT              DMSO
NS                     54
DS                      4
SWH             25510.203 Hz
FIDRES           0.389255 Hz
AQ              1.2845056 sec
RG                    406
DW                 19.600 usec
DE                   6.50 usec
TE                  295.5 K
D1             1.00000000 sec
TD0                     1
SFO1          128.4096891 MHz
NUC1                  11B
P1                   7.50 usec
PLW1          55.09999847 W

F2 - Processing parameters
SI                  32768
SF            128.4096891 MHz
WDW                    EM
SSB      0
LB                   1.00 Hz
GB       0
PC                   1.40
```

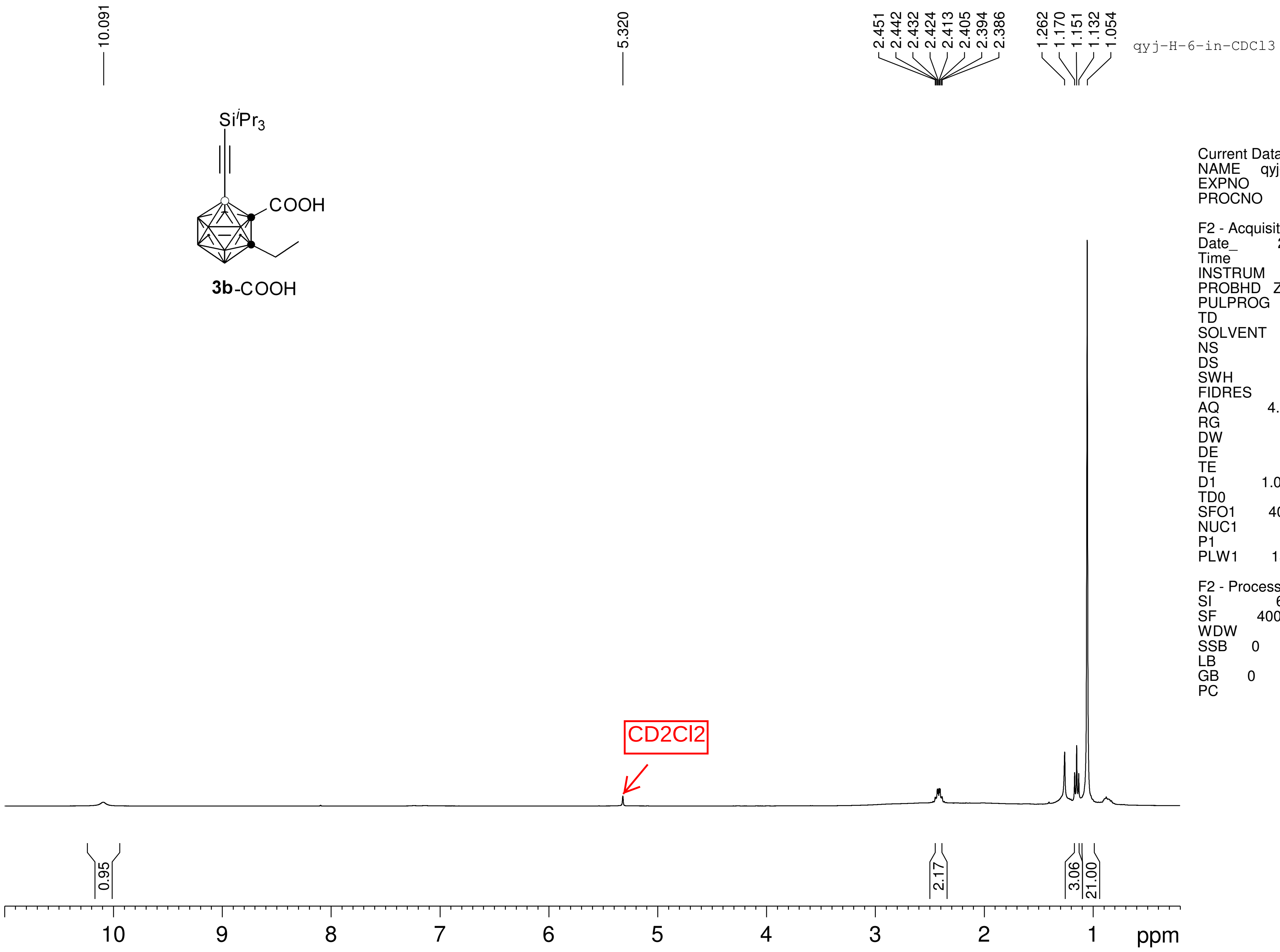

Current Data Parameters
NAME qyj-H-6-in-CDCl3
EXPNO 1
PROCNO 1

F2 - Acquisition Parameters
Date_ 20151002
Time 11.14 h
INSTRUM spect
PROBHD Z108618_0257 (
PULPROG zg30
TD 65536
SOLVENT CDCl3
NS 28
DS 2
SWH 8012.820 Hz
FIDRES 0.122266 Hz
AQ 4.0894465 sec
RG 80.6
DW 62.400 usec
DE 6.50 usec
TE 294.4 K
D1 1.00000000 sec
TD0 1
SFO1 400.2324714 MHz
NUC1 1H
P1 12.80 usec
PLW1 13.56000042 W

F2 - Processing parameters
SI 65536
SF 400.2307835 MHz
WDW EM
SSB 0
LB 0.30 Hz
GB 0
PC 1.00

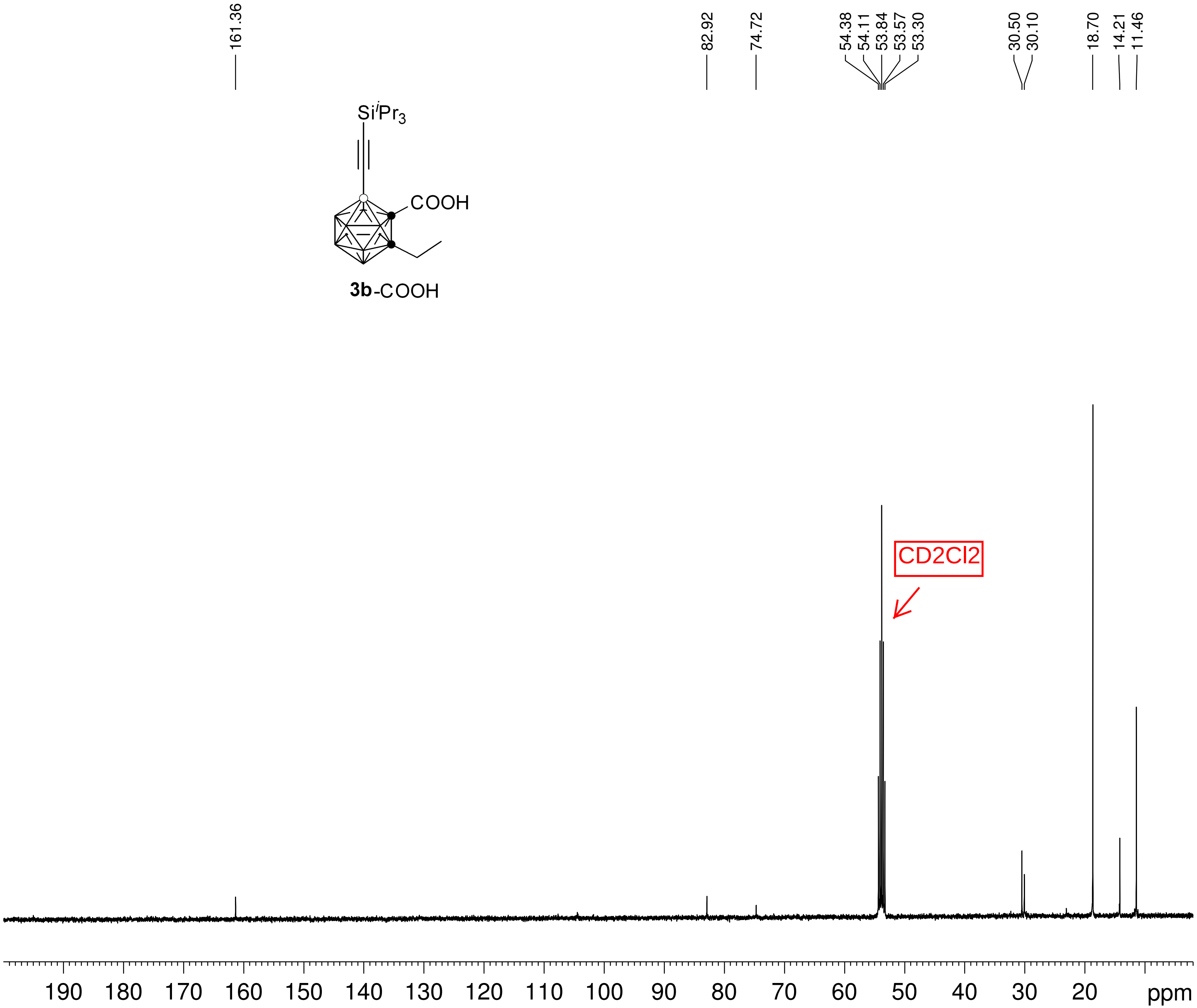

qyj-C-6-in-CDCl3

```
Current Data Parameters
NAME      qyj-C-6-in-CDCl3
EXPNO                    1
PROCNO                   1

F2 - Acquisition Parameters
Date_             20151002
Time                 12.43 h
INSTRUM              spect
PROBHD     Z108618_0257 (
PULPROG             zgpg30
TD                   65536
SOLVENT             CD2Cl2
NS                     710
DS                       4
SWH              29761.904 Hz
FIDRES            0.454131 Hz
AQ               1.1010048 sec
RG                     181
DW                  16.800 usec
DE                    6.50 usec
TE                   294.5 K
D1              2.00000000 sec
D11             0.03000000 sec
TD0                      1
SFO1           100.6479773 MHz
NUC1                   13C
P1                    9.50 usec
PLW1           55.34000015 W
SFO2           400.2316009 MHz
NUC2                    1H
CPDPRG[2           waltz16
PCPD2                90.00 usec
PLW2           13.56000042 W
PLW12           0.27428001 W
PLW13           0.13796000 W

F2 - Processing parameters
SI                   32768
SF             100.6378724 MHz
WDW                     EM
SSB      0
LB                    1.00 Hz
GB       0
PC                    1.40
```

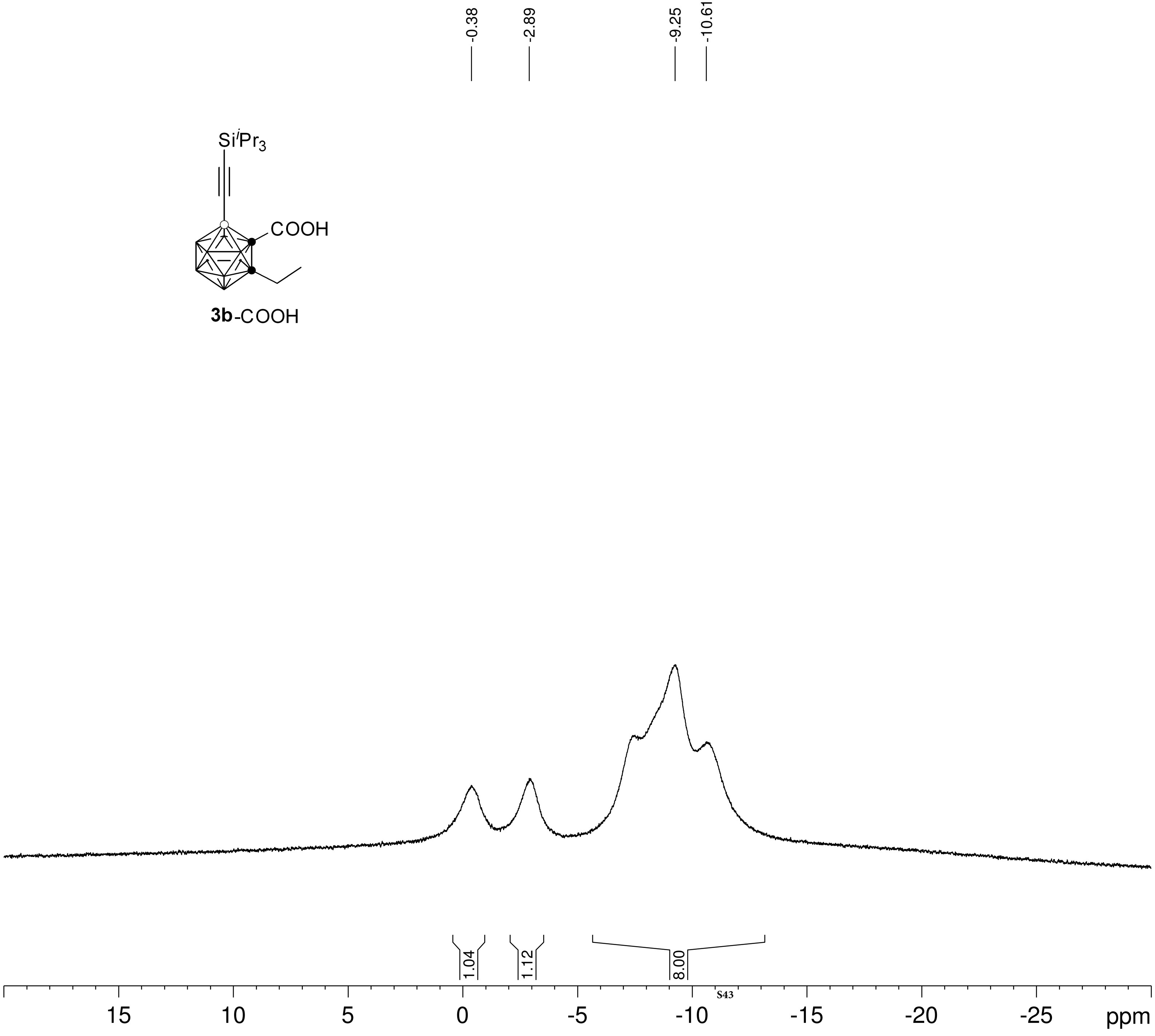

qyj-B-6-in-CDCl3

```
Current Data Parameters
NAME      qyj-B-6-in-CDCl3
EXPNO                    1
PROCNO                   1

F2 - Acquisition Parameters
Date_             20151002
Time                 11.05 h
INSTRUM              spect
PROBHD    Z108618_0257 (
PULPROG             zgpg30
TD                   65536
SOLVENT              CDCl3
NS                      40
DS                       2
SWH              29761.904 Hz
FIDRES            0.454131 Hz
AQ               1.1010048 sec
RG                     512
DW                  16.800 usec
DE                    6.50 usec
TE                   294.5 K
D1              1.00000000 sec
D11             0.03000000 sec
TD0                      1
SFO1           128.4096890 MHz
NUC1                   11B
P1                    7.50 usec
PLW1           55.09999847 W
SFO2           400.2316008 MHz
NUC2                    1H
CPDPRG[2           waltz16
PCPD2                90.00 usec
PLW2           13.56000042 W
PLW12           0.27428001 W
PLW13           0.13796000 W

F2 - Processing parameters
SI                   32768
SF             128.4095347 MHz
WDW                     EM
SSB       0
LB                    1.00 Hz
GB        0
PC                    1.40
```

qyj-B-6-in-CDCl3(C)

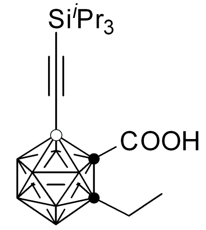

3b-COOH

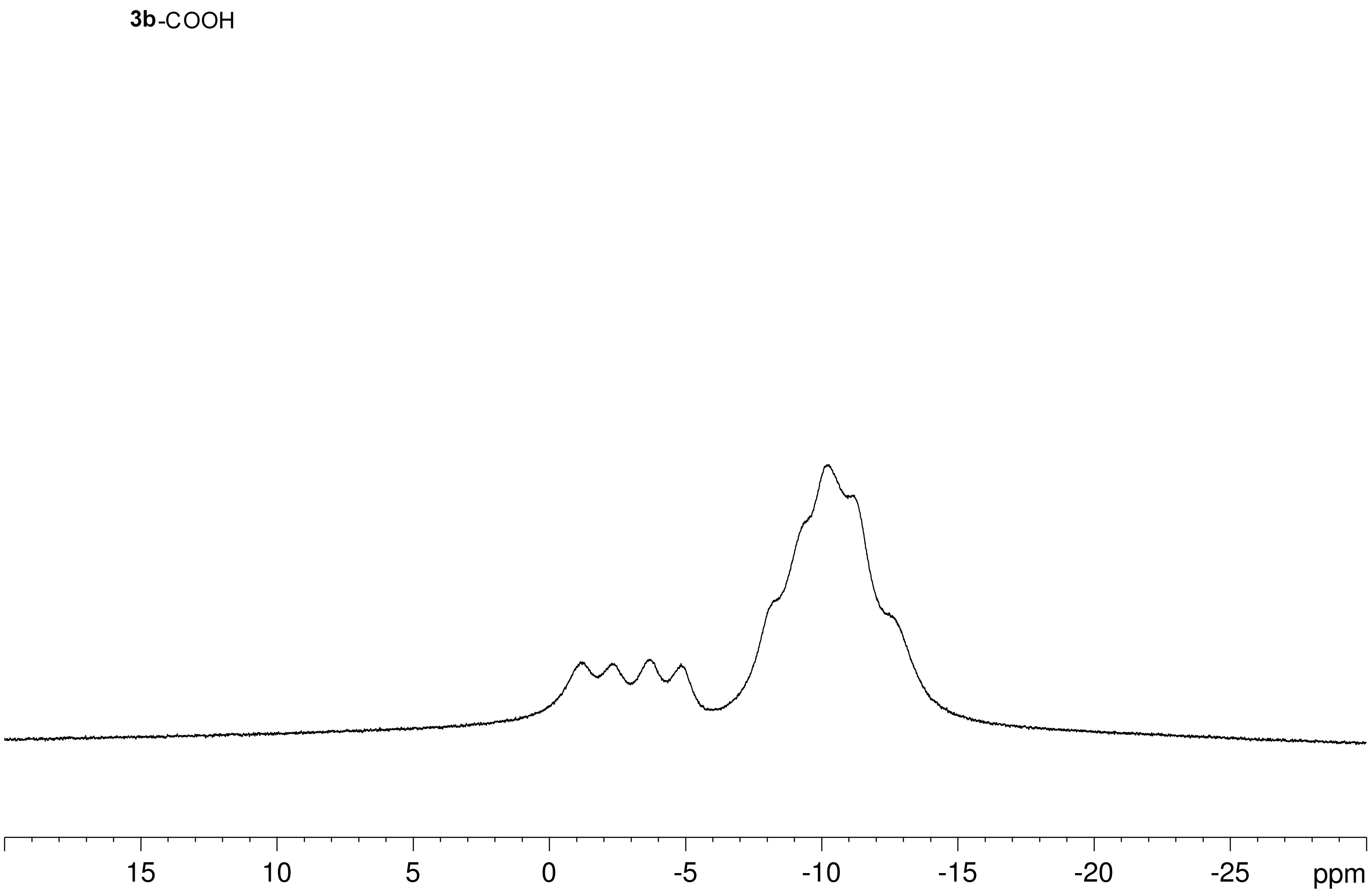


```
Current Data Parameters
NAME     qyj-B-6-in-CDCl3(C)
EXPNO                  1
PROCNO                 1

F2 - Acquisition Parameters
Date_            20151002
Time                11.08 h
INSTRUM             spect
PROBHD   Z108618_0257 (
PULPROG                zg
TD                  65536
SOLVENT             CDCl3
NS                     40
DS                      2
SWH             24038.461 Hz
FIDRES           0.366798 Hz
AQ              1.3631488 sec
RG                    362
DW                 20.800 usec
DE                   6.50 usec
TE                  294.4 K
D1             2.00000000 sec
TD0                     1
SFO1          128.4096890 MHz
NUC1                  11B
P1                   7.50 usec
PLW1          55.09999847 W

F2 - Processing parameters
SI                  32768
SF            128.4097117 MHz
WDW                    EM
SSB      0
LB                   1.00 Hz
GB       0
PC                   1.40
```

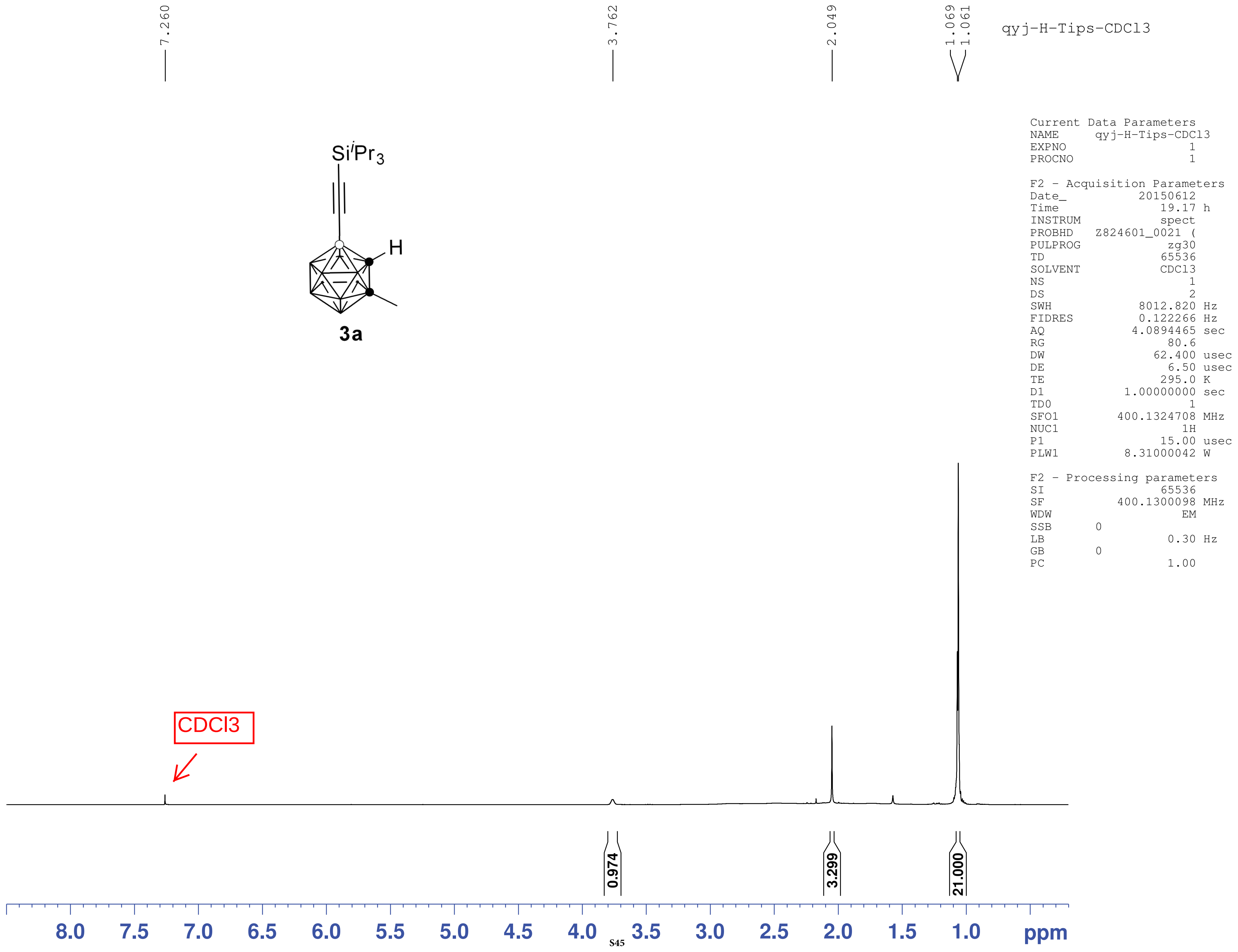
7.260
3.762
2.049
1.069
1.061
qyj-H-Tips-CDCl3
Si^{i}Pr$_3$
H
3a
Current Data Parameters
NAME qyj-H-Tips-CDCl3
EXPNO 1
PROCNO 1
F2 - Acquisition Parameters
Date_ 20150612
Time 19.17 h
INSTRUM spect
PROBHD Z824601_0021 (
PULPROG zg30
TD 65536
SOLVENT CDCl3
NS 1
DS 2
SWH 8012.820 Hz
FIDRES 0.122266 Hz
AQ 4.0894465 sec
RG 80.6
DW 62.400 usec
DE 6.50 usec
TE 295.0 K
D1 1.00000000 sec
TD0 1
SFO1 400.1324708 MHz
NUC1 1H
P1 15.00 usec
PLW1 8.31000042 W
F2 - Processing parameters
SI 65536
SF 400.1300098 MHz
WDW EM
SSB 0
LB 0.30 Hz
GB 0
PC 1.00
CDCl3
0.974
3.299
21.000
8.0
7.5
7.0
6.5
6.0
5.5
5.0
4.5
4.0
3.5
3.0
2.5
2.0
1.5
1.0
ppm

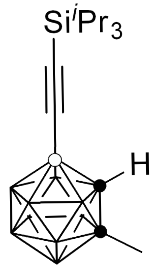

3a

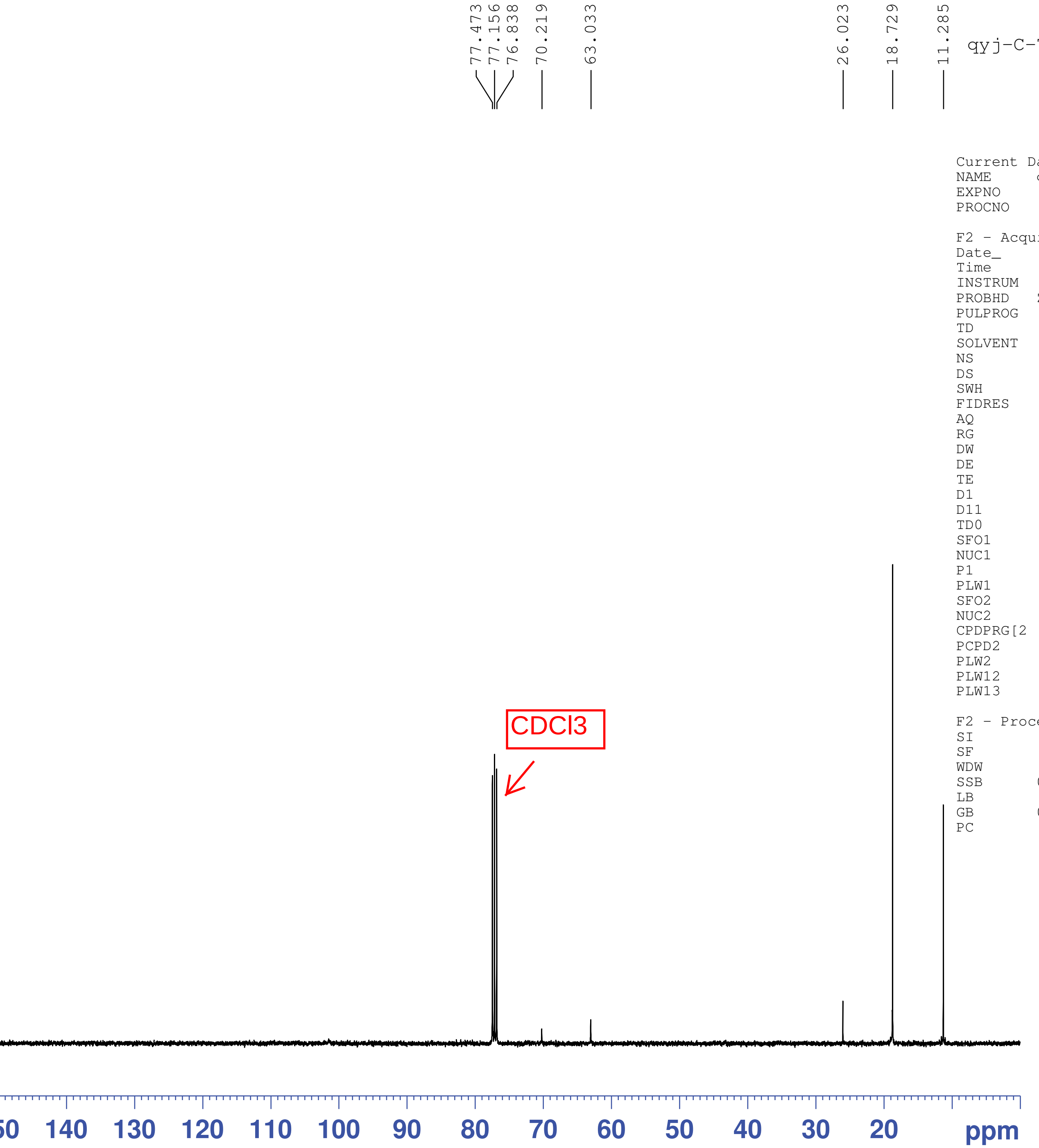

qyj-C-Tips-CDCl3

Current Data Parameters
NAME qyj-C-Tips-CDCl3
EXPNO 1
PROCNO 1

F2 - Acquisition Parameters
Date_ 20150612
Time 19.20 h
INSTRUM spect
PROBHD Z824601_0021 (
PULPROG zgpg30
TD 65536
SOLVENT CDCl3
NS 226
DS 4
SWH 24038.461 Hz
FIDRES 0.366798 Hz
AQ 1.3631488 sec
RG 203
DW 20.800 usec
DE 6.50 usec
TE 295.2 K
D1 2.00000000 sec
D11 0.03000000 sec
TD0 1
SFO1 100.6228298 MHz
NUC1 13C
P1 9.50 usec
PLW1 41.25000000 W
SFO2 400.1316005 MHz
NUC2 1H
CPDPRG[2 waltz16
PCPD2 90.00 usec
PLW2 8.31000042 W
PLW12 0.23083000 W
PLW13 0.11611000 W

F2 - Processing parameters
SI 32768
SF 100.6127557 MHz
WDW EM
SSB 0
LB 1.00 Hz
GB 0
PC 1.40

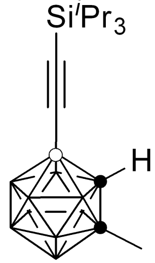

3a

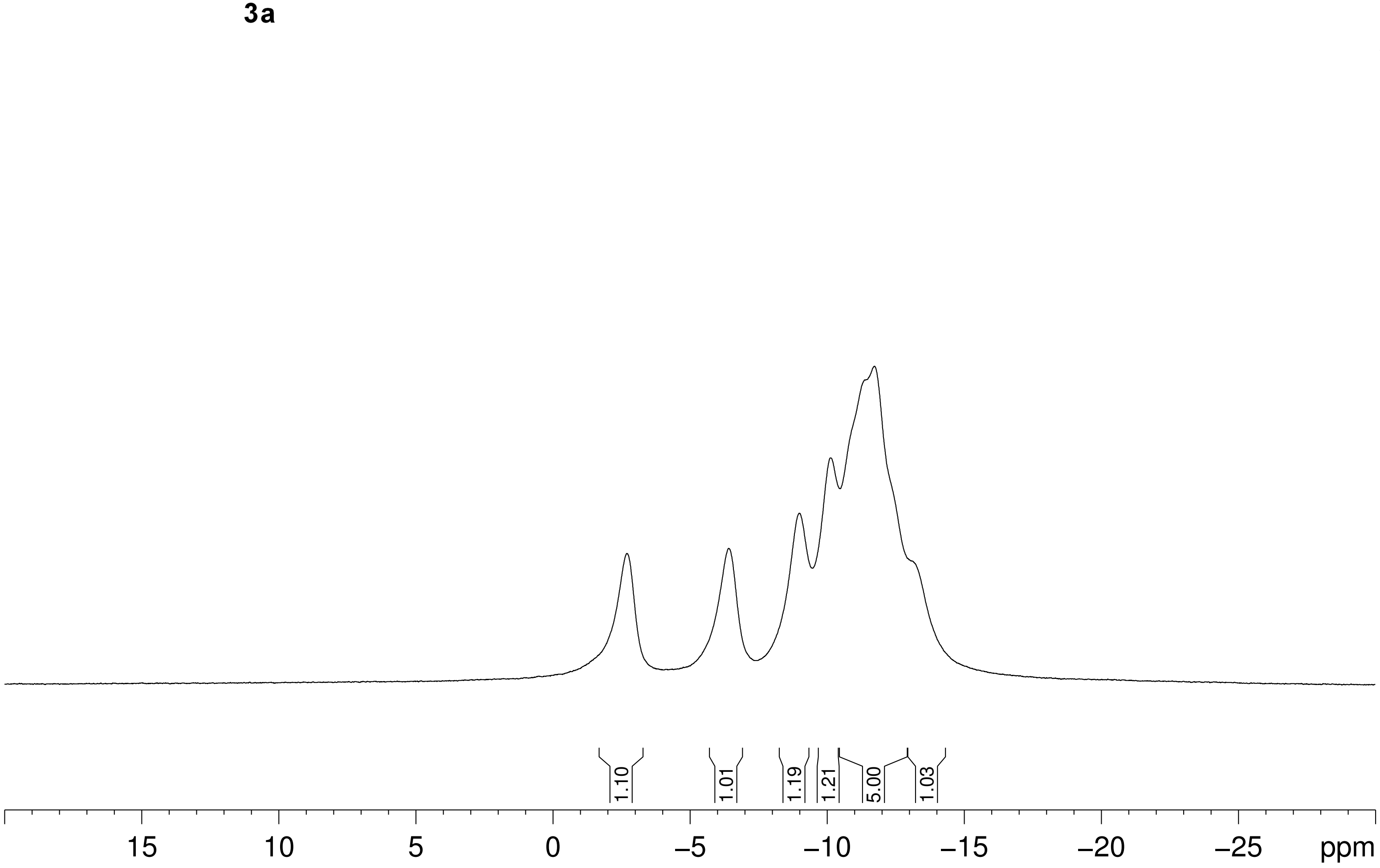

qyj-B-0358-CD2Cl2

```
NAME      qyj-B-0358-CD2Cl2
EXPNO                     1
PROCNO                    1
Date_              20141103
Time                  19.50
INSTRUM               spect
PROBHD    5 mm PABBO BB-
PULPROG                zgdc
TD                    65536
SOLVENT                C6D6
NS                        8
DS                        0
SWH               25510.203 Hz
FIDRES             0.389255 Hz
AQ                1.2845556 sec
RG                      181
DW                   19.600 usec
DE                     6.50 usec
TE                    299.5 K
D1               5.00000000 sec
D11              0.03000000 sec
TD0                       1

======== CHANNEL f1 ========
NUC1                    11B
P1                     7.60 usec
PL1                   -3.00 dB
PL1W            55.13059616 W
SFO1            128.3968556 MHz

======== CHANNEL f2 ========
CPDPRG2             waltz16
NUC2                     1H
PCPD2                 90.00 usec
PL2                   -1.00 dB
PL12                  15.16 dB
PL2W            13.56617069 W
PL12W            0.32844096 W
SFO2            400.1916008 MHz
SI                    32768
SF              128.3969291 MHz
WDW                      EM
SSB                       0
LB                     3.00 Hz
GB                        0
PC                     1.40
```

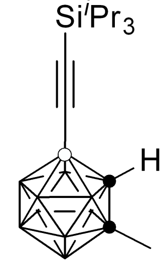

3a

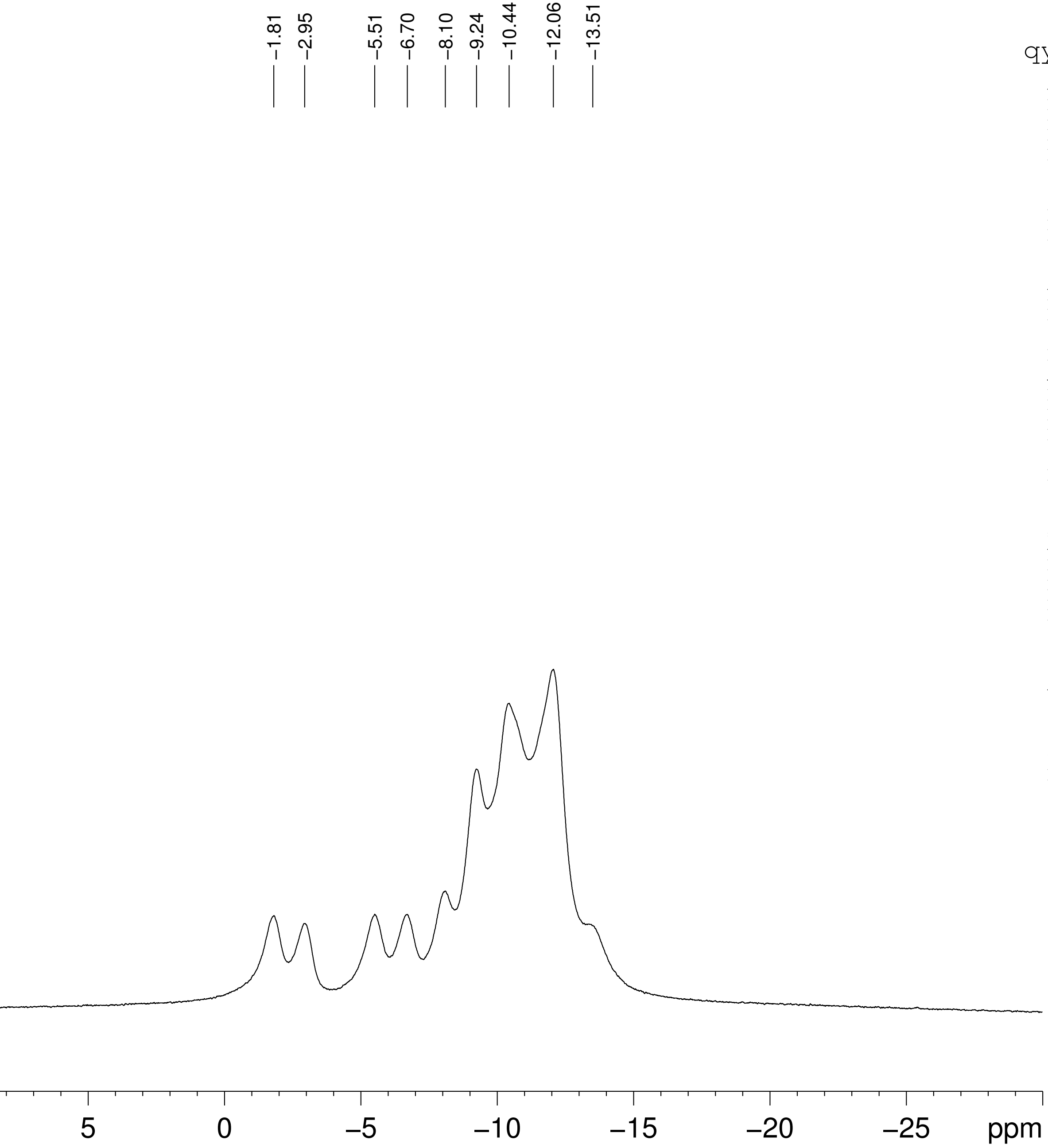

qyj-B-0358-CD2Cl2(C

NAME	qyj-B-0358-CD2Cl2(C)
EXPNO	1
PROCNO	1
Date_	20141103
Time	19.52
INSTRUM	spect
PROBHD	5 mm PABBO BB-
PULPROG	zg30
TD	65536
SOLVENT	C6D6
NS	20
DS	0
SWH	25510.203 Hz
FIDRES	0.389255 Hz
AQ	1.2845556 sec
RG	362
DW	19.600 usec
DE	6.50 usec
TE	299.1 K
D1	5.00000000 sec
TD0	1
======== CHANNEL f1 ========	
NUC1	11B
P1	7.60 usec
PL1	-3.00 dB
PL1W	55.13059616 W
SFO1	128.3968556 MHz
SI	32768
SF	128.3968865 MHz
WDW	EM
SSB	0
LB	3.00 Hz
GB	0
PC	1.40

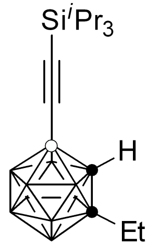

3b

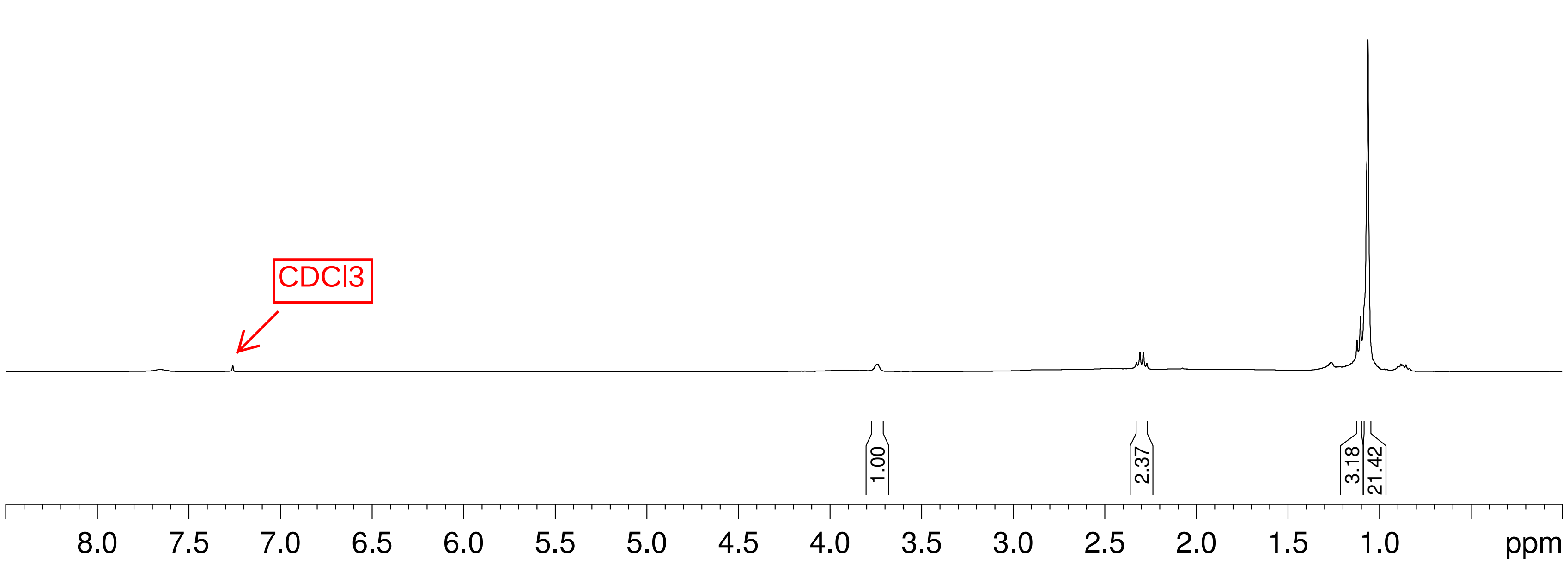

NAME	qyj–H–0369–CDCl3
EXPNO	1
PROCNO	1
Date_	20150516
Time	16.03
INSTRUM	spect
PROBHD	5 mm PABBO BB–
PULPROG	zg30
TD	65536
SOLVENT	CDCl3
NS	9
DS	2
SWH	8223.685 Hz
FIDRES	0.125483 Hz
AQ	3.9846387 sec
RG	40.3
DW	60.800 usec
DE	6.50 usec
TE	298.3 K
D1	1.00000000 sec
TD0	1

======== CHANNEL f1 ======

NUC1	1H
P1	14.00 usec
PL1	–1.00 dB
PL1W	13.56617069 W
SFO1	400.1924713 MHz
SI	32768
SF	400.1900154 MHz
WDW	EM
SSB	0
LB	0.30 Hz
GB	0
PC	1.00

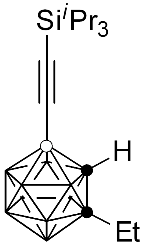

3b

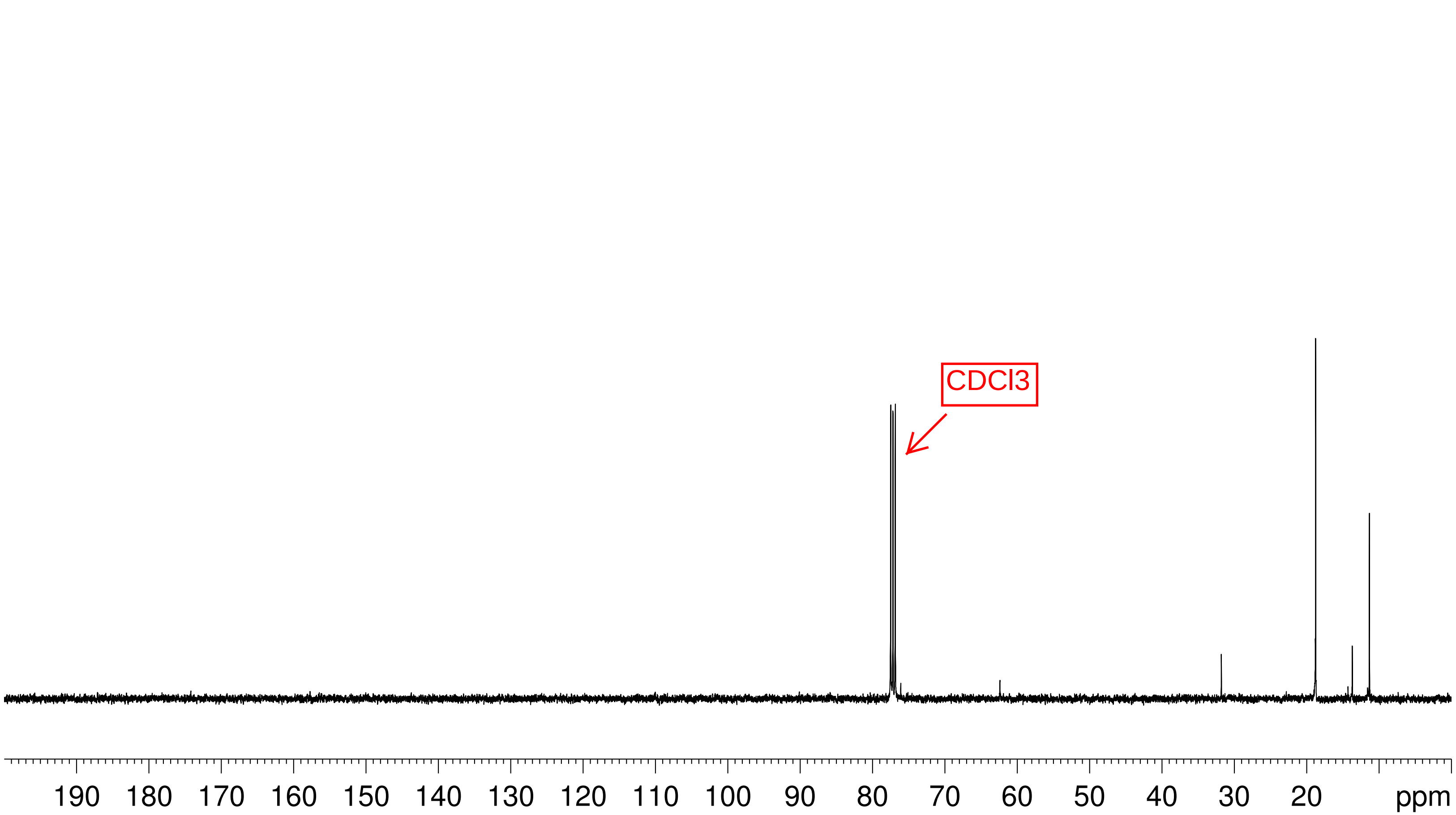

qyj-C-0456-CDCl3

NAME	qyj-C-0369-CDCl3	
EXPNO	1	
PROCNO	1	
Date_	20150516	
Time	16.07	
INSTRUM	spect	
PROBHD	5 mm PABBO BB-	
PULPROG	zgpg30	
TD	65536	
SOLVENT	CDCl3	
NS	100	
DS	4	
SWH	24038.461	Hz
FIDRES	0.366798	Hz
AQ	1.3631988	sec
RG	2050	
DW	20.800	usec
DE	6.50	usec
TE	298.6	K
D1	2.00000000	sec
D11	0.03000000	sec
TD0	1	

======== CHANNEL f1 ========

NUC1	13C	
P1	9.90	usec
PL1	-2.00	dB
PL1W	55.33689499	W
SFO1	100.6379183	MHz

======== CHANNEL f2 ========

CPDPRG2	waltz16	
NUC2	1H	
PCPD2	90.00	usec
PL2	-1.00	dB
PL12	15.16	dB
PL13	18.62	dB
PL2W	13.56617069	W
PL12W	0.32844096	W
PL13W	0.14806664	W
SFO2	400.1916008	MHz
SI	32768	
SF	100.6278431	MHz
WDW	EM	
SSB	0	
LB	1.00	Hz
GB	0	
PC	1.40	

qyj-B-0369-CDCl3

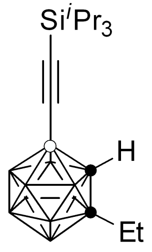

3b

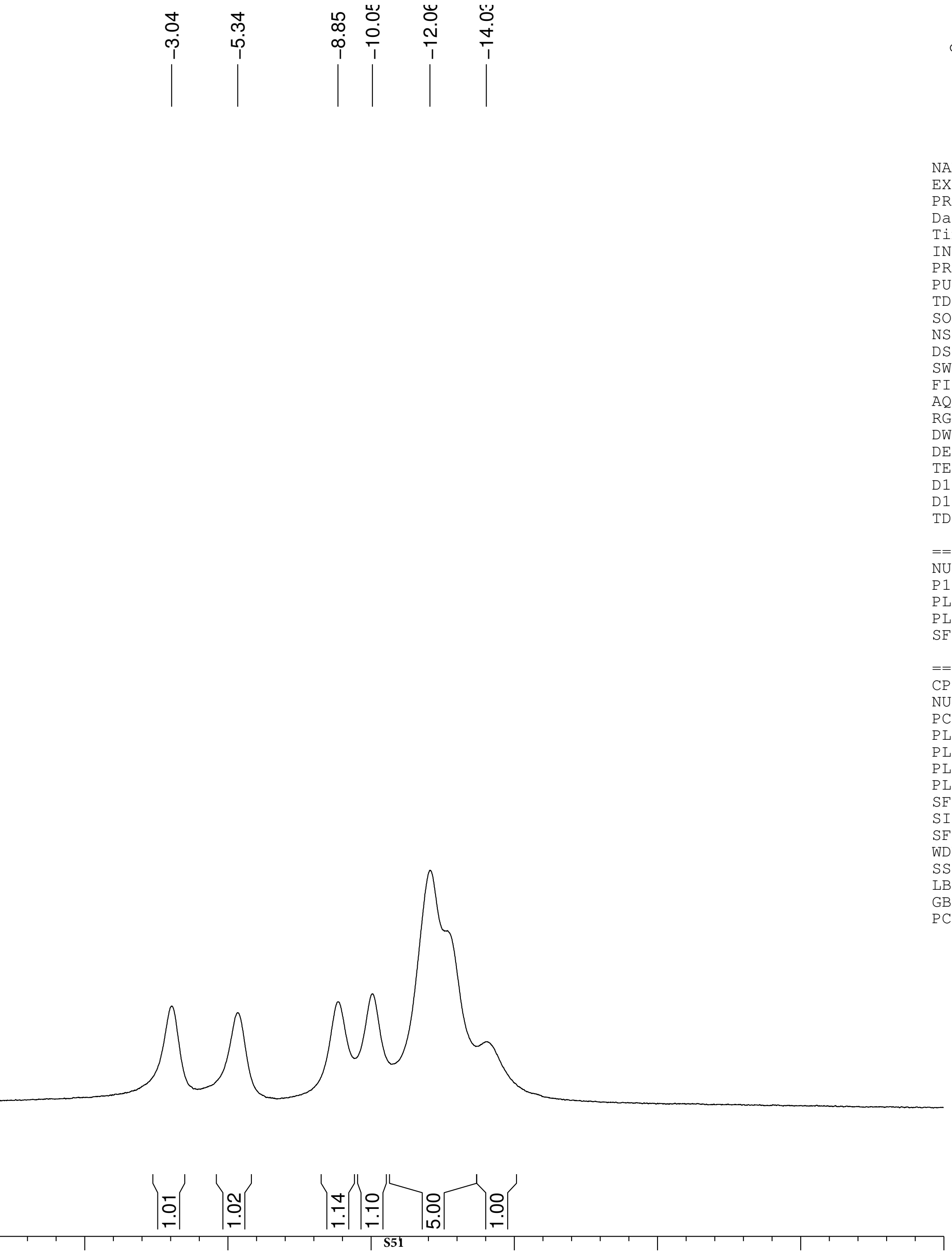

NAME	qyj-B-0369-CDCl3
EXPNO	1
PROCNO	1
Date_	20150516
Time	19.27
INSTRUM	spect
PROBHD	5 mm PABBO BB-
PULPROG	zgdc
TD	65536
SOLVENT	C6D6
NS	12
DS	0
SWH	25510.203 Hz
FIDRES	0.389255 Hz
AQ	1.2845556 sec
RG	362
DW	19.600 usec
DE	6.50 usec
TE	299.0 K
D1	5.00000000 sec
D11	0.03000000 sec
TD0	1
======== CHANNEL f1 ========	
NUC1	11B
P1	7.60 usec
PL1	-3.00 dB
PL1W	55.13059616 W
SFO1	128.3968556 MHz
======== CHANNEL f2 ========	
CPDPRG2	waltz16
NUC2	1H
PCPD2	90.00 usec
PL2	-1.00 dB
PL12	15.16 dB
PL2W	13.56617069 W
PL12W	0.32844096 W
SFO2	400.1916008 MHz
SI	32768
SF	128.3968847 MHz
WDW	EM
SSB	0
LB	3.00 Hz
GB	0
PC	1.40

qyj-B-0369-CDCl3(C)

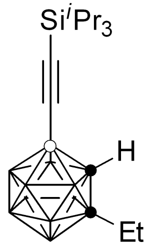

3b

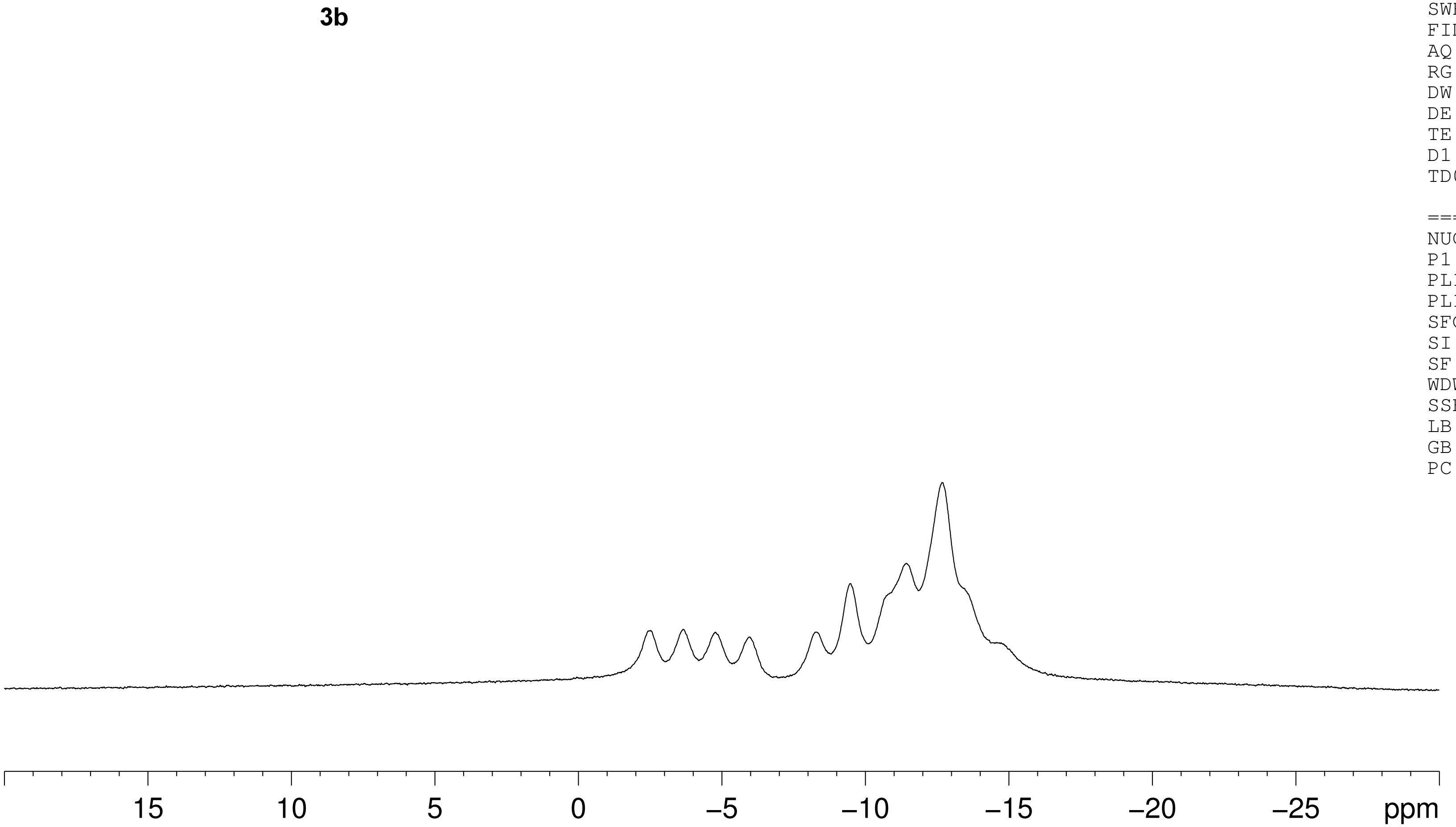

NAME	qyj-B-0369-CDCl3(C)	
EXPNO	1	
PROCNO	1	
Date_	20150516	
Time	19.29	
INSTRUM	spect	
PROBHD	5 mm PABBO BB-	
PULPROG	zg30	
TD	65536	
SOLVENT	C6D6	
NS	16	
DS	0	
SWH	25510.203	Hz
FIDRES	0.389255	Hz
AQ	1.2845556	sec
RG	512	
DW	19.600	usec
DE	6.50	usec
TE	298.7	K
D1	5.00000000	sec
TD0	1	

======== CHANNEL f1 ========

NUC1	11B	
P1	7.60	usec
PL1	-3.00	dB
PL1W	55.13059616	W
SFO1	128.3968556	MHz
SI	32768	
SF	128.3968865	MHz
WDW	EM	
SSB	0	
LB	3.00	Hz
GB	0	
PC	1.40	

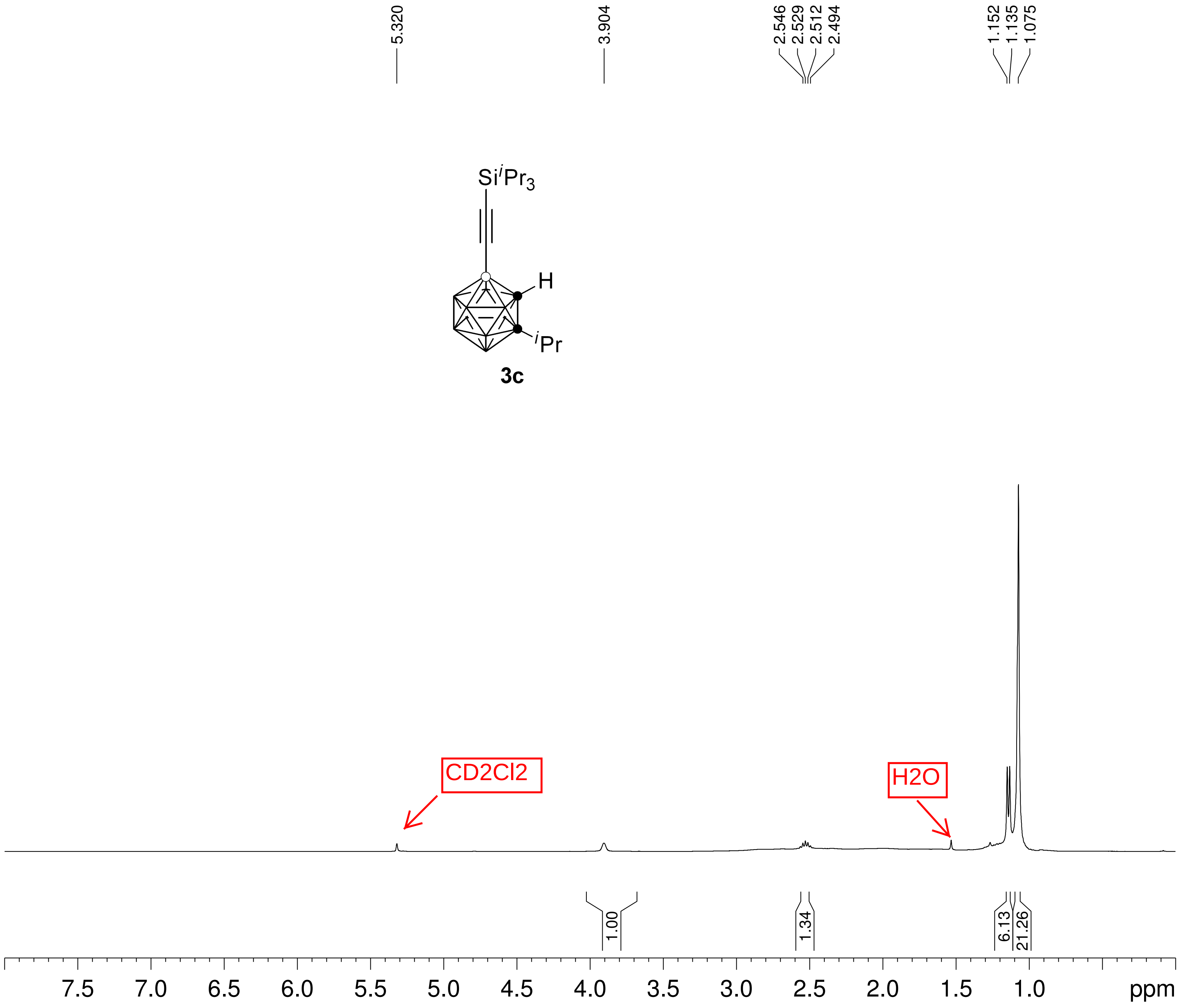

qyj-H-0360-CD2Cl2

NAME	qyj–H–0360–CD2Cl2
EXPNO	1
PROCNO	1
Date_	20141103
Time	17.19
INSTRUM	spect
PROBHD	5 mm PABBO BB–
PULPROG	zg30
TD	65536
SOLVENT	CD2Cl2
NS	12
DS	2
SWH	8223.685 Hz
FIDRES	0.125483 Hz
AQ	3.9846387 sec
RG	32
DW	60.800 usec
DE	6.50 usec
TE	298.7 K
D1	1.00000000 sec
TD0	1

======== CHANNEL f1 =====

NUC1	1H
P1	14.00 usec
PL1	–1.00 dB
PL1W	13.56617069 W
SFO1	400.1924713 MHz
SI	32768
SF	400.1900209 MHz
WDW	EM
SSB	0
LB	0.30 Hz
GB	0
PC	1.00

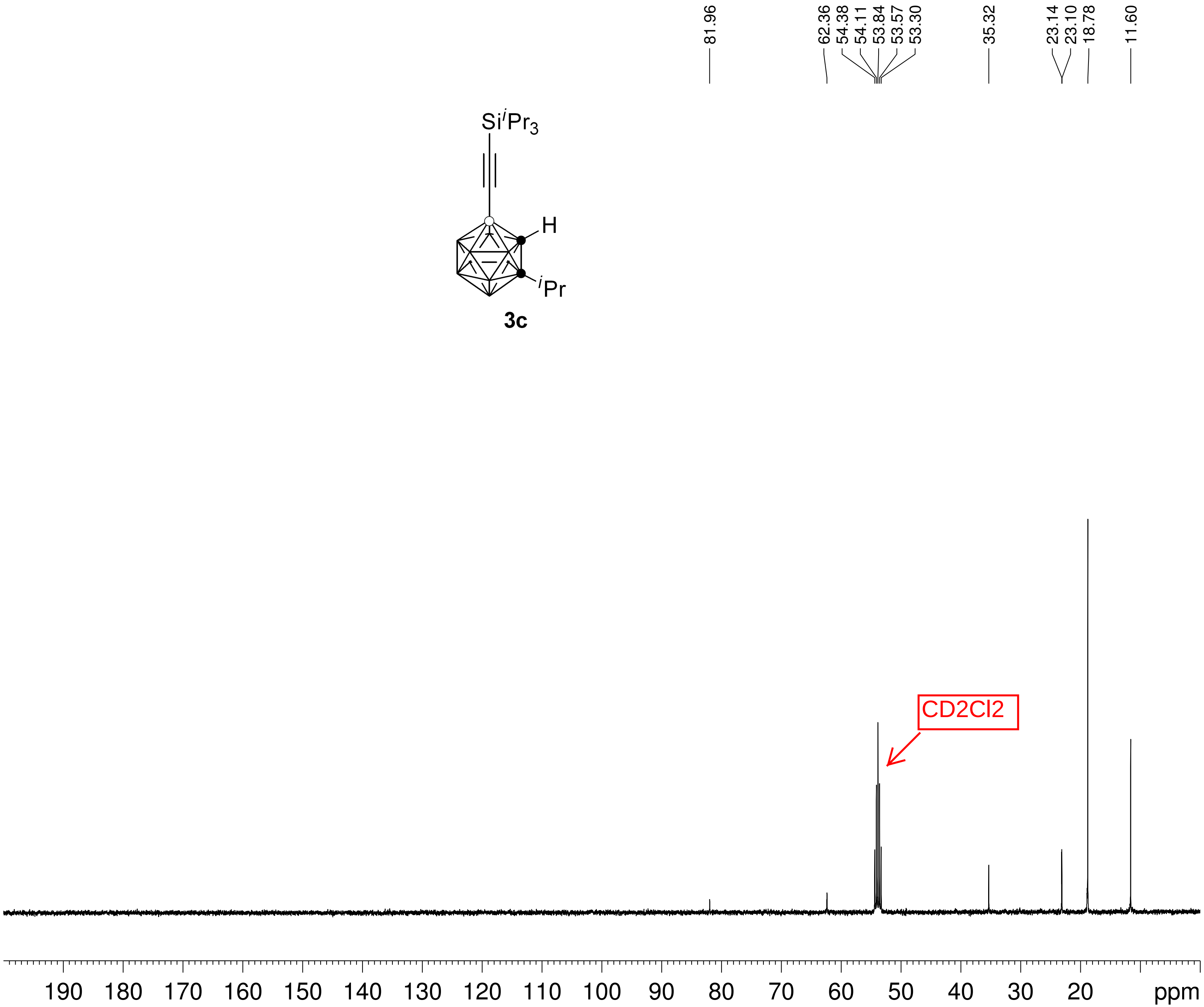

qyj-C-0360-CD2Cl2

```
NAME        qyj-C-0360-CD2Cl2
EXPNO                       1
PROCNO                      1
Date_                20141103
Time                    17.25
INSTRUM                 spect
PROBHD       5 mm PABBO BB-
PULPROG               zgpg30
TD                      65536
SOLVENT                CD2Cl2
NS                         96
DS                          4
SWH                 24038.461 Hz
FIDRES               0.366798 Hz
AQ                  1.3631988 sec
RG                        181
DW                     20.800 usec
DE                       6.50 usec
TE                      299.0 K
D1                 2.00000000 sec
D11                0.03000000 sec
TD0                         1

======== CHANNEL f1 ========
NUC1                      13C
P1                       9.90 usec
PL1                     -2.00 dB
PL1W              55.33689499 W
SFO1             100.6379183 MHz

======== CHANNEL f2 ========
CPDPRG2               waltz16
NUC2                       1H
PCPD2                   90.00 usec
PL2                     -1.00 dB
PL12                    15.16 dB
PL13                    18.62 dB
PL2W              13.56617069 W
PL12W              0.32844096 W
PL13W              0.14806664 W
SFO2             400.1916008 MHz
SI                      32768
SF               100.6278125 MHz
WDW                        EM
SSB                         0
LB                       1.00 Hz
GB                          0
PC                       1.40
```

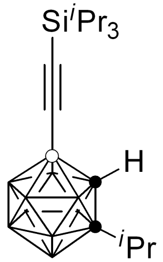

3c

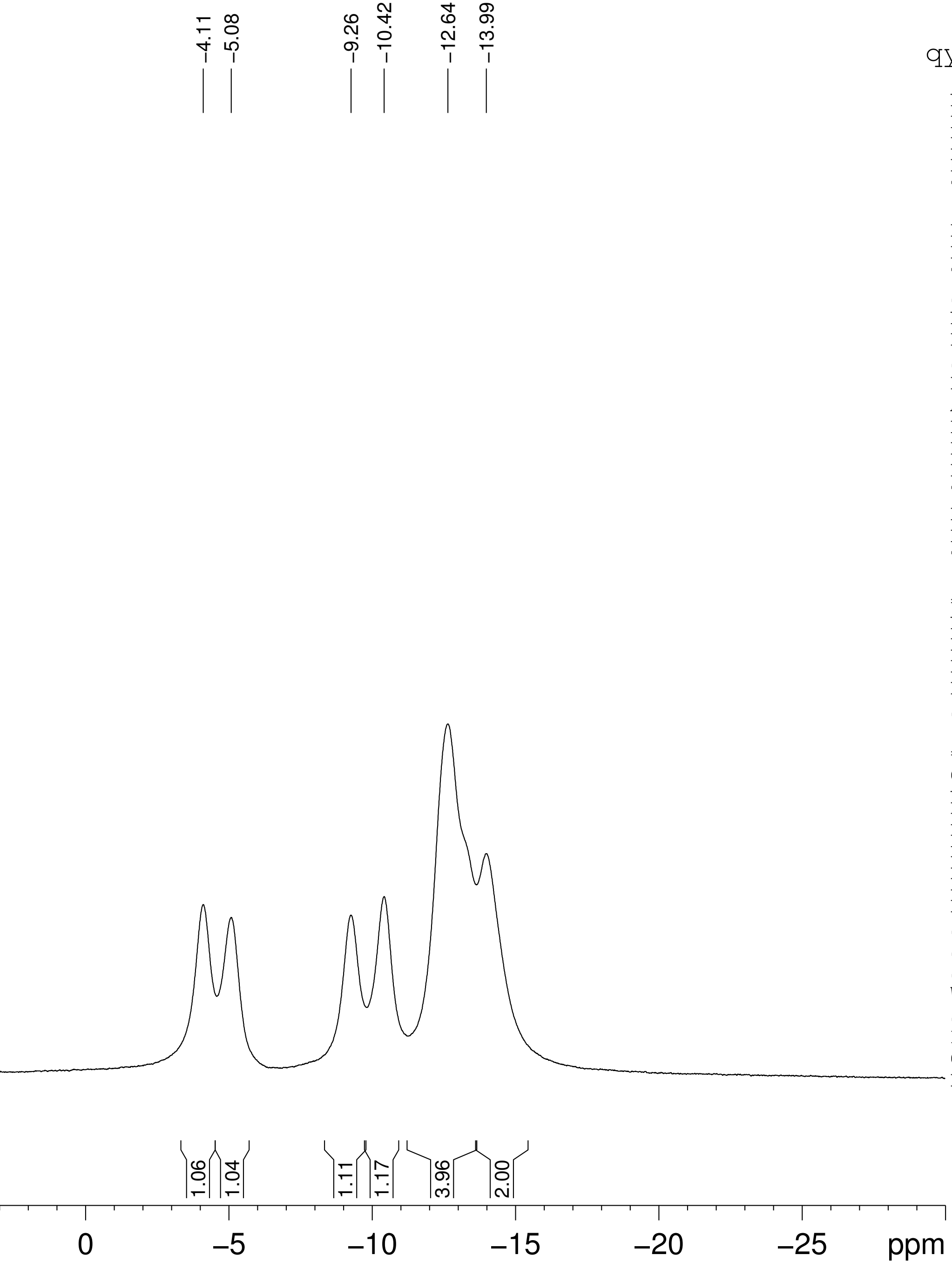

qyj-B-0360-CD2Cl2

```
NAME        qyj-B-0360-CD2Cl2
EXPNO                    1
PROCNO                   1
Date_             20141103
Time                 17.29
INSTRUM              spect
PROBHD    5 mm PABBO BB-
PULPROG               zgdc
TD                   65536
SOLVENT             CD2Cl2
NS                       8
DS                       0
SWH              25510.203 Hz
FIDRES            0.389255 Hz
AQ               1.2845556 sec
RG                     228
DW                  19.600 usec
DE                    6.50 usec
TE                   299.1 K
D1              5.00000000 sec
D11             0.03000000 sec
TD0                      1

======== CHANNEL f1 ========
NUC1                   11B
P1                    7.60 usec
PL1                  -3.00 dB
PL1W           55.13059616 W
SFO1           128.3968556 MHz

======== CHANNEL f2 ========
CPDPRG2            waltz16
NUC2                    1H
PCPD2                90.00 usec
PL2                  -1.00 dB
PL12                 15.16 dB
PL2W           13.56617069 W
PL12W           0.32844096 W
SFO2           400.1916008 MHz
SI                   32768
SF             128.3969291 MHz
WDW                     EM
SSB                      0
LB                    3.00 Hz
GB                       0
PC                    1.40
```

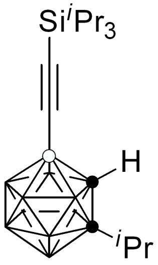

3c

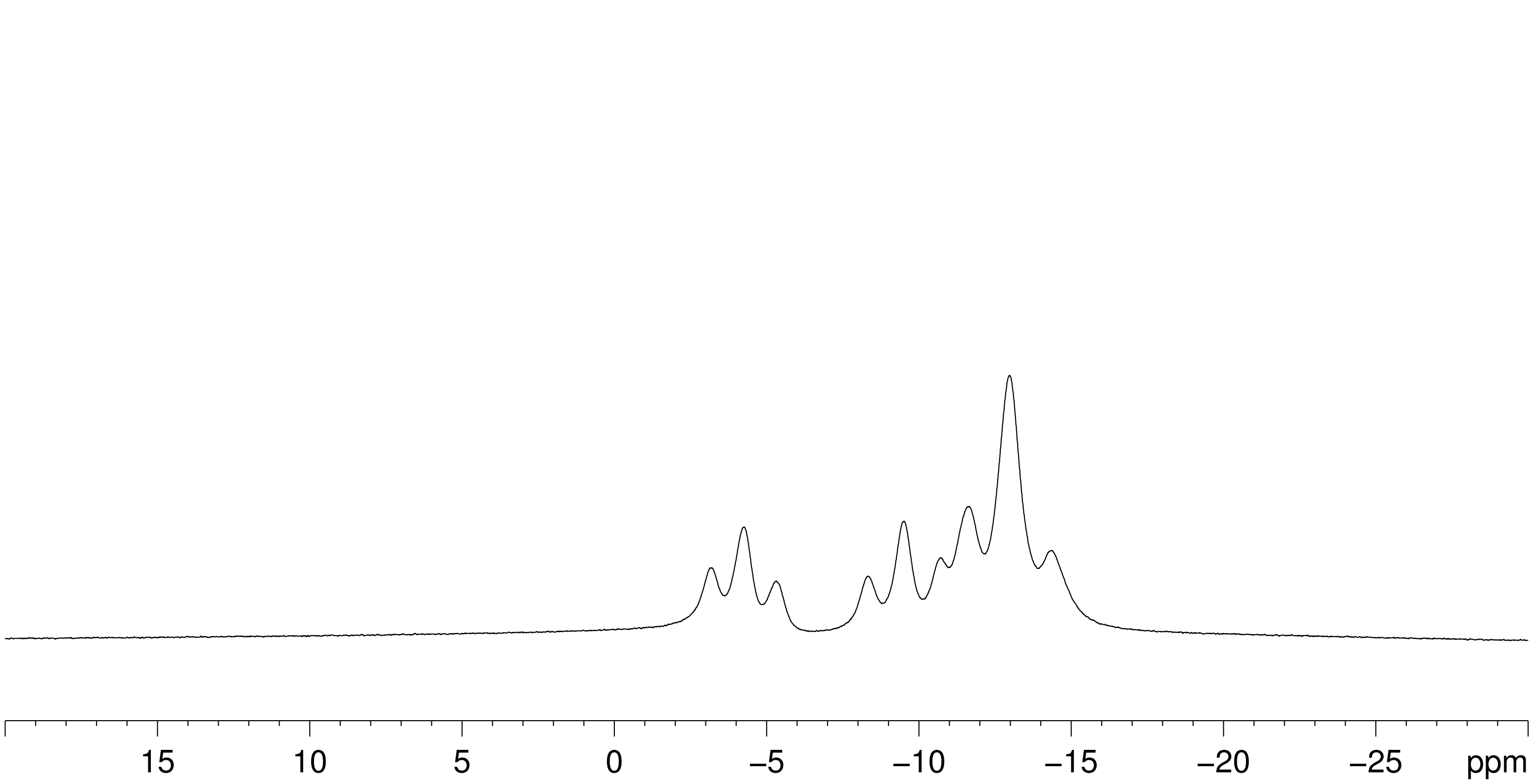

qyj-B-0360-CD2Cl2(C

NAME	qyj-B-0360-CD2Cl2(C)
EXPNO	1
PROCNO	1
Date_	20141103
Time	17.31
INSTRUM	spect
PROBHD	5 mm PABBO BB-
PULPROG	zg30
TD	65536
SOLVENT	CD2Cl2
NS	16
DS	0
SWH	25510.203 Hz
FIDRES	0.389255 Hz
AQ	1.2845556 sec
RG	362
DW	19.600 usec
DE	6.50 usec
TE	298.9 K
D1	5.00000000 sec
TD0	1

======== CHANNEL f1 ========

NUC1	11B
P1	7.60 usec
PL1	-3.00 dB
PL1W	55.13059616 W
SFO1	128.3968556 MHz
SI	32768
SF	128.3968865 MHz
WDW	EM
SSB	0
LB	3.00 Hz
GB	0
PC	1.40

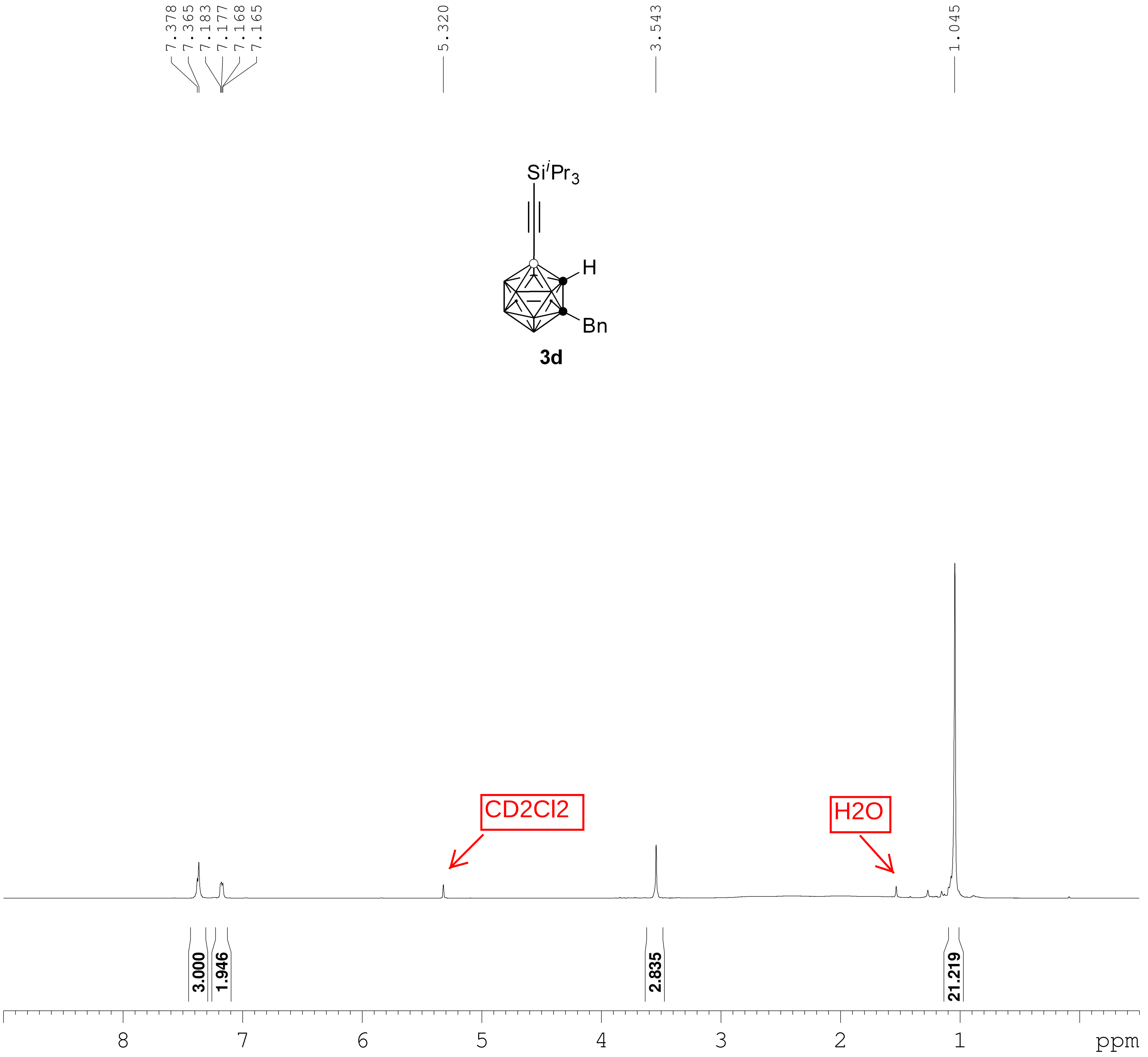

qyj-H-0363-cd2cl2

Bruker Advance III 400

NAME	qyj-H-0363-cd2cl2
EXPNO	1
PROCNO	1
Date_	20141108
Time	13.33
INSTRUM	spect
PROBHD	5 mm PADUL 13C
PULPROG	zg30
TD	65536
SOLVENT	CD2Cl2
NS	16
DS	2
SWH	8223.685 Hz
FIDRES	0.125483 Hz
AQ	3.9846387 sec
RG	128
DW	60.800 usec
DE	6.50 usec
TE	296.6 K
D1	1.00000000 sec
TD0	1

======== CHANNEL f1 ========

NUC1	1H
P1	14.83 usec
PL1	0.00 dB
PL1W	8.31434441 W
SFO1	400.1324710 MHz
SI	32768
SF	400.1300142 MHz
WDW	EM
SSB	0
LB	0.30 Hz
GB	0
PC	1.00

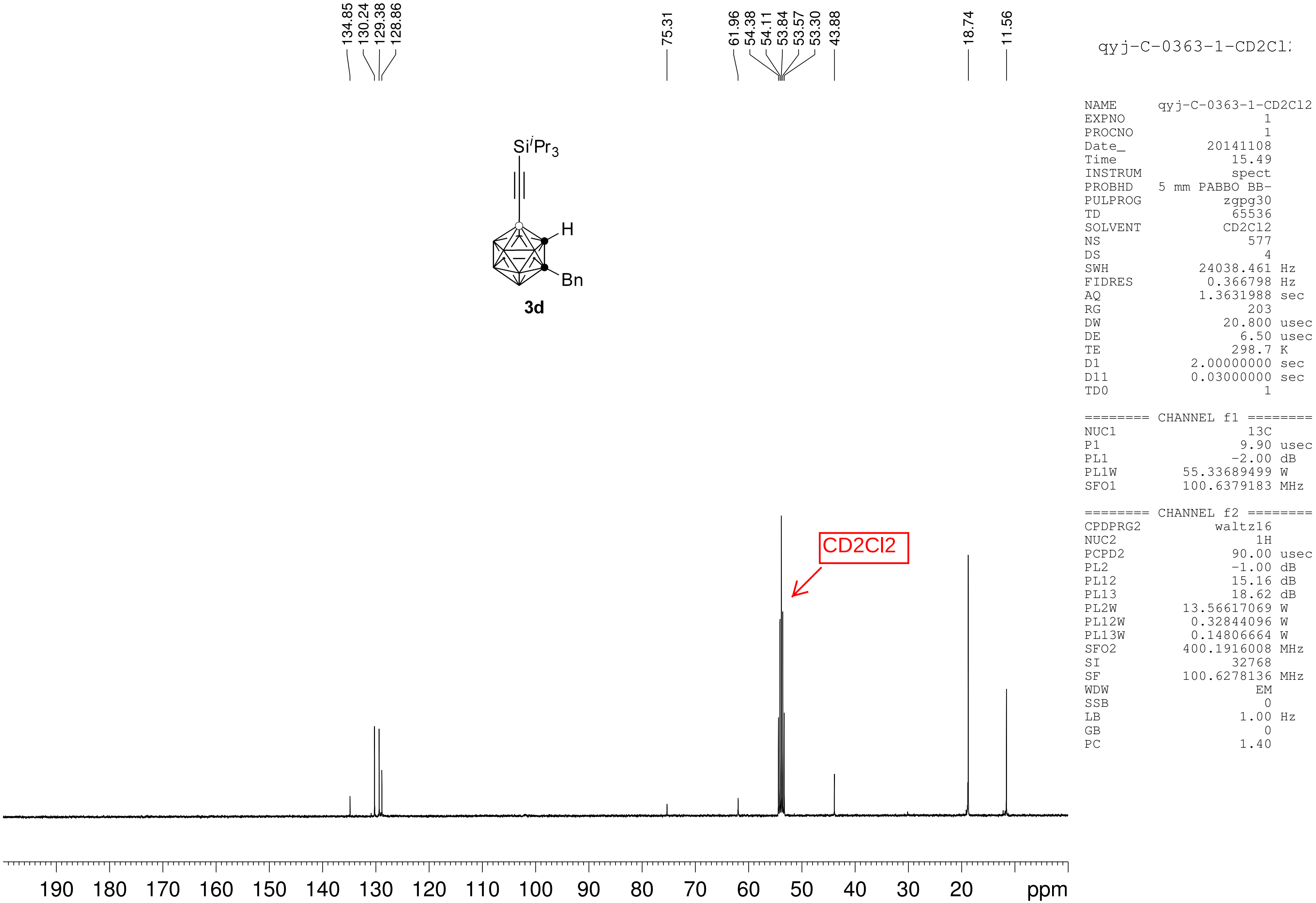
134.85
130.24
129.38
128.86
75.31
61.96
54.38
54.11
53.84
53.57
53.30
43.88
18.74
11.56
qyj-C-0363-1-CD2Cl:
Si^{i}Pr$_3$
H
Bn
3d
CD2Cl2
NAME qyj-C-0363-1-CD2Cl2
EXPNO 1
PROCNO 1
Date_ 20141108
Time 15.49
INSTRUM spect
PROBHD 5 mm PABBO BB-
PULPROG zgpg30
TD 65536
SOLVENT CD2Cl2
NS 577
DS 4
SWH 24038.461 Hz
FIDRES 0.366798 Hz
AQ 1.3631988 sec
RG 203
DW 20.800 usec
DE 6.50 usec
TE 298.7 K
D1 2.00000000 sec
D11 0.03000000 sec
TD0 1
======== CHANNEL f1 ========
NUC1 13C
P1 9.90 usec
PL1 -2.00 dB
PL1W 55.33689499 W
SFO1 100.6379183 MHz
======== CHANNEL f2 ========
CPDPRG2 waltz16
NUC2 1H
PCPD2 90.00 usec
PL2 -1.00 dB
PL12 15.16 dB
PL13 18.62 dB
PL2W 13.56617069 W
PL12W 0.32844096 W
PL13W 0.14806664 W
SFO2 400.1916008 MHz
SI 32768
SF 100.6278136 MHz
WDW EM
SSB 0
LB 1.00 Hz
GB 0
PC 1.40
190 180 170 160 150 140 130 120 110 100 90 80 70 60 50 40 30 20 ppm

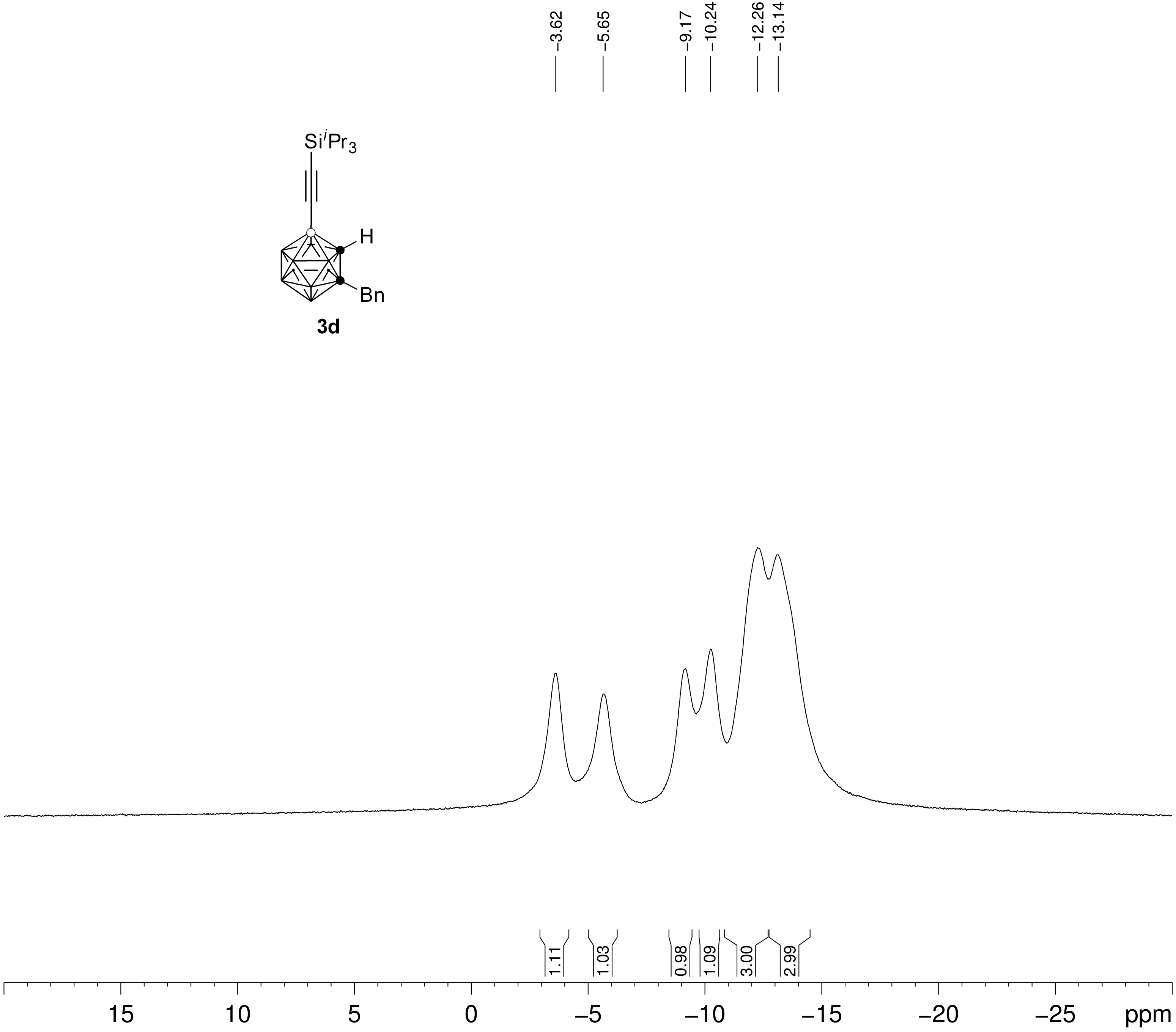

qyj-B-0363-1-CD2Cl2

NAME	qyj-B-0363-1-CD2Cl2
EXPNO	1
PROCNO	1
Date_	20141108
Time	15.33
INSTRUM	spect
PROBHD	5 mm PABBO BB-
PULPROG	zgdc
TD	65536
SOLVENT	CDCl3
NS	12
DS	0
SWH	25510.203 Hz
FIDRES	0.389255 Hz
AQ	1.2845556 sec
RG	181
DW	19.600 usec
DE	6.50 usec
TE	299.3 K
D1	5.00000000 sec
D11	0.03000000 sec
TD0	1

======== CHANNEL f1 ========

NUC1	11B
P1	7.60 usec
PL1	-3.00 dB
PL1W	55.13059616 W
SFO1	128.3968556 MHz

======== CHANNEL f2 ========

CPDPRG2	waltz16
NUC2	1H
PCPD2	90.00 usec
PL2	-1.00 dB
PL12	15.16 dB
PL2W	13.56617069 W
PL12W	0.32844096 W
SFO2	400.1916008 MHz
SI	32768
SF	128.3969291 MHz
WDW	EM
SSB	0
LB	3.00 Hz
GB	0
PC	1.40

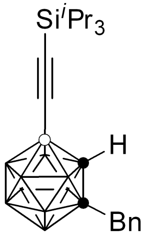

3d

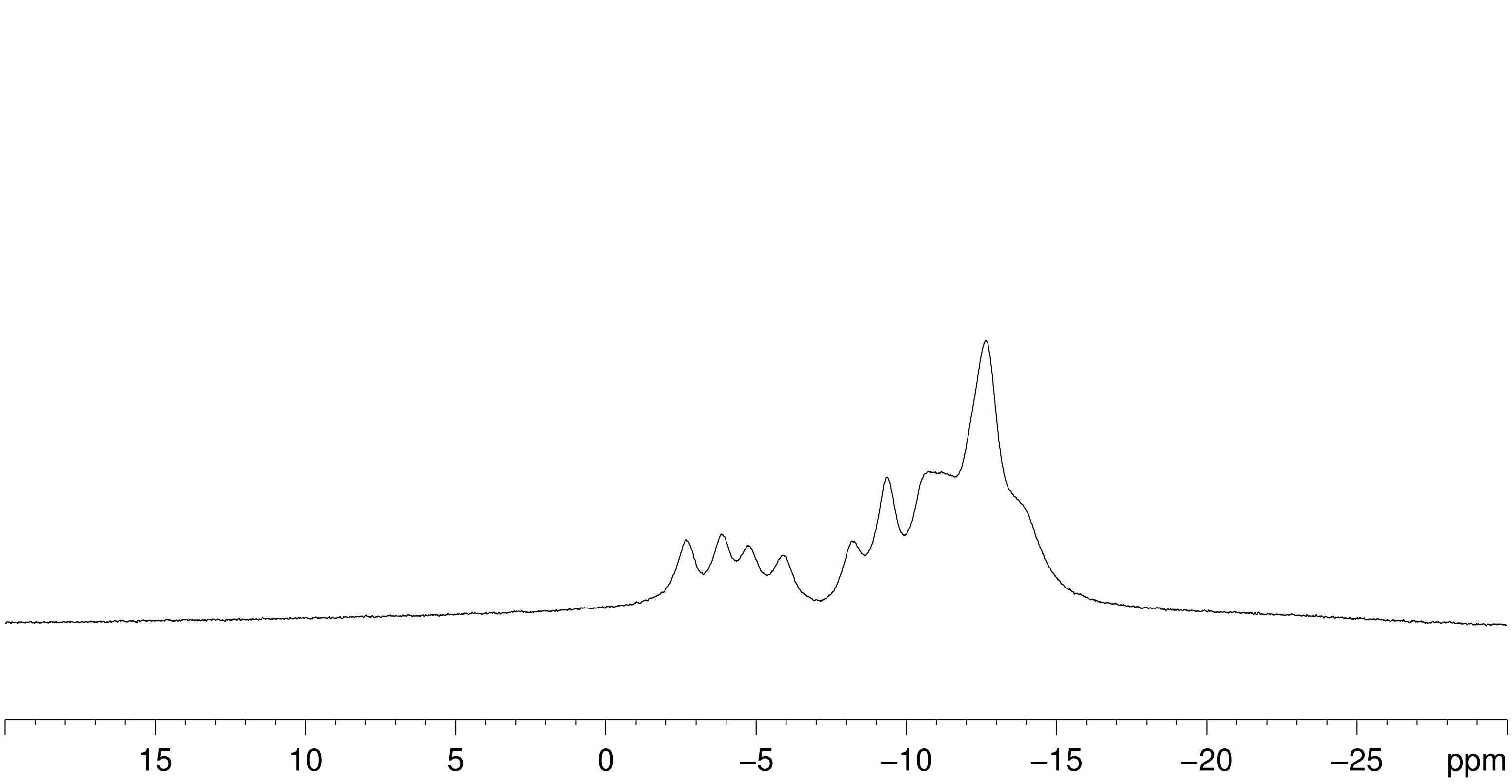

qyj-B-0363-1-CD2Cl2

NAME	qyj-B-0363-1-CD2Cl2(C)
EXPNO	1
PROCNO	1
Date_	20141108
Time	15.36
INSTRUM	spect
PROBHD	5 mm PABBO BB-
PULPROG	zg30
TD	65536
SOLVENT	CDCl3
NS	20
DS	0
SWH	25510.203 Hz
FIDRES	0.389255 Hz
AQ	1.2845556 sec
RG	362
DW	19.600 usec
DE	6.50 usec
TE	299.0 K
D1	5.00000000 sec
TD0	1

======== CHANNEL f1 ========

NUC1	11B
P1	7.60 usec
PL1	-3.00 dB
PL1W	55.13059616 W
SFO1	128.3968556 MHz
SI	32768
SF	128.3968865 MHz
WDW	EM
SSB	0
LB	3.00 Hz
GB	0
PC	1.40

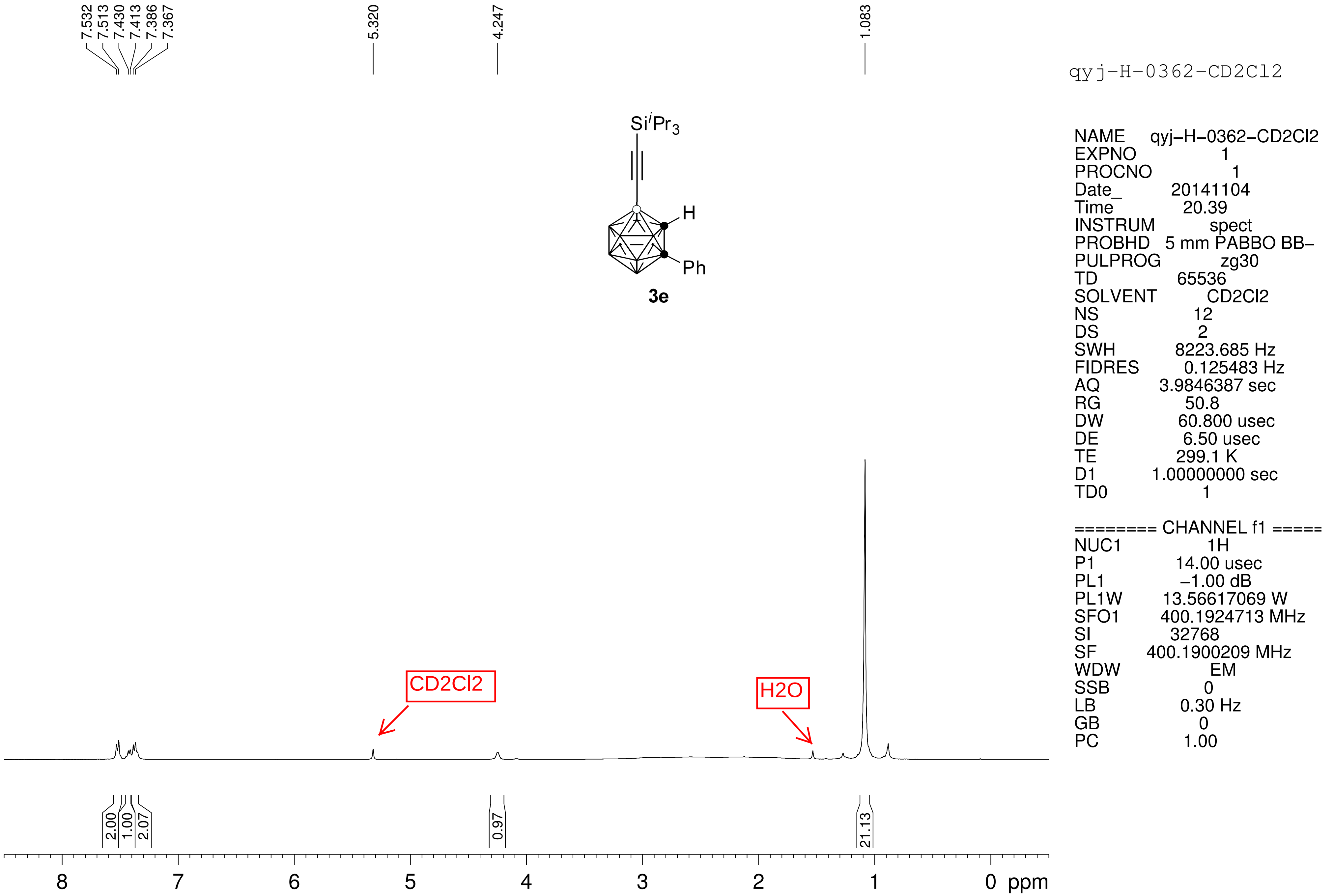
7.532
7.513
7.430
7.413
7.386
7.367
5.320
4.247
1.083
qyj-H-0362-CD2Cl2
Si^{i}Pr$_3$
H
Ph
3e
NAME qyj-H-0362-CD2Cl2
EXPNO 1
PROCNO 1
Date_ 20141104
Time 20.39
INSTRUM spect
PROBHD 5 mm PABBO BB-
PULPROG zg30
TD 65536
SOLVENT CD2Cl2
NS 12
DS 2
SWH 8223.685 Hz
FIDRES 0.125483 Hz
AQ 3.9846387 sec
RG 50.8
DW 60.800 usec
DE 6.50 usec
TE 299.1 K
D1 1.00000000 sec
TD0 1
======== CHANNEL f1 =====
NUC1 1H
P1 14.00 usec
PL1 -1.00 dB
PL1W 13.56617069 W
SFO1 400.1924713 MHz
SI 32768
SF 400.1900209 MHz
WDW EM
SSB 0
LB 0.30 Hz
GB 0
PC 1.00
CD2Cl2
H2O
2.00
1.00
2.07
0.97
21.13
8
7
6
5
4
3
2
1
0 ppm

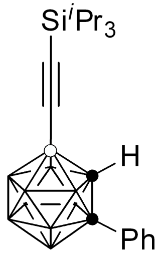

3e

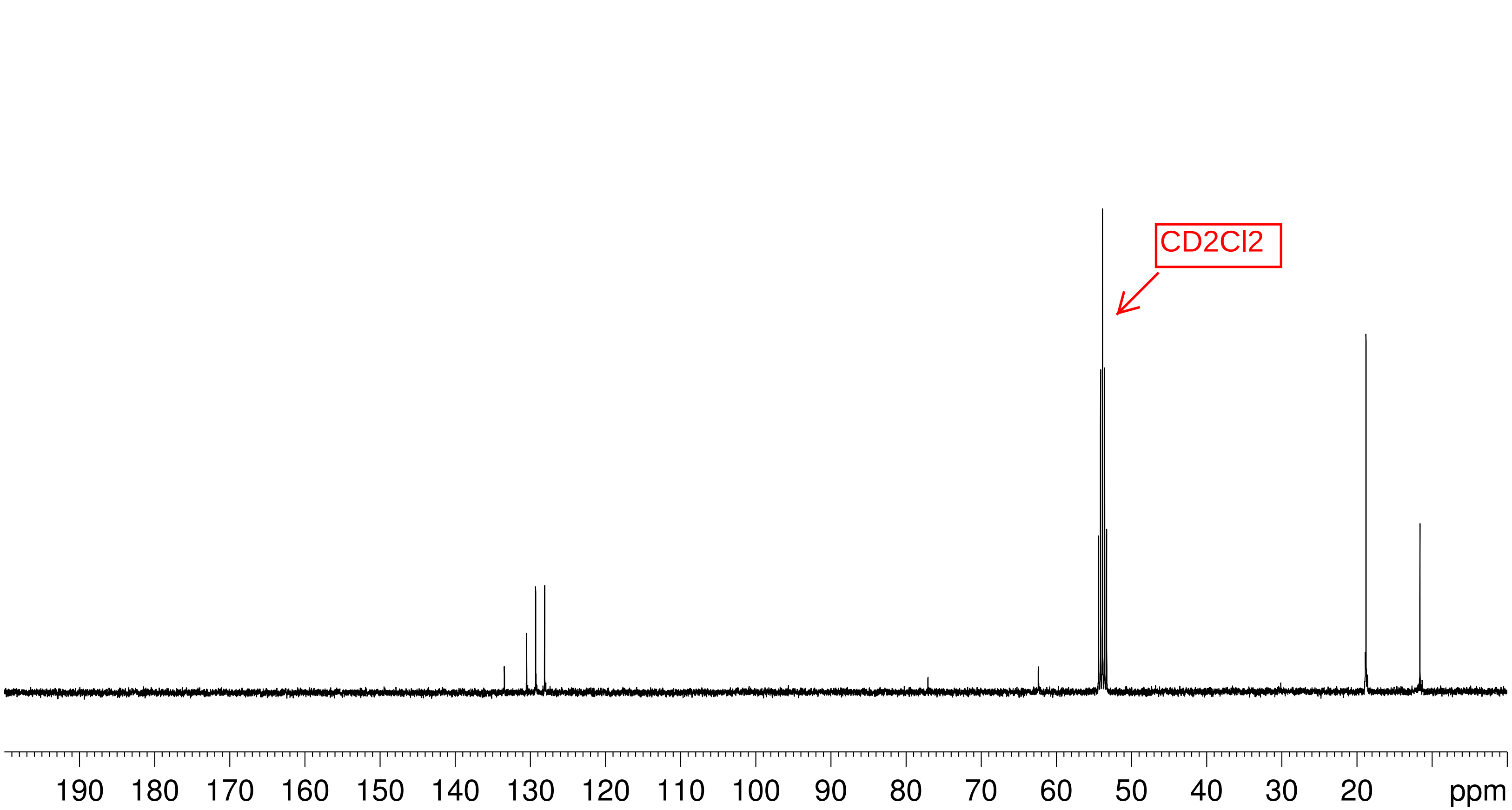

qyj-C-0362-CD2Cl2

NAME	qyj-C-0362-CD2Cl2	
EXPNO	1	
PROCNO	1	
Date_	20141104	
Time	20.43	
INSTRUM	spect	
PROBHD	5 mm PABBO BB-	
PULPROG	zgpg30	
TD	65536	
SOLVENT	CD2Cl2	
NS	100	
DS	4	
SWH	24038.461	Hz
FIDRES	0.366798	Hz
AQ	1.3631988	sec
RG	181	
DW	20.800	usec
DE	6.50	usec
TE	299.3	K
D1	2.00000000	sec
D11	0.03000000	sec
TD0	1	
======== CHANNEL f1 ========		
NUC1	13C	
P1	9.90	usec
PL1	-2.00	dB
PL1W	55.33689499	W
SFO1	100.6379183	MHz
======== CHANNEL f2 ========		
CPDPRG2	waltz16	
NUC2	1H	
PCPD2	90.00	usec
PL2	-1.00	dB
PL12	15.16	dB
PL13	18.62	dB
PL2W	13.56617069	W
PL12W	0.32844096	W
PL13W	0.14806664	W
SFO2	400.1916008	MHz
SI	32768	
SF	100.6278132	MHz
WDW	EM	
SSB	0	
LB	1.00	Hz
GB	0	
PC	1.40	

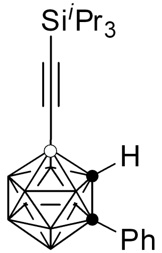

3e

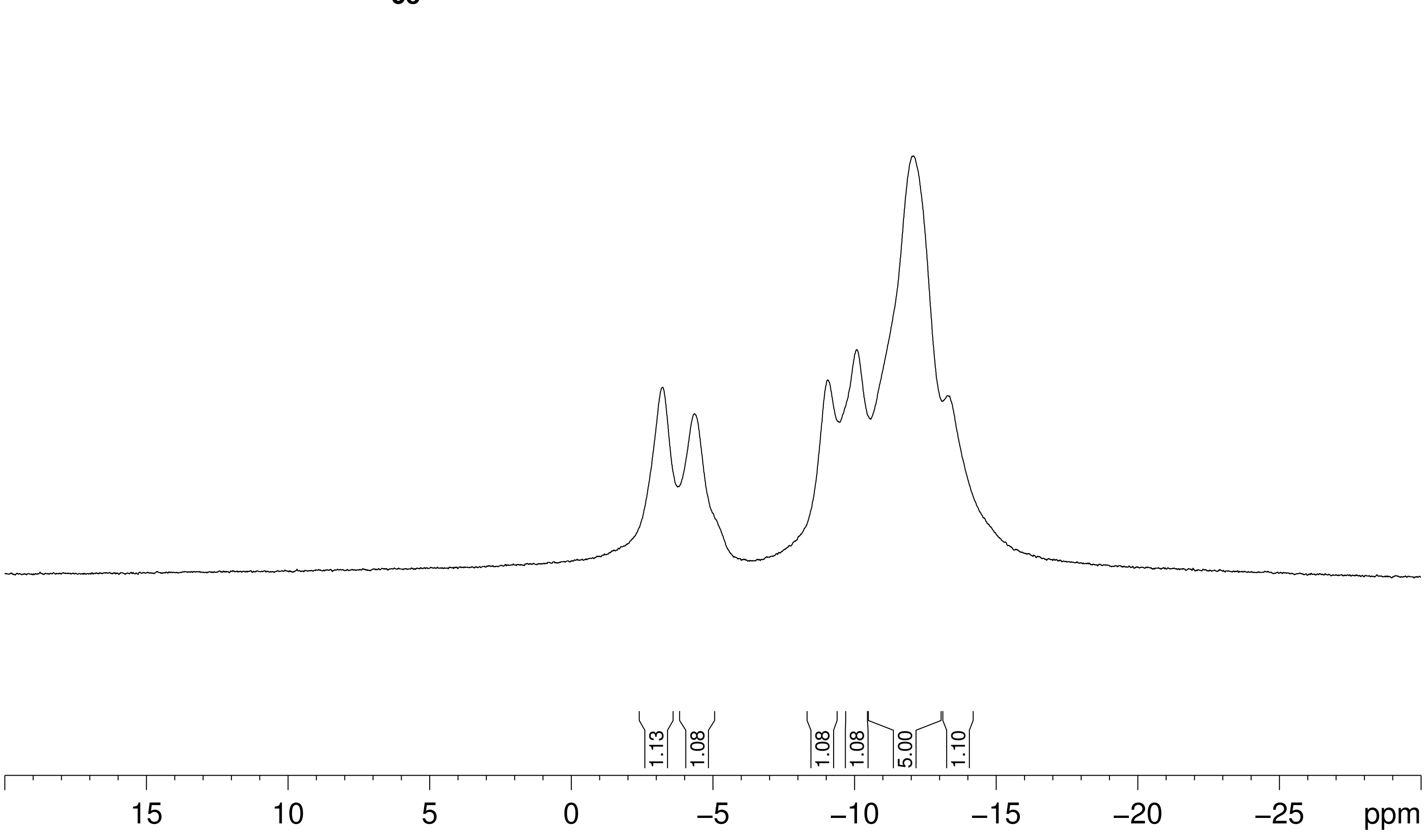

qyj-B-0362-CD2Cl2

NAME	qyj-B-0362-CD2Cl2
EXPNO	1
PROCNO	1
Date_	20141104
Time	20.27
INSTRUM	spect
PROBHD	5 mm PABBO BB-
PULPROG	zgdc
TD	65536
SOLVENT	CDCl3
NS	12
DS	0
SWH	25510.203 Hz
FIDRES	0.389255 Hz
AQ	1.2845556 sec
RG	181
DW	19.600 usec
DE	6.50 usec
TE	299.4 K
D1	5.00000000 sec
D11	0.03000000 sec
TD0	1

======== CHANNEL f1 ========

NUC1	11B
P1	7.60 usec
PL1	-3.00 dB
PL1W	55.13059616 W
SFO1	128.3968556 MHz

======== CHANNEL f2 ========

CPDPRG2	waltz16
NUC2	1H
PCPD2	90.00 usec
PL2	-1.00 dB
PL12	15.16 dB
PL2W	13.56617069 W
PL12W	0.32844096 W
SFO2	400.1916008 MHz
SI	32768
SF	128.3969291 MHz
WDW	EM
SSB	0
LB	3.00 Hz
GB	0
PC	1.40

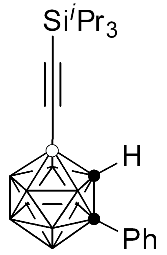

3e

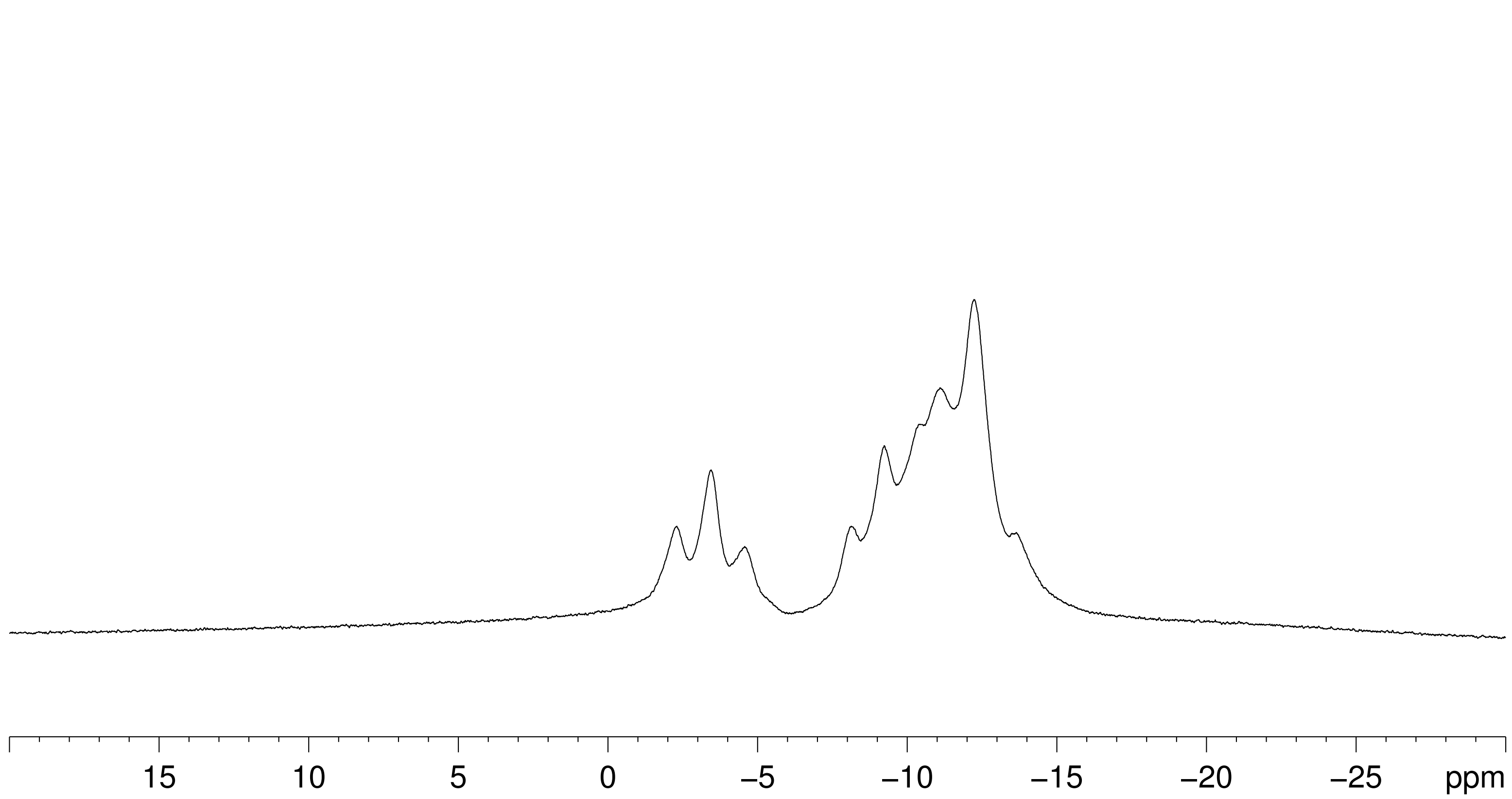

qyj-B-0362-CD2Cl2(C

NAME	qyj-B-0362-CD2Cl2(C)
EXPNO	1
PROCNO	1
Date_	20141104
Time	20.29
INSTRUM	spect
PROBHD	5 mm PABBO BB-
PULPROG	zg30
TD	65536
SOLVENT	CDCl3
NS	20
DS	0
SWH	25510.203 Hz
FIDRES	0.389255 Hz
AQ	1.2845556 sec
RG	362
DW	19.600 usec
DE	6.50 usec
TE	299.0 K
D1	5.00000000 sec
TD0	1

======== CHANNEL f1 ========

NUC1	11B
P1	7.60 usec
PL1	-3.00 dB
PL1W	55.13059616 W
SFO1	128.3968556 MHz
SI	32768
SF	128.3968865 MHz
WDW	EM
SSB	0
LB	3.00 Hz
GB	0
PC	1.40

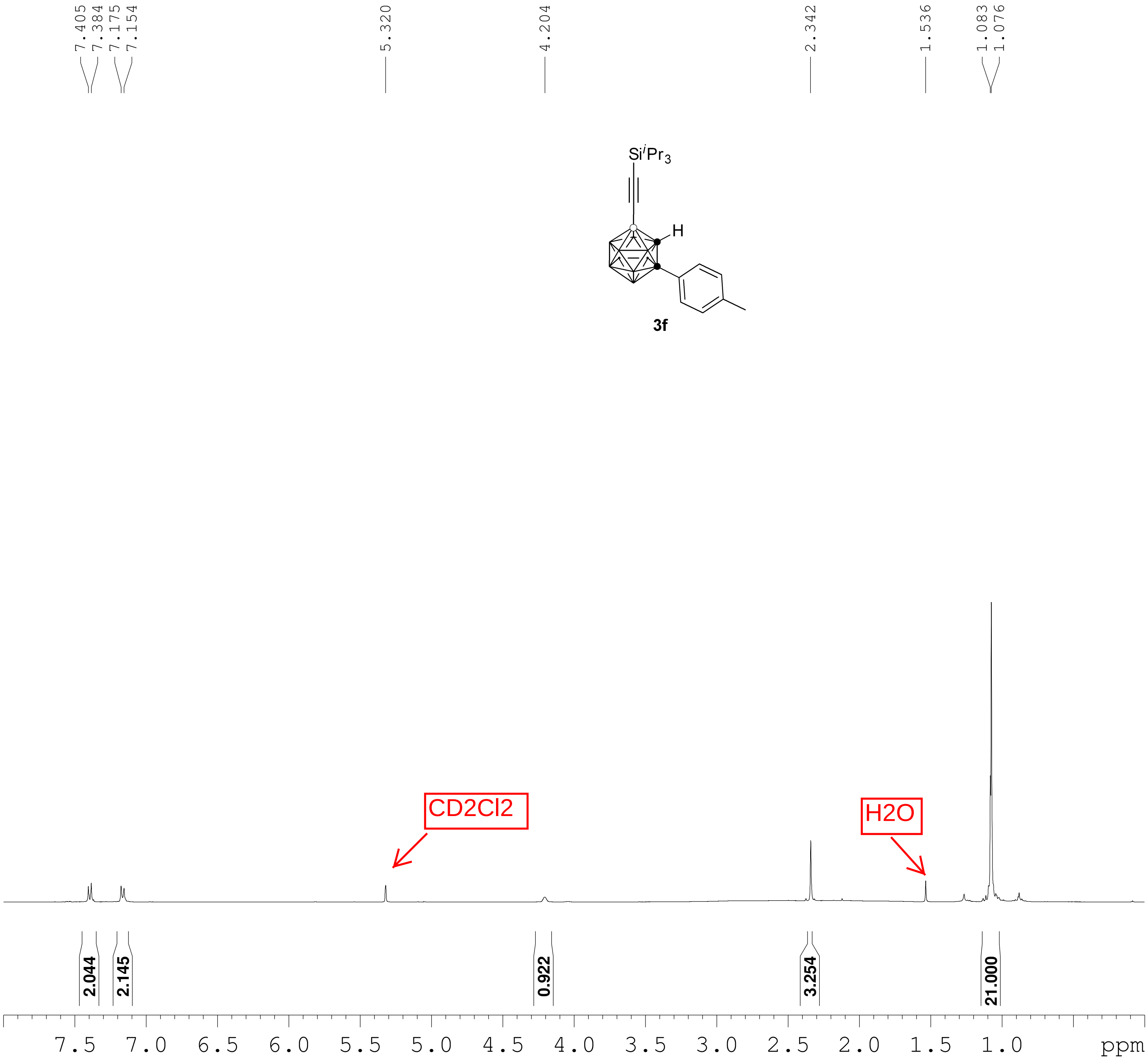

qyj-H-0366-cd2cl2

Bruker Advance III 400

NAME	qyj-H-0366-cd2cl2
EXPNO	1
PROCNO	1
Date_	20141110
Time	16.43
INSTRUM	spect
PROBHD	5 mm PADUL 13C
PULPROG	zg30
TD	65536
SOLVENT	CD2Cl2
NS	12
DS	2
SWH	8223.685 Hz
FIDRES	0.125483 Hz
AQ	3.9846387 sec
RG	203
DW	60.800 usec
DE	6.50 usec
TE	296.9 K
D1	1.00000000 sec
TD0	1

======== CHANNEL f1 ========

NUC1	1H
P1	14.83 usec
PL1	0.00 dB
PL1W	8.31434441 W
SFO1	400.1324710 MHz
SI	32768
SF	400.1300144 MHz
WDW	EM
SSB	0
LB	0.30 Hz
GB	0
PC	1.00

qyj-C-0366-cd2cl2

Bruker Advance III 400

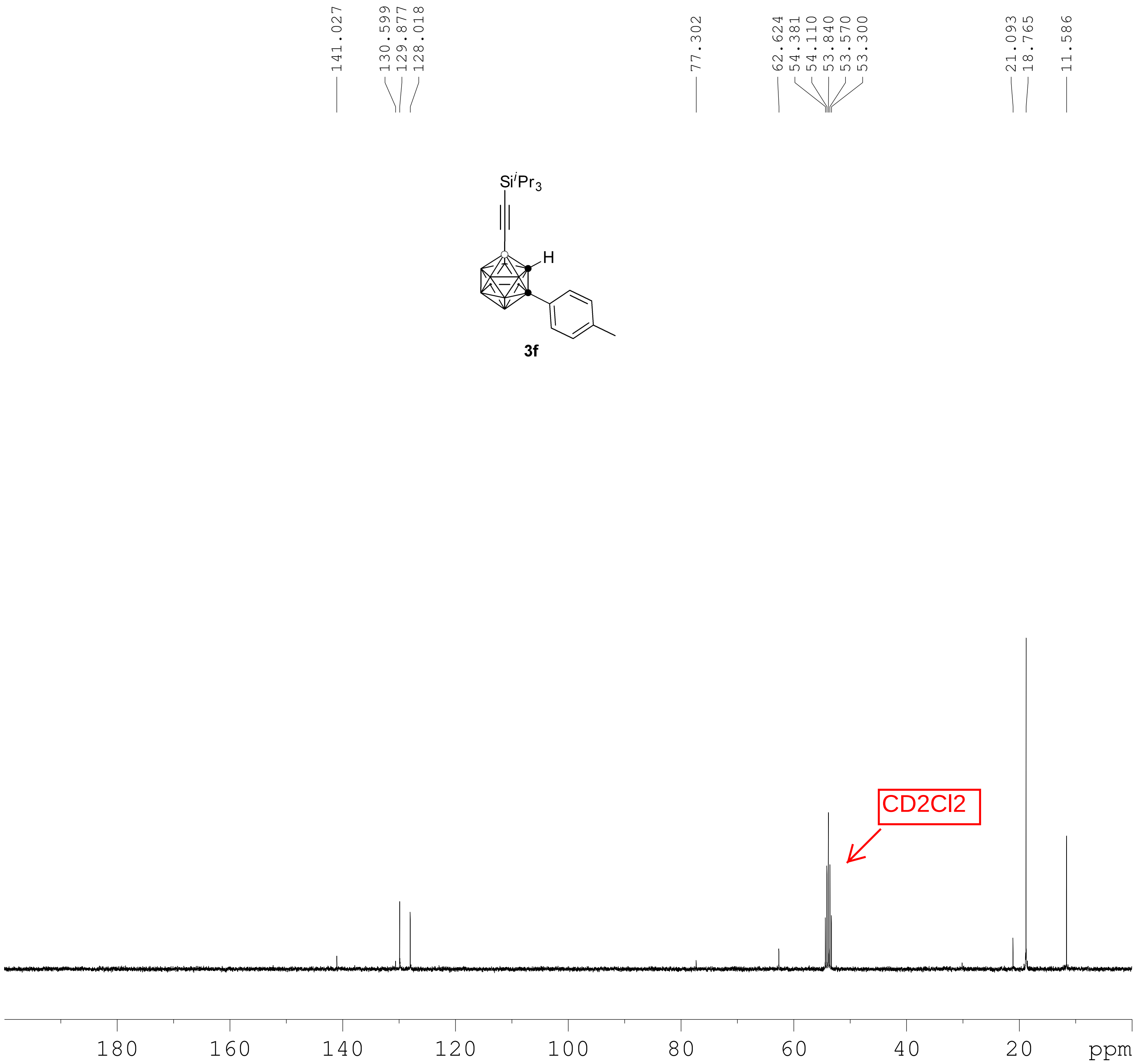

NAME	qyj-C-0366-cd2cl2
EXPNO	1
PROCNO	1
Date_	20141110
Time	16.46
INSTRUM	spect
PROBHD	5 mm PADUL 13C
PULPROG	zgdc
TD	131072
SOLVENT	CD2Cl2
NS	222
DS	0
SWH	29761.904 Hz
FIDRES	0.227065 Hz
AQ	2.2020595 sec
RG	203
DW	16.800 usec
DE	6.50 usec
TE	297.2 K
D1	1.00000000 sec
D11	0.03000000 sec
TD0	1

======== CHANNEL f1 ========

NUC1	13C
P1	9.68 usec
PL1	-0.60 dB
PL1W	41.24164963 W
SFO1	100.6227690 MHz

======== CHANNEL f2 ========

CPDPRG2	waltz16
NUC2	1H
PCPD2	90.00 usec
PL2	0.00 dB
PL12	15.66 dB
PL2W	8.31434441 W
PL12W	0.22585411 W
SFO2	400.1320007 MHz
SI	131072
SF	100.6127255 MHz
WDW	EM
SSB	0
LB	1.00 Hz
GB	0
PC	1.40

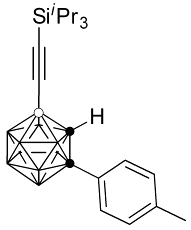

3f

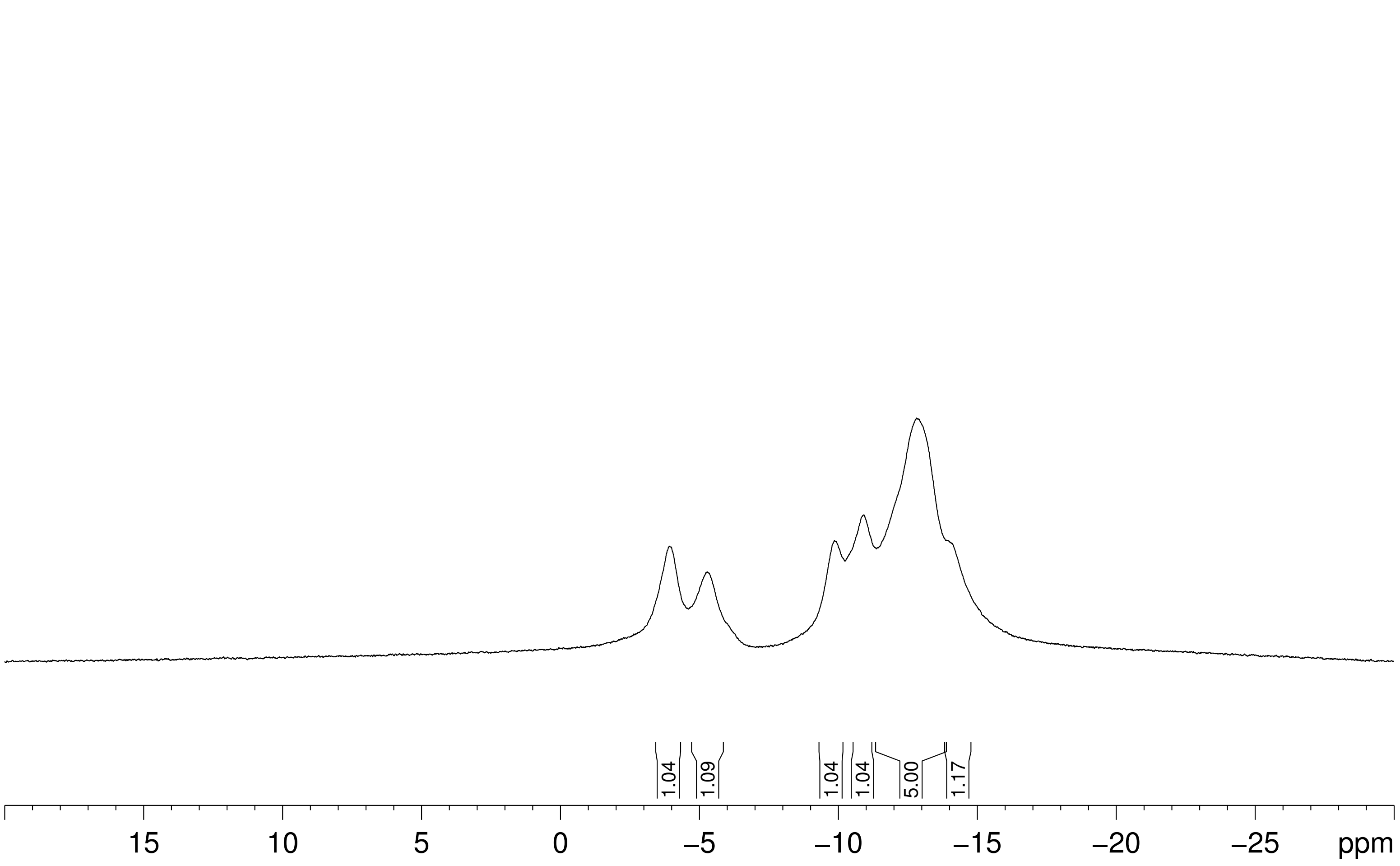

qyj-B-0366-CD2Cl2

```
NAME       qyj-B-0366-CD2Cl2
EXPNO                      1
PROCNO                     1
Date_               20141110
Time                   16.32
INSTRUM                spect
PROBHD      5 mm PABBO BB-
PULPROG                 zgdc
TD                     65536
SOLVENT                 MeOD
NS                        16
DS                         0
SWH                25510.203 Hz
FIDRES              0.389255 Hz
AQ                 1.2845556 sec
RG                       362
DW                    19.600 usec
DE                      6.50 usec
TE                     299.0 K
D1                5.00000000 sec
D11               0.03000000 sec
TD0                        1

======== CHANNEL f1 ========
NUC1                     11B
P1                      7.60 usec
PL1                    -3.00 dB
PL1W             55.13059616 W
SFO1             128.3968556 MHz

======== CHANNEL f2 ========
CPDPRG2              waltz16
NUC2                      1H
PCPD2                  90.00 usec
PL2                    -1.00 dB
PL12                   15.16 dB
PL2W             13.56617069 W
PL12W             0.32844096 W
SFO2             400.1916008 MHz
SI                     32768
SF               128.3969291 MHz
WDW                       EM
SSB                        0
LB                      3.00 Hz
GB                         0
PC                      1.40
```

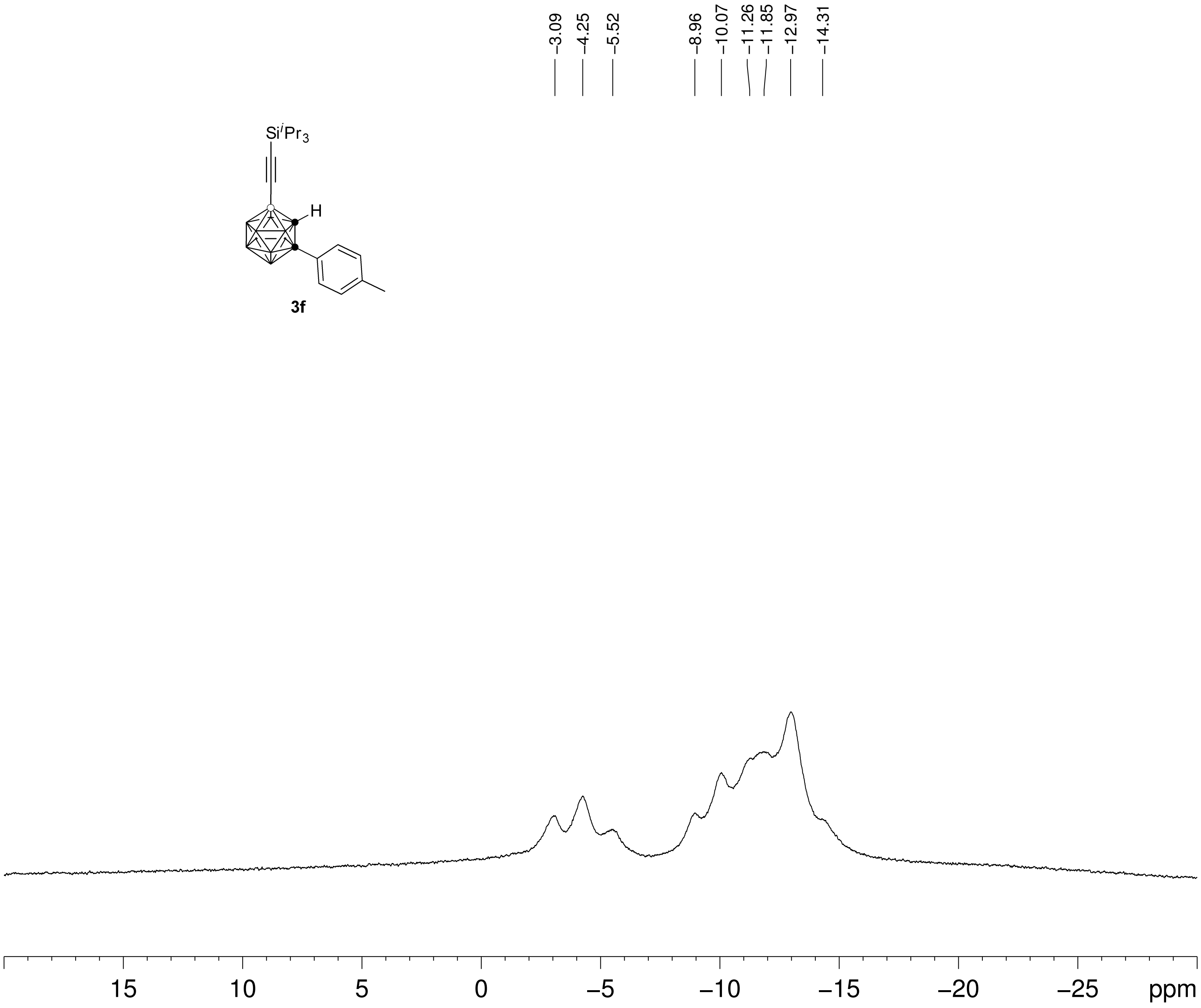

qyj-B-0366-CD2Cl2(C

NAME	qyj-B-0366-CD2Cl2(C)
EXPNO	1
PROCNO	1
Date_	20141110
Time	16.34
INSTRUM	spect
PROBHD	5 mm PABBO BB-
PULPROG	zg30
TD	65536
SOLVENT	MeOD
NS	24
DS	0
SWH	25510.203 Hz
FIDRES	0.389255 Hz
AQ	1.2845556 sec
RG	645
DW	19.600 usec
DE	6.50 usec
TE	298.5 K
D1	5.00000000 sec
TD0	1

======== CHANNEL f1 ========

NUC1	11B
P1	7.60 usec
PL1	-3.00 dB
PL1W	55.13059616 W
SFO1	128.3968556 MHz
SI	32768
SF	128.3968865 MHz
WDW	EM
SSB	0
LB	3.00 Hz
GB	0
PC	1.40

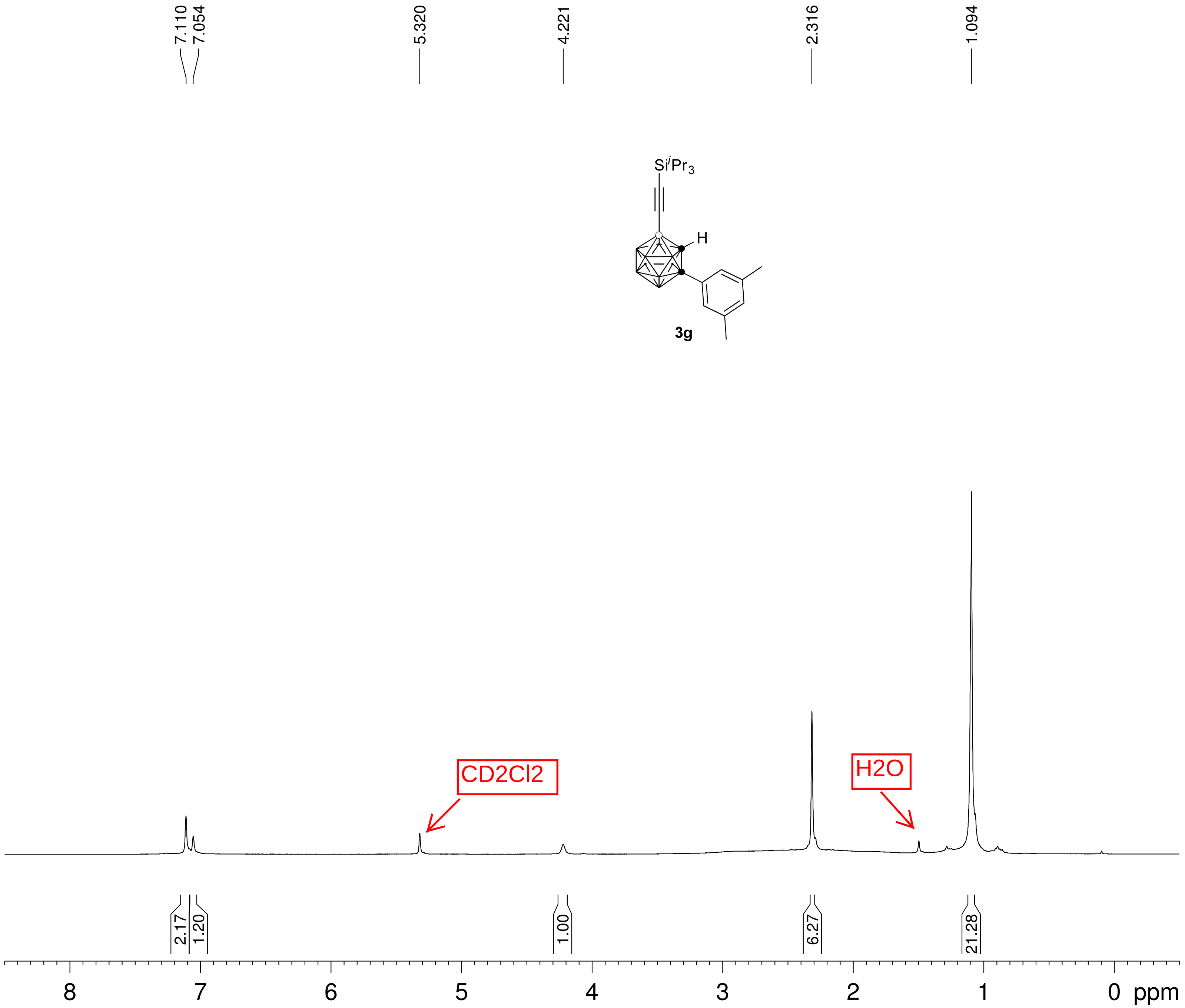

qyj-H-0373-CD2CL2

NAME	qyj–H–0373–CD2CL2
EXPNO	1
PROCNO	1
Date_	20141220
Time	15.11
INSTRUM	spect
PROBHD	5 mm PABBO BB–
PULPROG	zg30
TD	65536
SOLVENT	CD2Cl2
NS	12
DS	2
SWH	8223.685 Hz
FIDRES	0.125483 Hz
AQ	3.9846387 sec
RG	80.6
DW	60.800 usec
DE	6.50 usec
TE	313.1 K
D1	1.00000000 sec
TD0	1

======== CHANNEL f1 =====

NUC1	1H
P1	14.00 usec
PL1	–1.00 dB
PL1W	13.56617069 W
SFO1	400.1924713 MHz
SI	32768
SF	400.1900203 MHz
WDW	EM
SSB	0
LB	0.30 Hz
GB	0
PC	1.00

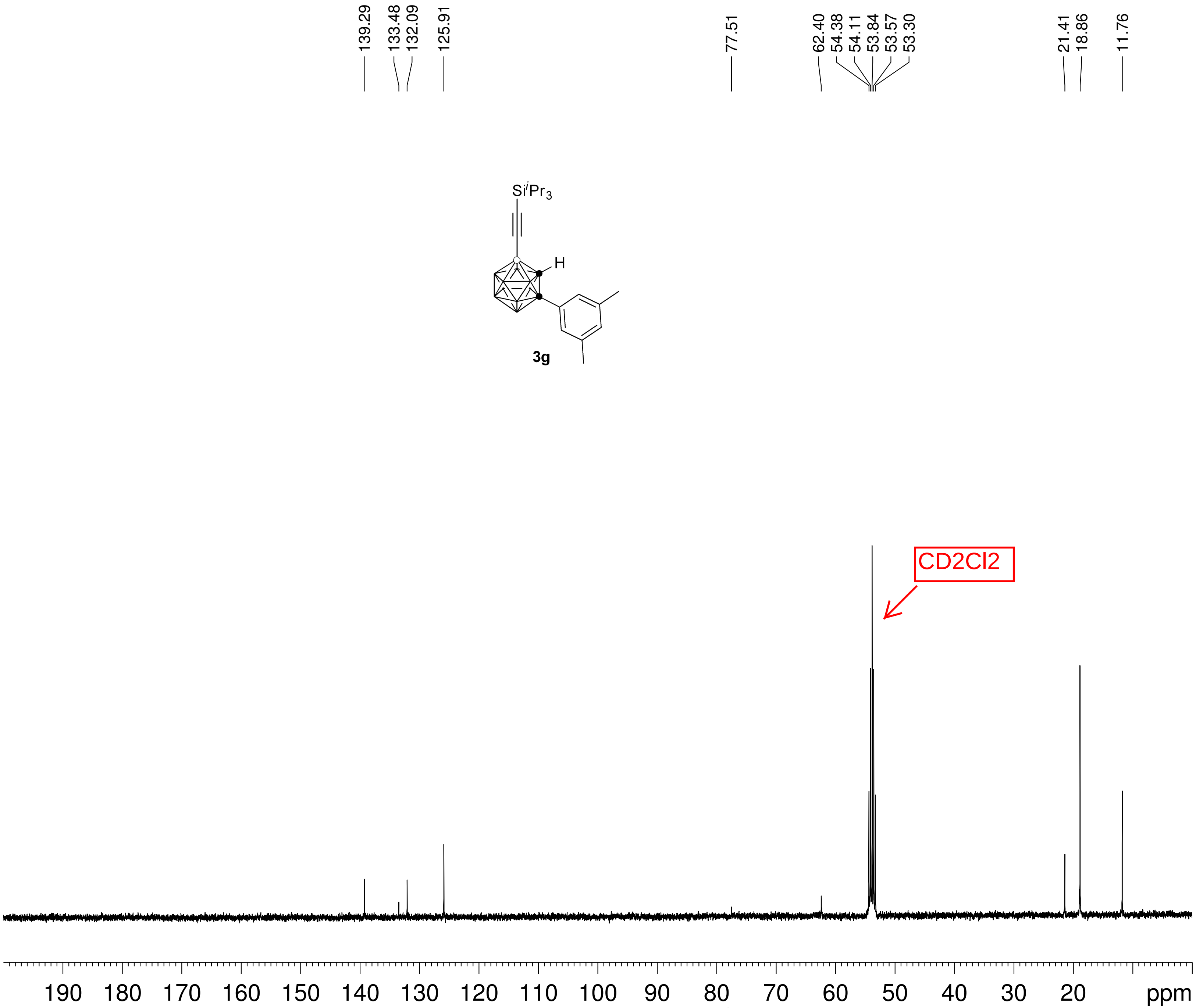

qyj-C-0373-CD2CL2

NAME	qyj-C-0373-CD2CL2
EXPNO	1
PROCNO	1
Date_	20141220
Time	15.14
INSTRUM	spect
PROBHD	5 mm PABBO BB-
PULPROG	zgpg30
TD	65536
SOLVENT	CD2Cl2
NS	320
DS	4
SWH	24038.461 Hz
FIDRES	0.366798 Hz
AQ	1.3631988 sec
RG	181
DW	20.800 usec
DE	6.50 usec
TE	313.3 K
D1	2.00000000 sec
D11	0.03000000 sec
TD0	1

======== CHANNEL f1 ========

NUC1	13C
P1	9.90 usec
PL1	-2.00 dB
PL1W	55.33689499 W
SFO1	100.6379183 MHz

======== CHANNEL f2 ========

CPDPRG2	waltz16
NUC2	1H
PCPD2	90.00 usec
PL2	-1.00 dB
PL12	15.16 dB
PL13	18.62 dB
PL2W	13.56617069 W
PL12W	0.32844096 W
PL13W	0.14806664 W
SFO2	400.1916008 MHz
SI	32768
SF	100.6278015 MHz
WDW	EM
SSB	0
LB	1.00 Hz
GB	0
PC	1.40

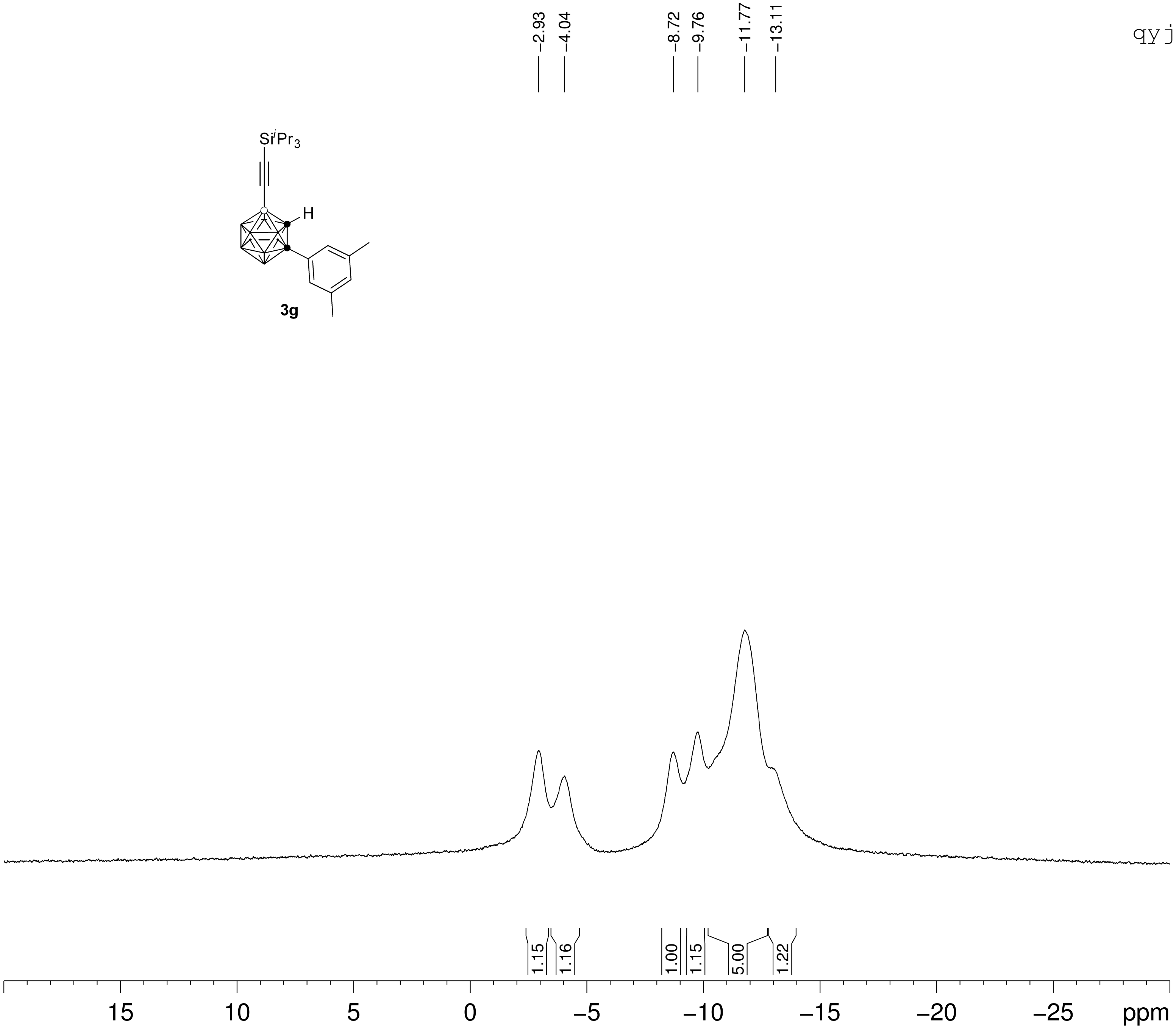

qyj-B-0373-CD2CL2

NAME	qyj-B-0373-CD2CL2
EXPNO	1
PROCNO	1
Date_	20141220
Time	15.34
INSTRUM	spect
PROBHD	5 mm PABBO BB-
PULPROG	zgdc
TD	65536
SOLVENT	CD2Cl2
NS	8
DS	0
SWH	25510.203 Hz
FIDRES	0.389255 Hz
AQ	1.2845556 sec
RG	406
DW	19.600 usec
DE	6.50 usec
TE	313.4 K
D1	5.00000000 sec
D11	0.03000000 sec
TD0	1

======== CHANNEL f1 ========

NUC1	11B
P1	7.60 usec
PL1	-3.00 dB
PL1W	55.13059616 W
SFO1	128.3968556 MHz

======== CHANNEL f2 ========

CPDPRG2	waltz16
NUC2	1H
PCPD2	90.00 usec
PL2	-1.00 dB
PL12	15.16 dB
PL2W	13.56617069 W
PL12W	0.32844096 W
SFO2	400.1916008 MHz
SI	32768
SF	128.3968847 MHz
WDW	EM
SSB	0
LB	3.00 Hz
GB	0
PC	1.40

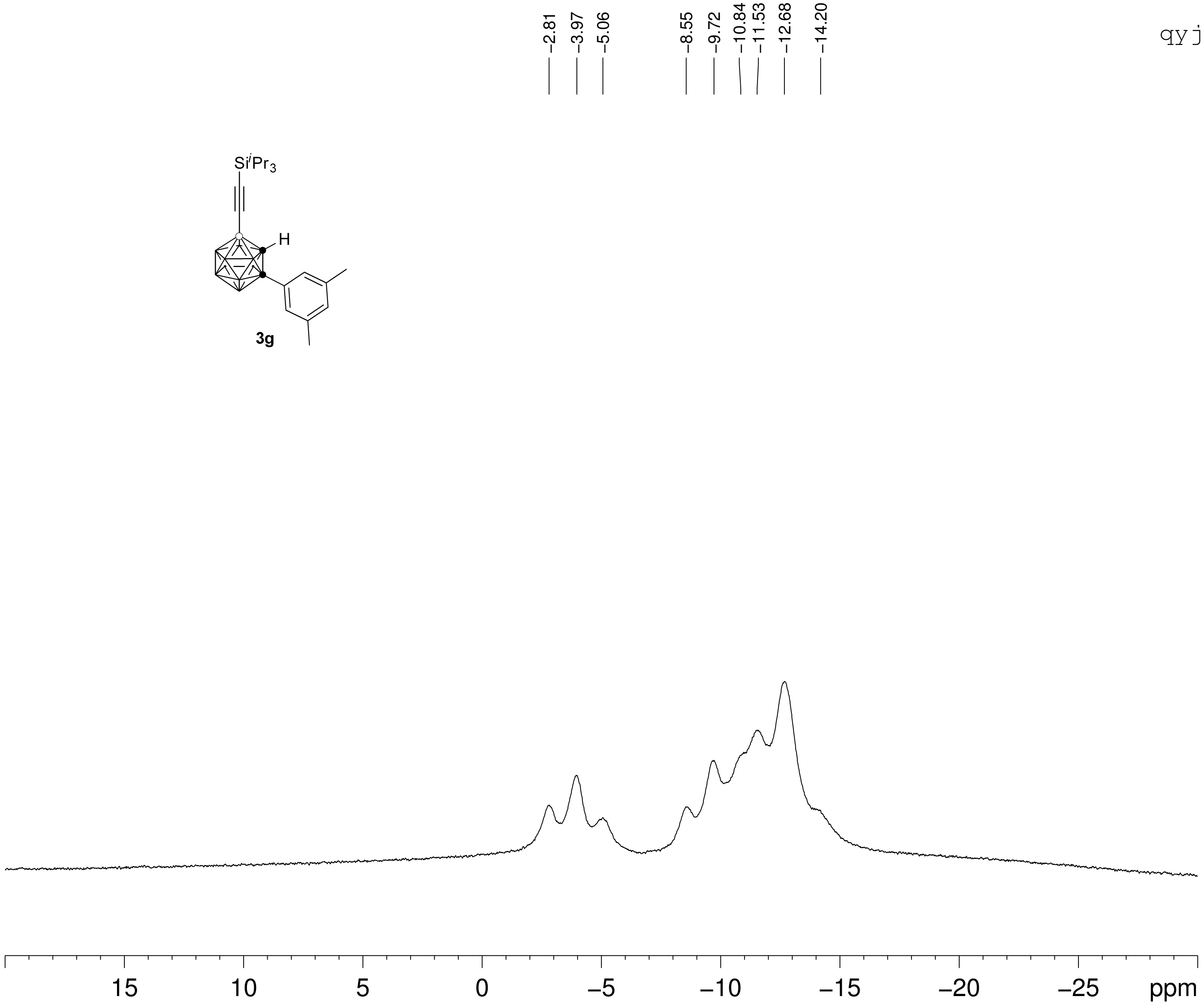

qyj-B-0373-CD2CL2(C)

```
NAME       qyj-B-0373-CD2CL2(C)
EXPNO                     1
PROCNO                    1
Date_              20141220
Time                  15.36
INSTRUM               spect
PROBHD     5 mm PABBO BB-
PULPROG                zg30
TD                    65536
SOLVENT              CD2Cl2
NS                       24
DS                        0
SWH               38461.539 Hz
FIDRES             0.586877 Hz
AQ                0.8520180 sec
RG                      406
DW                   13.000 usec
DE                     6.50 usec
TE                    313.1 K
D1               5.00000000 sec
TD0                       1

======== CHANNEL f1 ========
NUC1                    11B
P1                     7.60 usec
PL1                   -3.00 dB
PL1W            55.13059616 W
SFO1            128.3968556 MHz
SI                    32768
SF              128.3969425 MHz
WDW                      EM
SSB                       0
LB                     3.00 Hz
GB                        0
PC                     1.40
```

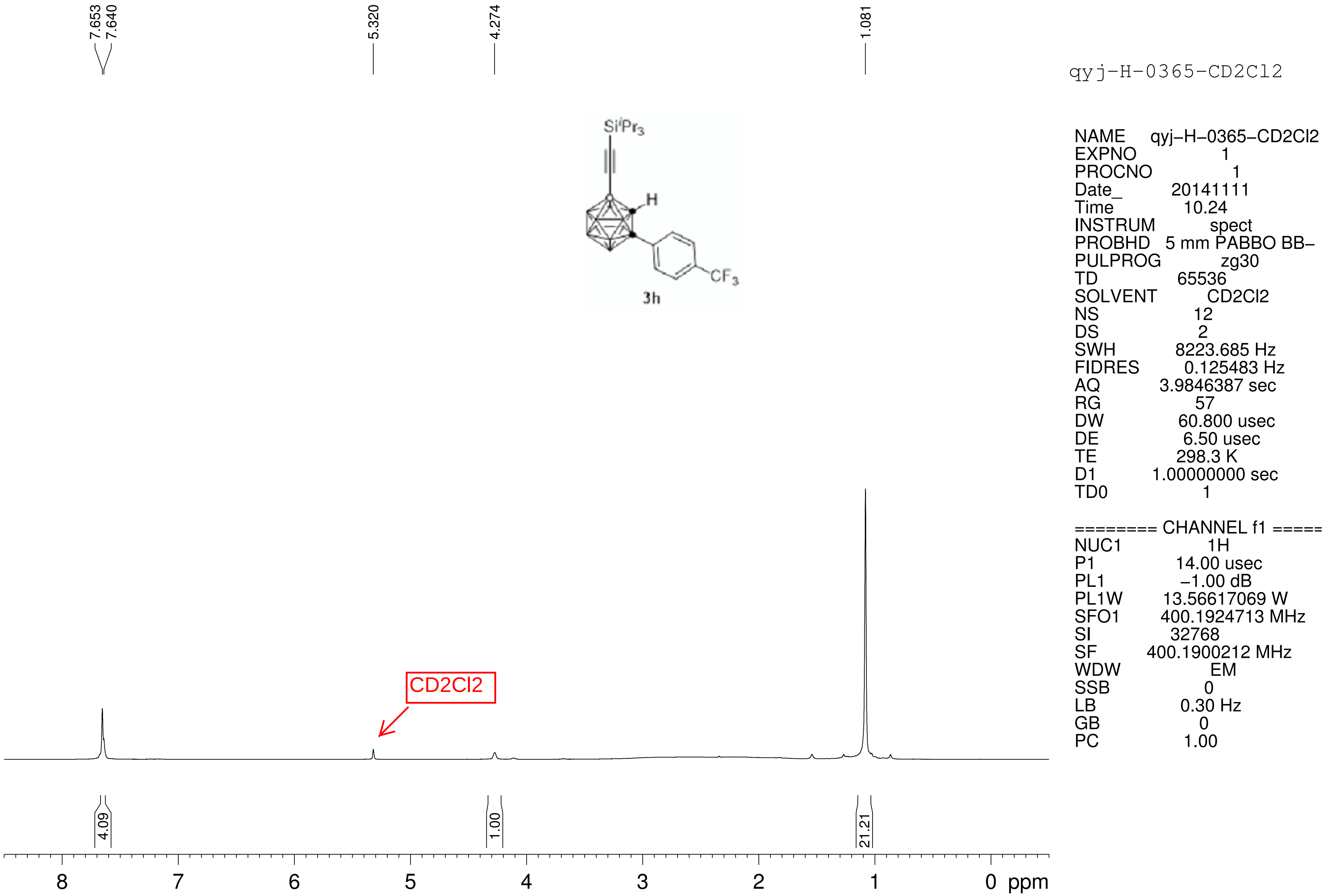

7.653
7.640
5.320
4.274
1.081
qyj-H-0365-CD2Cl2
SiⁱPr3
H
CF3
3h
CD2Cl2
4.09
1.00
21.21
8 7 6 5 4 3 2 1 0 ppm
NAME qyj-H-0365-CD2Cl2
EXPNO 1
PROCNO 1
Date_ 20141111
Time 10.24
INSTRUM spect
PROBHD 5 mm PABBO BB-
PULPROG zg30
TD 65536
SOLVENT CD2Cl2
NS 12
DS 2
SWH 8223.685 Hz
FIDRES 0.125483 Hz
AQ 3.9846387 sec
RG 57
DW 60.800 usec
DE 6.50 usec
TE 298.3 K
D1 1.00000000 sec
TD0 1
======== CHANNEL f1 =====
NUC1 1H
P1 14.00 usec
PL1 -1.00 dB
PL1W 13.56617069 W
SFO1 400.1924713 MHz
SI 32768
SF 400.1900212 MHz
WDW EM
SSB 0
LB 0.30 Hz
GB 0
PC 1.00

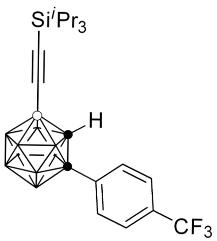

3h

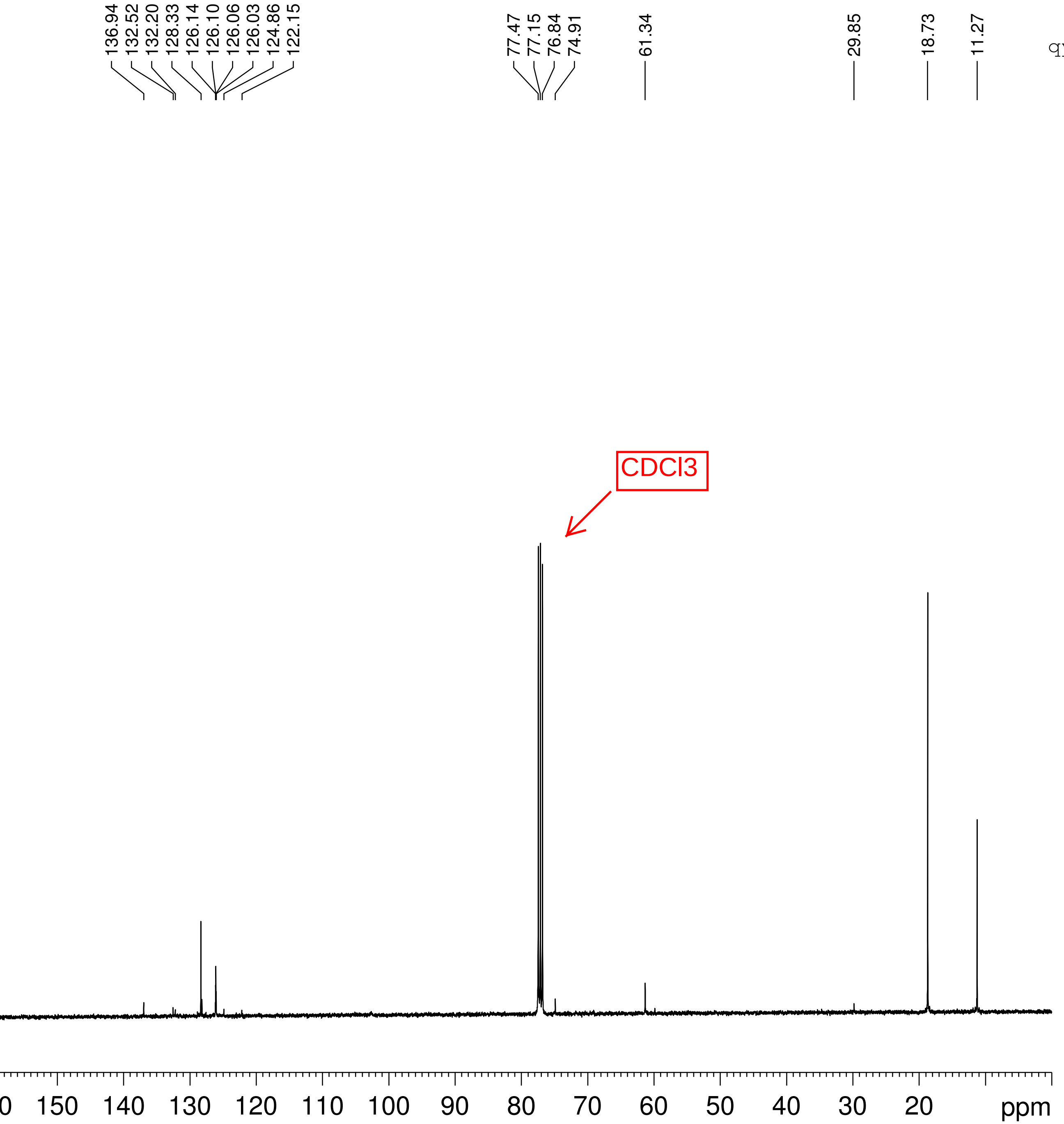

qyj-C-6-cf3-cdcl3

```
Current Data Parameters
NAME      qyj-C-6-cf3-cdcl3
EXPNO                     1
PROCNO                    1

F2 - Acquisition Parameters
Date_              20160401
Time                  17.00 h
INSTRUM               spect
PROBHD     Z108618_0257 (
PULPROG              zgpg30
TD                    65536
SOLVENT               CDCl3
NS                     1261
DS                        4
SWH               29761.904 Hz
FIDRES             0.454131 Hz
AQ                1.1010048 sec
RG                      144
DW                   16.800 usec
DE                     6.50 usec
TE                    294.3 K
D1               2.00000000 sec
D11              0.03000000 sec
TD0                       1
SFO1            100.6479773 MHz
NUC1                    13C
P1                     9.50 usec
PLW1            55.34000015 W
SFO2            400.2316009 MHz
NUC2                     1H
CPDPRG[2            waltz16
PCPD2                 90.00 usec
PLW2            13.56000042 W
PLW12            0.27428001 W
PLW13            0.13796000 W

F2 - Processing parameters
SI                    32768
SF              100.6379006 MHz
WDW                      EM
SSB      0
LB                     1.00 Hz
GB       0
PC                     1.40
```

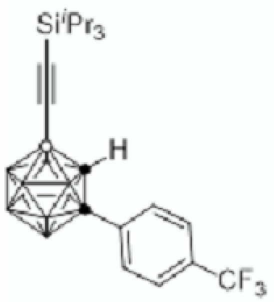

3h

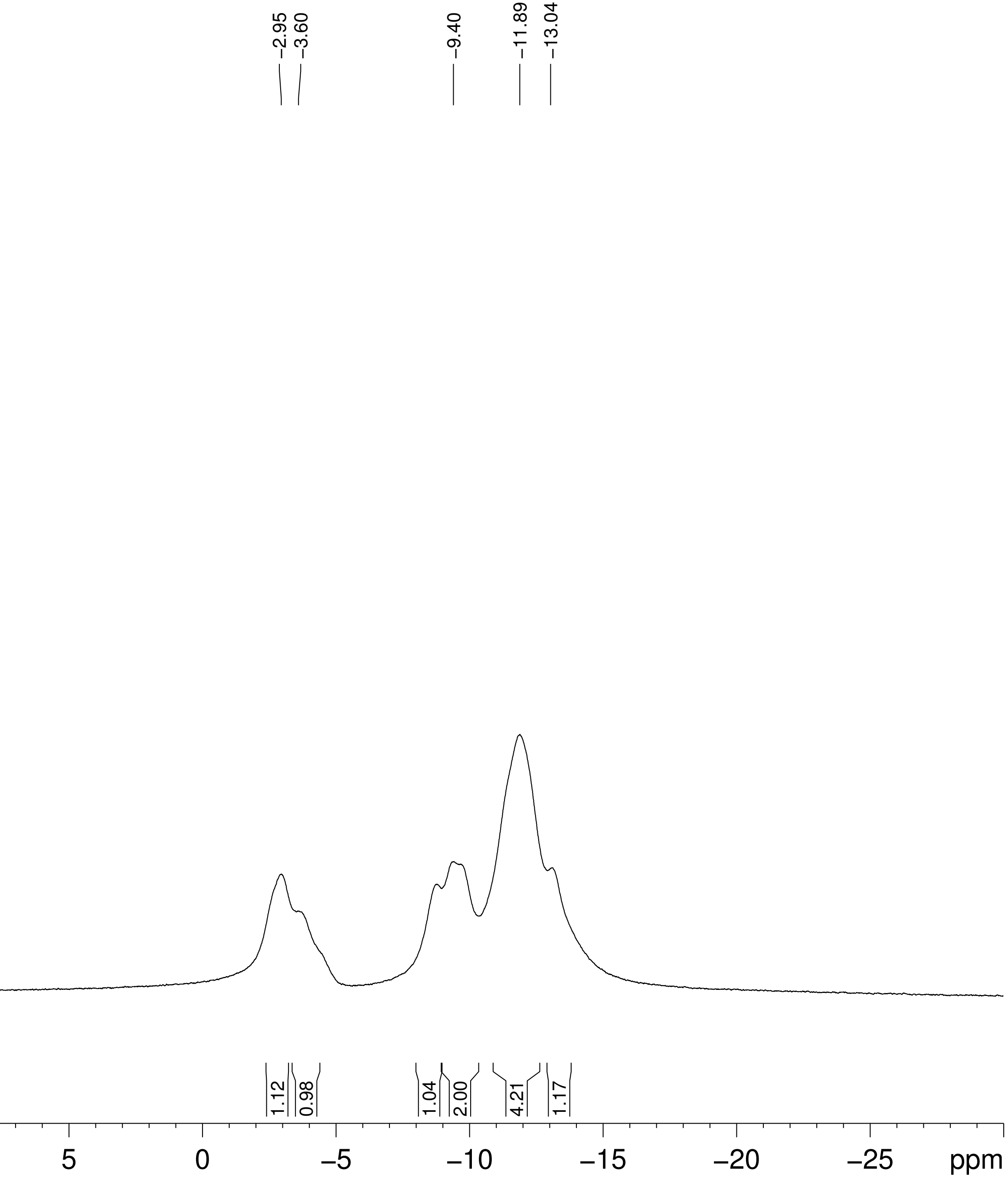

qyj-B-0365-CD2Cl2

NAME	qyj-B-0365-CD2Cl2
EXPNO	1
PROCNO	1
Date_	20141111
Time	10.16
INSTRUM	spect
PROBHD	5 mm PABBO BB-
PULPROG	zgdc
TD	65536
SOLVENT	CDCl3
NS	12
DS	0
SWH	25510.203 Hz
FIDRES	0.389255 Hz
AQ	1.2845556 sec
RG	287
DW	19.600 usec
DE	6.50 usec
TE	298.6 K
D1	5.00000000 sec
D11	0.03000000 sec
TD0	1

======== CHANNEL f1 ========

NUC1	11B
P1	7.60 usec
PL1	-3.00 dB
PL1W	55.13059616 W
SFO1	128.3968556 MHz

======== CHANNEL f2 ========

CPDPRG2	waltz16
NUC2	1H
PCPD2	90.00 usec
PL2	-1.00 dB
PL12	15.16 dB
PL2W	13.56617069 W
PL12W	0.32844096 W
SFO2	400.1916008 MHz
SI	32768
SF	128.3969291 MHz
WDW	EM
SSB	0
LB	3.00 Hz
GB	0
PC	1.40

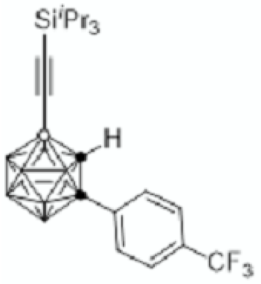

3h

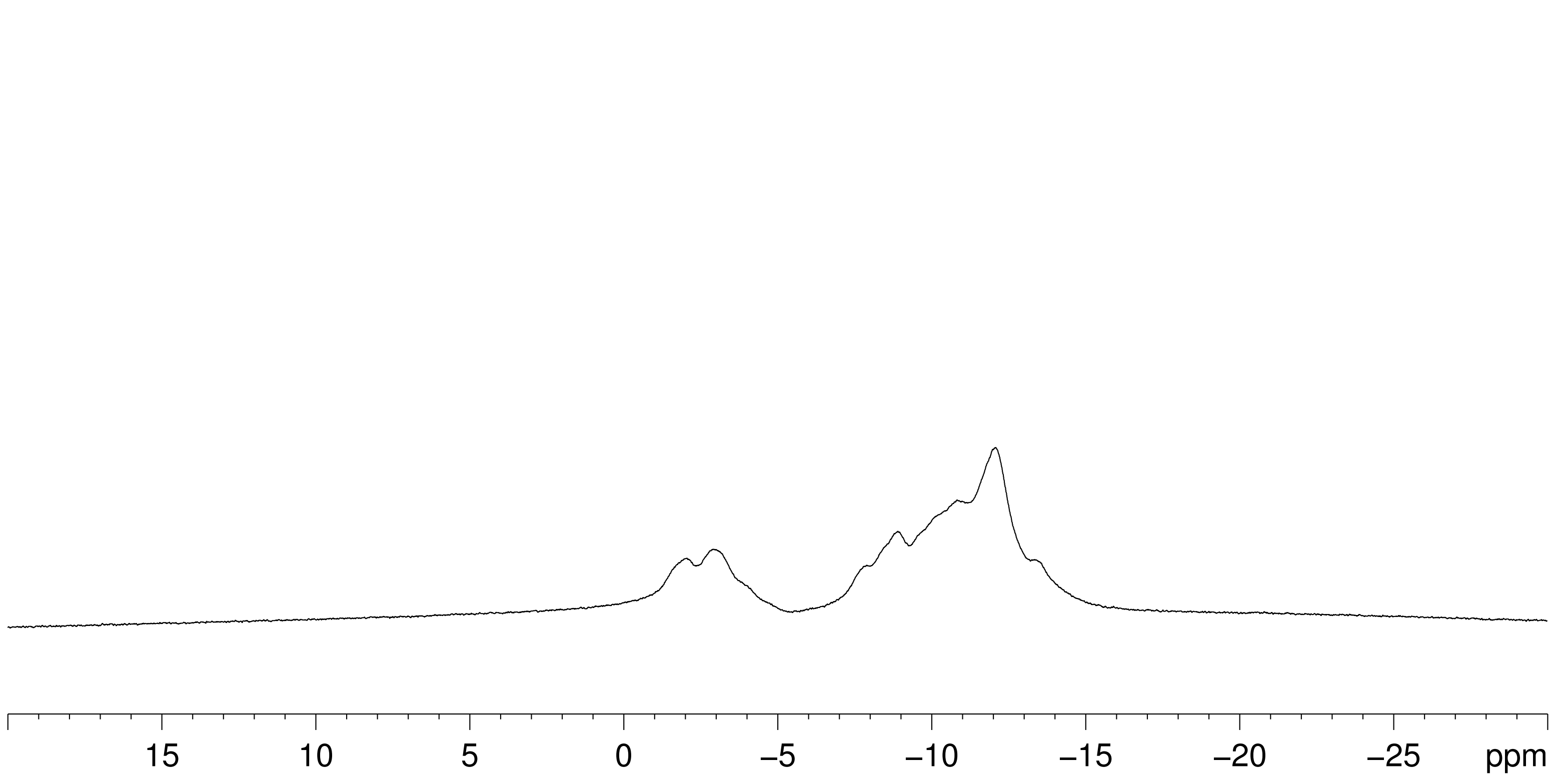

qyj-B-0365-CD2Cl2(C

NAME	qyj-B-0365-CD2Cl2(C)
EXPNO	1
PROCNO	1
Date_	20141111
Time	10.19
INSTRUM	spect
PROBHD	5 mm PABBO BB-
PULPROG	zg30
TD	65536
SOLVENT	CDCl3
NS	16
DS	0
SWH	25510.203 Hz
FIDRES	0.389255 Hz
AQ	1.2845556 sec
RG	406
DW	19.600 usec
DE	6.50 usec
TE	298.1 K
D1	5.00000000 sec
TD0	1

======== CHANNEL f1 ========

NUC1	11B
P1	7.60 usec
PL1	-3.00 dB
PL1W	55.13059616 W
SFO1	128.3968556 MHz
SI	32768
SF	128.3968865 MHz
WDW	EM
SSB	0
LB	3.00 Hz
GB	0
PC	1.40

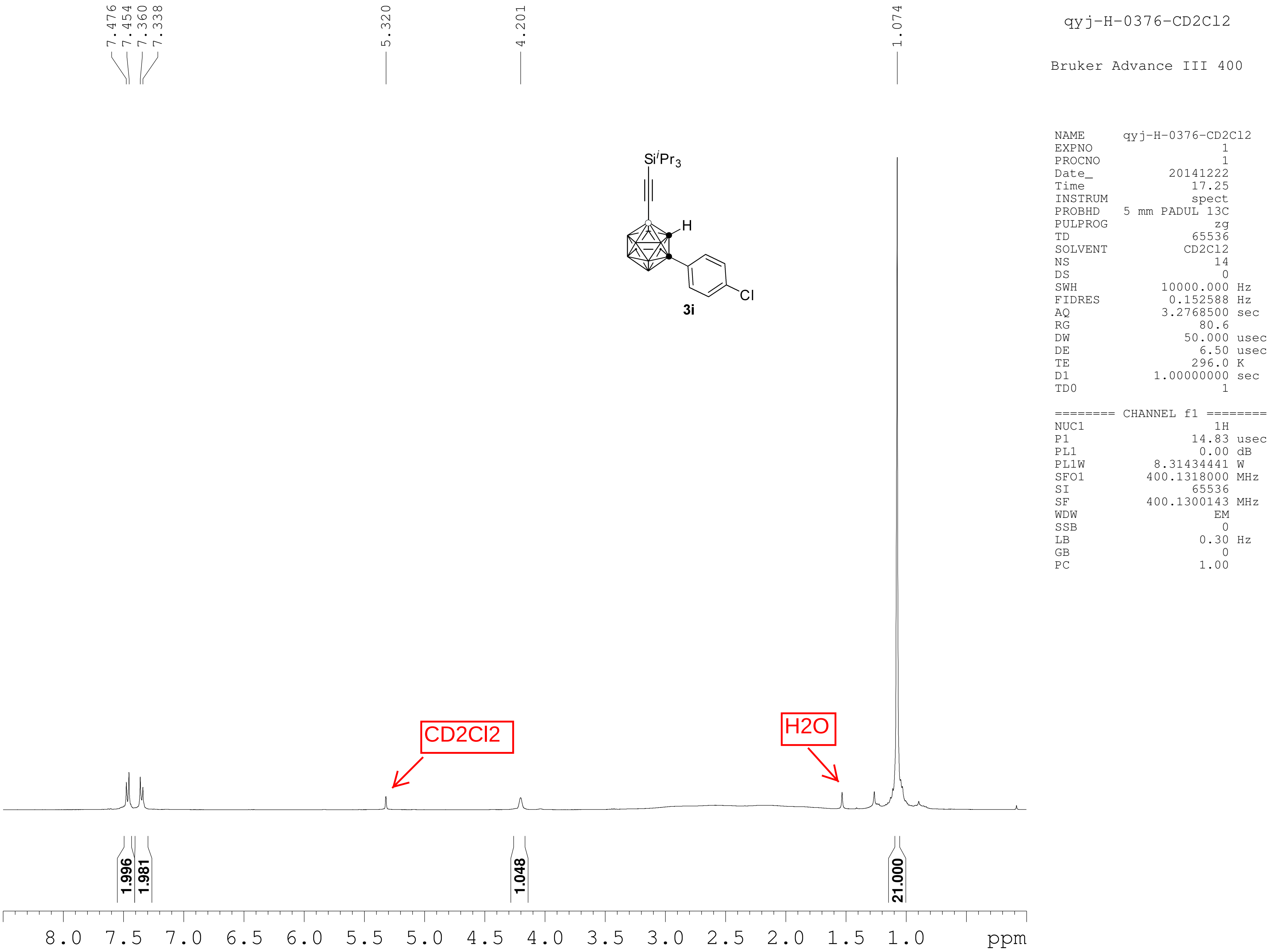

7.476
7.454
7.360
7.338
5.320
4.201
1.074
qyj-H-0376-CD2Cl2
Bruker Advance III 400
NAME qyj-H-0376-CD2Cl2
EXPNO 1
PROCNO 1
Date_ 20141222
Time 17.25
INSTRUM spect
PROBHD 5 mm PADUL 13C
PULPROG zg
TD 65536
SOLVENT CD2Cl2
NS 14
DS 0
SWH 10000.000 Hz
FIDRES 0.152588 Hz
AQ 3.2768500 sec
RG 80.6
DW 50.000 usec
DE 6.50 usec
TE 296.0 K
D1 1.00000000 sec
TD0 1
======== CHANNEL f1 ========
NUC1 1H
P1 14.83 usec
PL1 0.00 dB
PL1W 8.31434441 W
SFO1 400.1318000 MHz
SI 65536
SF 400.1300143 MHz
WDW EM
SSB 0
LB 0.30 Hz
GB 0
PC 1.00
Si^{i}Pr$_3$
H
Cl
3i
CD2Cl2
H2O
1.996
1.981
1.048
21.000
8.0 7.5 7.0 6.5 6.0 5.5 5.0 4.5 4.0 3.5 3.0 2.5 2.0 1.5 1.0 ppm

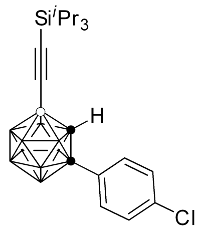

3i

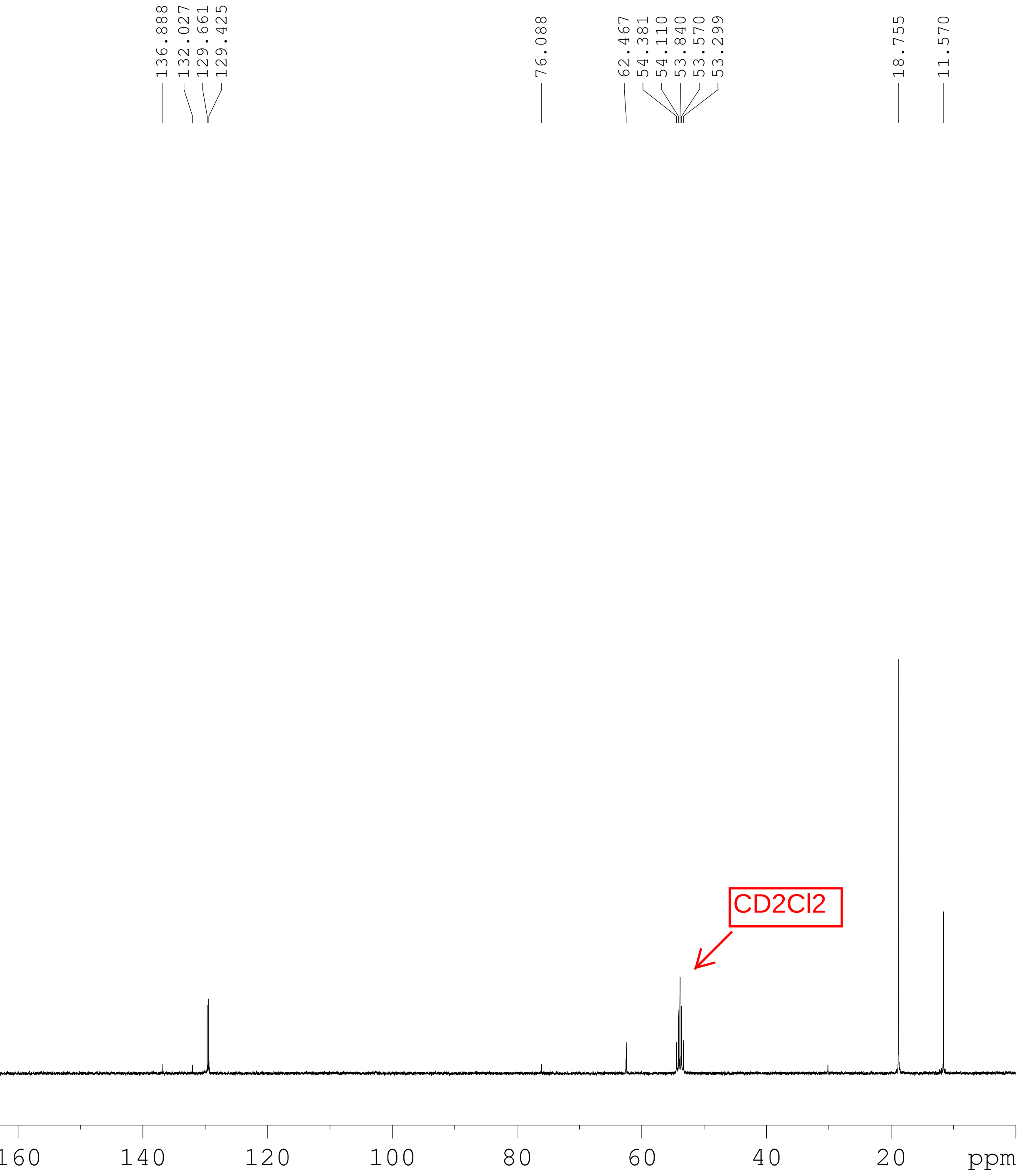

qyj-C-0376-CD2Cl2

Bruker Advance III 400

NAME	qyj-C-0376-CD2Cl2
EXPNO	1
PROCNO	1
Date_	20141222
Time	17.29
INSTRUM	spect
PROBHD	5 mm PADUL 13C
PULPROG	zgdc
TD	131072
SOLVENT	CD2Cl2
NS	1106
DS	0
SWH	29761.904 Hz
FIDRES	0.227065 Hz
AQ	2.2020595 sec
RG	203
DW	16.800 usec
DE	6.50 usec
TE	296.2 K
D1	1.00000000 sec
D11	0.03000000 sec
TD0	1

======== CHANNEL f1 ========

NUC1	13C
P1	9.68 usec
PL1	-0.60 dB
PL1W	41.24164963 W
SFO1	100.6227690 MHz

======== CHANNEL f2 ========

CPDPRG2	waltz16
NUC2	1H
PCPD2	90.00 usec
PL2	0.00 dB
PL12	15.66 dB
PL2W	8.31434441 W
PL12W	0.22585411 W
SFO2	400.1320007 MHz
SI	131072
SF	100.6127258 MHz
WDW	EM
SSB	0
LB	1.00 Hz
GB	0
PC	1.40

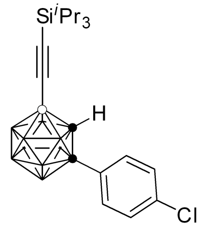

3i

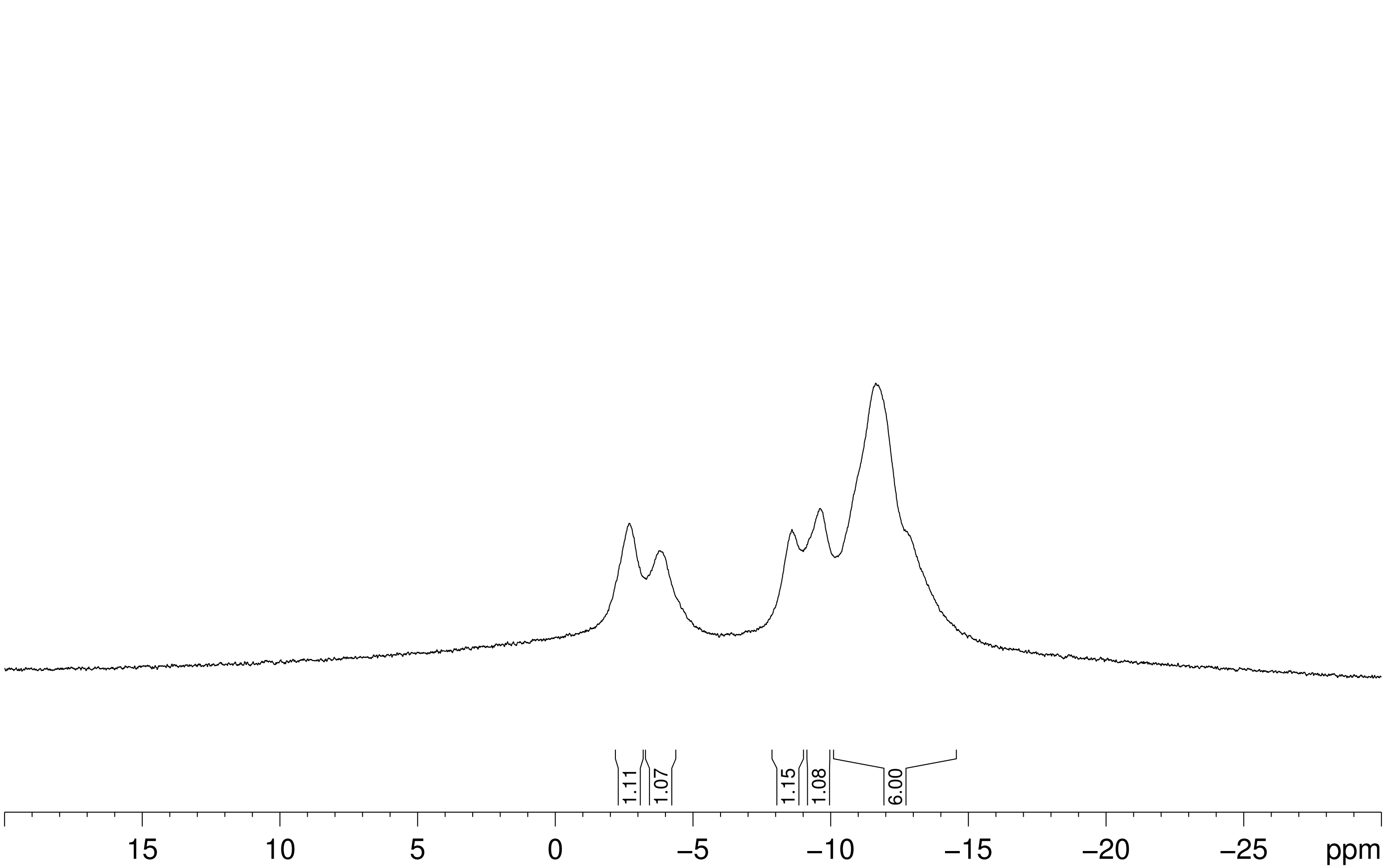

qyj-B-0376-1-CD2CL2

NAME	qyj-B-0376-1-CD2CL2	
EXPNO	1	
PROCNO	1	
Date_	20141222	
Time	18.44	
INSTRUM	spect	
PROBHD	5 mm PABBO BB-	
PULPROG	zgdc	
TD	65536	
SOLVENT	CDCl3	
NS	8	
DS	0	
SWH	25510.203	Hz
FIDRES	0.389255	Hz
AQ	1.2845556	sec
RG	406	
DW	19.600	usec
DE	6.50	usec
TE	298.0	K
D1	5.00000000	sec
D11	0.03000000	sec
TD0	1	

======== CHANNEL f1 ========

NUC1	11B	
P1	7.60	usec
PL1	-3.00	dB
PL1W	55.13059616	W
SFO1	128.3968556	MHz

======== CHANNEL f2 ========

CPDPRG2	waltz16	
NUC2	1H	
PCPD2	90.00	usec
PL2	-1.00	dB
PL12	15.16	dB
PL2W	13.56617069	W
PL12W	0.32844096	W
SFO2	400.1916008	MHz
SI	32768	
SF	128.3968847	MHz
WDW	EM	
SSB	0	
LB	3.00	Hz
GB	0	
PC	1.40	

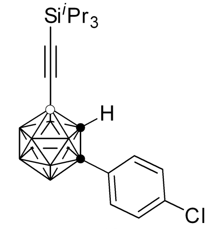

3i

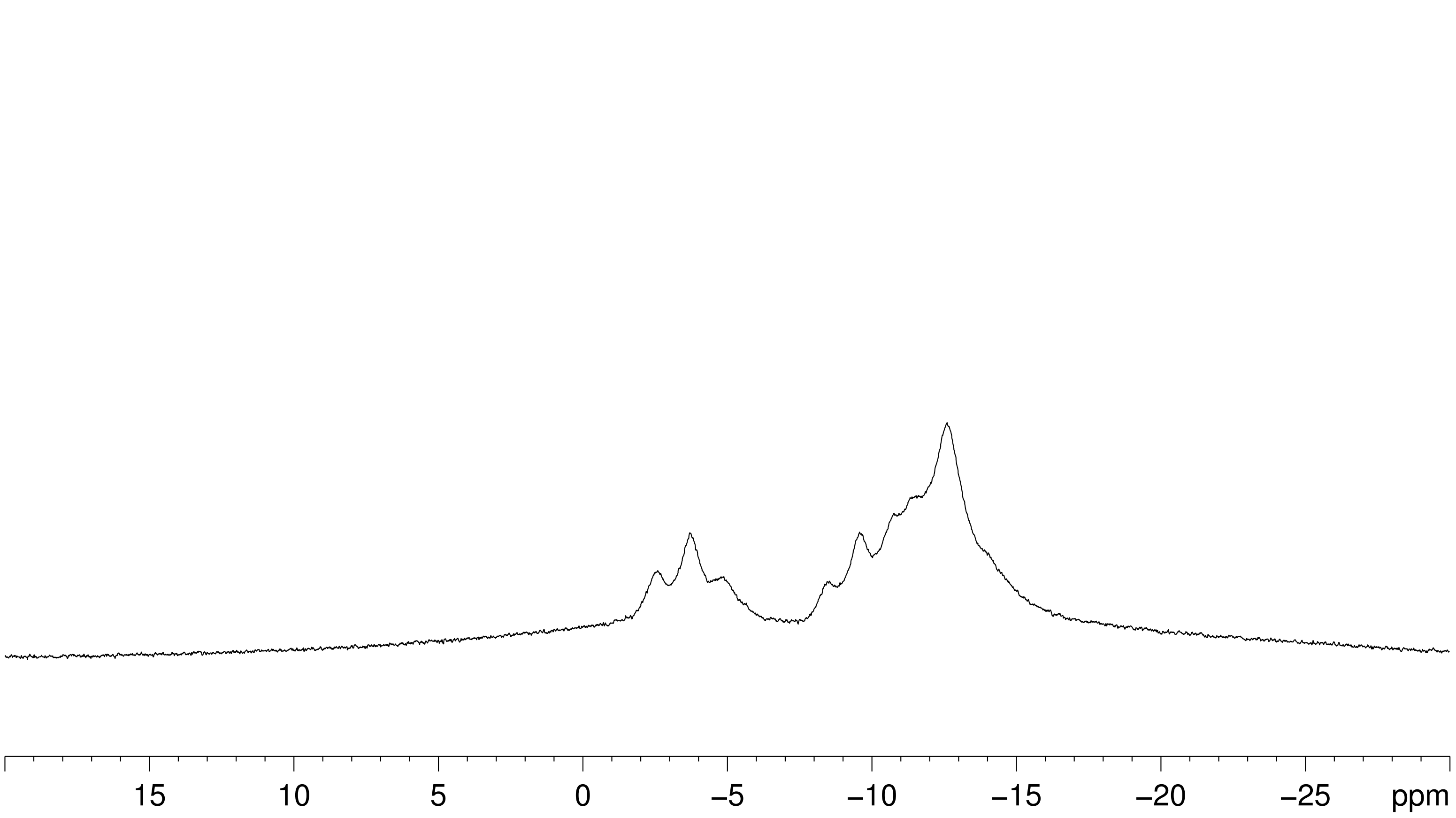

qyj-B-0376-1-CD2CL2(C)

NAME	qyj-B-0376-1-CD2CL2(C)	
EXPNO	1	
PROCNO	1	
Date_	20141222	
Time	18.46	
INSTRUM	spect	
PROBHD	5 mm PABBO BB-	
PULPROG	zg30	
TD	65536	
SOLVENT	CDCl3	
NS	28	
DS	0	
SWH	38461.539	Hz
FIDRES	0.586877	Hz
AQ	0.8520180	sec
RG	812	
DW	13.000	usec
DE	6.50	usec
TE	297.9	K
D1	5.00000000	sec
TD0	1	
========	CHANNEL f1	========
NUC1	11B	
P1	7.60	usec
PL1	-3.00	dB
PL1W	55.13059616	W
SFO1	128.3968556	MHz
SI	32768	
SF	128.3969425	MHz
WDW	EM	
SSB	0	
LB	3.00	Hz
GB	0	
PC	1.40	

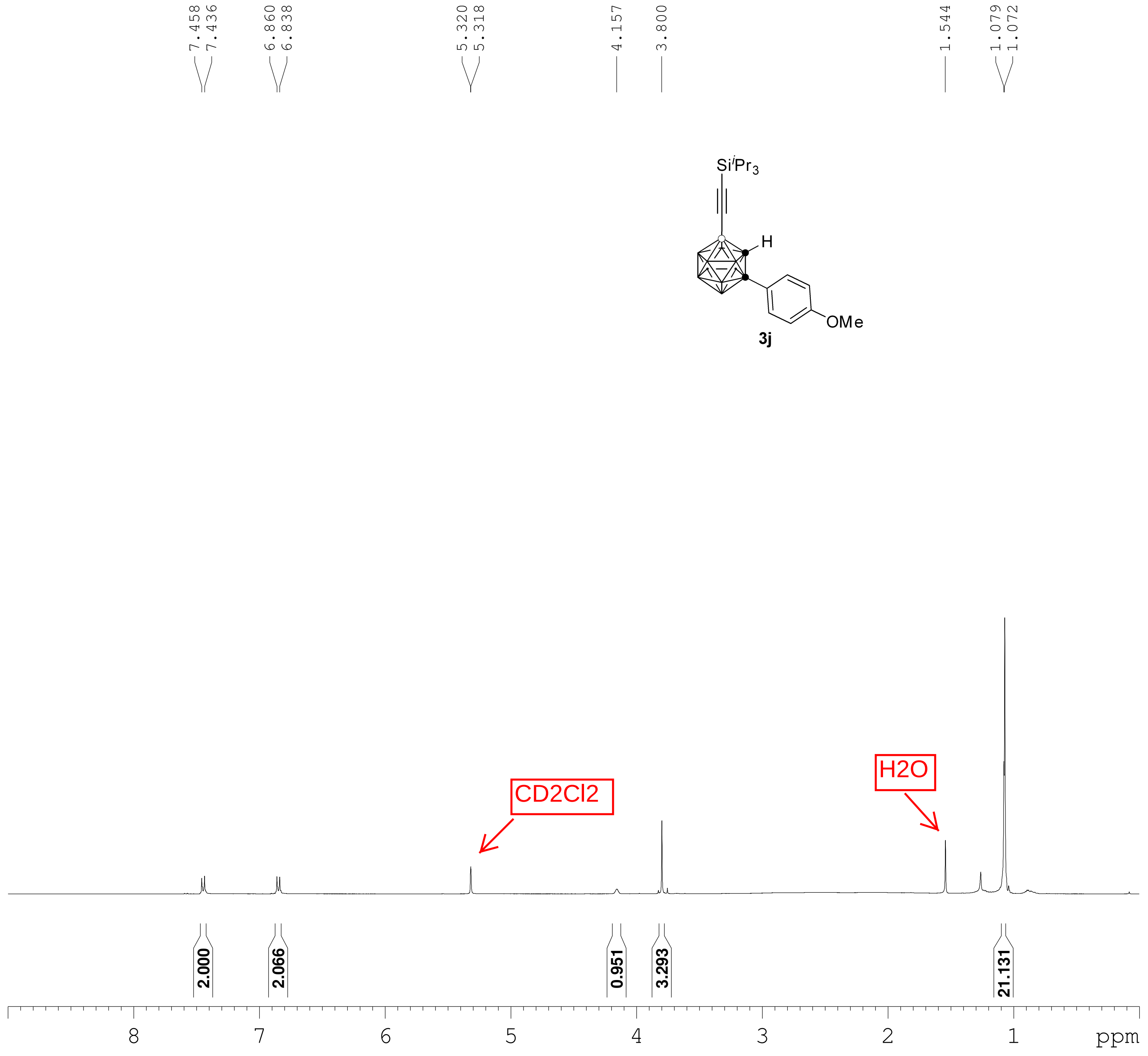

qyj-H-0364-cd2cl2

Bruker Advance III 400

NAME	qyj-H-0364-cd2cl2	
EXPNO	1	
PROCNO	1	
Date_	20141108	
Time	13.56	
INSTRUM	spect	
PROBHD	5 mm PADUL 13C	
PULPROG	zg30	
TD	65536	
SOLVENT	CD2Cl2	
NS	16	
DS	2	
SWH	8223.685	Hz
FIDRES	0.125483	Hz
AQ	3.9846387	sec
RG	203	
DW	60.800	usec
DE	6.50	usec
TE	296.7	K
D1	1.00000000	sec
TD0	1	

======== CHANNEL f1 ========

NUC1	1H	
P1	14.83	usec
PL1	0.00	dB
PL1W	8.31434441	W
SFO1	400.1324710	MHz
SI	32768	
SF	400.1300147	MHz
WDW	EM	
SSB	0	
LB	0.30	Hz
GB	0	
PC	1.00	

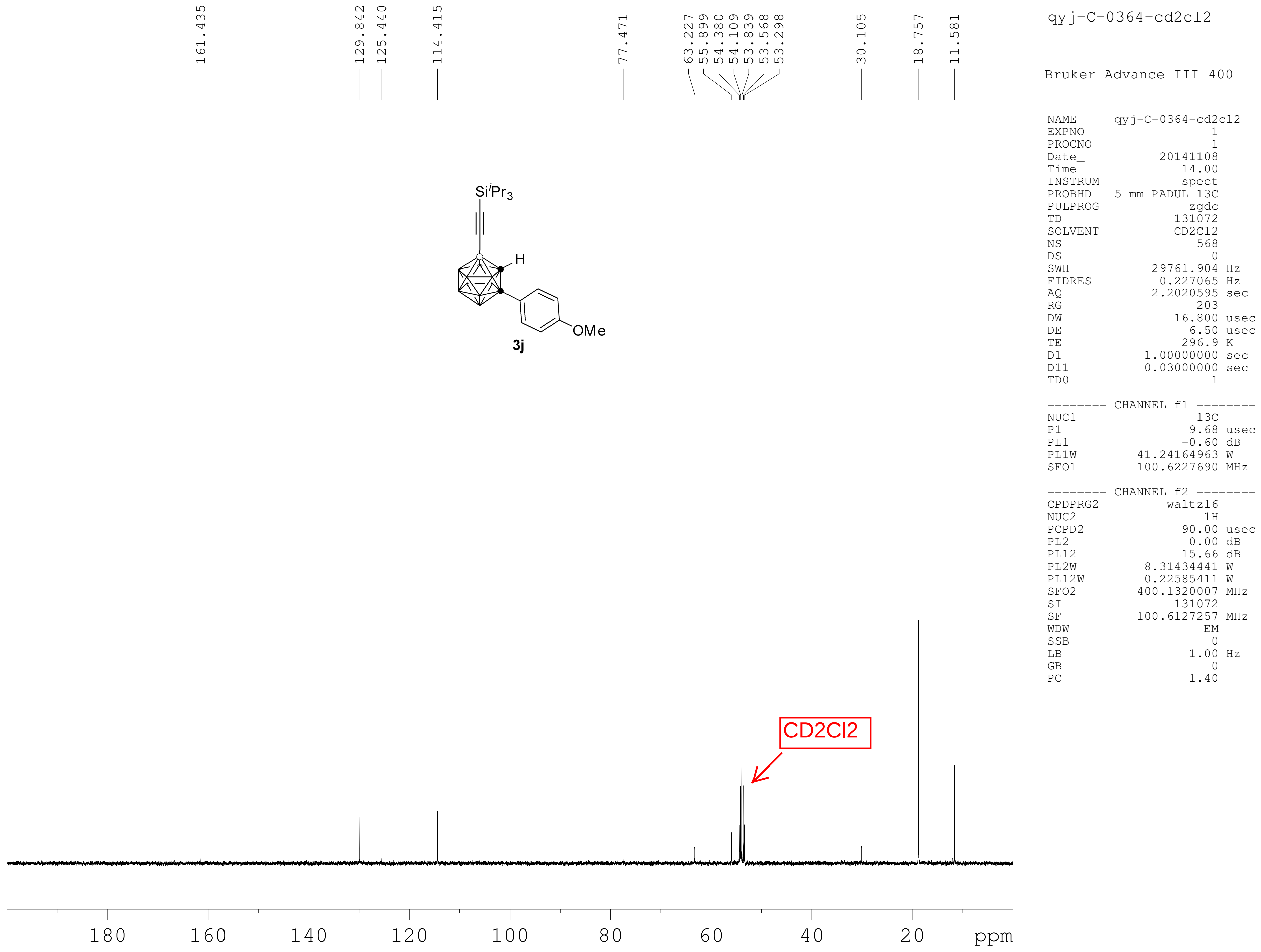
161.435
129.842
125.440
114.415
77.471
63.227
55.899
54.380
54.109
53.839
53.568
53.298
30.105
18.757
11.581
Si^{i}Pr$_3$
H
OMe
3j
CD2Cl2
180
160
140
120
100
80
60
40
20
ppm
qyj-C-0364-cd2cl2
Bruker Advance III 400
NAME qyj-C-0364-cd2cl2
EXPNO 1
PROCNO 1
Date_ 20141108
Time 14.00
INSTRUM spect
PROBHD 5 mm PADUL 13C
PULPROG zgdc
TD 131072
SOLVENT CD2Cl2
NS 568
DS 0
SWH 29761.904 Hz
FIDRES 0.227065 Hz
AQ 2.2020595 sec
RG 203
DW 16.800 usec
DE 6.50 usec
TE 296.9 K
D1 1.00000000 sec
D11 0.03000000 sec
TD0 1
======== CHANNEL f1 ========
NUC1 13C
P1 9.68 usec
PL1 -0.60 dB
PL1W 41.24164963 W
SFO1 100.6227690 MHz
======== CHANNEL f2 ========
CPDPRG2 waltz16
NUC2 1H
PCPD2 90.00 usec
PL2 0.00 dB
PL12 15.66 dB
PL2W 8.31434441 W
PL12W 0.22585411 W
SFO2 400.1320007 MHz
SI 131072
SF 100.6127257 MHz
WDW EM
SSB 0
LB 1.00 Hz
GB 0
PC 1.40

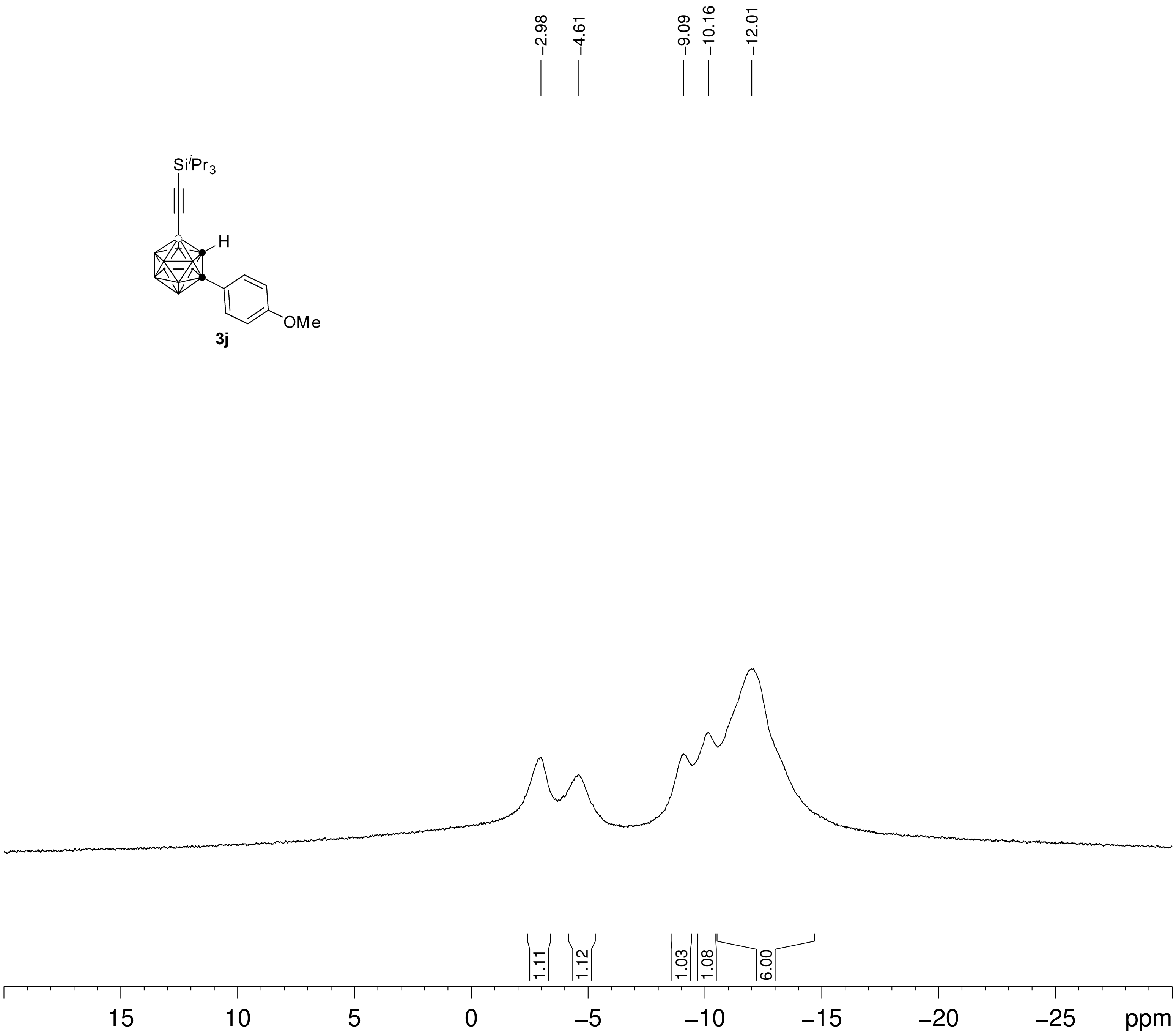

qyj-B-0364-CD2Cl2

NAME	qyj-B-0364-CD2Cl2	
EXPNO	1	
PROCNO	1	
Date_	20141108	
Time	15.38	
INSTRUM	spect	
PROBHD	5 mm PABBO BB-	
PULPROG	zgdc	
TD	65536	
SOLVENT	CDCl3	
NS	20	
DS	0	
SWH	25510.203	Hz
FIDRES	0.389255	Hz
AQ	1.2845556	sec
RG	406	
DW	19.600	usec
DE	6.50	usec
TE	299.3	K
D1	5.00000000	sec
D11	0.03000000	sec
TD0	1	
======== CHANNEL f1 ========		
NUC1	11B	
P1	7.60	usec
PL1	-3.00	dB
PL1W	55.13059616	W
SFO1	128.3968556	MHz
======== CHANNEL f2 ========		
CPDPRG2	waltz16	
NUC2	1H	
PCPD2	90.00	usec
PL2	-1.00	dB
PL12	15.16	dB
PL2W	13.56617069	W
PL12W	0.32844096	W
SFO2	400.1916008	MHz
SI	32768	
SF	128.3969291	MHz
WDW	EM	
SSB	0	
LB	3.00	Hz
GB	0	
PC	1.40	

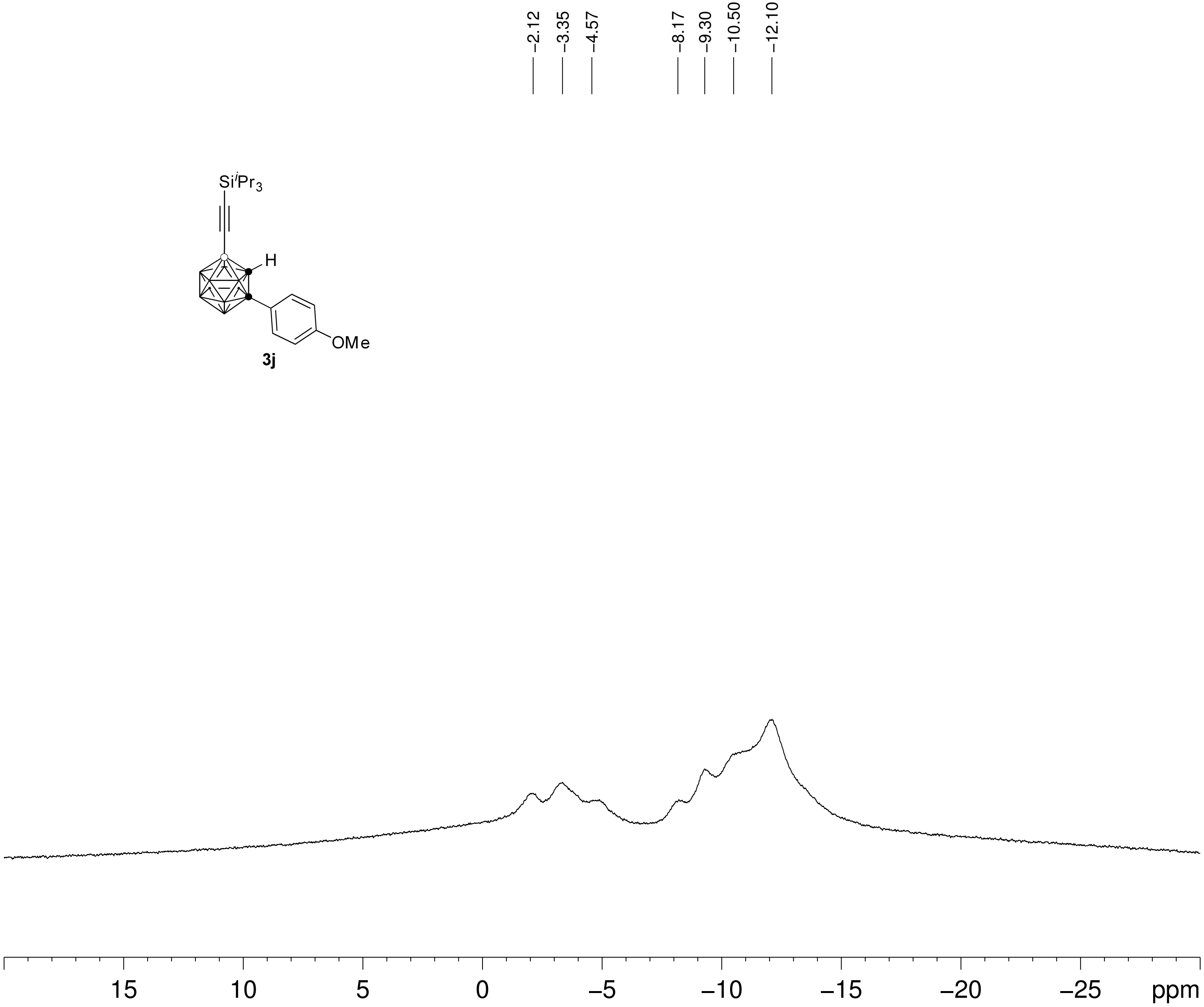

3j

qyj-B-0364-CD2Cl2(C

NAME	qyj-B-0364-CD2Cl2(C)
EXPNO	1
PROCNO	1
Date_	20141108
Time	15.41
INSTRUM	spect
PROBHD	5 mm PABBO BB-
PULPROG	zg30
TD	65536
SOLVENT	CDCl3
NS	40
DS	0
SWH	25510.203 Hz
FIDRES	0.389255 Hz
AQ	1.2845556 sec
RG	362
DW	19.600 usec
DE	6.50 usec
TE	298.8 K
D1	5.00000000 sec
TD0	1

======== CHANNEL f1 ========

NUC1	11B
P1	7.60 usec
PL1	-3.00 dB
PL1W	55.13059616 W
SFO1	128.3968556 MHz
SI	32768
SF	128.3968865 MHz
WDW	EM
SSB	0
LB	3.00 Hz
GB	0
PC	1.40

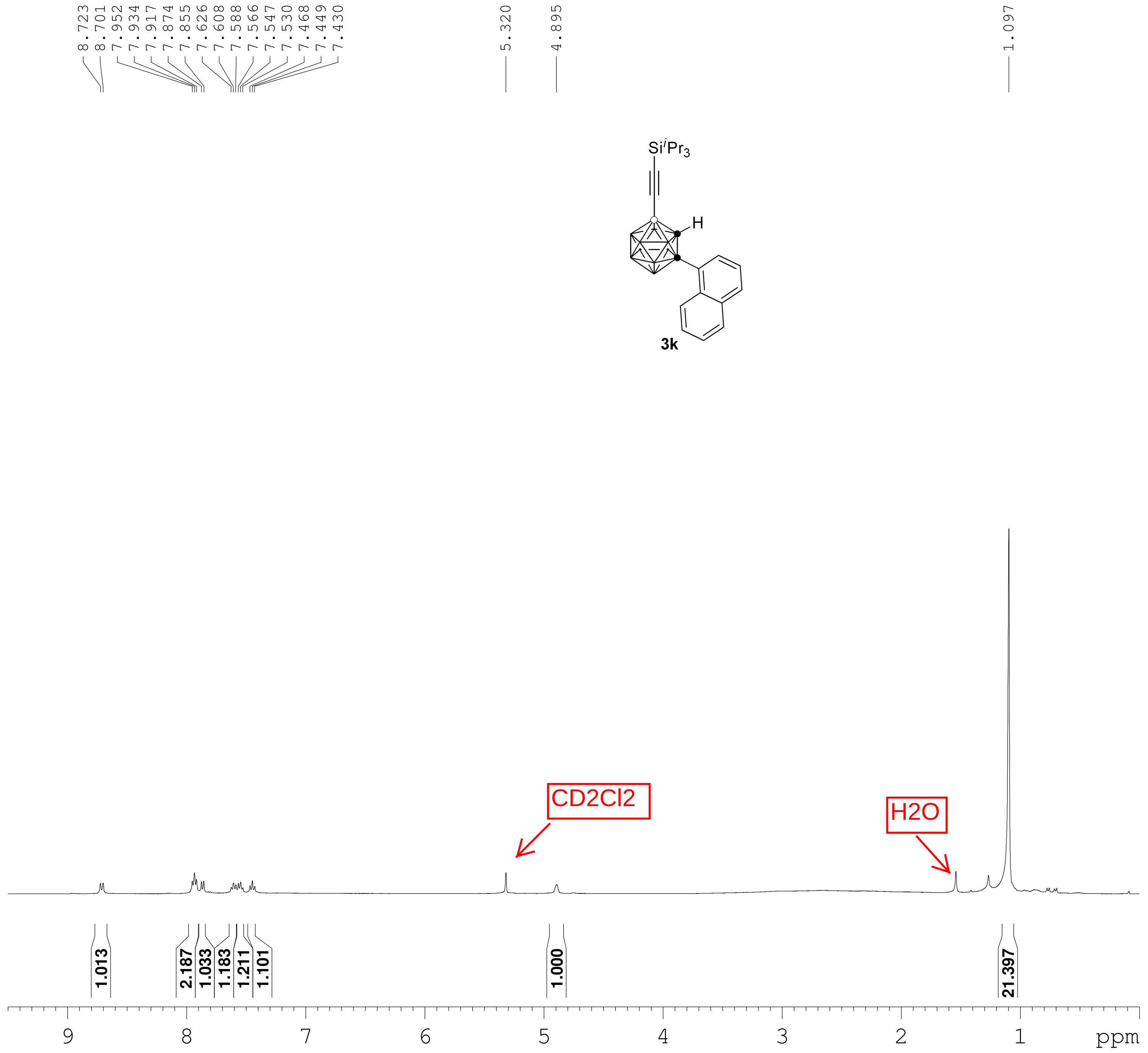

qyj-H-0367-cd2cl2

Bruker Advance III 400

NAME	qyj-H-0367-cd2cl2
EXPNO	1
PROCNO	1
Date_	20141113
Time	14.08
INSTRUM	spect
PROBHD	5 mm PADUL 13C
PULPROG	zg30
TD	65536
SOLVENT	CD2Cl2
NS	12
DS	2
SWH	8223.685 Hz
FIDRES	0.125483 Hz
AQ	3.9846387 sec
RG	203
DW	60.800 usec
DE	6.50 usec
TE	297.4 K
D1	1.00000000 sec
TD0	1

======== CHANNEL f1 ========

NUC1	1H
P1	14.83 usec
PL1	0.00 dB
PL1W	8.31434441 W
SFO1	400.1324710 MHz
SI	32768
SF	400.1300136 MHz
WDW	EM
SSB	0
LB	0.30 Hz
GB	0
PC	1.00

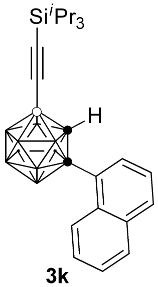

3k

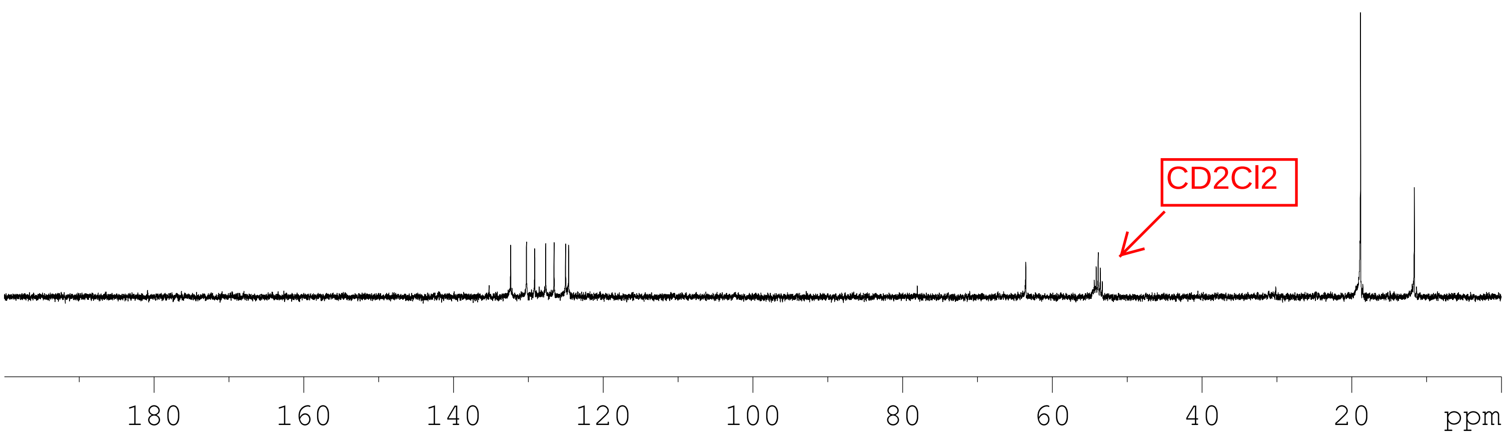

qyj-C-0367-cd2cl2

Bruker Advance III 400

NAME	qyj-C-0367-cd2cl2
EXPNO	1
PROCNO	1
Date_	20141115
Time	14.14
INSTRUM	spect
PROBHD	5 mm PADUL 13C
PULPROG	zgdc
TD	131072
SOLVENT	CD2Cl2
NS	360
DS	0
SWH	29761.904 Hz
FIDRES	0.227065 Hz
AQ	2.2020595 sec
RG	203
DW	16.800 usec
DE	6.50 usec
TE	296.7 K
D1	1.00000000 sec
D11	0.03000000 sec
TD0	1

======== CHANNEL f1 ========

NUC1	13C
P1	9.68 usec
PL1	-0.60 dB
PL1W	41.24164963 W
SFO1	100.6227690 MHz

======== CHANNEL f2 ========

CPDPRG2	waltz16
NUC2	1H
PCPD2	90.00 usec
PL2	0.00 dB
PL12	15.66 dB
PL2W	8.31434441 W
PL12W	0.22585411 W
SFO2	400.1320007 MHz
SI	131072
SF	100.6127249 MHz
WDW	EM
SSB	0
LB	1.00 Hz
GB	0
PC	1.40

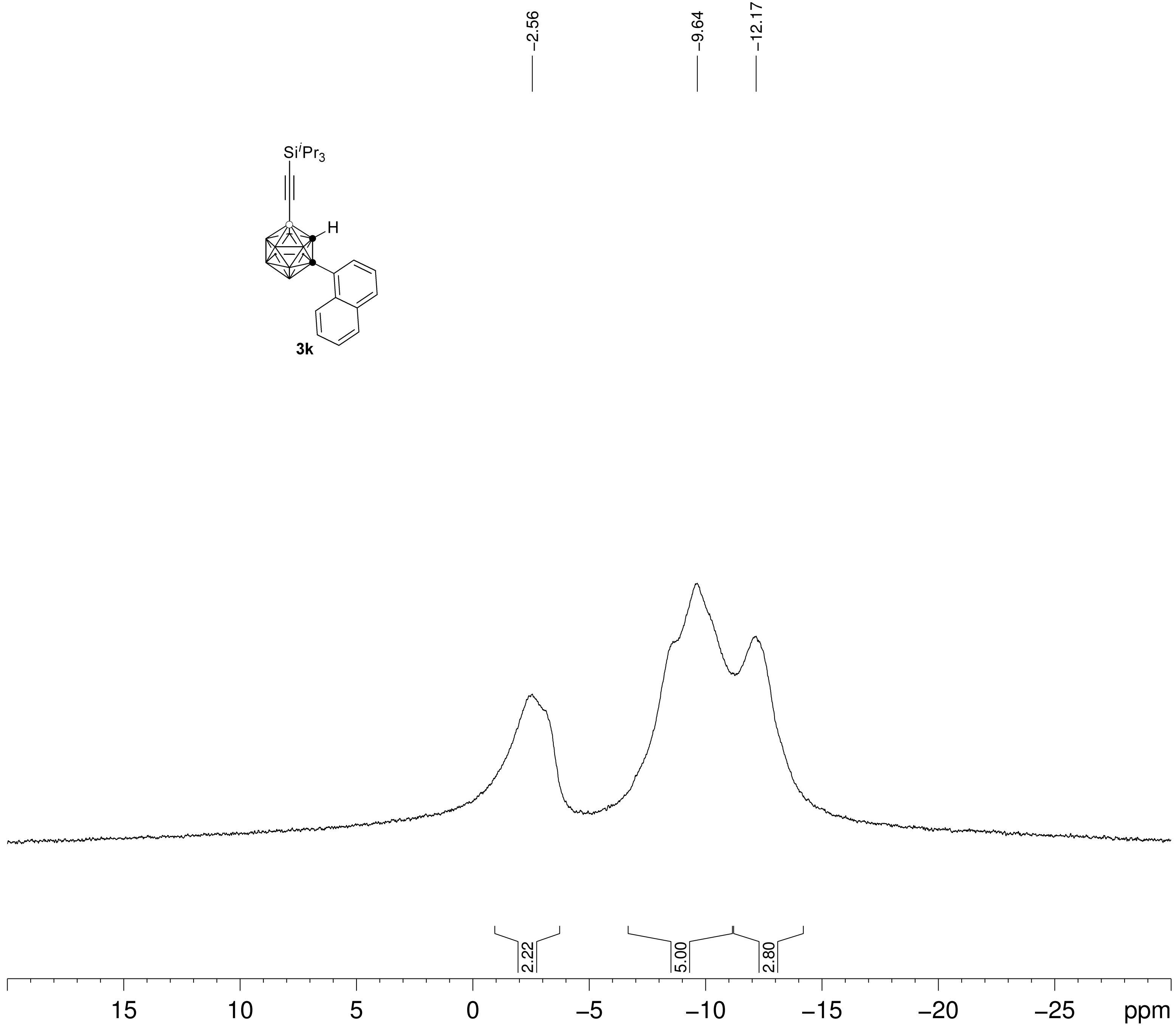

qyj-B-0367-CD2Cl2

```
NAME     qyj-B-0367-CD2Cl2
EXPNO                    1
PROCNO                   1
Date_             20141115
Time                 14.40
INSTRUM              spect
PROBHD   5 mm PABBO BB-
PULPROG               zgdc
TD                   65536
SOLVENT              CDCl3
NS                       8
DS                       0
SWH              25510.203 Hz
FIDRES            0.389255 Hz
AQ               1.2845556 sec
RG                     228
DW                  19.600 usec
DE                    6.50 usec
TE                   298.7 K
D1              5.00000000 sec
D11             0.03000000 sec
TD0                      1

======== CHANNEL f1 ========
NUC1                   11B
P1                    7.60 usec
PL1                  -3.00 dB
PL1W           55.13059616 W
SFO1           128.3968556 MHz

======== CHANNEL f2 ========
CPDPRG2            waltz16
NUC2                    1H
PCPD2                90.00 usec
PL2                  -1.00 dB
PL12                 15.16 dB
PL2W           13.56617069 W
PL12W           0.32844096 W
SFO2           400.1916008 MHz
SI                   32768
SF             128.3969291 MHz
WDW                     EM
SSB                      0
LB                    3.00 Hz
GB                       0
PC                    1.40
```

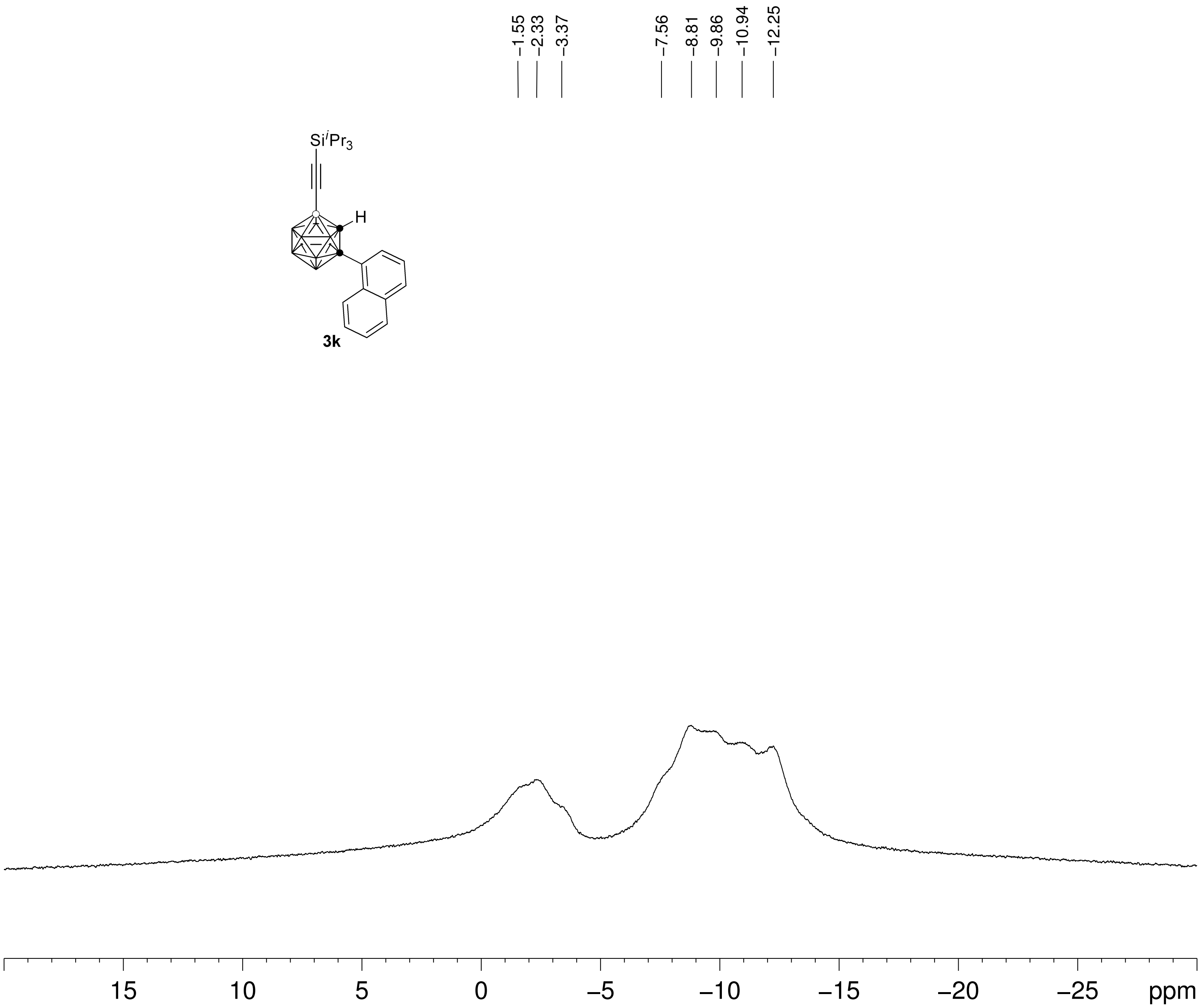

qyj-B-0367-CD2Cl2(C

NAME	qyj-B-0367-CD2Cl2(C)
EXPNO	1
PROCNO	1
Date_	20141115
Time	14.43
INSTRUM	spect
PROBHD	5 mm PABBO BB-
PULPROG	zg30
TD	65536
SOLVENT	CDCl3
NS	24
DS	0
SWH	25510.203 Hz
FIDRES	0.389255 Hz
AQ	1.2845556 sec
RG	406
DW	19.600 usec
DE	6.50 usec
TE	298.3 K
D1	5.00000000 sec
TD0	1

======== CHANNEL f1 ========

NUC1	11B
P1	7.60 usec
PL1	-3.00 dB
PL1W	55.13059616 W
SFO1	128.3968556 MHz
SI	32768
SF	128.3968865 MHz
WDW	EM
SSB	0
LB	3.00 Hz
GB	0
PC	1.40

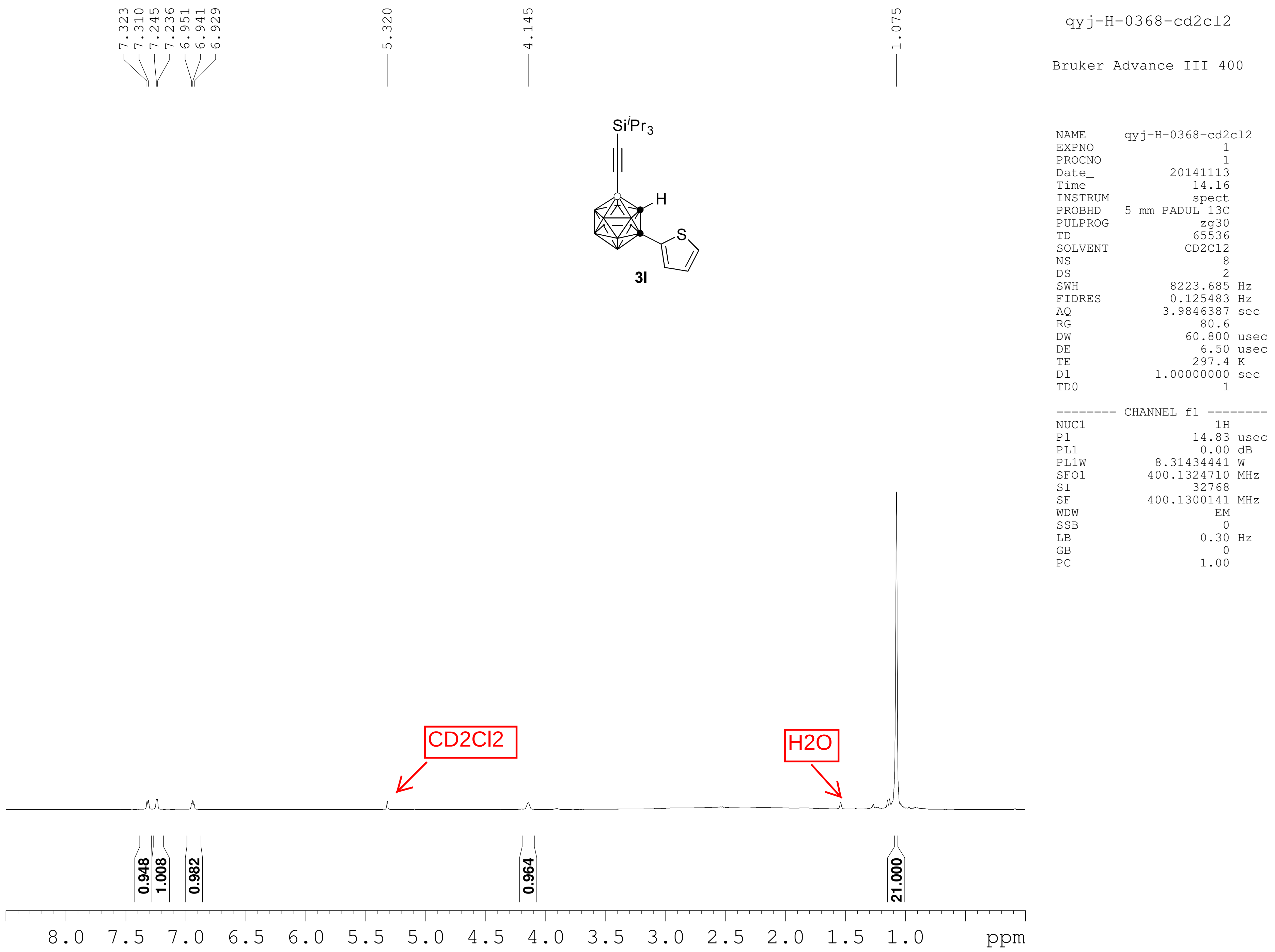
7.323
7.310
7.245
7.236
6.951
6.941
6.929
5.320
4.145
1.075
qyj-H-0368-cd2cl2
Bruker Advance III 400
SiiPr3
H
S
3l
NAME qyj-H-0368-cd2cl2
EXPNO 1
PROCNO 1
Date_ 20141113
Time 14.16
INSTRUM spect
PROBHD 5 mm PADUL 13C
PULPROG zg30
TD 65536
SOLVENT CD2Cl2
NS 8
DS 2
SWH 8223.685 Hz
FIDRES 0.125483 Hz
AQ 3.9846387 sec
RG 80.6
DW 60.800 usec
DE 6.50 usec
TE 297.4 K
D1 1.00000000 sec
TD0 1
======== CHANNEL f1 ========
NUC1 1H
P1 14.83 usec
PL1 0.00 dB
PL1W 8.31434441 W
SFO1 400.1324710 MHz
SI 32768
SF 400.1300141 MHz
WDW EM
SSB 0
LB 0.30 Hz
GB 0
PC 1.00
CD2Cl2
H2O
0.948
1.008
0.982
0.964
21.000
8.0 7.5 7.0 6.5 6.0 5.5 5.0 4.5 4.0 3.5 3.0 2.5 2.0 1.5 1.0 ppm

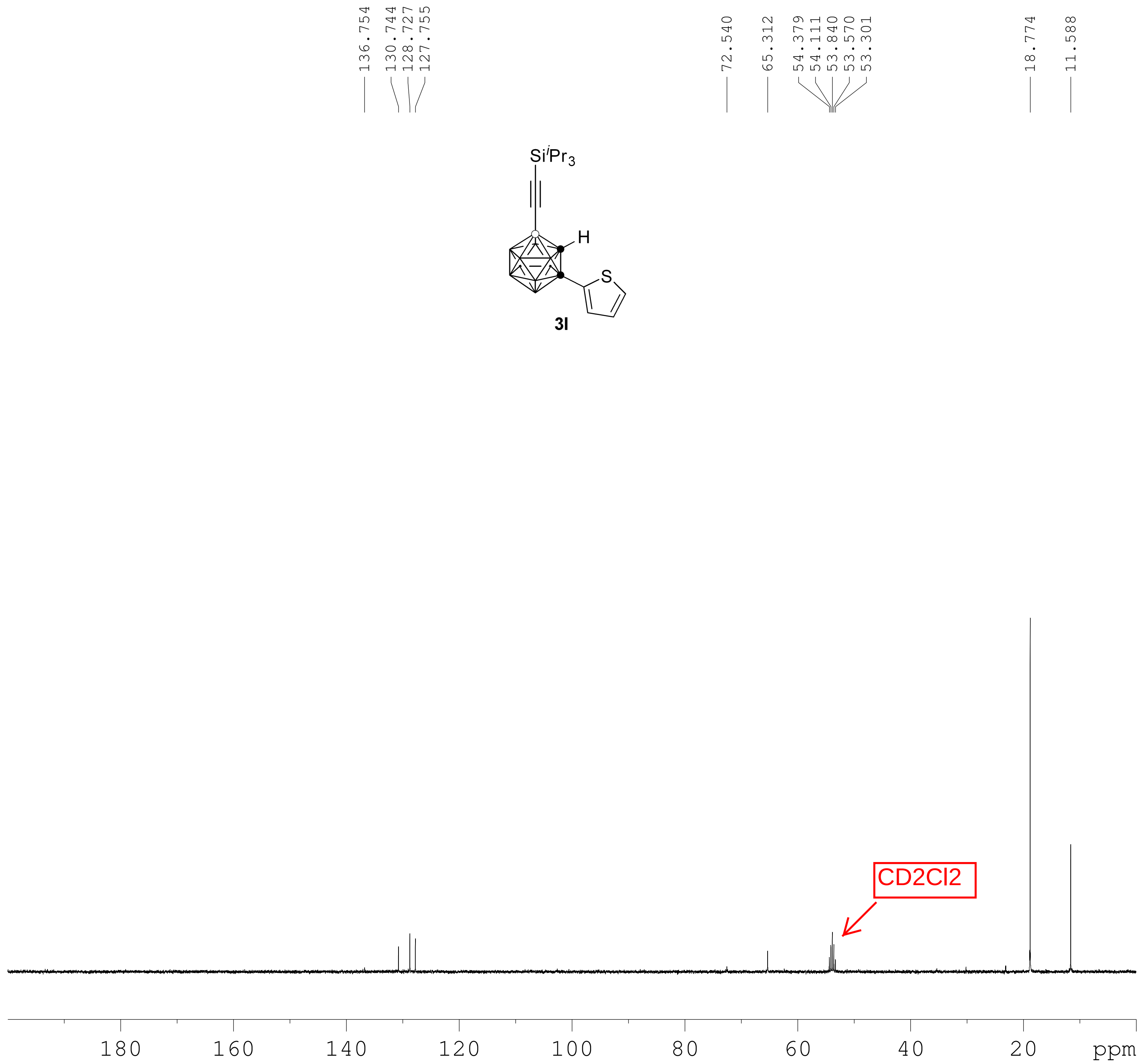

qyj-C-0368-cd2cl2

Bruker Advance III 400

NAME	qyj-C-0368-cd2cl2	
EXPNO	1	
PROCNO	1	
Date_	20141113	
Time	14.20	
INSTRUM	spect	
PROBHD	5 mm PADUL 13C	
PULPROG	zgdc	
TD	131072	
SOLVENT	CD2Cl2	
NS	125	
DS	0	
SWH	29761.904	Hz
FIDRES	0.227065	Hz
AQ	2.2020595	sec
RG	203	
DW	16.800	usec
DE	6.50	usec
TE	297.9	K
D1	1.00000000	sec
D11	0.03000000	sec
TD0	1	

======== CHANNEL f1 ========

NUC1	13C	
P1	9.68	usec
PL1	-0.60	dB
PL1W	41.24164963	W
SFO1	100.6227690	MHz

======== CHANNEL f2 ========

CPDPRG2	waltz16	
NUC2	1H	
PCPD2	90.00	usec
PL2	0.00	dB
PL12	15.66	dB
PL2W	8.31434441	W
PL12W	0.22585411	W
SFO2	400.1320007	MHz
SI	131072	
SF	100.6127243	MHz
WDW	EM	
SSB	0	
LB	1.00	Hz
GB	0	
PC	1.40	

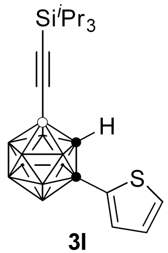

3l

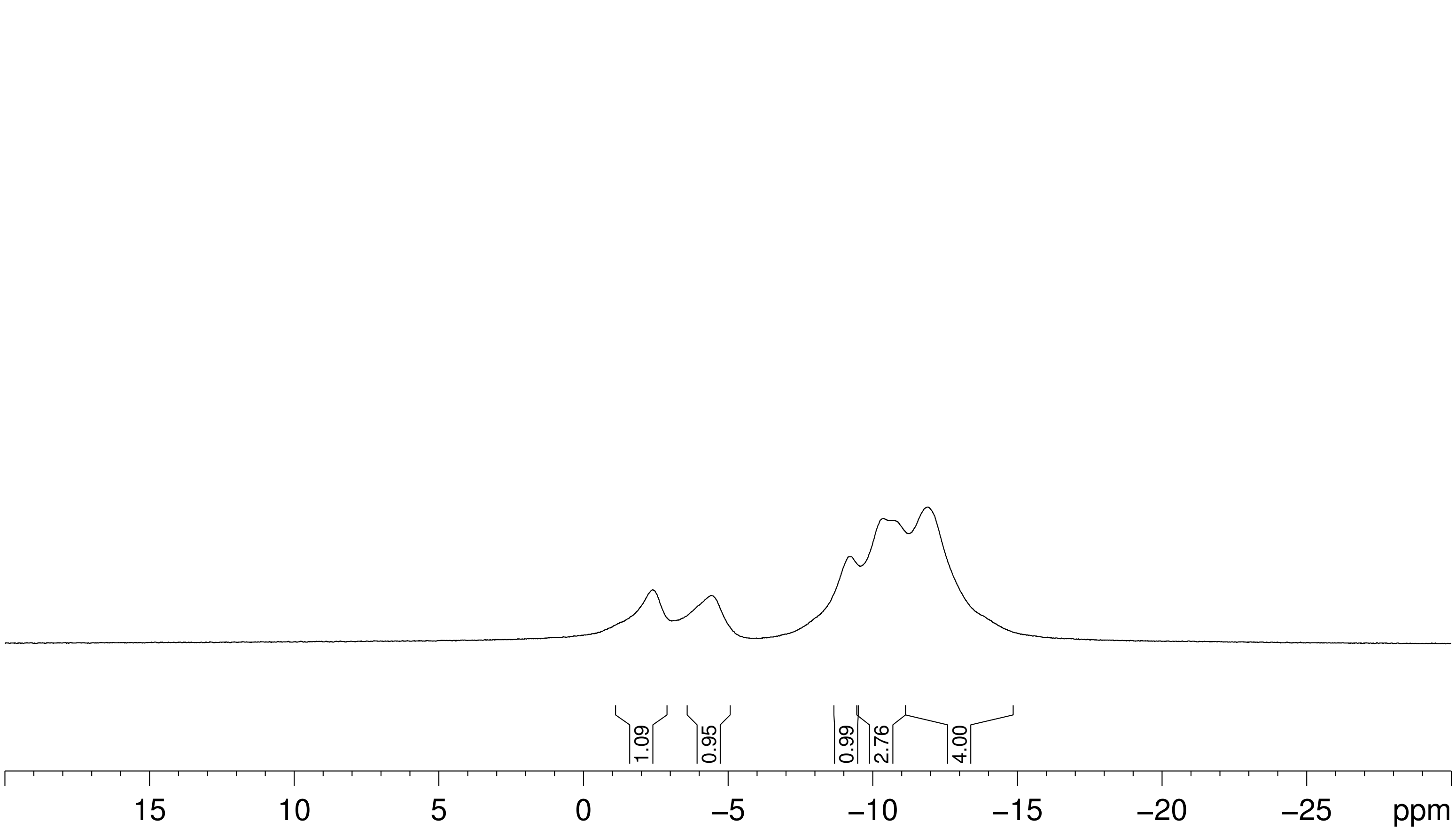

qyj-B-0368-CD2Cl2

NAME	qyj-B-0368-CD2Cl2
EXPNO	1
PROCNO	1
Date_	20141115
Time	14.23
INSTRUM	spect
PROBHD	5 mm PABBO BB-
PULPROG	zgdc
TD	65536
SOLVENT	CDCl3
NS	5
DS	0
SWH	25510.203 Hz
FIDRES	0.389255 Hz
AQ	1.2845556 sec
RG	228
DW	19.600 usec
DE	6.50 usec
TE	298.8 K
D1	5.00000000 sec
D11	0.03000000 sec
TD0	1
======== CHANNEL f1 ========	
NUC1	11B
P1	7.60 usec
PL1	-3.00 dB
PL1W	55.13059616 W
SFO1	128.3968556 MHz
======== CHANNEL f2 ========	
CPDPRG2	waltz16
NUC2	1H
PCPD2	90.00 usec
PL2	-1.00 dB
PL12	15.16 dB
PL2W	13.56617069 W
PL12W	0.32844096 W
SFO2	400.1916008 MHz
SI	32768
SF	128.3969291 MHz
WDW	EM
SSB	0
LB	3.00 Hz
GB	0
PC	1.40

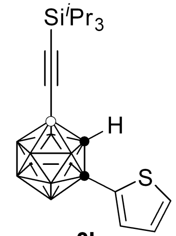

3l

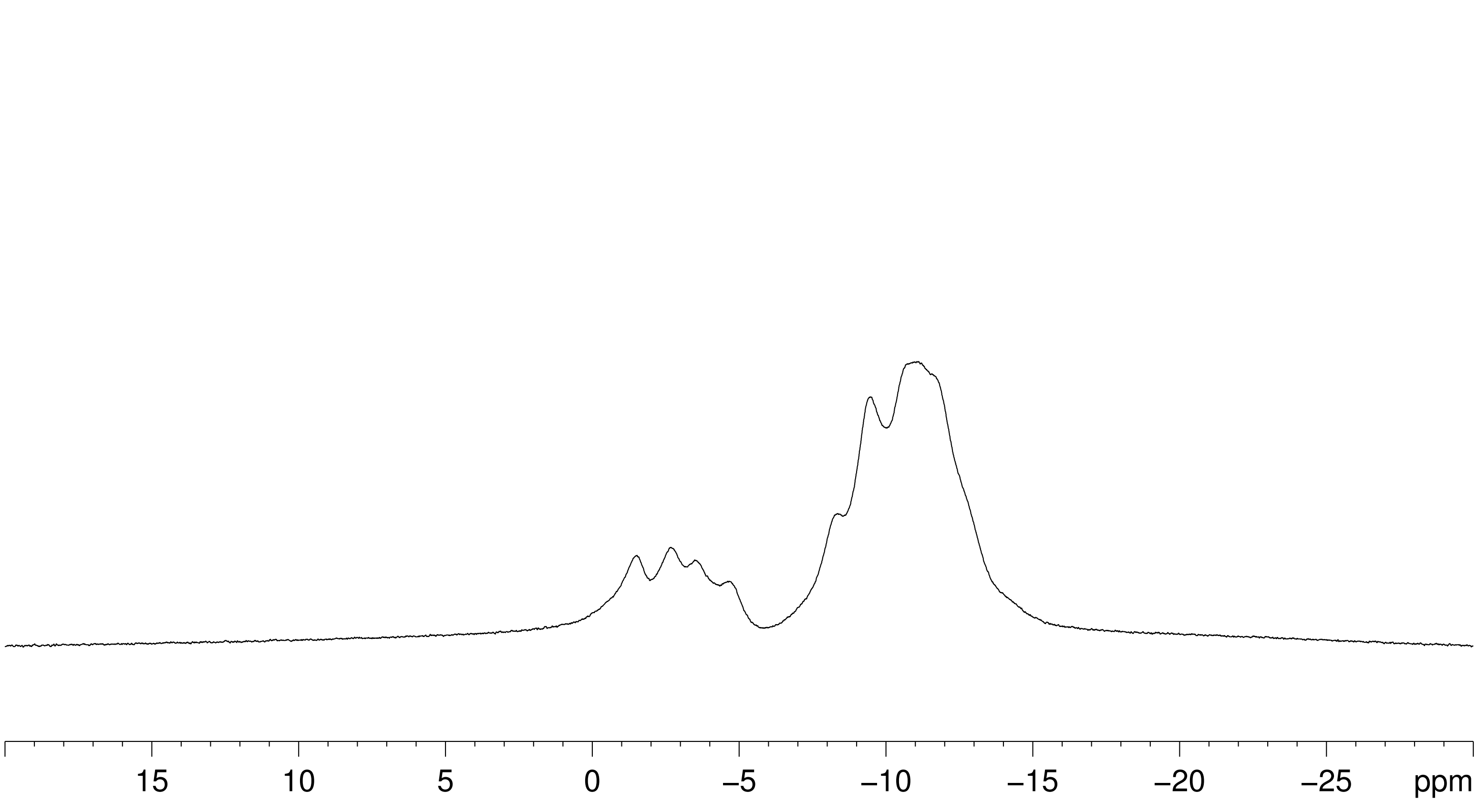

qyj-B-0368-CD2Cl2(C

NAME	qyj-B-0368-CD2Cl2(C)
EXPNO	1
PROCNO	1
Date_	20141115
Time	14.24
INSTRUM	spect
PROBHD	5 mm PABBO BB-
PULPROG	zg30
TD	65536
SOLVENT	CDCl3
NS	16
DS	0
SWH	25510.203 Hz
FIDRES	0.389255 Hz
AQ	1.2845556 sec
RG	406
DW	19.600 usec
DE	6.50 usec
TE	298.6 K
D1	5.00000000 sec
TD0	1

======== CHANNEL f1 ========

NUC1	11B
P1	7.60 usec
PL1	-3.00 dB
PL1W	55.13059616 W
SFO1	128.3968556 MHz
SI	32768
SF	128.3968865 MHz
WDW	EM
SSB	0
LB	3.00 Hz
GB	0
PC	1.40

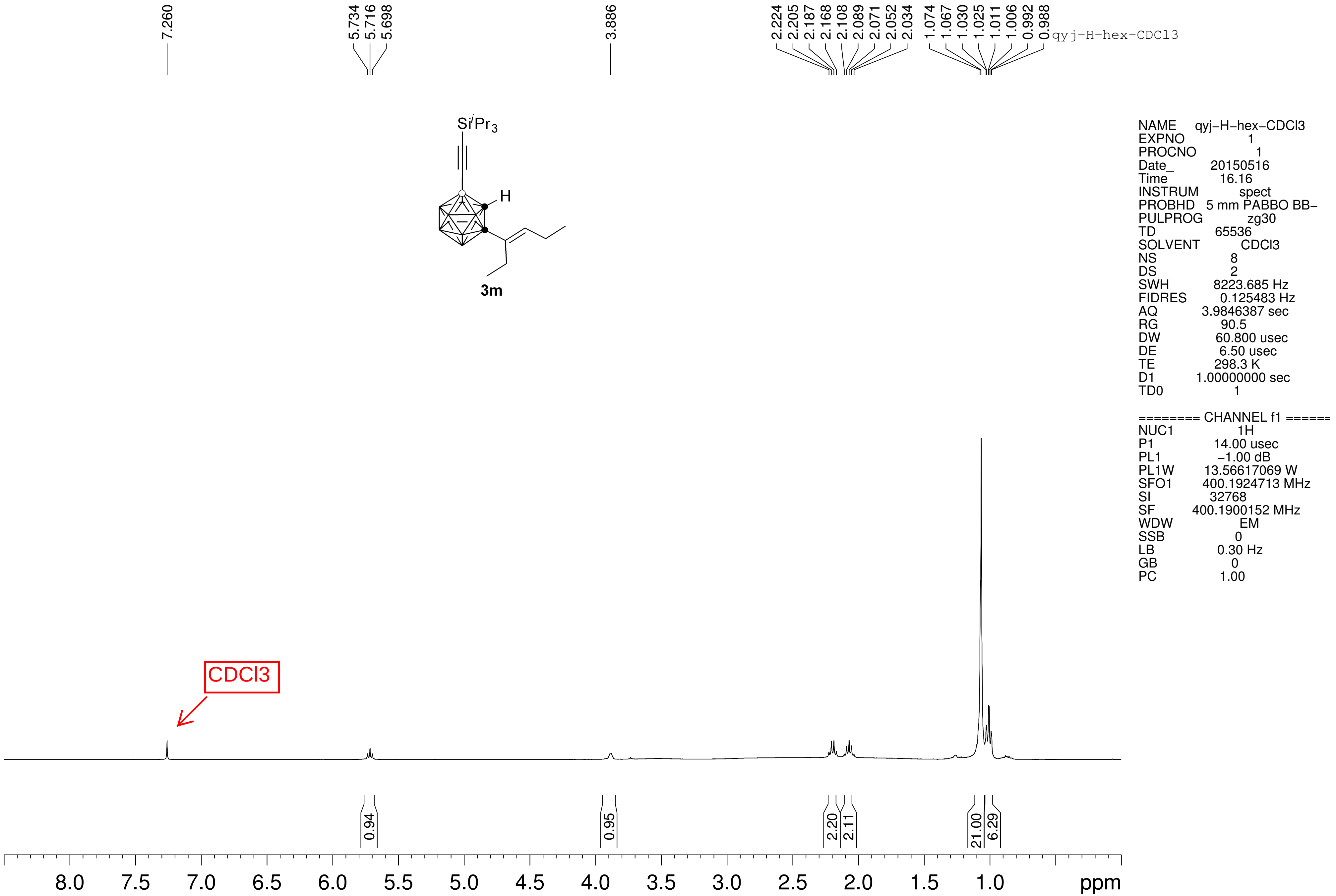
7.260
5.734
5.716
5.698
3.886
2.224
2.205
2.187
2.168
2.108
2.089
2.071
2.052
2.034
1.074
1.067
1.030
1.025
1.011
1.006
0.992
0.988
qyj-H-hex-CDCl3
3m
CDCl3
0.94
0.95
2.20
2.11
21.00
6.29
8.0 7.5 7.0 6.5 6.0 5.5 5.0 4.5 4.0 3.5 3.0 2.5 2.0 1.5 1.0 ppm
NAME qyj–H–hex–CDCl3
EXPNO 1
PROCNO 1
Date_ 20150516
Time 16.16
INSTRUM spect
PROBHD 5 mm PABBO BB–
PULPROG zg30
TD 65536
SOLVENT CDCl3
NS 8
DS 2
SWH 8223.685 Hz
FIDRES 0.125483 Hz
AQ 3.9846387 sec
RG 90.5
DW 60.800 usec
DE 6.50 usec
TE 298.3 K
D1 1.00000000 sec
TD0 1
======== CHANNEL f1 ======
NUC1 1H
P1 14.00 usec
PL1 –1.00 dB
PL1W 13.56617069 W
SFO1 400.1924713 MHz
SI 32768
SF 400.1900152 MHz
WDW EM
SSB 0
LB 0.30 Hz
GB 0
PC 1.00

qyj-C-hex-CDCl3

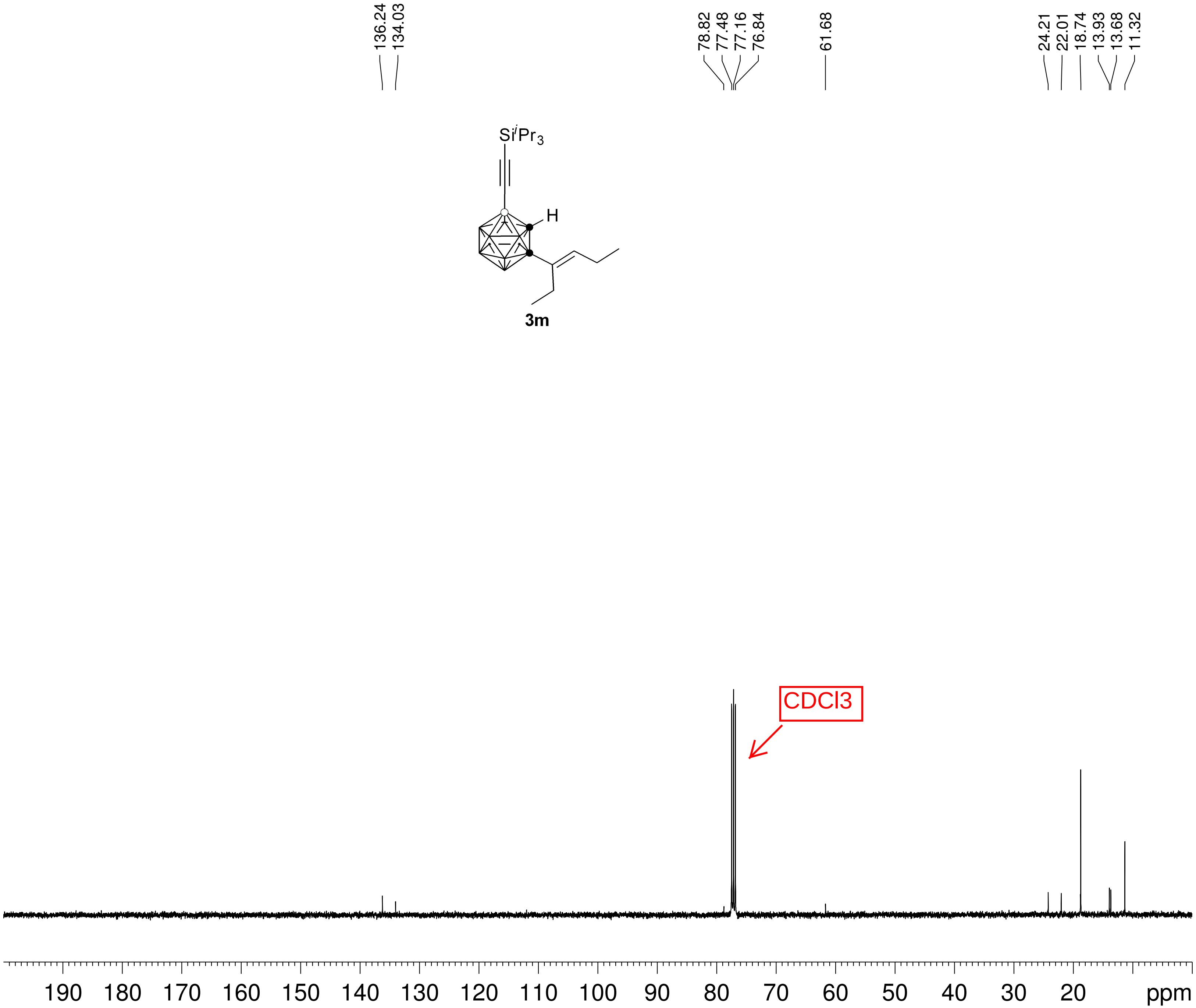

NAME	qyj-C-hex-CDCl3
EXPNO	1
PROCNO	1
Date_	20150516
Time	16.19
INSTRUM	spect
PROBHD	5 mm PABBO BB-
PULPROG	zgpg30
TD	65536
SOLVENT	CDCl3
NS	120
DS	4
SWH	24038.461 Hz
FIDRES	0.366798 Hz
AQ	1.3631988 sec
RG	203
DW	20.800 usec
DE	6.50 usec
TE	298.5 K
D1	2.00000000 sec
D11	0.03000000 sec
TD0	1
======== CHANNEL f1 ========	
NUC1	13C
P1	9.90 usec
PL1	-2.00 dB
PL1W	55.33689499 W
SFO1	100.6379183 MHz
======== CHANNEL f2 ========	
CPDPRG2	waltz16
NUC2	1H
PCPD2	90.00 usec
PL2	-1.00 dB
PL12	15.16 dB
PL13	18.62 dB
PL2W	13.56617069 W
PL12W	0.32844096 W
PL13W	0.14806664 W
SFO2	400.1916008 MHz
SI	32768
SF	100.6278427 MHz
WDW	EM
SSB	0
LB	1.00 Hz
GB	0
PC	1.40

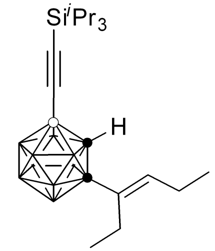

3m

qyj-B-hex-CDCl3

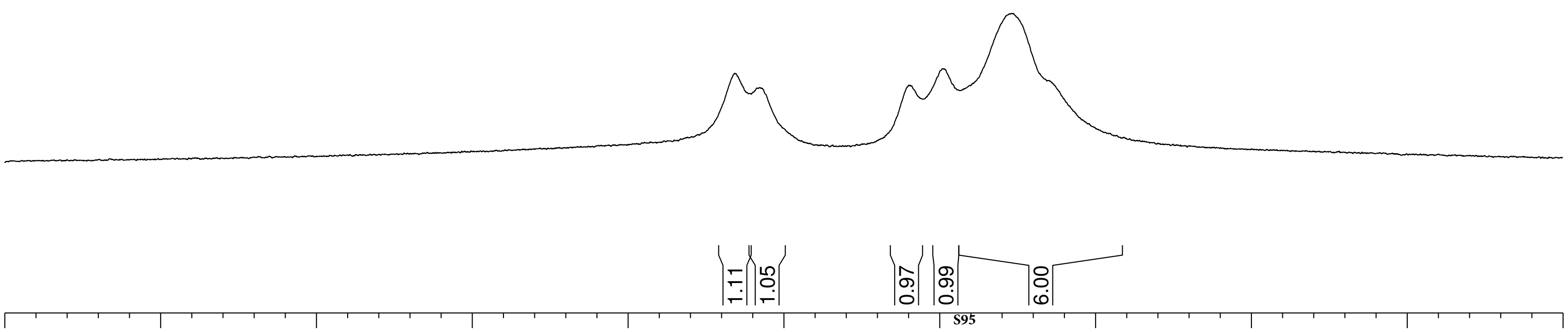


```
NAME      qyj-B-hex-CDCl3
EXPNO                   1
PROCNO                  1
Date_            20150516
Time                19.31
INSTRUM             spect
PROBHD    5 mm PABBO BB-
PULPROG              zgdc
TD                  65536
SOLVENT              C6D6
NS                     20
DS                      0
SWH             25510.203 Hz
FIDRES           0.389255 Hz
AQ              1.2845556 sec
RG                    362
DW                 19.600 usec
DE                   6.50 usec
TE                  299.2 K
D1             5.00000000 sec
D11            0.03000000 sec
TD0                     1

======== CHANNEL f1 ========
NUC1                  11B
P1                   7.60 usec
PL1                 -3.00 dB
PL1W          55.13059616 W
SFO1          128.3968556 MHz

======== CHANNEL f2 ========
CPDPRG2           waltz16
NUC2                   1H
PCPD2               90.00 usec
PL2                 -1.00 dB
PL12                15.16 dB
PL2W          13.56617069 W
PL12W          0.32844096 W
SFO2          400.1916008 MHz
SI                  32768
SF            128.3968847 MHz
WDW                    EM
SSB                     0
LB                   3.00 Hz
GB                      0
PC                   1.40
```

qyj-B-hex-CDCl3(C)

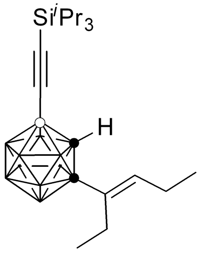

3m

NAME	qyj-B-hex-CDCl3(C)
EXPNO	1
PROCNO	1
Date_	20150516
Time	19.34
INSTRUM	spect
PROBHD	5 mm PABBO BB-
PULPROG	zg30
TD	65536
SOLVENT	C6D6
NS	24
DS	0
SWH	25510.203 Hz
FIDRES	0.389255 Hz
AQ	1.2845556 sec
RG	512
DW	19.600 usec
DE	6.50 usec
TE	298.8 K
D1	5.00000000 sec
TD0	1
======== CHANNEL f1 ========	
NUC1	11B
P1	7.60 usec
PL1	-3.00 dB
PL1W	55.13059616 W
SFO1	128.3968556 MHz
SI	32768
SF	128.3968865 MHz
WDW	EM
SSB	0
LB	3.00 Hz
GB	0
PC	1.40

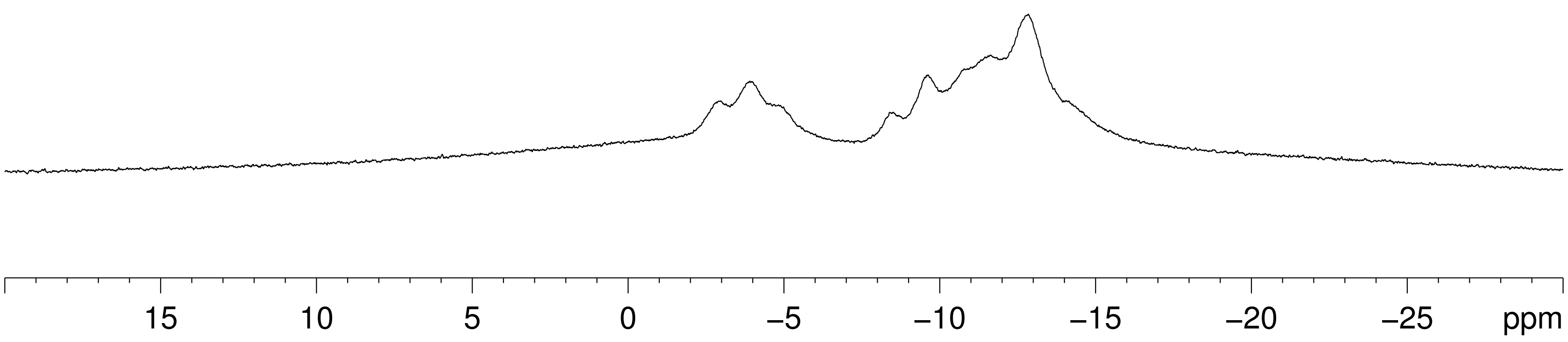

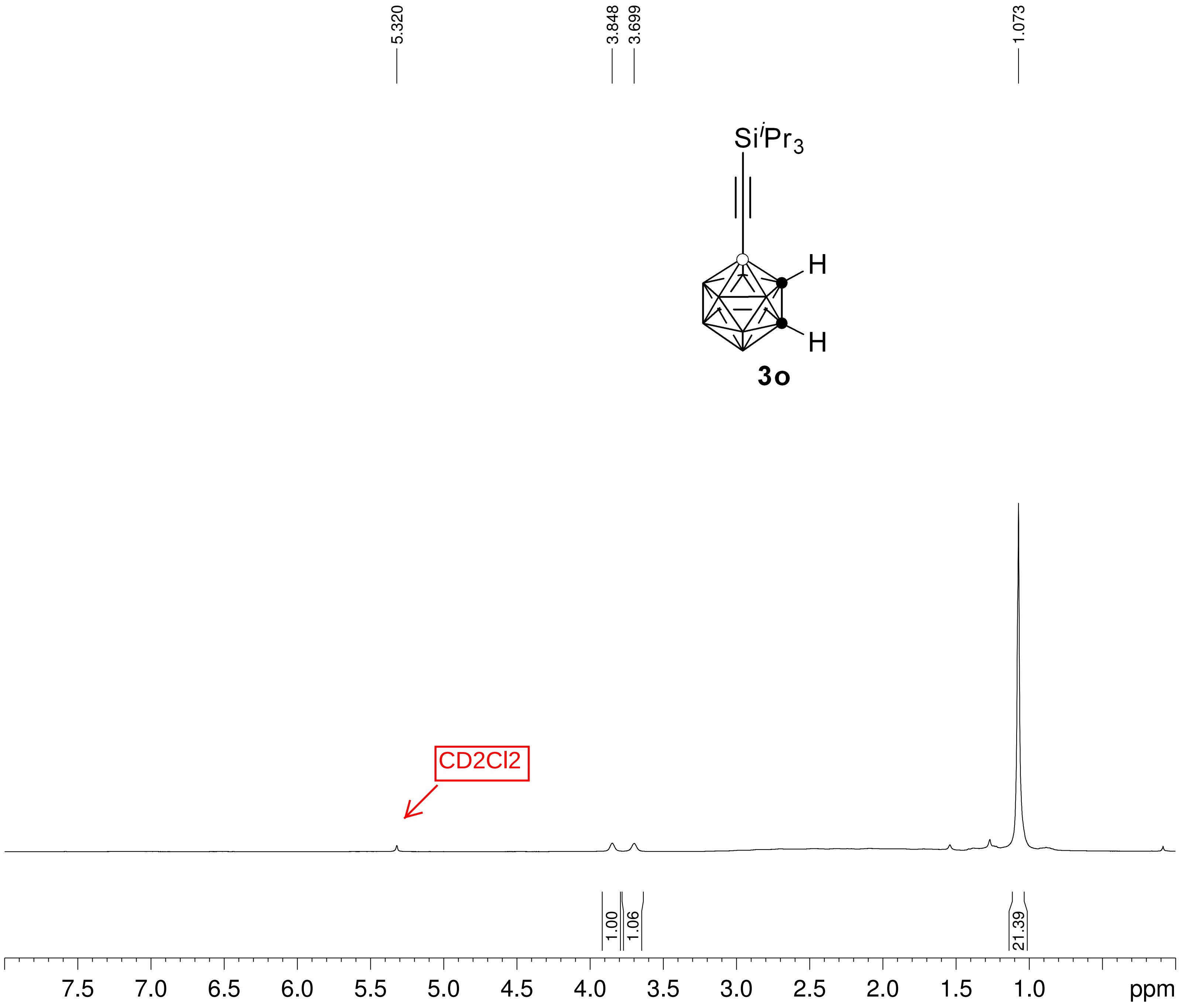

qyj-H-0363-CD2Cl2

NAME	qyj-H-0363-CD2Cl2
EXPNO	1
PROCNO	1
Date_	20141105
Time	16.01
INSTRUM	spect
PROBHD	5 mm PABBO BB-
PULPROG	zg30
TD	65536
SOLVENT	CD2Cl2
NS	8
DS	2
SWH	8223.685 Hz
FIDRES	0.125483 Hz
AQ	3.9846387 sec
RG	36
DW	60.800 usec
DE	6.50 usec
TE	299.0 K
D1	1.00000000 sec
TD0	1

======== CHANNEL f1 =====

NUC1	1H
P1	14.00 usec
PL1	-1.00 dB
PL1W	13.56617069 W
SFO1	400.1924713 MHz
SI	32768
SF	400.1900213 MHz
WDW	EM
SSB	0
LB	0.30 Hz
GB	0
PC	1.00

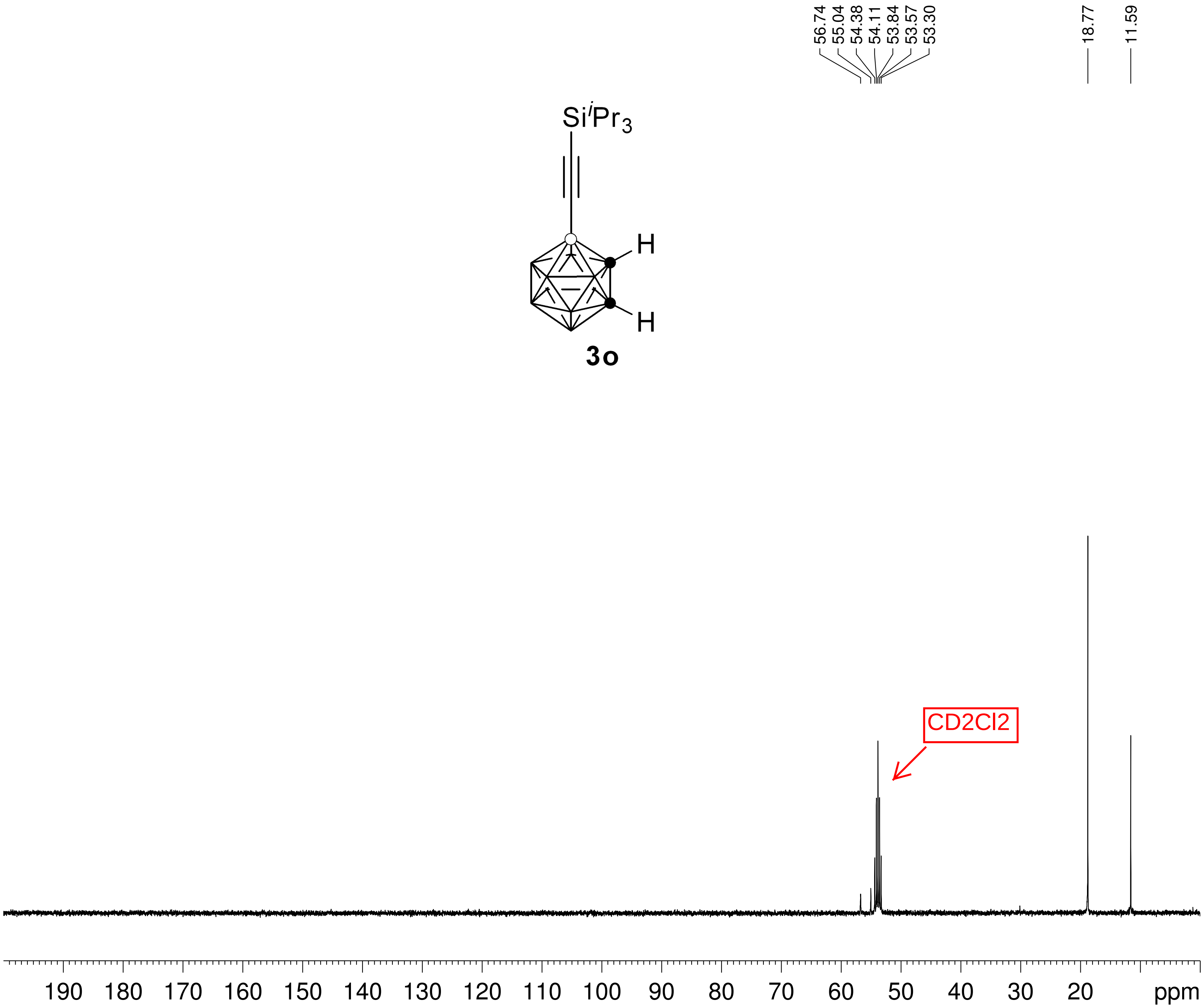

qyj-C-0363-CD2Cl2

```
NAME       qyj-C-0363-CD2Cl2
EXPNO                     1
PROCNO                    1
Date_              20141105
Time                  16.03
INSTRUM               spect
PROBHD     5 mm PABBO BB-
PULPROG              zgpg30
TD                    65536
SOLVENT              CD2Cl2
NS                      100
DS                        4
SWH               24038.461 Hz
FIDRES             0.366798 Hz
AQ                1.3631988 sec
RG                      181
DW                   20.800 usec
DE                     6.50 usec
TE                    299.4 K
D1               2.00000000 sec
D11              0.03000000 sec
TD0                       1

======== CHANNEL f1 ========
NUC1                    13C
P1                     9.90 usec
PL1                   -2.00 dB
PL1W            55.33689499 W
SFO1            100.6379183 MHz

======== CHANNEL f2 ========
CPDPRG2             waltz16
NUC2                     1H
PCPD2                 90.00 usec
PL2                   -1.00 dB
PL12                  15.16 dB
PL13                  18.62 dB
PL2W            13.56617069 W
PL12W            0.32844096 W
PL13W            0.14806664 W
SFO2            400.1916008 MHz
SI                    32768
SF              100.6278125 MHz
WDW                      EM
SSB                       0
LB                     1.00 Hz
GB                        0
PC                     1.40
```

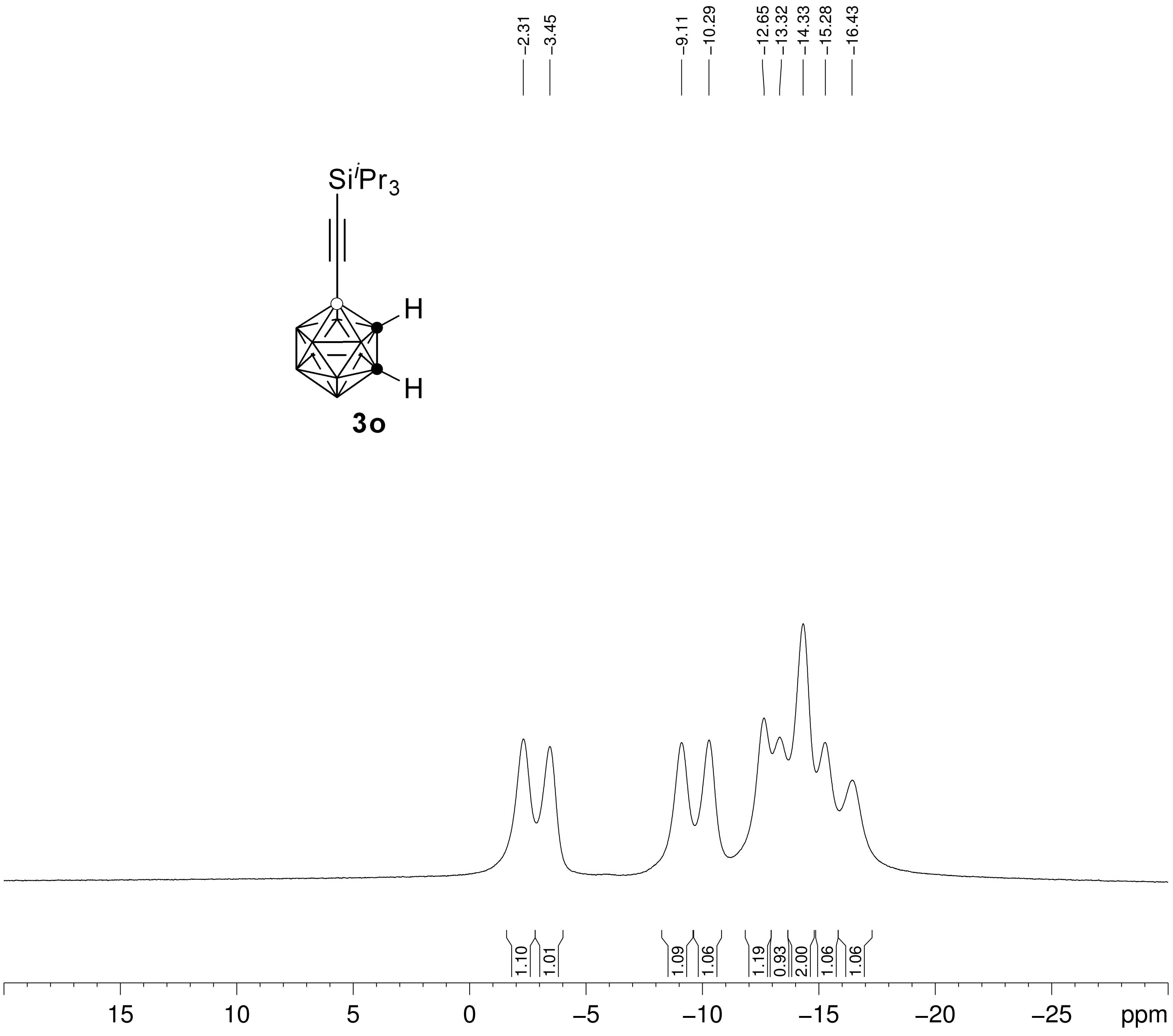

qyj-B-0363-CD2Cl2

NAME	qyj-B-0363-CD2Cl2
EXPNO	1
PROCNO	1
Date_	20141105
Time	15.52
INSTRUM	spect
PROBHD	5 mm PABBO BB-
PULPROG	zgdc
TD	65536
SOLVENT	C6D6
NS	12
DS	0
SWH	25510.203 Hz
FIDRES	0.389255 Hz
AQ	1.2845556 sec
RG	181
DW	19.600 usec
DE	6.50 usec
TE	299.0 K
D1	5.00000000 sec
D11	0.03000000 sec
TD0	1

======== CHANNEL f1 ========

NUC1	11B
P1	7.60 usec
PL1	-3.00 dB
PL1W	55.13059616 W
SFO1	128.3968556 MHz

======== CHANNEL f2 ========

CPDPRG2	waltz16
NUC2	1H
PCPD2	90.00 usec
PL2	-1.00 dB
PL12	15.16 dB
PL2W	13.56617069 W
PL12W	0.32844096 W
SFO2	400.1916008 MHz
SI	32768
SF	128.3969291 MHz
WDW	EM
SSB	0
LB	3.00 Hz
GB	0
PC	1.40

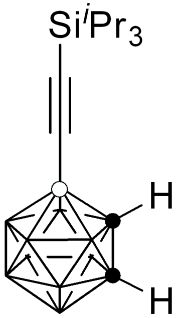

3o

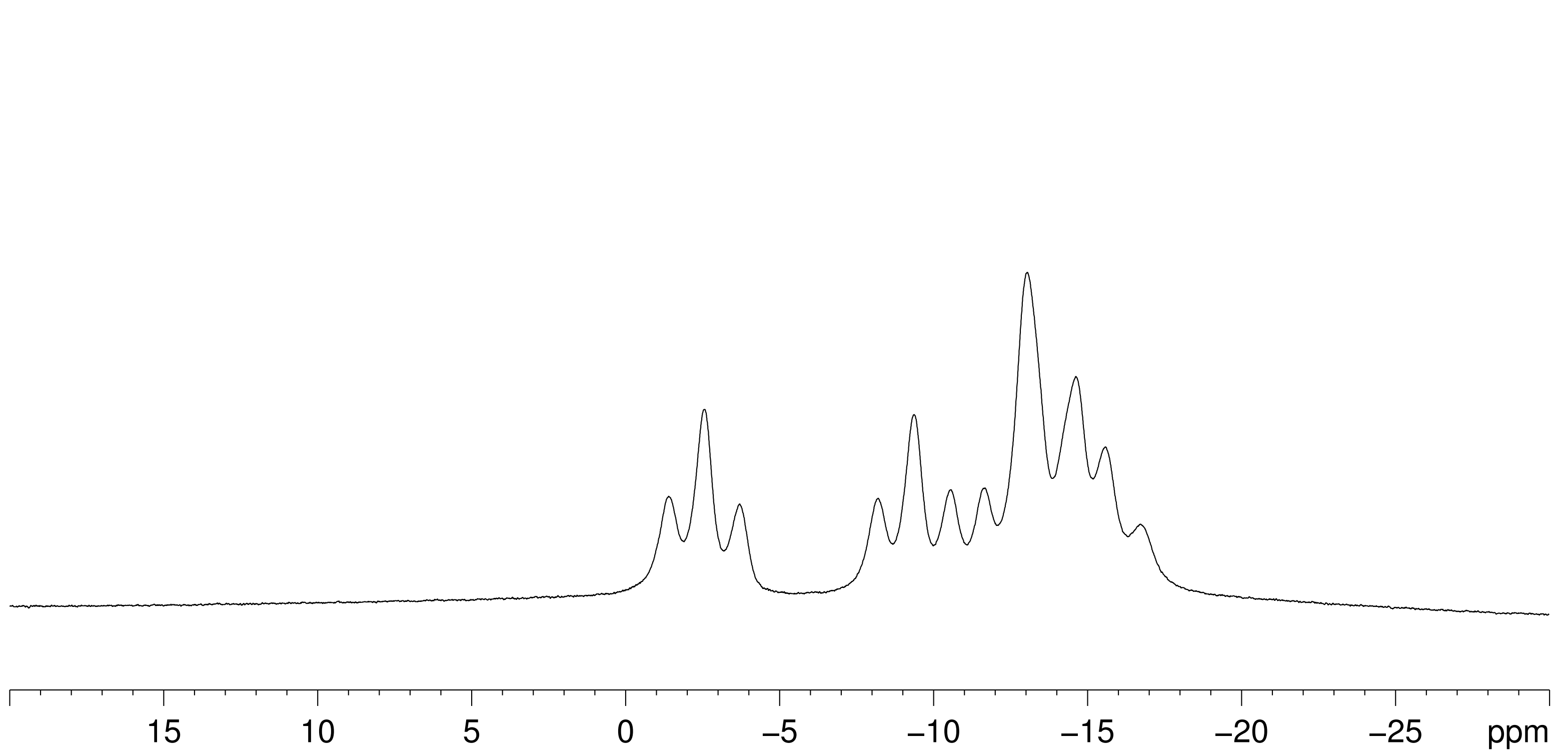

qyj-B-0363-CD2Cl2(C

NAME	qyj-B-0363-CD2Cl2(C)	
EXPNO	1	
PROCNO	1	
Date_	20141105	
Time	15.55	
INSTRUM	spect	
PROBHD	5 mm PABBO BB-	
PULPROG	zg30	
TD	65536	
SOLVENT	C6D6	
NS	16	
DS	0	
SWH	25510.203	Hz
FIDRES	0.389255	Hz
AQ	1.2845556	sec
RG	362	
DW	19.600	usec
DE	6.50	usec
TE	298.9	K
D1	5.00000000	sec
TD0	1	

======== CHANNEL f1 ========

NUC1	11B	
P1	7.60	usec
PL1	-3.00	dB
PL1W	55.13059616	W
SFO1	128.3968556	MHz
SI	32768	
SF	128.3968865	MHz
WDW	EM	
SSB	0	
LB	3.00	Hz
GB	0	
PC	1.40	

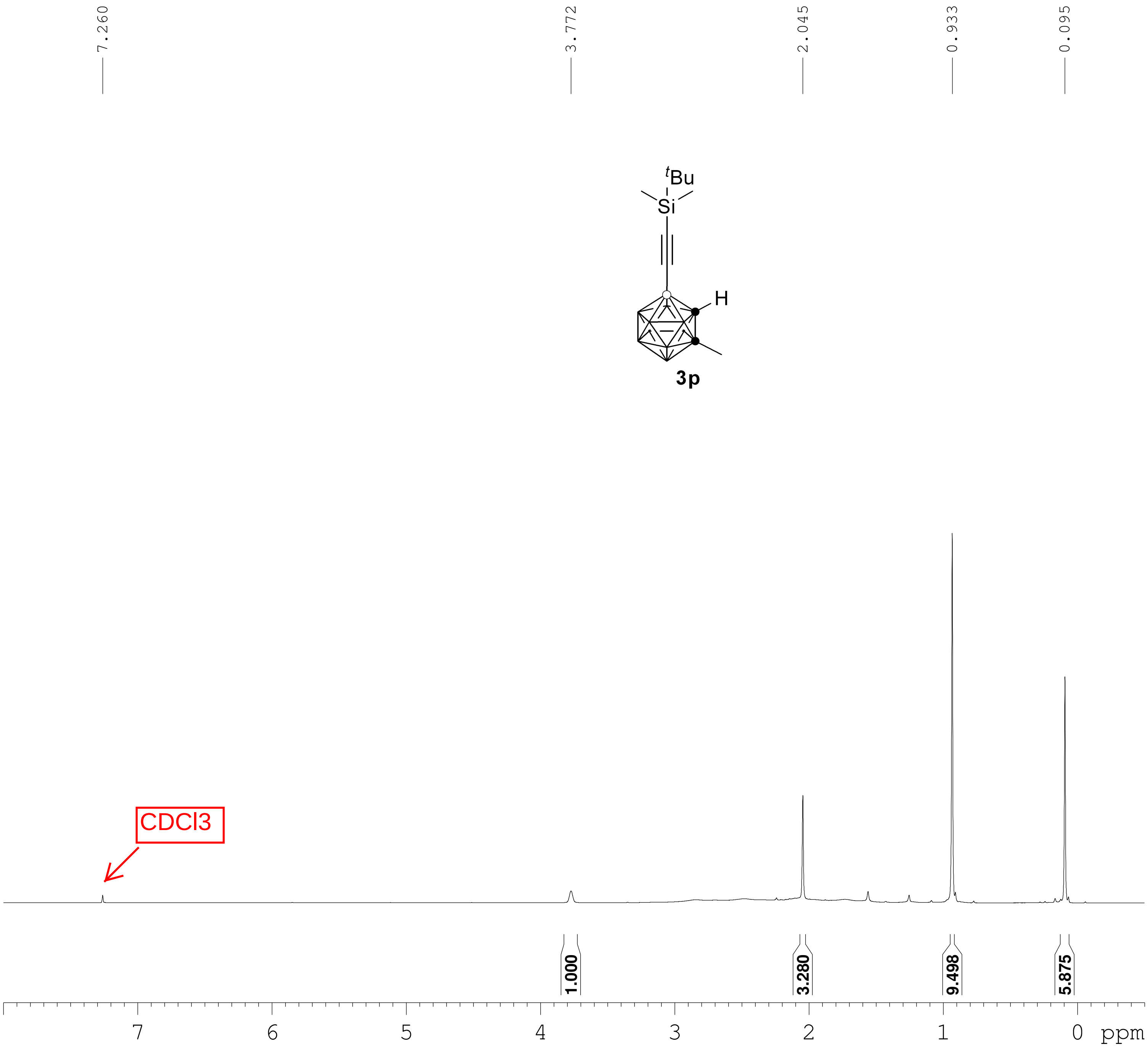

qyj-H-0386-CDCl3

Bruker Advance III 400

NAME	qyj-H-0386-CDCl3
EXPNO	1
PROCNO	1
Date_	20141229
Time	16.02
INSTRUM	spect
PROBHD	5 mm PADUL 13C
PULPROG	zg
TD	65536
SOLVENT	CDCl3
NS	9
DS	0
SWH	10000.000 Hz
FIDRES	0.152588 Hz
AQ	3.2768500 sec
RG	71.8
DW	50.000 usec
DE	6.50 usec
TE	295.3 K
D1	1.00000000 sec
TD0	1

======== CHANNEL f1 ========

NUC1	1H
P1	14.83 usec
PL1	0.00 dB
PL1W	8.31434441 W
SFO1	400.1318000 MHz
SI	65536
SF	400.1300080 MHz
WDW	EM
SSB	0
LB	0.30 Hz
GB	0
PC	1.00

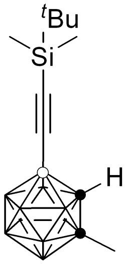

3p

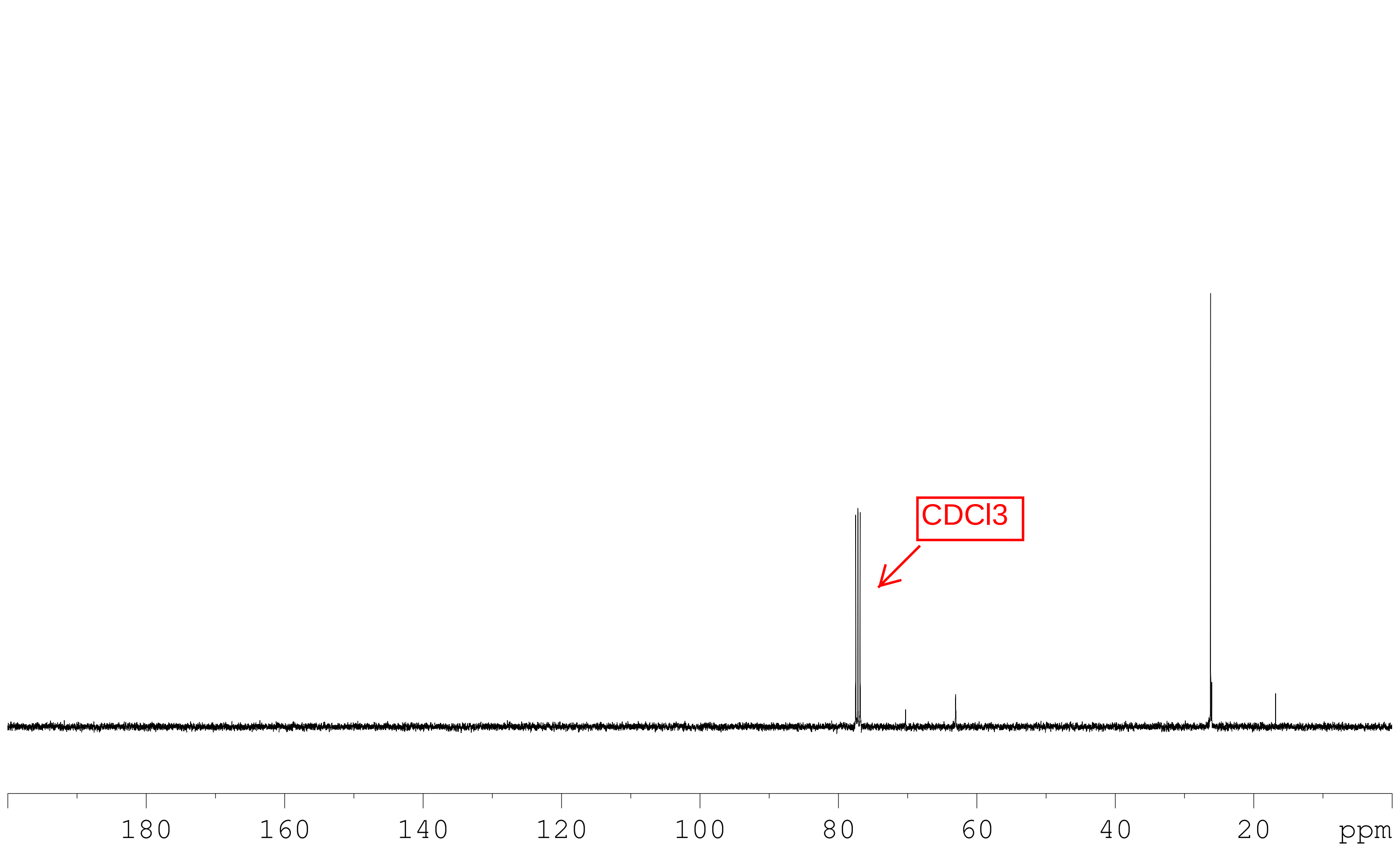

qyj-C-0386-CDCl3

Bruker Advance III 400

NAME	qyj-C-0386-CDCl3
EXPNO	1
PROCNO	1
Date_	20141229
Time	16.05
INSTRUM	spect
PROBHD	5 mm PADUL 13C
PULPROG	zgdc
TD	131072
SOLVENT	CDCl3
NS	200
DS	0
SWH	29761.904 Hz
FIDRES	0.227065 Hz
AQ	2.2020595 sec
RG	203
DW	16.800 usec
DE	6.50 usec
TE	295.6 K
D1	1.00000000 sec
D11	0.03000000 sec
TD0	1

======== CHANNEL f1 ========

NUC1	13C
P1	9.68 usec
PL1	-0.60 dB
PL1W	41.24164963 W
SFO1	100.6227690 MHz

======== CHANNEL f2 ========

CPDPRG2	waltz16
NUC2	1H
PCPD2	90.00 usec
PL2	0.00 dB
PL12	15.66 dB
PL2W	8.31434441 W
PL12W	0.22585411 W
SFO2	400.1320007 MHz
SI	131072
SF	100.6127547 MHz
WDW	EM
SSB	0
LB	1.00 Hz
GB	0
PC	1.40

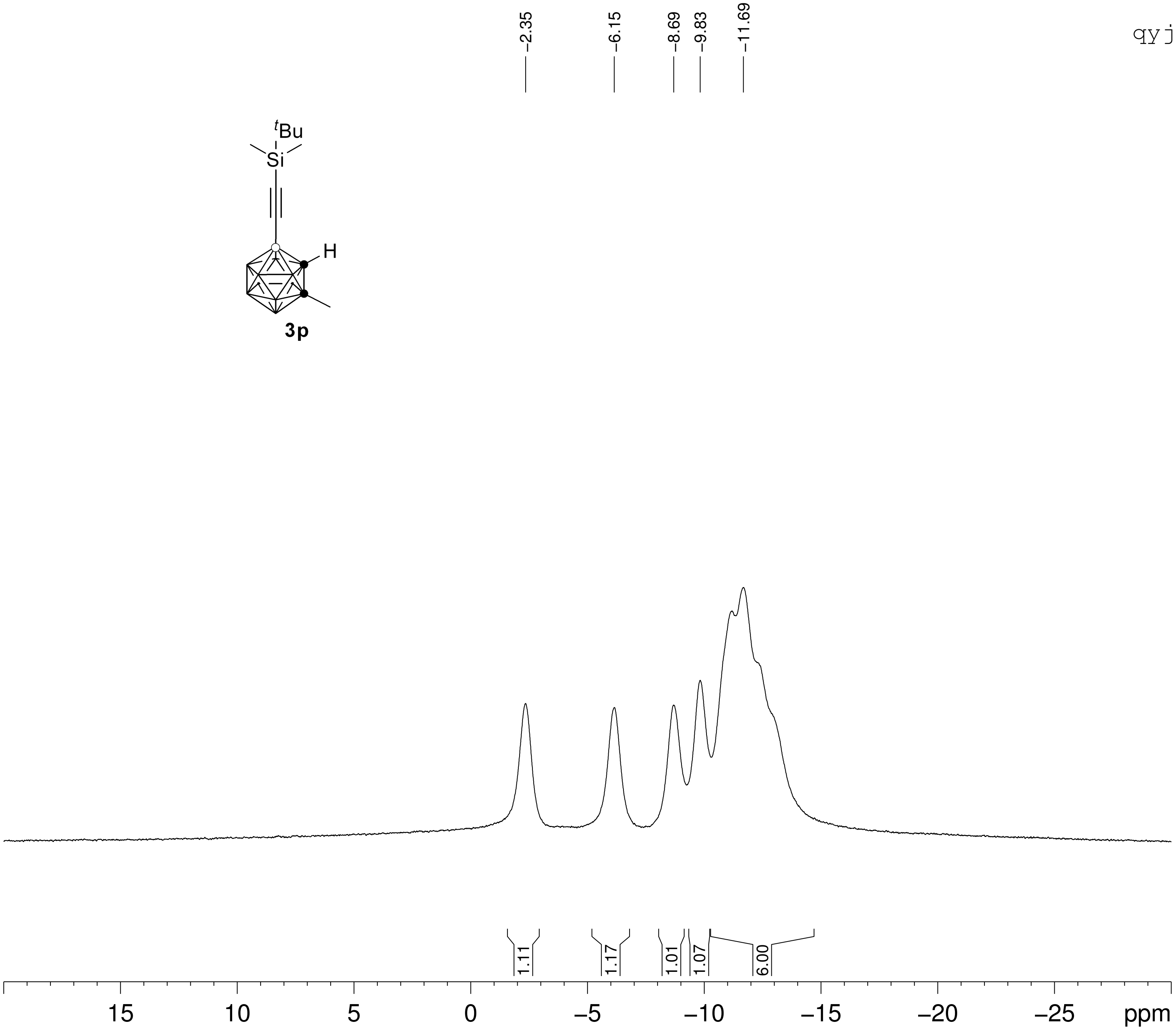

qyj-B-0386-CDCl3

```
NAME       qyj-B-0386-CDCl3
EXPNO                     1
PROCNO                    1
Date_              20141229
Time                  16.24
INSTRUM               spect
PROBHD      5 mm PABBO BB-
PULPROG                zgdc
TD                    65536
SOLVENT                C6D6
NS                        8
DS                        0
SWH               25510.203 Hz
FIDRES             0.389255 Hz
AQ                1.2845556 sec
RG                      287
DW                   19.600 usec
DE                     6.50 usec
TE                    298.9 K
D1               5.00000000 sec
D11              0.03000000 sec
TD0                       1

======== CHANNEL f1 ========
NUC1                    11B
P1                     7.60 usec
PL1                   -3.00 dB
PL1W            55.13059616 W
SFO1            128.3968556 MHz

======== CHANNEL f2 ========
CPDPRG2             waltz16
NUC2                     1H
PCPD2                 90.00 usec
PL2                   -1.00 dB
PL12                  15.16 dB
PL2W            13.56617069 W
PL12W            0.32844096 W
SFO2            400.1916008 MHz
SI                    32768
SF              128.3968847 MHz
WDW                      EM
SSB                       0
LB                     3.00 Hz
GB                        0
PC                     1.40
```

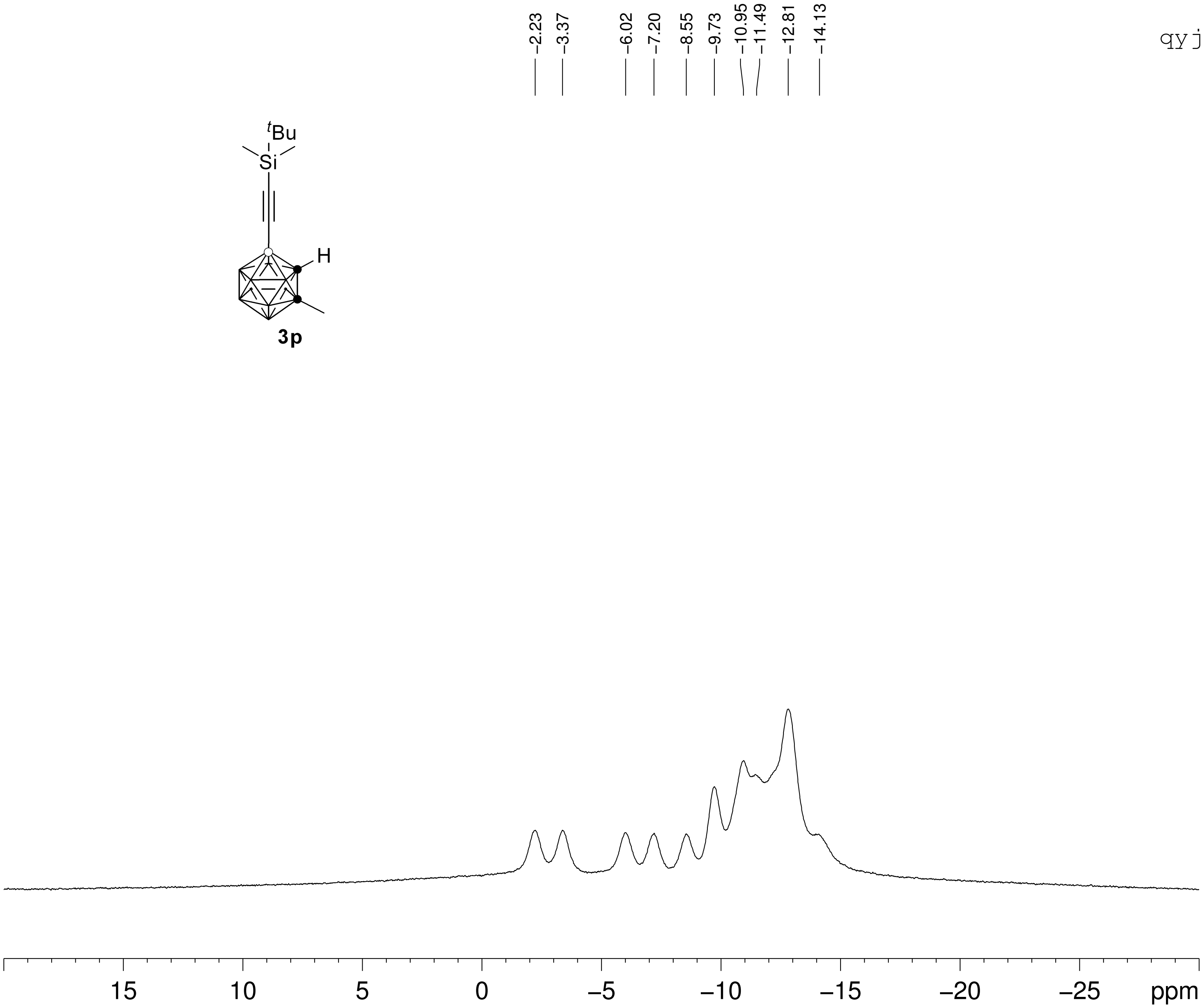

qyj-B-0386-CDCl3(C)

NAME	qyj-B-0386-CDCl3(C)
EXPNO	1
PROCNO	1
Date_	20141229
Time	16.26
INSTRUM	spect
PROBHD	5 mm PABBO BB-
PULPROG	zg30
TD	65536
SOLVENT	C6D6
NS	16
DS	0
SWH	38461.539 Hz
FIDRES	0.586877 Hz
AQ	0.8520180 sec
RG	406
DW	13.000 usec
DE	6.50 usec
TE	298.9 K
D1	5.00000000 sec
TD0	1

======== CHANNEL f1 ========

NUC1	11B
P1	7.60 usec
PL1	-3.00 dB
PL1W	55.13059616 W
SFO1	128.3968556 MHz
SI	32768
SF	128.3969425 MHz
WDW	EM
SSB	0
LB	3.00 Hz
GB	0
PC	1.40

qyj-H-6-2-CDCl3

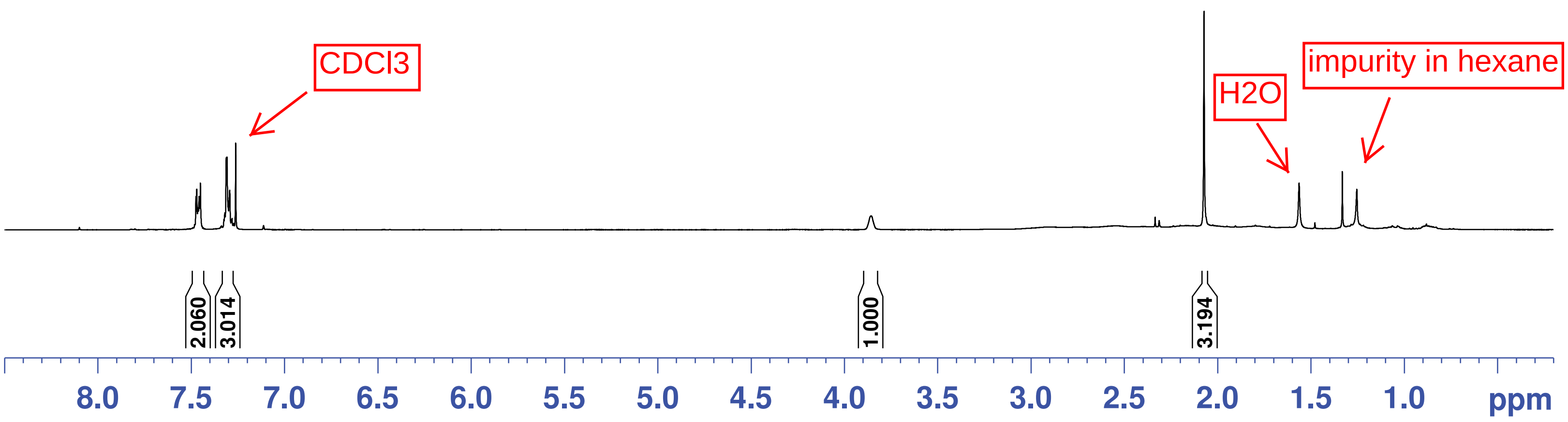

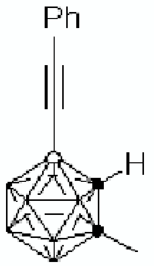

3r

```
Current Data Parameters
NAME      qyj-H-6-2-CDCl3
EXPNO                    1
PROCNO                   1

F2 - Acquisition Parameters
Date_             20150614
Time                 11.50 h
INSTRUM              spect
PROBHD     Z824601_0021 (
PULPROG               zg30
TD                   65536
SOLVENT              CDCl3
NS                      16
DS                       2
SWH               8012.820 Hz
FIDRES            0.122266 Hz
AQ               4.0894465 sec
RG                     203
DW                  62.400 usec
DE                    6.50 usec
TE                   294.9 K
D1              1.00000000 sec
TD0                      1
SFO1           400.1324708 MHz
NUC1                    1H
P1                   15.00 usec
PLW1            8.31000042 W

F2 - Processing parameters
SI                   65536
SF             400.1300099 MHz
WDW                     EM
SSB       0
LB                    0.30 Hz
GB        0
PC                    1.00
```

qyj-C-6-2-CDCl3

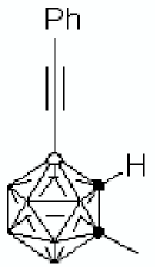

3r

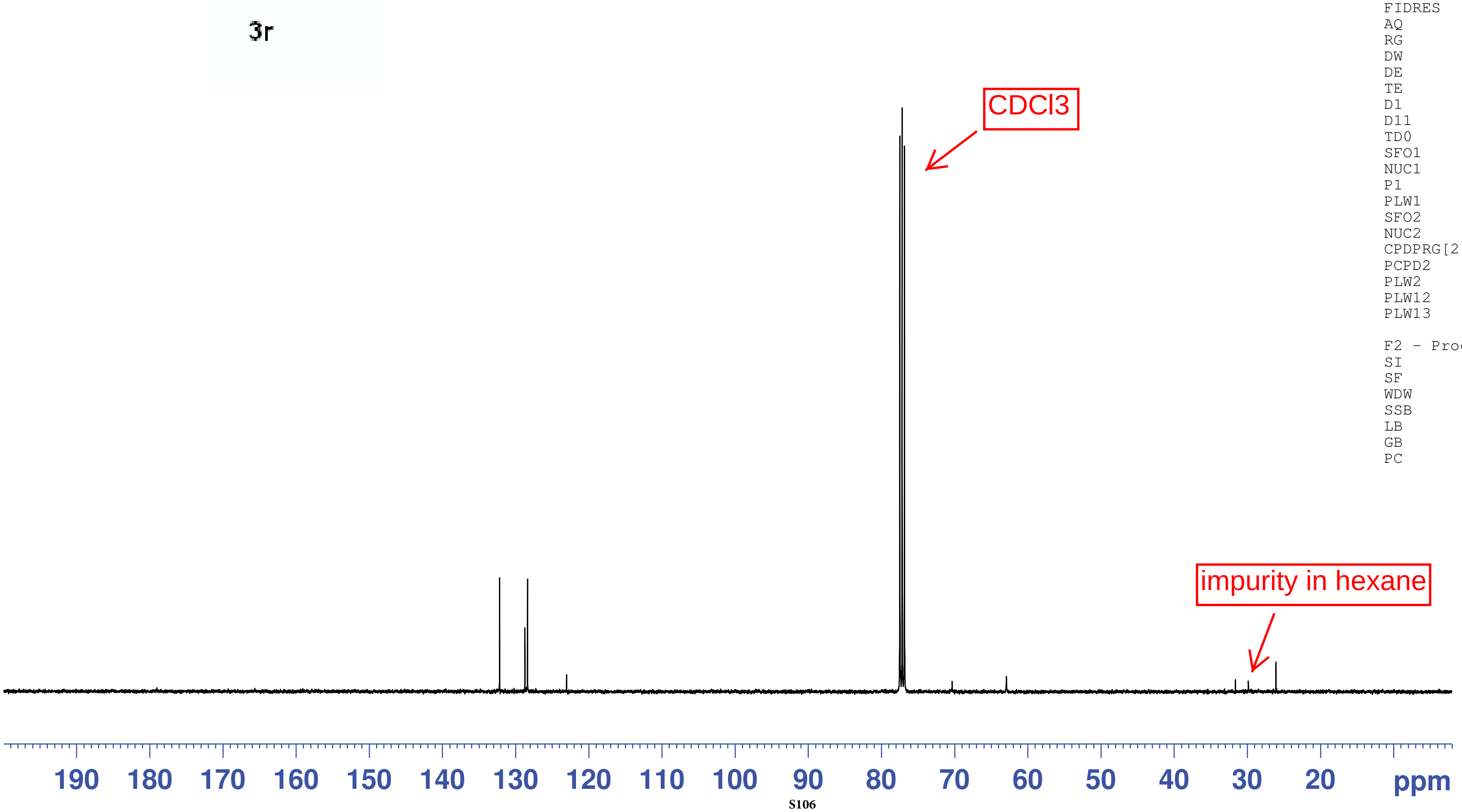


```
Current Data Parameters
NAME      qyj-C-6-2-CDCl3
EXPNO                   1
PROCNO                  1

F2 - Acquisition Parameters
Date_            20150614
Time                12.13 h
INSTRUM             spect
PROBHD    Z824601_0021 (
PULPROG           zgpg30
TD                 65536
SOLVENT            CDCl3
NS                  1655
DS                     4
SWH            24038.461 Hz
FIDRES          0.366798 Hz
AQ             1.3631488 sec
RG                   203
DW                20.800 usec
DE                  6.50 usec
TE                 295.3 K
D1            2.00000000 sec
D11           0.03000000 sec
TD0                    1
SFO1         100.6228298 MHz
NUC1                 13C
P1                  9.50 usec
PLW1         41.25000000 W
SFO2         400.1316005 MHz
NUC2                  1H
CPDPRG[2         waltz16
PCPD2              90.00 usec
PLW2          8.31000042 W
PLW12         0.23083000 W
PLW13         0.11611000 W

F2 - Processing parameters
SI                 32768
SF           100.6127557 MHz
WDW                   EM
SSB       0
LB                  1.00 Hz
GB        0
PC                  1.40
```

qyj-B-6-2-CDCl3

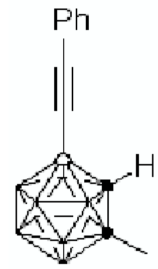

3r

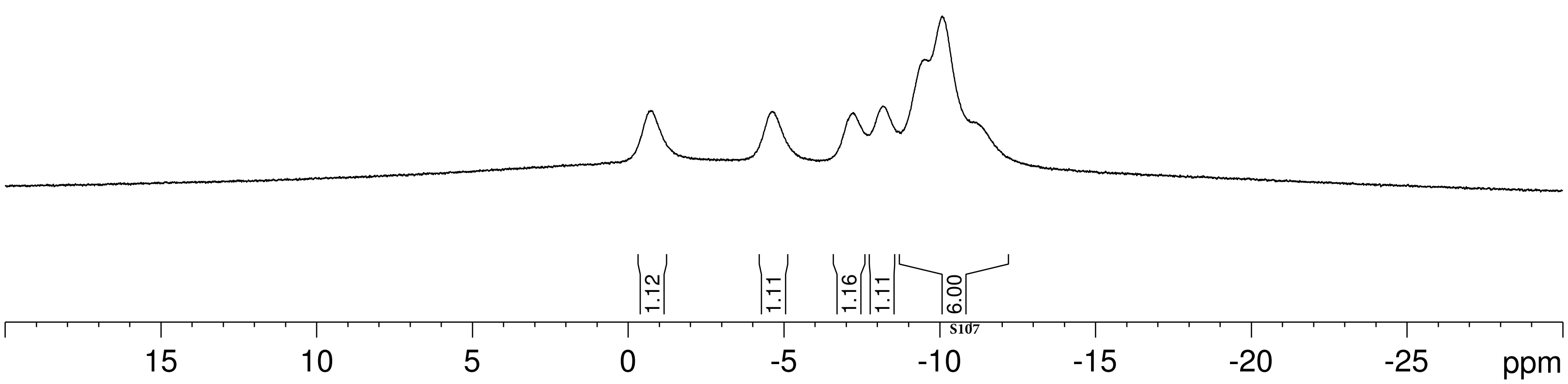

Current Data Parameters
NAME qyj-B-6-2-CDCl3
EXPNO 1
PROCNO 1

F2 - Acquisition Parameters
Date_ 20150622
Time 10.25 h
INSTRUM spect
PROBHD Z108618_0257 (
PULPROG zgpg30
TD 65536
SOLVENT C6D6
NS 40
DS 4
SWH 29761.904 Hz
FIDRES 0.454131 Hz
AQ 1.1010048 sec
RG 512
DW 16.800 usec
DE 6.50 usec
TE 296.8 K
D1 2.00000000 sec
D11 0.03000000 sec
TD0 1
SFO1 128.4096890 MHz
NUC1 11B
P1 7.50 usec
PLW1 55.09999847 W
SFO2 400.2316008 MHz
NUC2 1H
CPDPRG[2 waltz16
PCPD2 90.00 usec
PLW2 13.56000042 W
PLW12 0.27428001 W
PLW13 0.13796000 W

F2 - Processing parameters
SI 32768
SF 128.4095347 MHz
WDW EM
SSB 0
LB 1.00 Hz
GB 0
PC 1.40

qyj-B-6-2-CDCl3(C)

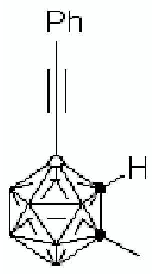

3r

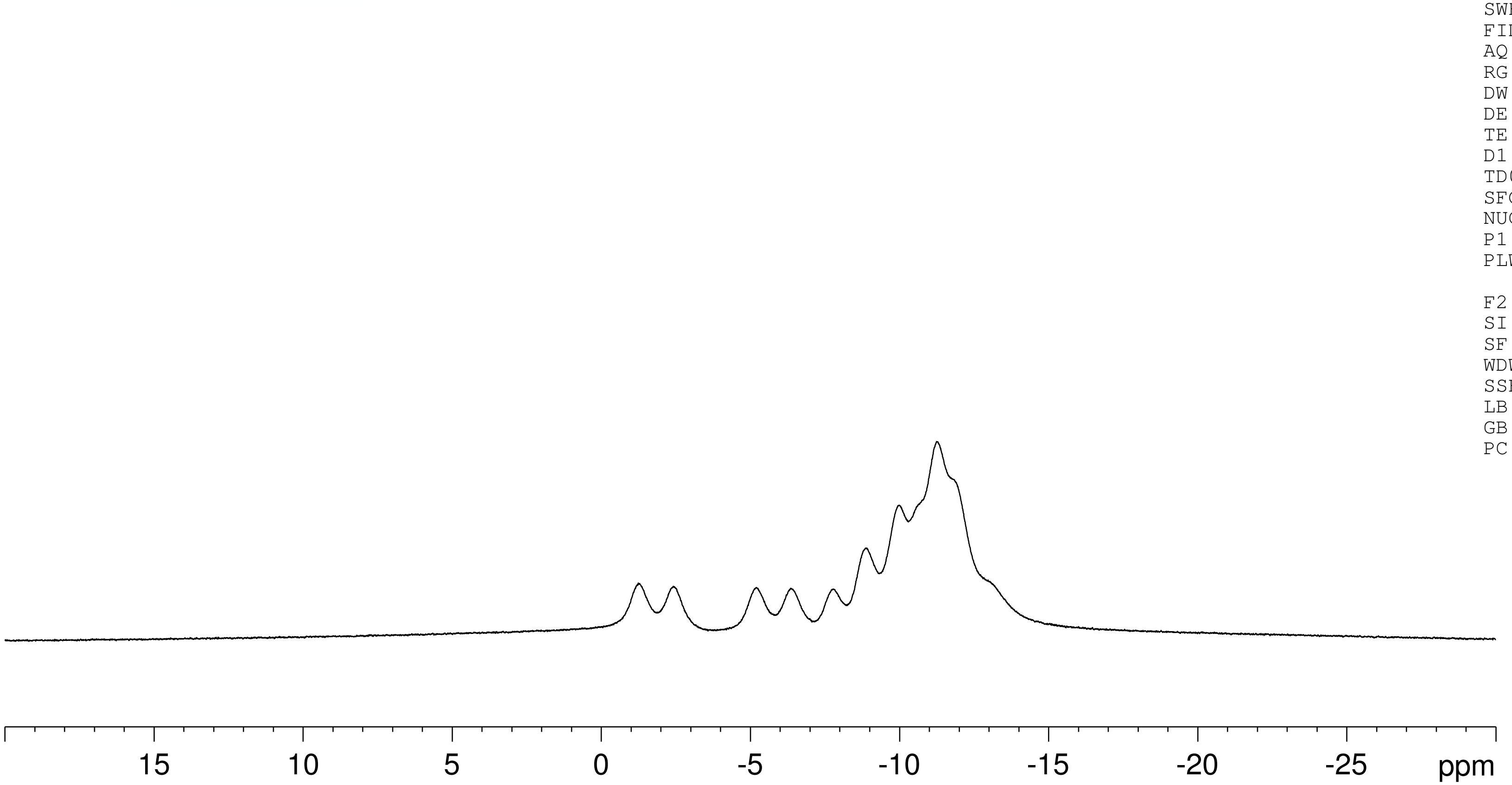

Current Data Parameters
NAME qyj-B-6-2-CDCl3(C)
EXPNO 1
PROCNO 1

F2 - Acquisition Parameters
Date_ 20150622
Time 10.28 h
INSTRUM spect
PROBHD Z108618_0257 (
PULPROG zg
TD 65536
SOLVENT C6D6
NS 44
DS 4
SWH 25510.203 Hz
FIDRES 0.389255 Hz
AQ 1.2845056 sec
RG 322
DW 19.600 usec
DE 6.50 usec
TE 296.4 K
D1 1.00000000 sec
TD0 1
SFO1 128.4096891 MHz
NUC1 11B
P1 7.50 usec
PLW1 55.09999847 W

F2 - Processing parameters
SI 32768
SF 128.4096891 MHz
WDW EM
SSB 0
LB 1.00 Hz
GB 0
PC 1.40

qyj-H-A3-CDCl3

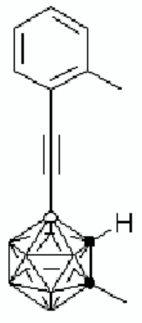

3s

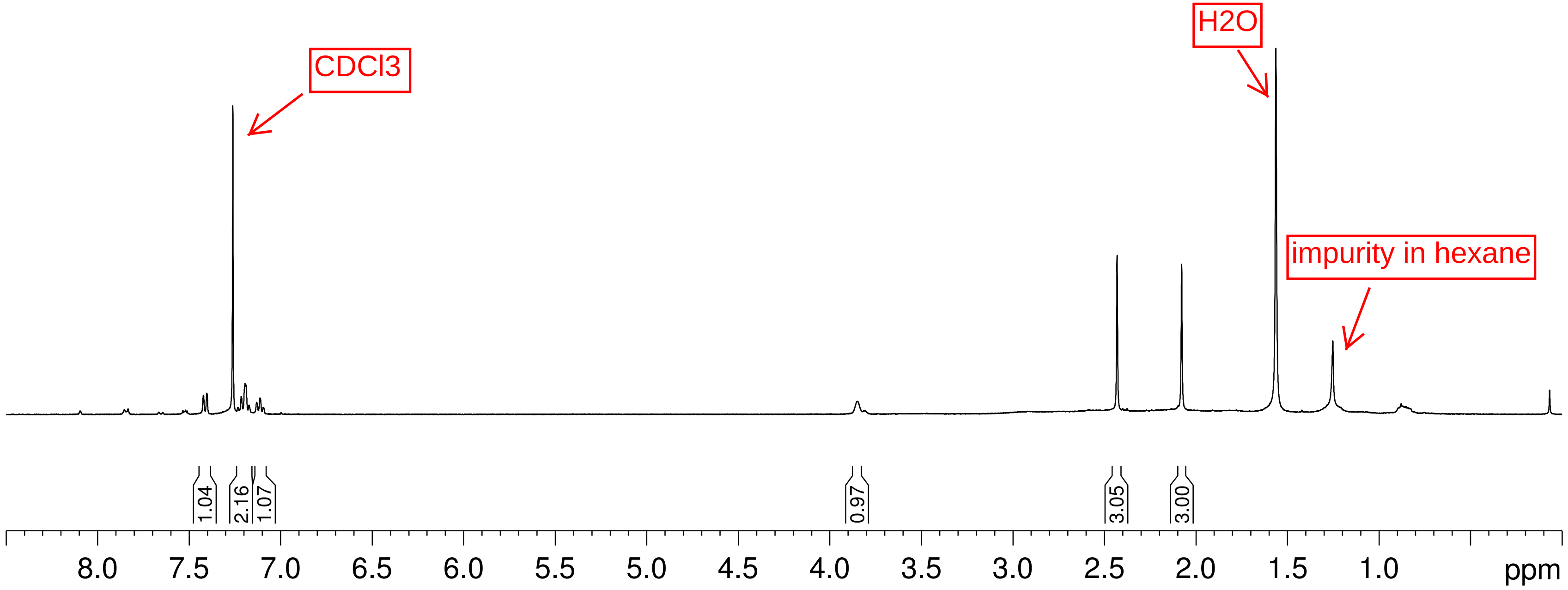

Current Data Parameters
NAME qyj-H-A3-CDCl3
EXPNO 2
PROCNO 1

F2 - Acquisition Parameters
Date_ 20151228
Time 17.42 h
INSTRUM spect
PROBHD Z108618_0257 (
PULPROG zg30
TD 65536
SOLVENT CDCl3
NS 14
DS 2
SWH 8012.820 Hz
FIDRES 0.122266 Hz
AQ 4.0894465 sec
RG 161
DW 62.400 usec
DE 6.50 usec
TE 295.3 K
D1 1.00000000 sec
TD0 1
SFO1 400.2324714 MHz
NUC1 1H
P1 12.80 usec
PLW1 13.56000042 W

F2 - Processing parameters
SI 65536
SF 400.2300107 MHz
WDW EM
SSB 0
LB 0.30 Hz
GB 0
PC 1.00

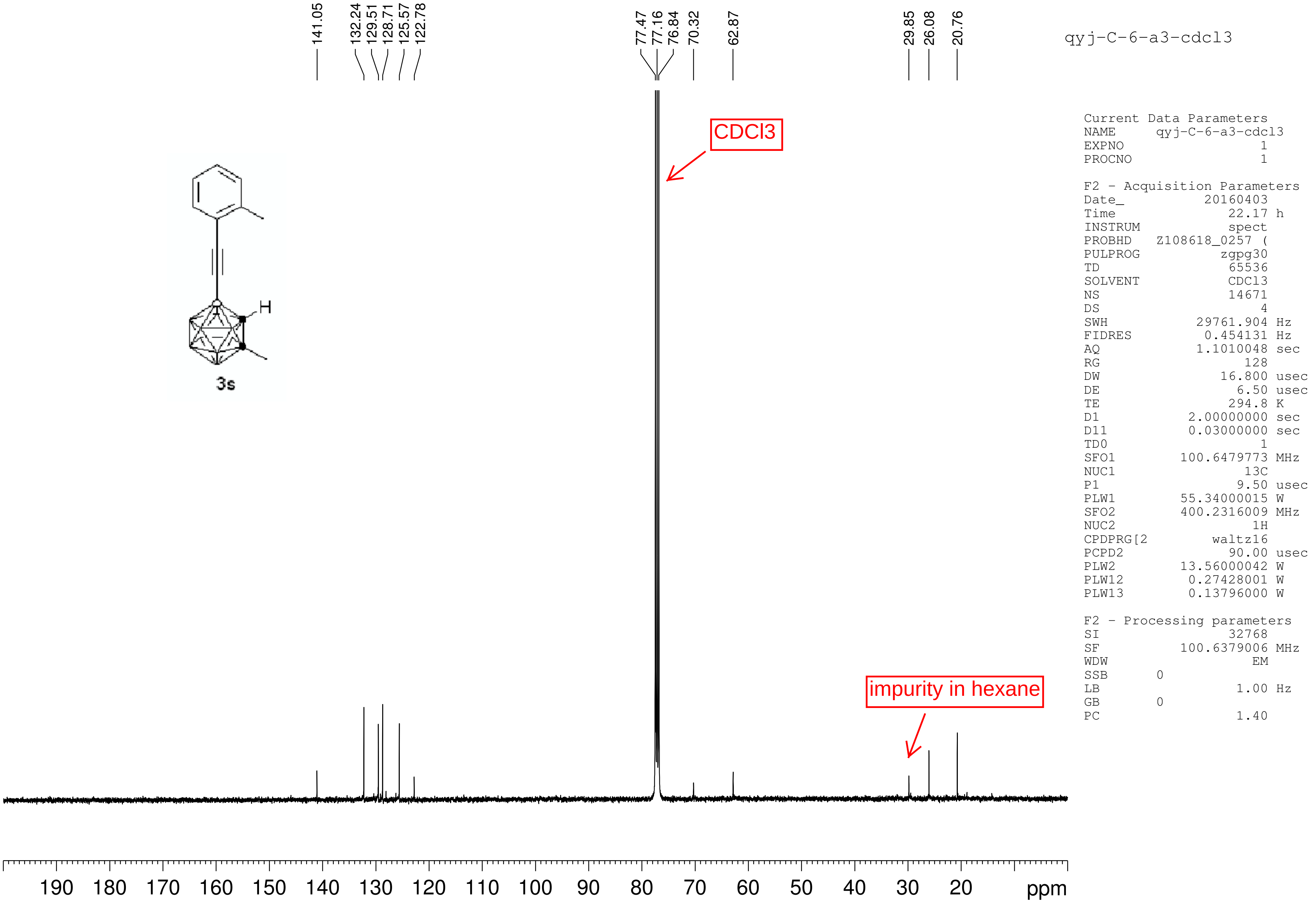
141.05
132.24
129.51
128.71
125.57
122.78
77.47
77.16
76.84
70.32
62.87
29.85
26.08
20.76
qyj-C-6-a3-cdcl3
CDCl3
impurity in hexane
H
3s
190 180 170 160 150 140 130 120 110 100 90 80 70 60 50 40 30 20 ppm
Current Data Parameters
NAME qyj-C-6-a3-cdcl3
EXPNO 1
PROCNO 1
F2 - Acquisition Parameters
Date_ 20160403
Time 22.17 h
INSTRUM spect
PROBHD Z108618_0257 (
PULPROG zgpg30
TD 65536
SOLVENT CDCl3
NS 14671
DS 4
SWH 29761.904 Hz
FIDRES 0.454131 Hz
AQ 1.1010048 sec
RG 128
DW 16.800 usec
DE 6.50 usec
TE 294.8 K
D1 2.00000000 sec
D11 0.03000000 sec
TD0 1
SFO1 100.6479773 MHz
NUC1 13C
P1 9.50 usec
PLW1 55.34000015 W
SFO2 400.2316009 MHz
NUC2 1H
CPDPRG[2 waltz16
PCPD2 90.00 usec
PLW2 13.56000042 W
PLW12 0.27428001 W
PLW13 0.13796000 W
F2 - Processing parameters
SI 32768
SF 100.6379006 MHz
WDW EM
SSB 0
LB 1.00 Hz
GB 0
PC 1.40

qyj-B-A3-CDCl3

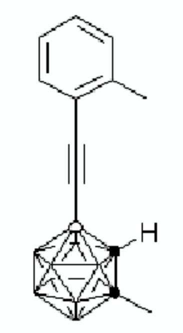

3s

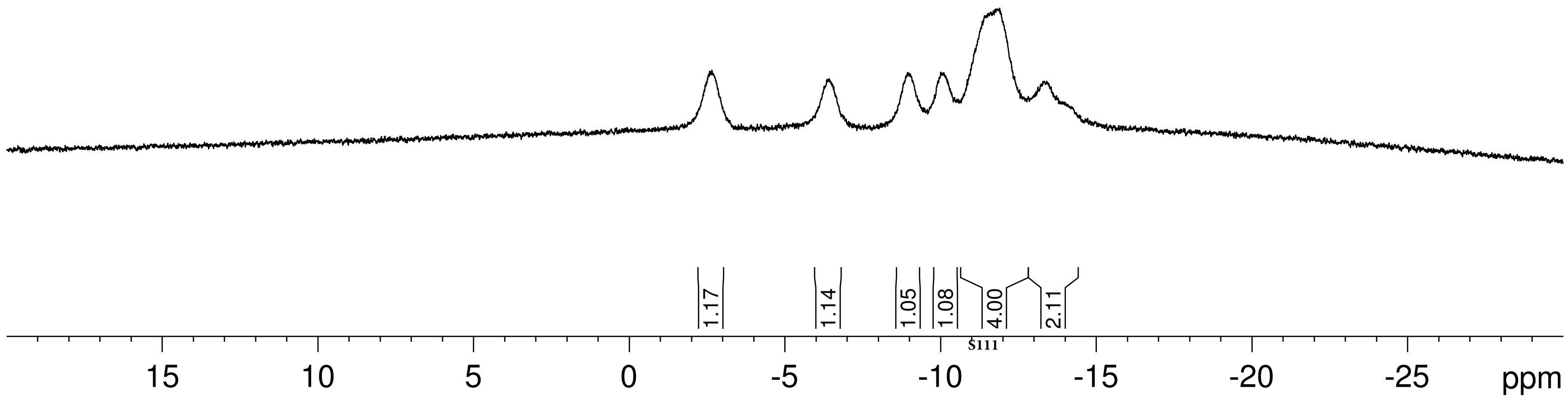

Current Data Parameters
NAME qyj-B-A3-CDCl3
EXPNO 1
PROCNO 1

F2 - Acquisition Parameters
Date_ 20151228
Time 17.46 h
INSTRUM spect
PROBHD Z108618_0257 (
PULPROG zgdc
TD 65536
SOLVENT CDCl3
NS 48
DS 4
SWH 24038.461 Hz
FIDRES 0.366798 Hz
AQ 1.3631488 sec
RG 456
DW 20.800 usec
DE 6.50 usec
TE 295.8 K
D1 2.00000000 sec
D11 0.03000000 sec
TD0 1
SFO1 128.4096890 MHz
NUC1 11B
P1 7.50 usec
PLW1 55.09999847 W
SFO2 400.2316009 MHz
NUC2 1H
CPDPRG[2 waltz16
PCPD2 90.00 usec
PLW2 13.56000042 W
PLW12 0.27428001 W

F2 - Processing parameters
SI 32768
SF 128.4097615 MHz
WDW EM
SSB 0
LB 1.00 Hz
GB 0
PC 1.40

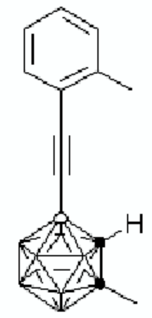

3s

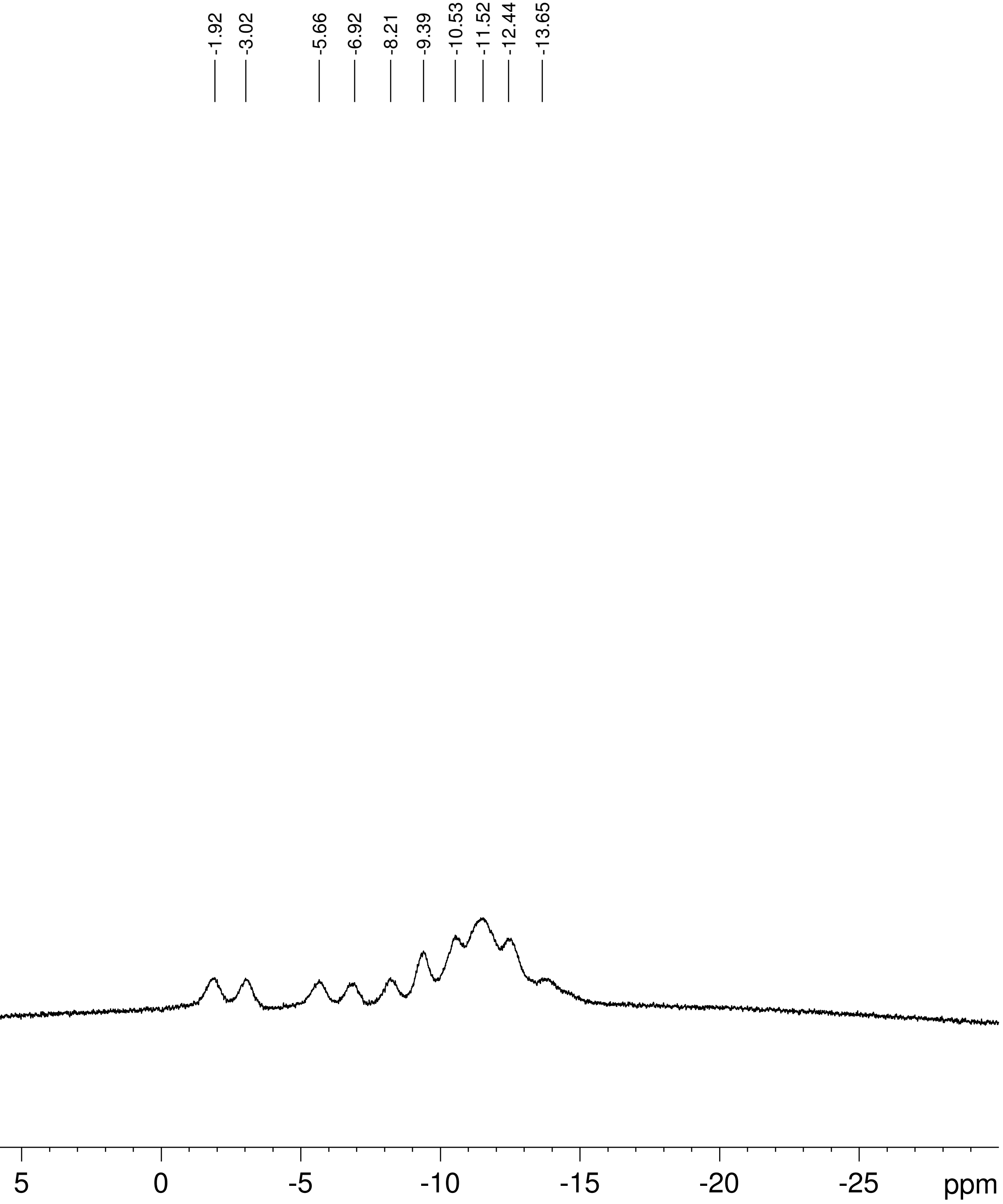

qyj-B-A3-CDCl3(C)

```
Current Data Parameters
NAME     qyj-B-A3-CDCl3(C)
EXPNO                    1
PROCNO                   1

F2 - Acquisition Parameters
Date_            20151228
Time                17.50 h
INSTRUM             spect
PROBHD   Z108618_0257 (
PULPROG                zg
TD                  65536
SOLVENT             CDCl3
NS                     60
DS                      2
SWH             24038.461 Hz
FIDRES           0.366798 Hz
AQ              1.3631488 sec
RG                    512
DW                 20.800 usec
DE                   6.50 usec
TE                  295.3 K
D1             2.00000000 sec
TD0                     1
SFO1          128.4096890 MHz
NUC1                  11B
P1                   7.50 usec
PLW1          55.09999847 W

F2 - Processing parameters
SI                  32768
SF            128.4097430 MHz
WDW                    EM
SSB      0
LB                   1.00 Hz
GB       0
PC                   1.40
```

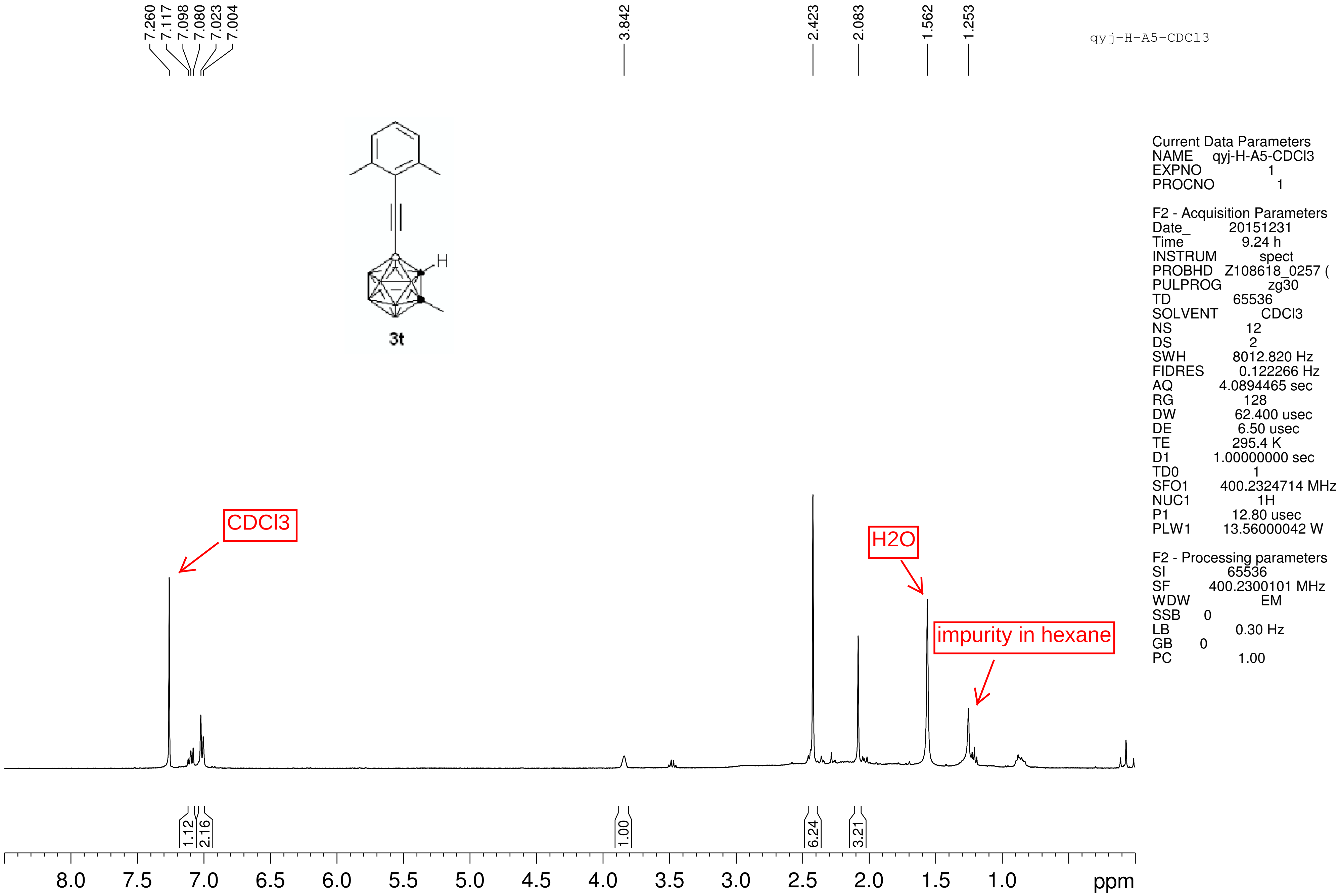

7.260
7.117
7.098
7.080
7.023
7.004
3.842
2.423
2.083
1.562
1.253
qyj-H-A5-CDCl3
H
3t
CDCl3
H2O
impurity in hexane
1.12
2.16
1.00
6.24
3.21
8.0 7.5 7.0 6.5 6.0 5.5 5.0 4.5 4.0 3.5 3.0 2.5 2.0 1.5 1.0 ppm
Current Data Parameters
NAME qyj-H-A5-CDCl3
EXPNO 1
PROCNO 1
F2 - Acquisition Parameters
Date_ 20151231
Time 9.24 h
INSTRUM spect
PROBHD Z108618_0257 (
PULPROG zg30
TD 65536
SOLVENT CDCl3
NS 12
DS 2
SWH 8012.820 Hz
FIDRES 0.122266 Hz
AQ 4.0894465 sec
RG 128
DW 62.400 usec
DE 6.50 usec
TE 295.4 K
D1 1.00000000 sec
TD0 1
SFO1 400.2324714 MHz
NUC1 1H
P1 12.80 usec
PLW1 13.56000042 W
F2 - Processing parameters
SI 65536
SF 400.2300101 MHz
WDW EM
SSB 0
LB 0.30 Hz
GB 0
PC 1.00

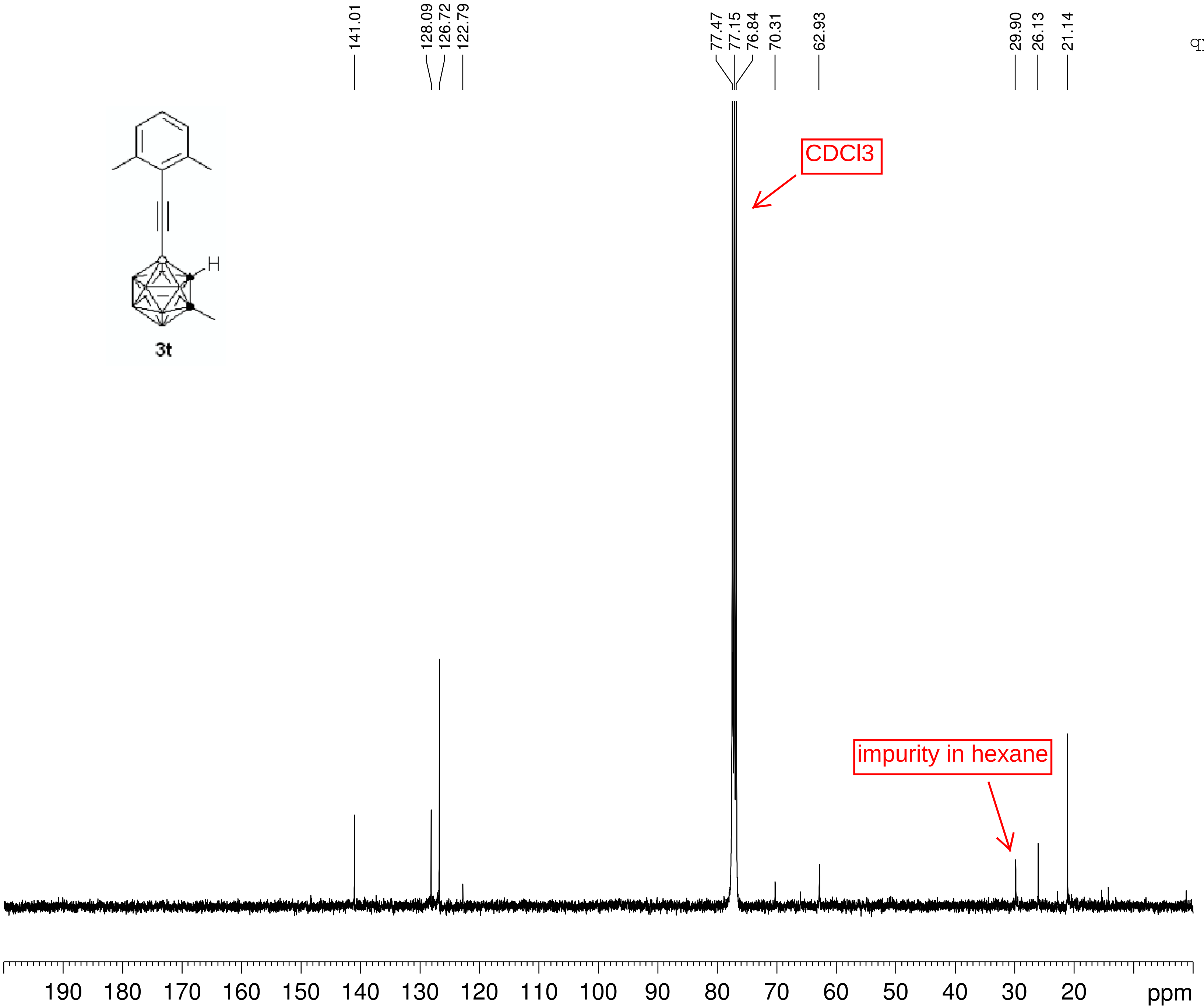

qyj-C-A5-CDCl3

```
Current Data Parameters
NAME       qyj-C-A5-CDCl3
EXPNO                   1
PROCNO                  1

F2 - Acquisition Parameters
Date_            20151231
Time                20.59 h
INSTRUM             spect
PROBHD     Z108618_0257 (
PULPROG           zgpg30
TD                 65536
SOLVENT            CDCl3
NS                 16207
DS                     4
SWH            29761.904 Hz
FIDRES          0.454131 Hz
AQ             1.1010048 sec
RG                   203
DW                16.800 usec
DE                  6.50 usec
TE                 295.2 K
D1            2.00000000 sec
D11           0.03000000 sec
TD0                    1
SFO1         100.6479773 MHz
NUC1                 13C
P1                  9.50 usec
PLW1         55.34000015 W
SFO2         400.2316009 MHz
NUC2                  1H
CPDPRG[2         waltz16
PCPD2              90.00 usec
PLW2         13.56000042 W
PLW12         0.27428001 W
PLW13         0.13796000 W

F2 - Processing parameters
SI                 32768
SF           100.6379006 MHz
WDW                   EM
SSB       0
LB                  1.00 Hz
GB        0
PC                  1.40
```

qyj-B-A5-CDCl3

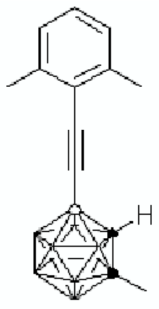

3t

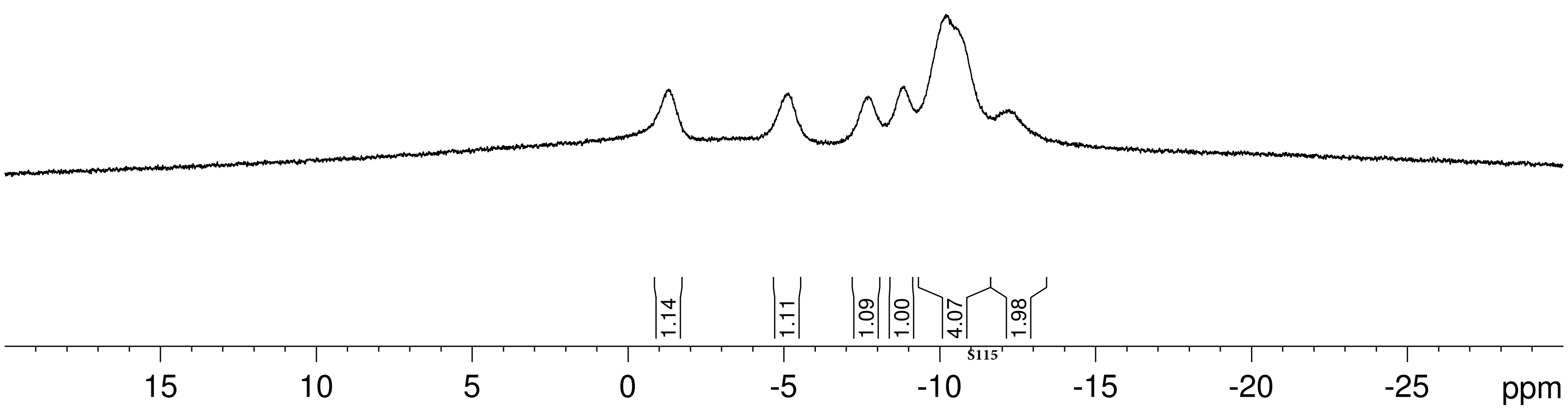

Current Data Parameters
NAME qyj-B-A5-CDCl3
EXPNO 1
PROCNO 1

F2 - Acquisition Parameters
Date_ 20151230
Time 19.25 h
INSTRUM spect
PROBHD Z108618_0257 (
PULPROG zgdc
TD 65536
SOLVENT CDCl3
NS 32
DS 4
SWH 24038.461 Hz
FIDRES 0.366798 Hz
AQ 1.3631488 sec
RG 456
DW 20.800 usec
DE 6.50 usec
TE 295.6 K
D1 2.00000000 sec
D11 0.03000000 sec
TD0 1
SFO1 128.4096890 MHz
NUC1 11B
P1 7.50 usec
PLW1 55.09999847 W
SFO2 400.2316009 MHz
NUC2 1H
CPDPRG[2 waltz16
PCPD2 90.00 usec
PLW2 13.56000042 W
PLW12 0.27428001 W

F2 - Processing parameters
SI 32768
SF 128.4097615 MHz
WDW EM
SSB 0
LB 1.00 Hz
GB 0
PC 1.40

qyj-B-A5-CDCl3(C)

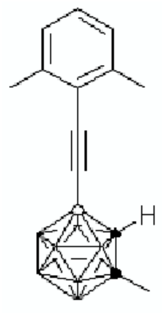

3t

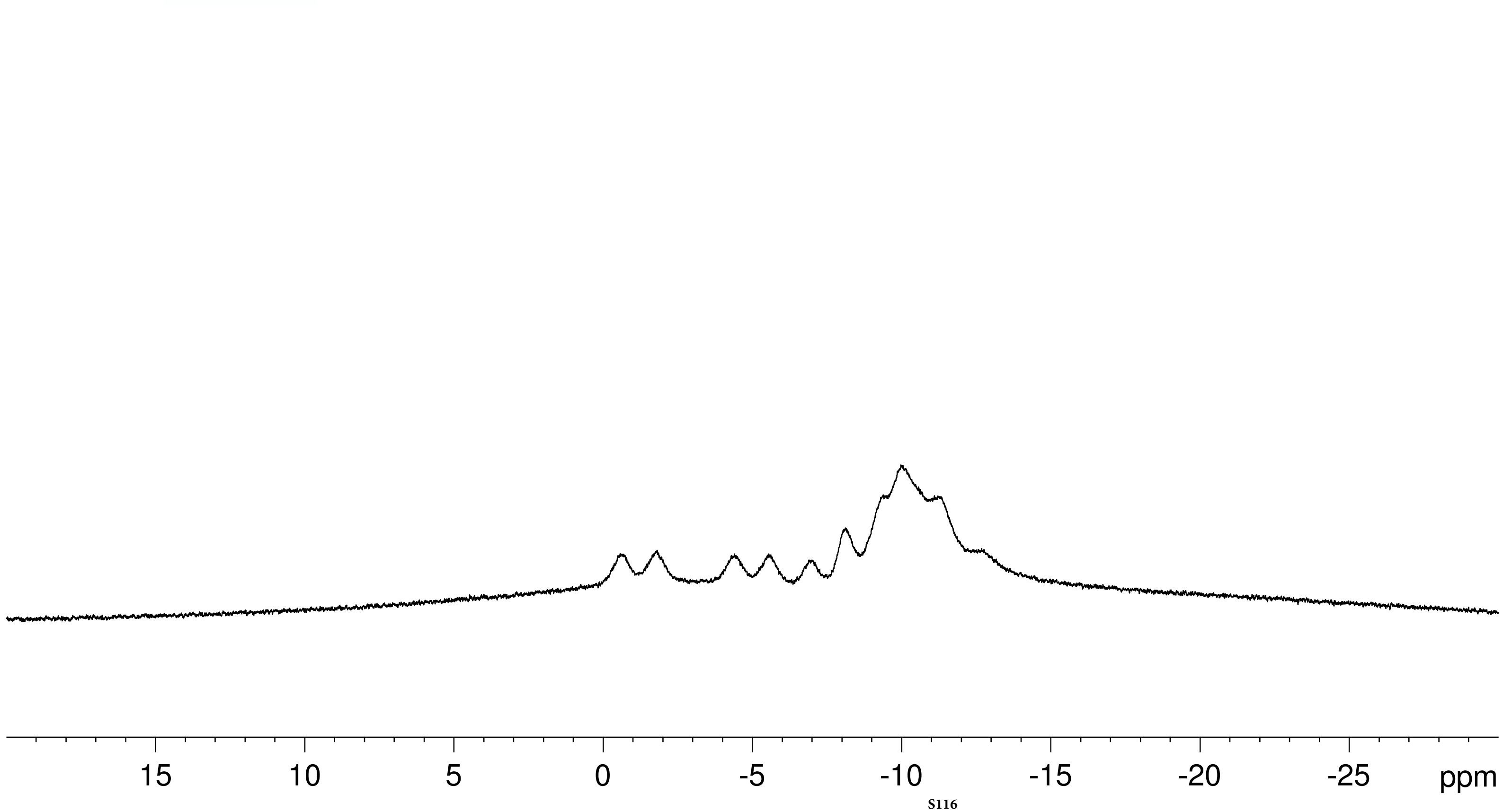


```
Current Data Parameters
NAME      qyj-B-A5-CDCl3(C)
EXPNO                     1
PROCNO                    1

F2 - Acquisition Parameters
Date_            20151230
Time                19.30 h
INSTRUM             spect
PROBHD   Z108618_0257 (
PULPROG                zg
TD                  65536
SOLVENT             CDCl3
NS                     44
DS                      2
SWH             24038.461 Hz
FIDRES           0.366798 Hz
AQ              1.3631488 sec
RG                    512
DW                 20.800 usec
DE                   6.50 usec
TE                  295.5 K
D1             2.00000000 sec
TD0                     1
SFO1          128.4096890 MHz
NUC1                  11B
P1                   7.50 usec
PLW1          55.09999847 W

F2 - Processing parameters
SI                  32768
SF            128.4097430 MHz
WDW                    EM
SSB      0
LB                   1.00 Hz
GB       0
PC                   1.40
```

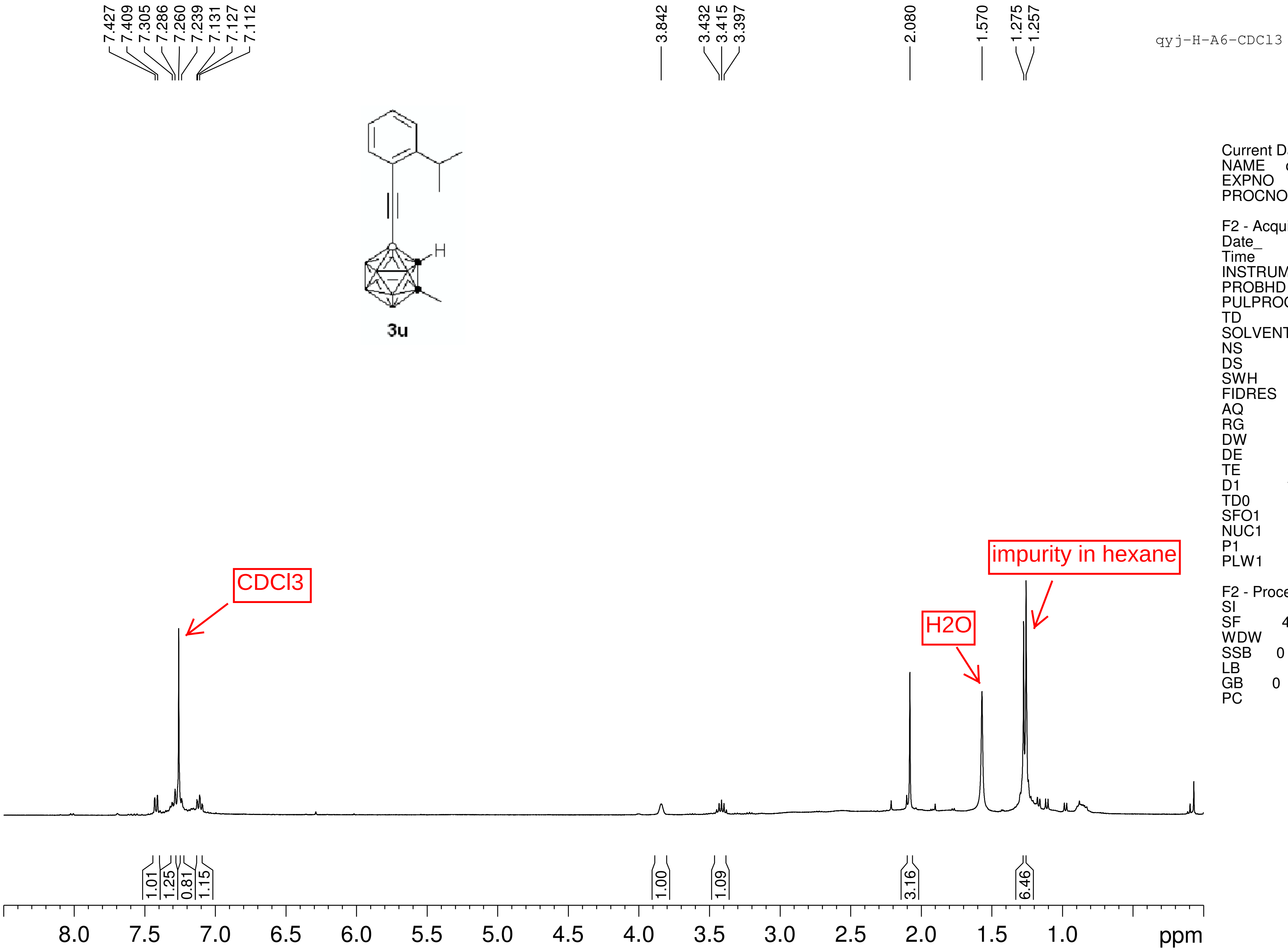

Current Data Parameters
NAME qyj-H-A6-CDCl3
EXPNO 1
PROCNO 1

F2 - Acquisition Parameters
Date_ 20160105
Time 9.41 h
INSTRUM spect
PROBHD Z108618_0257 (
PULPROG zg30
TD 65536
SOLVENT CDCl3
NS 11
DS 2
SWH 8012.820 Hz
FIDRES 0.122266 Hz
AQ 4.0894465 sec
RG 128
DW 62.400 usec
DE 6.50 usec
TE 295.1 K
D1 1.00000000 sec
TD0 1
SFO1 400.2324714 MHz
NUC1 1H
P1 12.80 usec
PLW1 13.56000042 W

F2 - Processing parameters
SI 65536
SF 400.2300105 MHz
WDW EM
SSB 0
LB 0.30 Hz
GB 0
PC 1.00

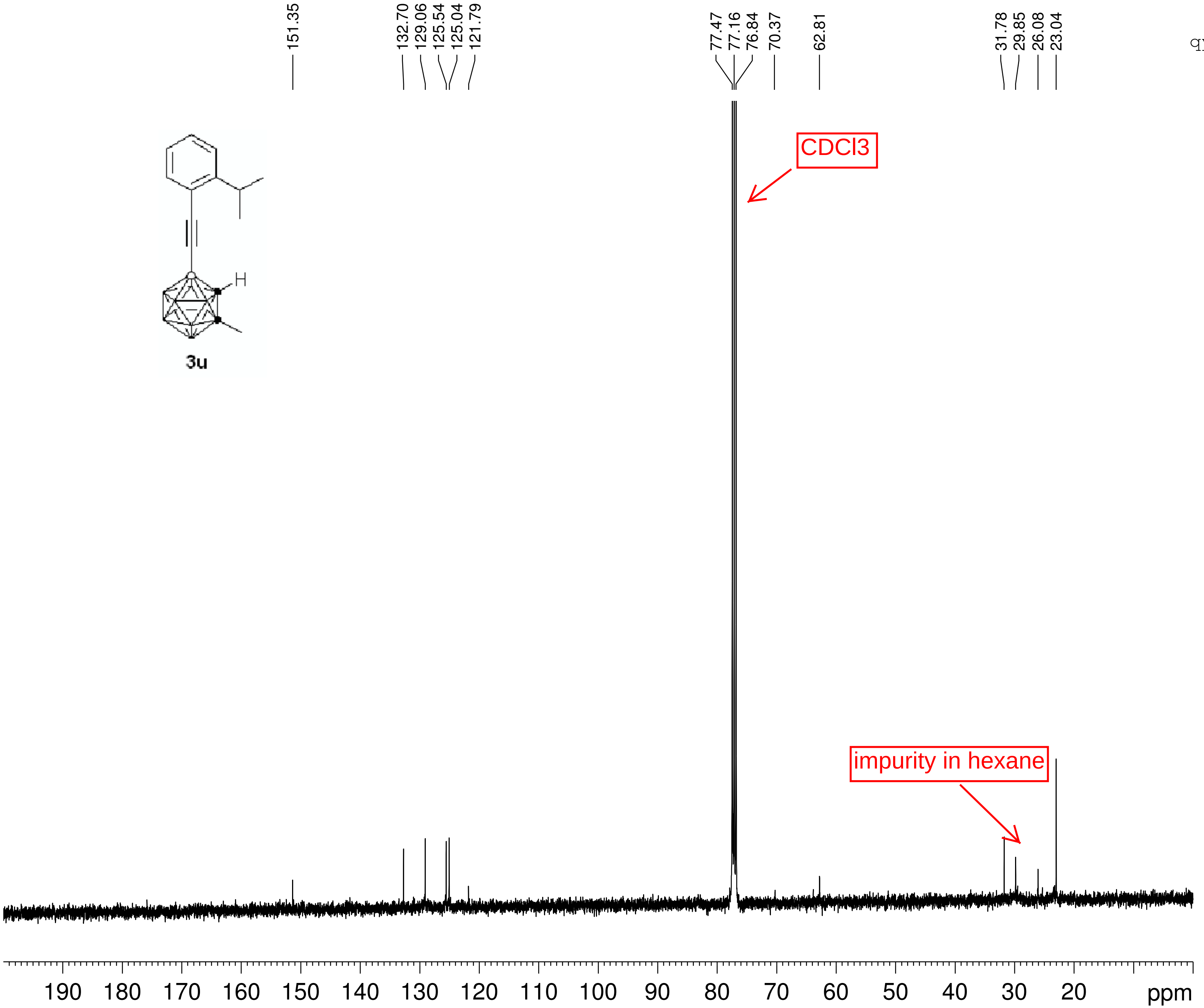

3u

qyj-C-A6-CDCl3

```
Current Data Parameters
NAME       qyj-C-A6-CDCl3
EXPNO                    1
PROCNO                   1

F2 - Acquisition Parameters
Date_             20160105
Time                 10.05 h
INSTRUM              spect
PROBHD    Z108618_0257 (
PULPROG            zgpg30
TD                  65536
SOLVENT             CDCl3
NS                   1095
DS                      4
SWH             29761.904 Hz
FIDRES           0.454131 Hz
AQ              1.1010048 sec
RG                    181
DW                 16.800 usec
DE                   6.50 usec
TE                  295.2 K
D1             2.00000000 sec
D11            0.03000000 sec
TD0                     1
SFO1          100.6479773 MHz
NUC1                  13C
P1                   9.50 usec
PLW1          55.34000015 W
SFO2          400.2316009 MHz
NUC2                   1H
CPDPRG[2          waltz16
PCPD2               90.00 usec
PLW2          13.56000042 W
PLW12          0.27428001 W
PLW13          0.13796000 W

F2 - Processing parameters
SI                  32768
SF            100.6379006 MHz
WDW                    EM
SSB       0
LB                   1.00 Hz
GB        0
PC                   1.40
```

qyj-B-A6-CDCl3

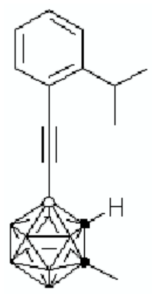

3u

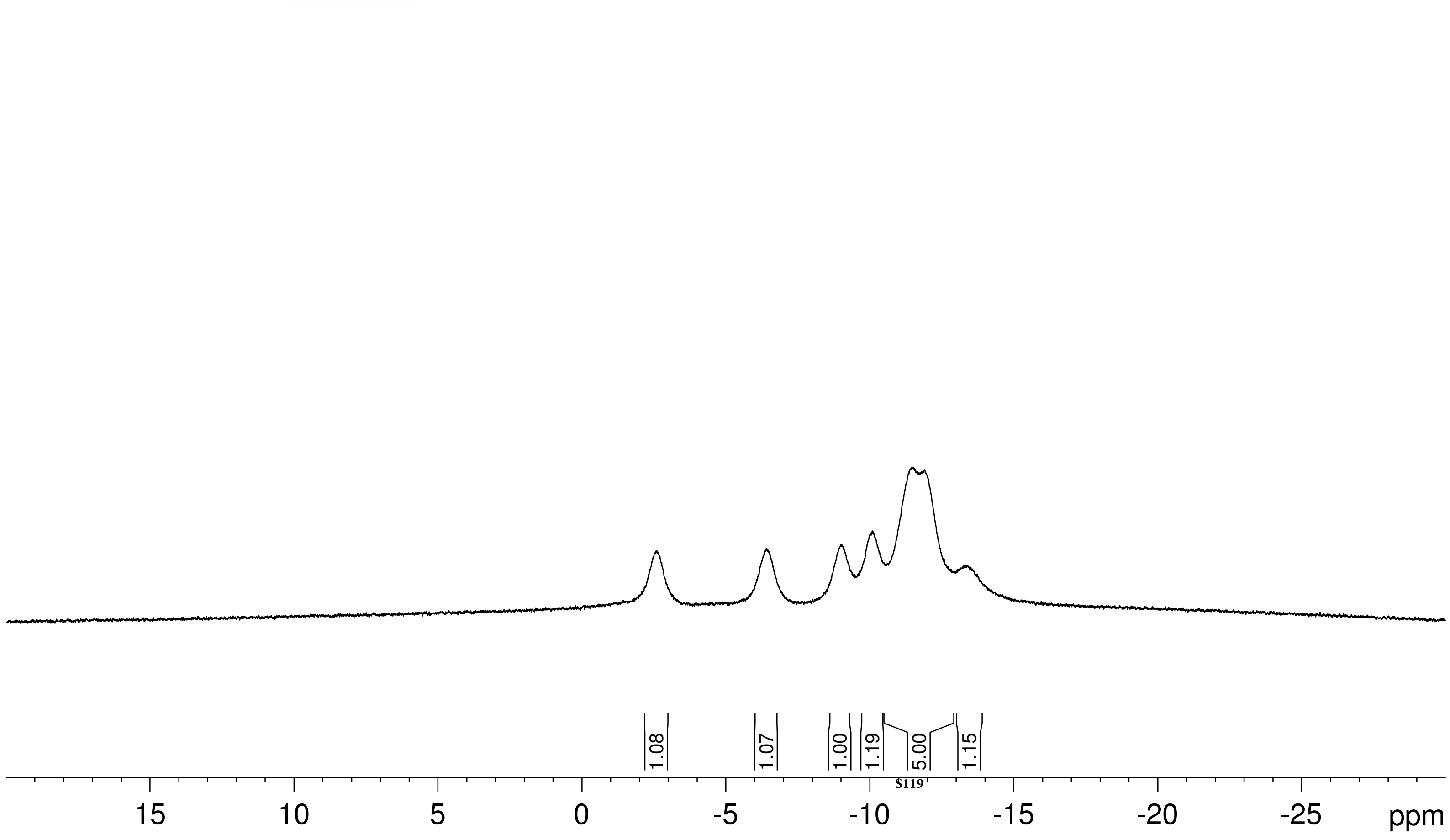

Current Data Parameters
NAME qyj-B-A6-CDCl3
EXPNO 1
PROCNO 1

F2 - Acquisition Parameters
Date_ 20160105
Time 9.47 h
INSTRUM spect
PROBHD Z108618_0257 (
PULPROG zgdc
TD 65536
SOLVENT CDCl3
NS 48
DS 4
SWH 24038.461 Hz
FIDRES 0.366798 Hz
AQ 1.3631488 sec
RG 456
DW 20.800 usec
DE 6.50 usec
TE 295.6 K
D1 2.00000000 sec
D11 0.03000000 sec
TD0 1
SFO1 128.4096890 MHz
NUC1 11B
P1 7.50 usec
PLW1 55.09999847 W
SFO2 400.2316009 MHz
NUC2 1H
CPDPRG[2 waltz16
PCPD2 90.00 usec
PLW2 13.56000042 W
PLW12 0.27428001 W

F2 - Processing parameters
SI 32768
SF 128.4097615 MHz
WDW EM
SSB 0
LB 1.00 Hz
GB 0
PC 1.40

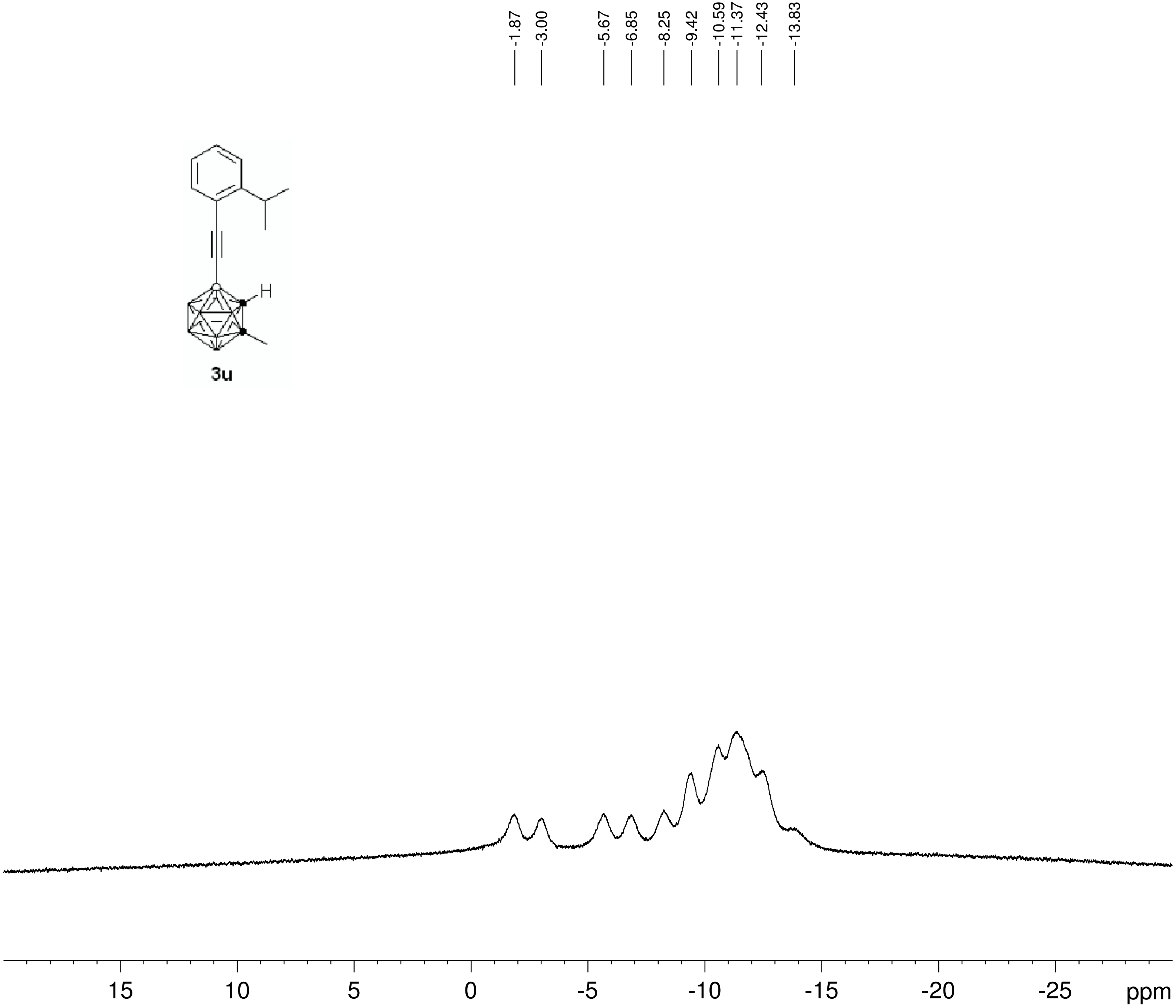

qyj-B-A6-CDCl3(C)

Current Data Parameters
NAME qyj-B-A6-CDCl3(C)
EXPNO 1
PROCNO 1

F2 - Acquisition Parameters
Date_ 20160105
Time 9.48 h
INSTRUM spect
PROBHD Z108618_0257 (
PULPROG zg
TD 65536
SOLVENT CDCl3
NS 48
DS 2
SWH 24038.461 Hz
FIDRES 0.366798 Hz
AQ 1.3631488 sec
RG 512
DW 20.800 usec
DE 6.50 usec
TE 295.1 K
D1 2.00000000 sec
TD0 1
SFO1 128.4096890 MHz
NUC1 11B
P1 7.50 usec
PLW1 55.09999847 W

F2 - Processing parameters
SI 32768
SF 128.4097430 MHz
WDW EM
SSB 0
LB 1.00 Hz
GB 0
PC 1.40

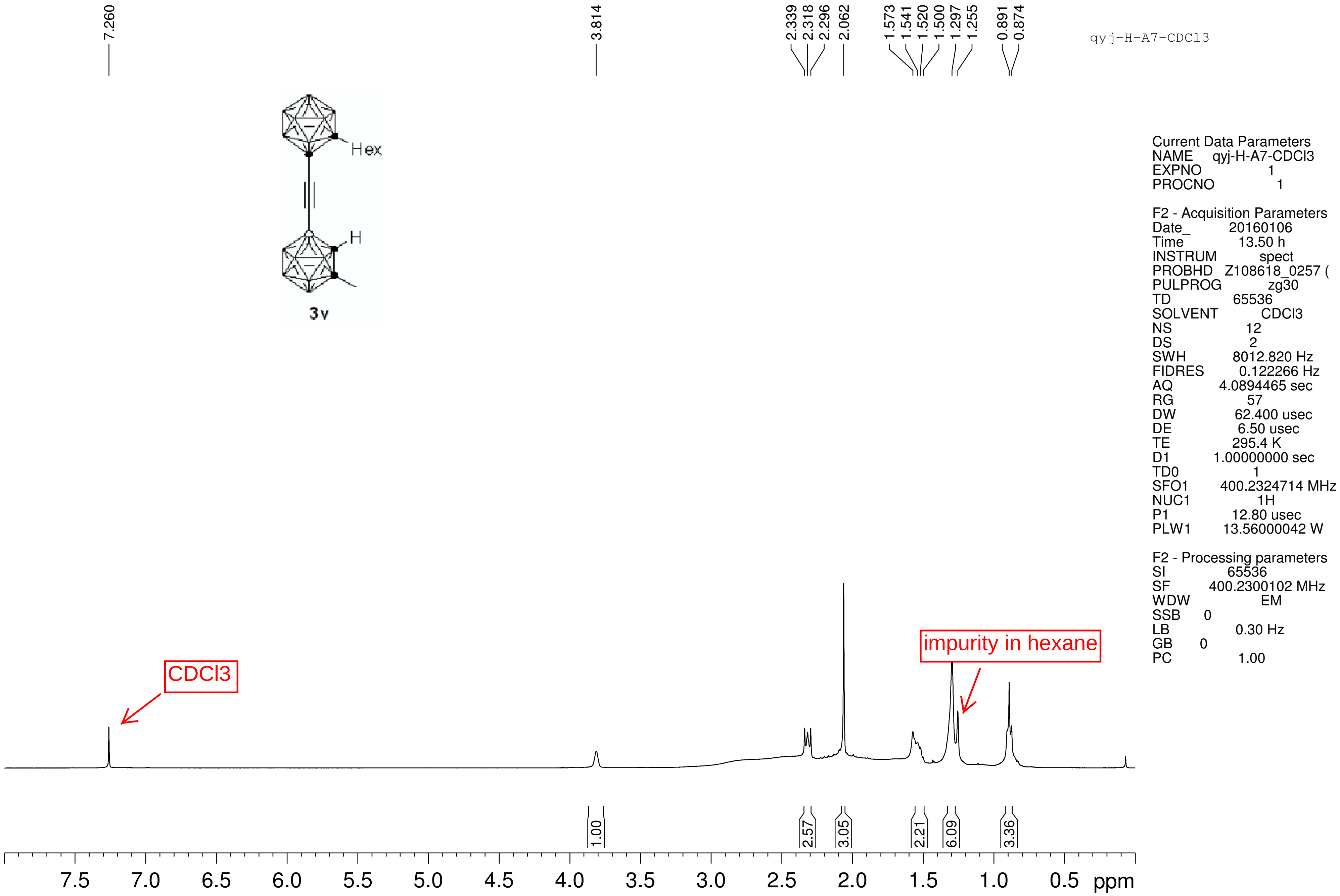

7.260
3.814
2.339
2.318
2.296
2.062
1.573
1.541
1.520
1.500
1.297
1.255
0.891
0.874
qyj-H-A7-CDCl3
Hex
H
3v
Current Data Parameters
NAME qyj-H-A7-CDCl3
EXPNO 1
PROCNO 1
F2 - Acquisition Parameters
Date_ 20160106
Time 13.50 h
INSTRUM spect
PROBHD Z108618_0257 (
PULPROG zg30
TD 65536
SOLVENT CDCl3
NS 12
DS 2
SWH 8012.820 Hz
FIDRES 0.122266 Hz
AQ 4.0894465 sec
RG 57
DW 62.400 usec
DE 6.50 usec
TE 295.4 K
D1 1.00000000 sec
TD0 1
SFO1 400.2324714 MHz
NUC1 1H
P1 12.80 usec
PLW1 13.56000042 W
F2 - Processing parameters
SI 65536
SF 400.2300102 MHz
WDW EM
SSB 0
LB 0.30 Hz
GB 0
PC 1.00
CDCl3
impurity in hexane
1.00
2.57
3.05
2.21
6.09
3.36
7.5
7.0
6.5
6.0
5.5
5.0
4.5
4.0
3.5
3.0
2.5
2.0
1.5
1.0
0.5
ppm

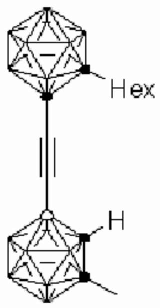

3v

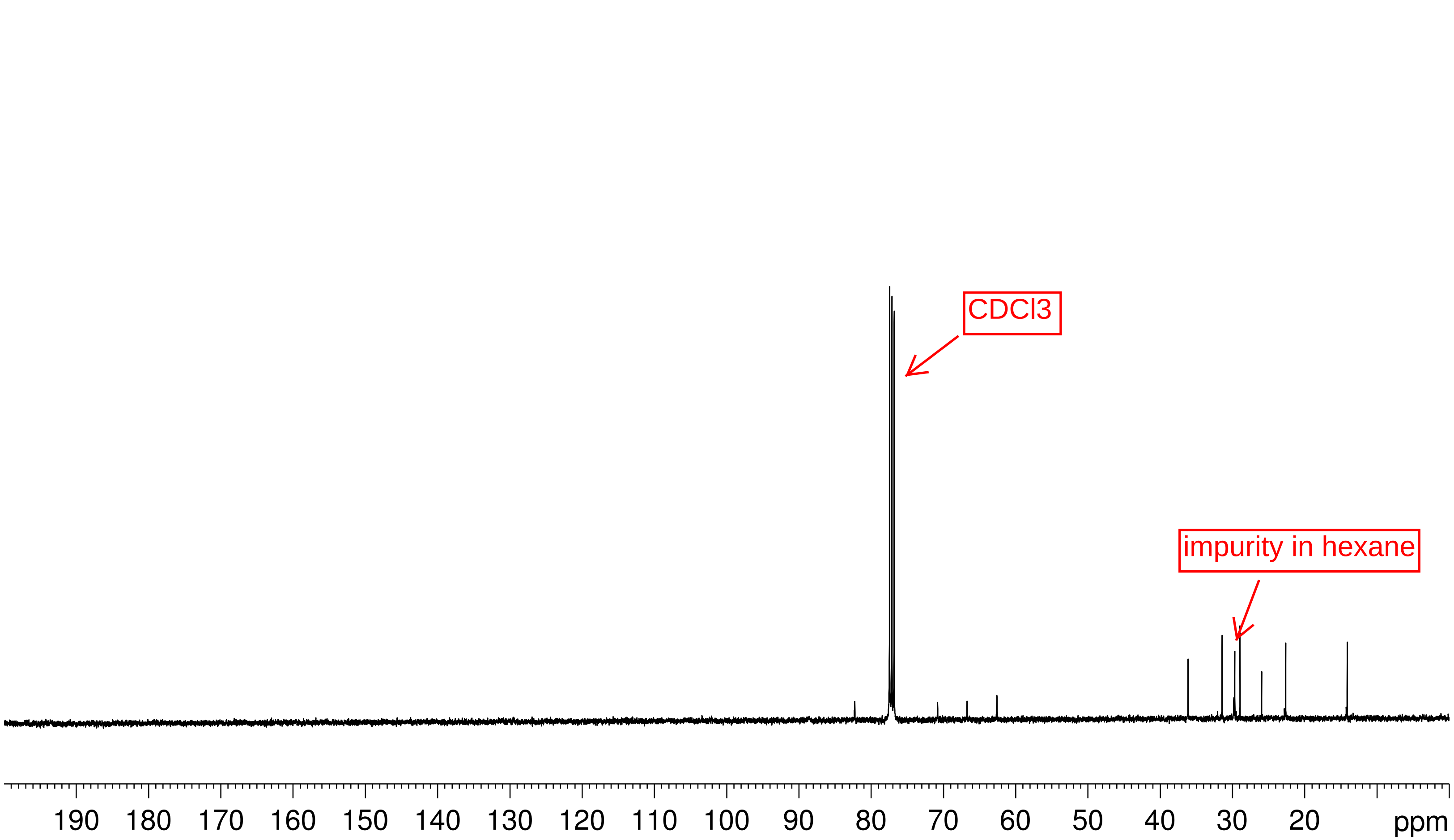

qyj-C-A7-CDCl3

```
Current Data Parameters
NAME       qyj-C-A7-CDCl3
EXPNO                   1
PROCNO                  1

F2 - Acquisition Parameters
Date_            20160106
Time                13.59 h
INSTRUM             spect
PROBHD     Z108618_0257 (
PULPROG            zgpg30
TD                  65536
SOLVENT             CDCl3
NS                    548
DS                      4
SWH             29761.904 Hz
FIDRES           0.454131 Hz
AQ              1.1010048 sec
RG                    181
DW                 16.800 usec
DE                   6.50 usec
TE                  295.3 K
D1             2.00000000 sec
D11            0.03000000 sec
TD0                     1
SFO1          100.6479773 MHz
NUC1                  13C
P1                   9.50 usec
PLW1          55.34000015 W
SFO2          400.2316009 MHz
NUC2                   1H
CPDPRG[2          waltz16
PCPD2               90.00 usec
PLW2          13.56000042 W
PLW12          0.27428001 W
PLW13          0.13796000 W

F2 - Processing parameters
SI                  32768
SF            100.6379015 MHz
WDW                    EM
SSB      0
LB                   1.00 Hz
GB       0
PC                   1.40
```

qyj-B-A7-CDCl3

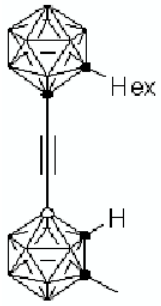

3v

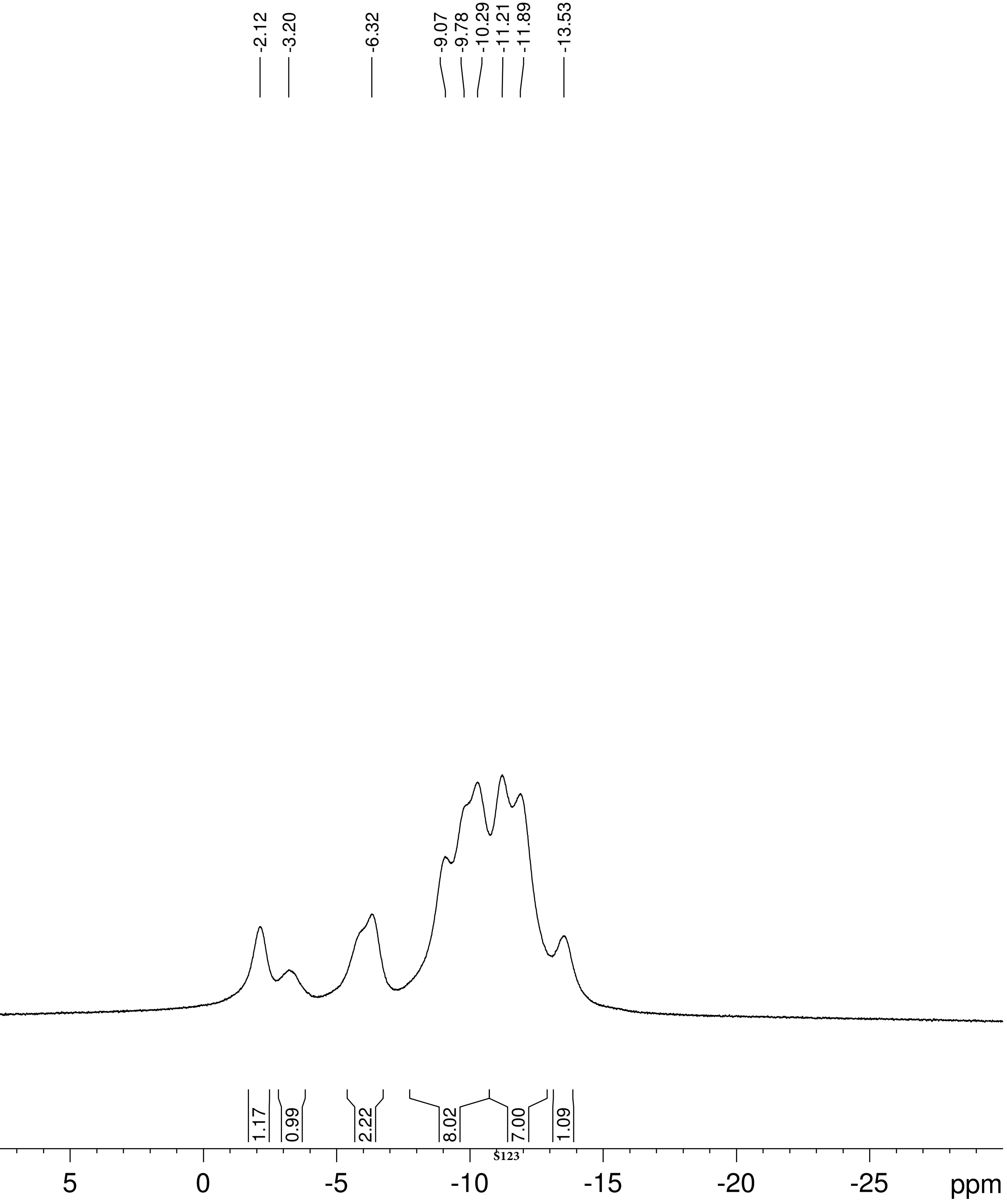


```
Current Data Parameters
NAME      qyj-B-A7-CDCl3
EXPNO                  1
PROCNO                 1

F2 - Acquisition Parameters
Date_           20160106
Time               13.54 h
INSTRUM            spect
PROBHD   Z108618_0257 (
PULPROG             zgdc
TD                 65536
SOLVENT            CDCl3
NS                    21
DS                     4
SWH            24038.461 Hz
FIDRES          0.366798 Hz
AQ             1.3631488 sec
RG                   287
DW                20.800 usec
DE                  6.50 usec
TE                 295.9 K
D1            2.00000000 sec
D11           0.03000000 sec
TD0                    1
SFO1         128.4096890 MHz
NUC1                 11B
P1                  7.50 usec
PLW1         55.09999847 W
SFO2         400.2316009 MHz
NUC2                  1H
CPDPRG[2         waltz16
PCPD2              90.00 usec
PLW2         13.56000042 W
PLW12         0.27428001 W

F2 - Processing parameters
SI                 32768
SF           128.4097615 MHz
WDW                   EM
SSB      0
LB                  1.00 Hz
GB       0
PC                  1.40
```

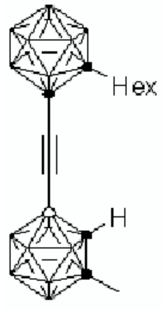

3v

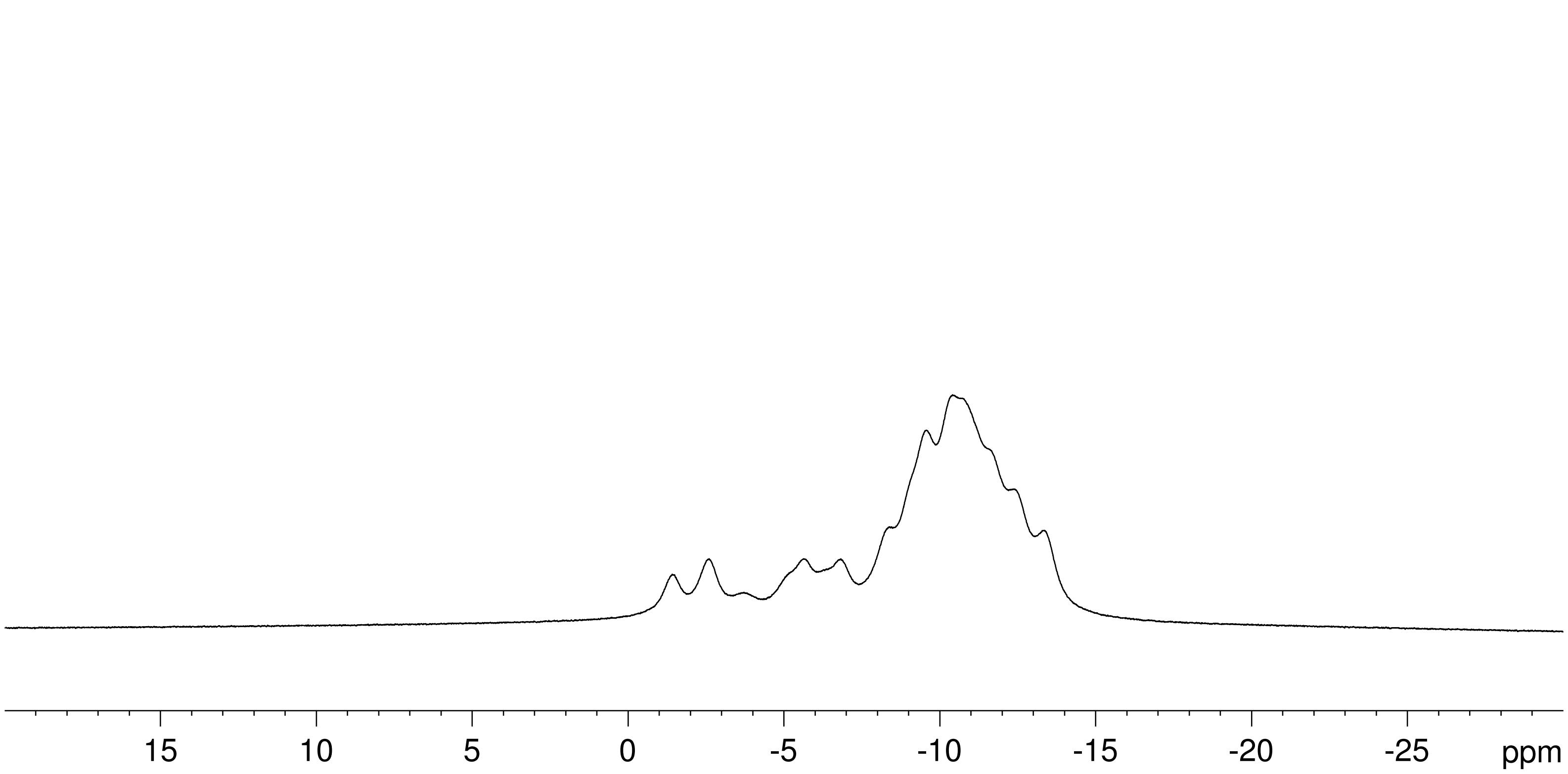

qyj-B-A7-CDCl3(C)

Current Data Parameters
NAME qyj-B-A7-CDCl3(C)
EXPNO 1
PROCNO 1

F2 - Acquisition Parameters
Date_ 20160106
Time 13.58 h
INSTRUM spect
PROBHD Z108618_0257 (
PULPROG zg
TD 65536
SOLVENT CDCl3
NS 51
DS 2
SWH 24038.461 Hz
FIDRES 0.366798 Hz
AQ 1.3631488 sec
RG 287
DW 20.800 usec
DE 6.50 usec
TE 295.2 K
D1 2.00000000 sec
TD0 1
SFO1 128.4096890 MHz
NUC1 11B
P1 7.50 usec
PLW1 55.09999847 W

F2 - Processing parameters
SI 32768
SF 128.4097430 MHz
WDW EM
SSB 0
LB 1.00 Hz
GB 0
PC 1.40

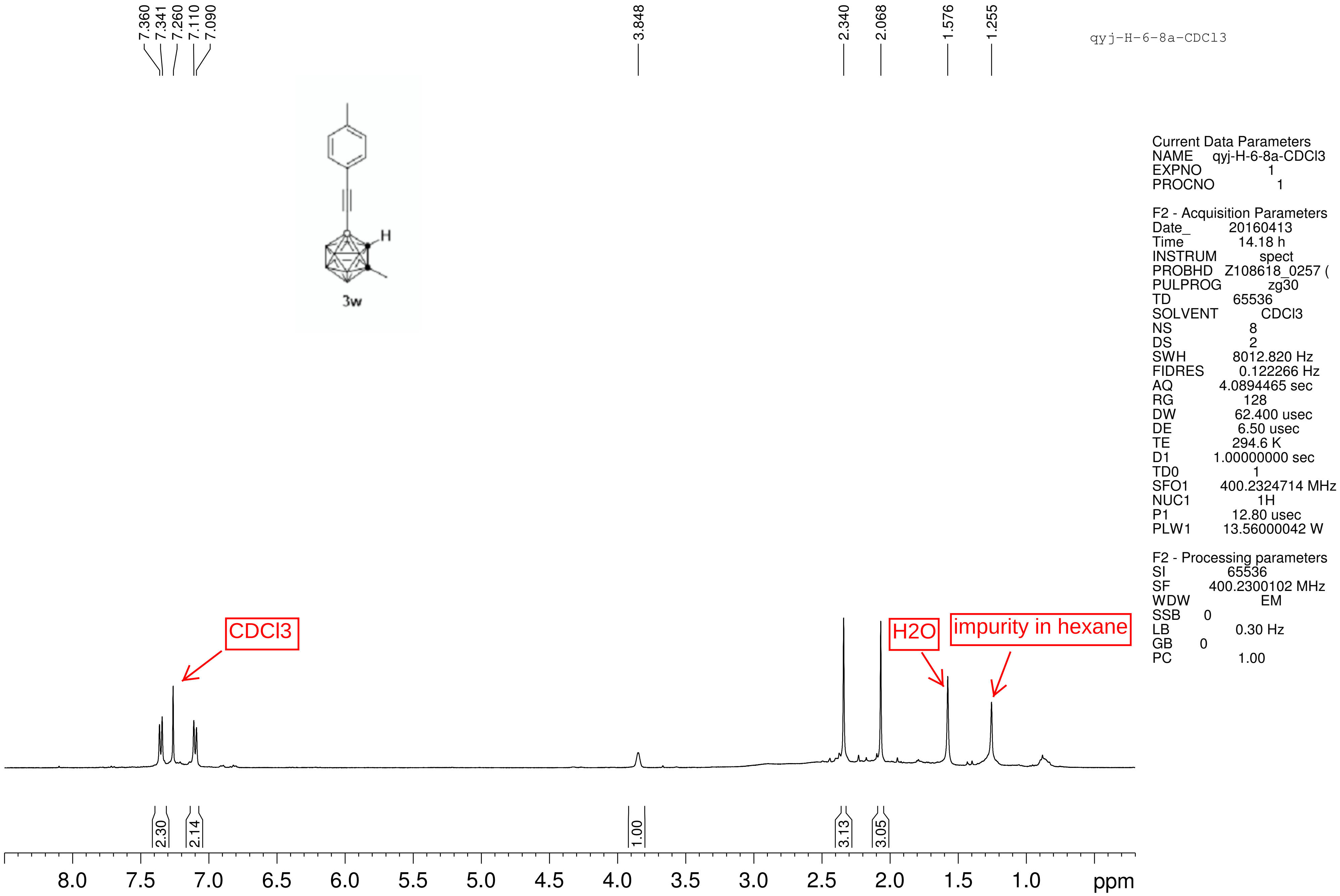
7.360
7.341
7.260
7.110
7.090
3.848
2.340
2.068
1.576
1.255
qyj-H-6-8a-CDCl3
H
3w
Current Data Parameters
NAME qyj-H-6-8a-CDCl3
EXPNO 1
PROCNO 1
F2 - Acquisition Parameters
Date_ 20160413
Time 14.18 h
INSTRUM spect
PROBHD Z108618_0257 (
PULPROG zg30
TD 65536
SOLVENT CDCl3
NS 8
DS 2
SWH 8012.820 Hz
FIDRES 0.122266 Hz
AQ 4.0894465 sec
RG 128
DW 62.400 usec
DE 6.50 usec
TE 294.6 K
D1 1.00000000 sec
TD0 1
SFO1 400.2324714 MHz
NUC1 1H
P1 12.80 usec
PLW1 13.56000042 W
F2 - Processing parameters
SI 65536
SF 400.2300102 MHz
WDW EM
SSB 0
LB 0.30 Hz
GB 0
PC 1.00
CDCl3
H2O
impurity in hexane
2.30
2.14
1.00
3.13
3.05
8.0
7.5
7.0
6.5
6.0
5.5
5.0
4.5
4.0
3.5
3.0
2.5
2.0
1.5
1.0
ppm

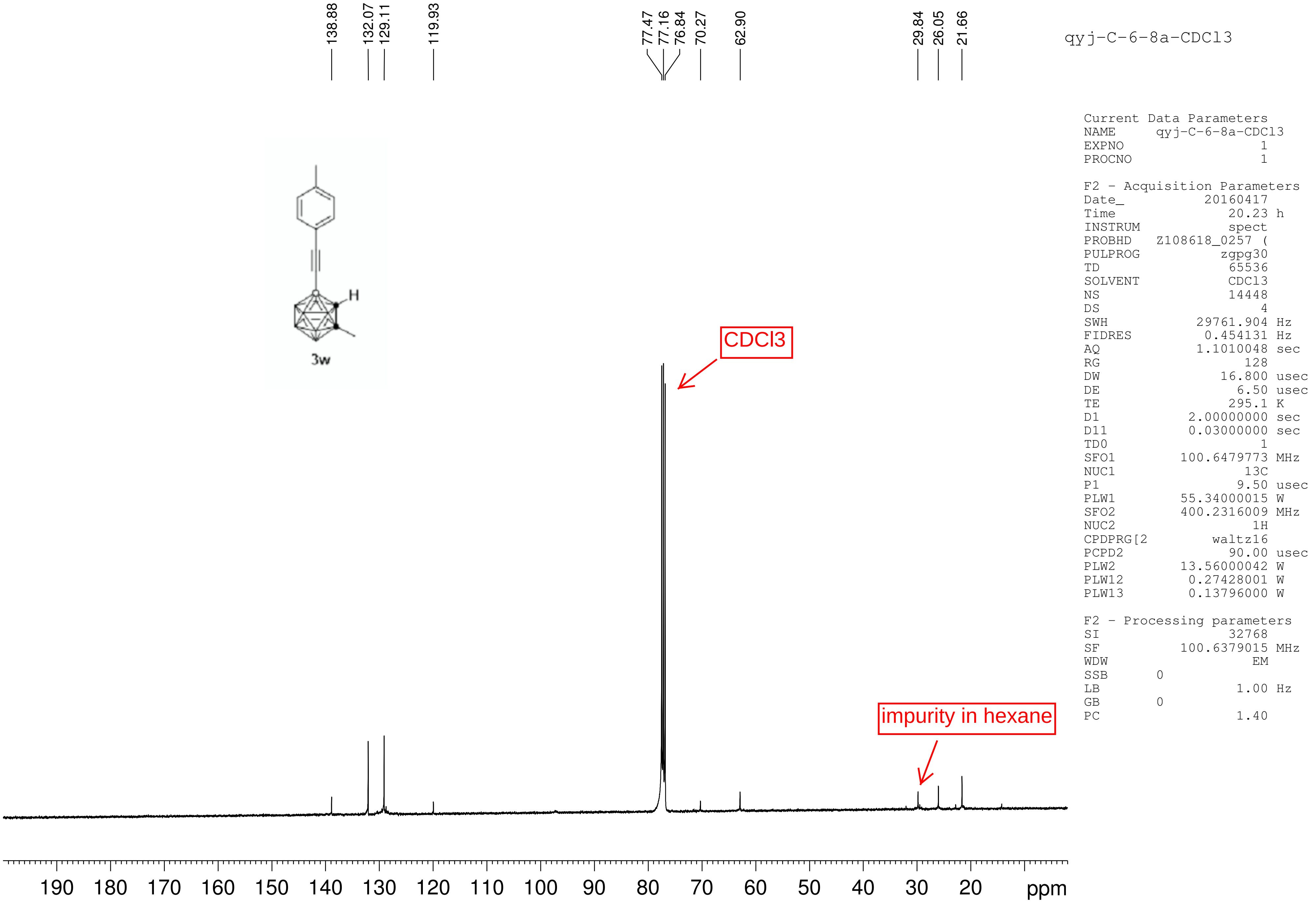
138.88
132.07
129.11
119.93
77.47
77.16
76.84
70.27
62.90
29.84
26.05
21.66
qyj-C-6-8a-CDCl3
3w
CDCl3
impurity in hexane
190 180 170 160 150 140 130 120 110 100 90 80 70 60 50 40 30 20 ppm
Current Data Parameters
NAME qyj-C-6-8a-CDCl3
EXPNO 1
PROCNO 1
F2 - Acquisition Parameters
Date_ 20160417
Time 20.23 h
INSTRUM spect
PROBHD Z108618_0257 (
PULPROG zgpg30
TD 65536
SOLVENT CDCl3
NS 14448
DS 4
SWH 29761.904 Hz
FIDRES 0.454131 Hz
AQ 1.1010048 sec
RG 128
DW 16.800 usec
DE 6.50 usec
TE 295.1 K
D1 2.00000000 sec
D11 0.03000000 sec
TD0 1
SFO1 100.6479773 MHz
NUC1 13C
P1 9.50 usec
PLW1 55.34000015 W
SFO2 400.2316009 MHz
NUC2 1H
CPDPRG[2 waltz16
PCPD2 90.00 usec
PLW2 13.56000042 W
PLW12 0.27428001 W
PLW13 0.13796000 W
F2 - Processing parameters
SI 32768
SF 100.6379015 MHz
WDW EM
SSB 0
LB 1.00 Hz
GB 0
PC 1.40

qyj-B-6-8a-CDCl3

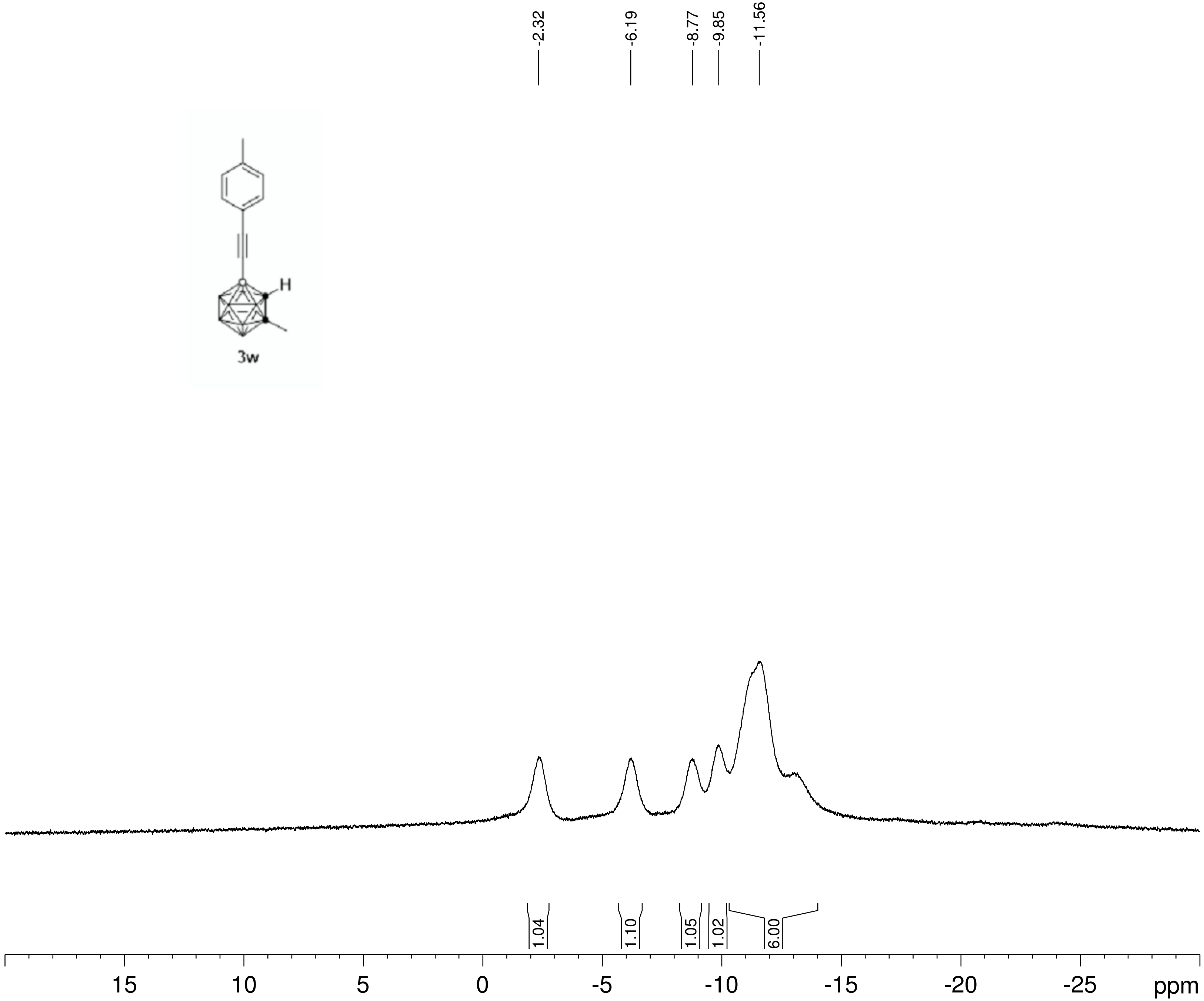


```
Current Data Parameters
NAME      qyj-B-6-8a-CDCl3
EXPNO                    1
PROCNO                   1

F2 - Acquisition Parameters
Date_             20160413
Time                 14.02 h
INSTRUM              spect
PROBHD    Z108618_0257 (
PULPROG               zgdc
TD                   65536
SOLVENT              CDCl3
NS                      20
DS                       4
SWH              24038.461 Hz
FIDRES            0.366798 Hz
AQ               1.3631488 sec
RG                     287
DW                  20.800 usec
DE                    6.50 usec
TE                   295.3 K
D1              2.00000000 sec
D11             0.03000000 sec
TD0                      1
SFO1           128.4096890 MHz
NUC1                   11B
P1                    7.50 usec
PLW1           55.09999847 W
SFO2           400.2316009 MHz
NUC2                    1H
CPDPRG[2          waltz16
PCPD2                90.00 usec
PLW2           13.56000042 W
PLW12           0.27428001 W

F2 - Processing parameters
SI                   32768
SF             128.4097615 MHz
WDW                     EM
SSB      0
LB                    1.00 Hz
GB       0
PC                    1.40
```

qyj-B-6-8a-CDCl3(c)

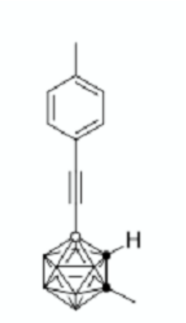

3w

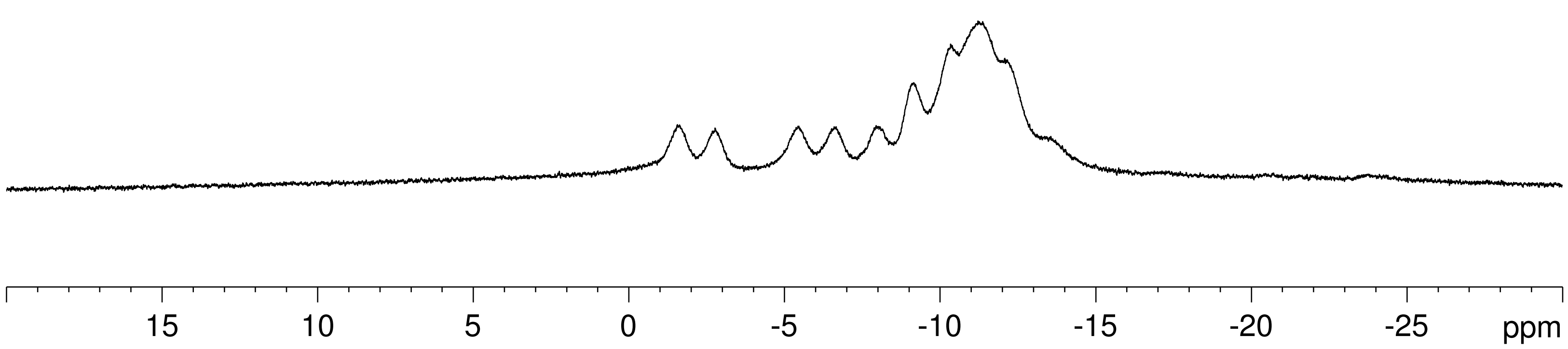

Current Data Parameters

NAME	qyj-B-6-8a-CDCl3(c)
EXPNO	1
PROCNO	1

F2 - Acquisition Parameters

Date_	20160413
Time	14.05 h
INSTRUM	spect
PROBHD	Z108618_0257 (
PULPROG	zg
TD	65536
SOLVENT	CDCl3
NS	20
DS	2
SWH	24038.461 Hz
FIDRES	0.366798 Hz
AQ	1.3631488 sec
RG	287
DW	20.800 usec
DE	6.50 usec
TE	295.1 K
D1	2.00000000 sec
TD0	1
SFO1	128.4096890 MHz
NUC1	11B
P1	7.50 usec
PLW1	55.09999847 W

F2 - Processing parameters

SI	32768
SF	128.4097430 MHz
WDW	EM
SSB	0
LB	1.00 Hz
GB	0
PC	1.40

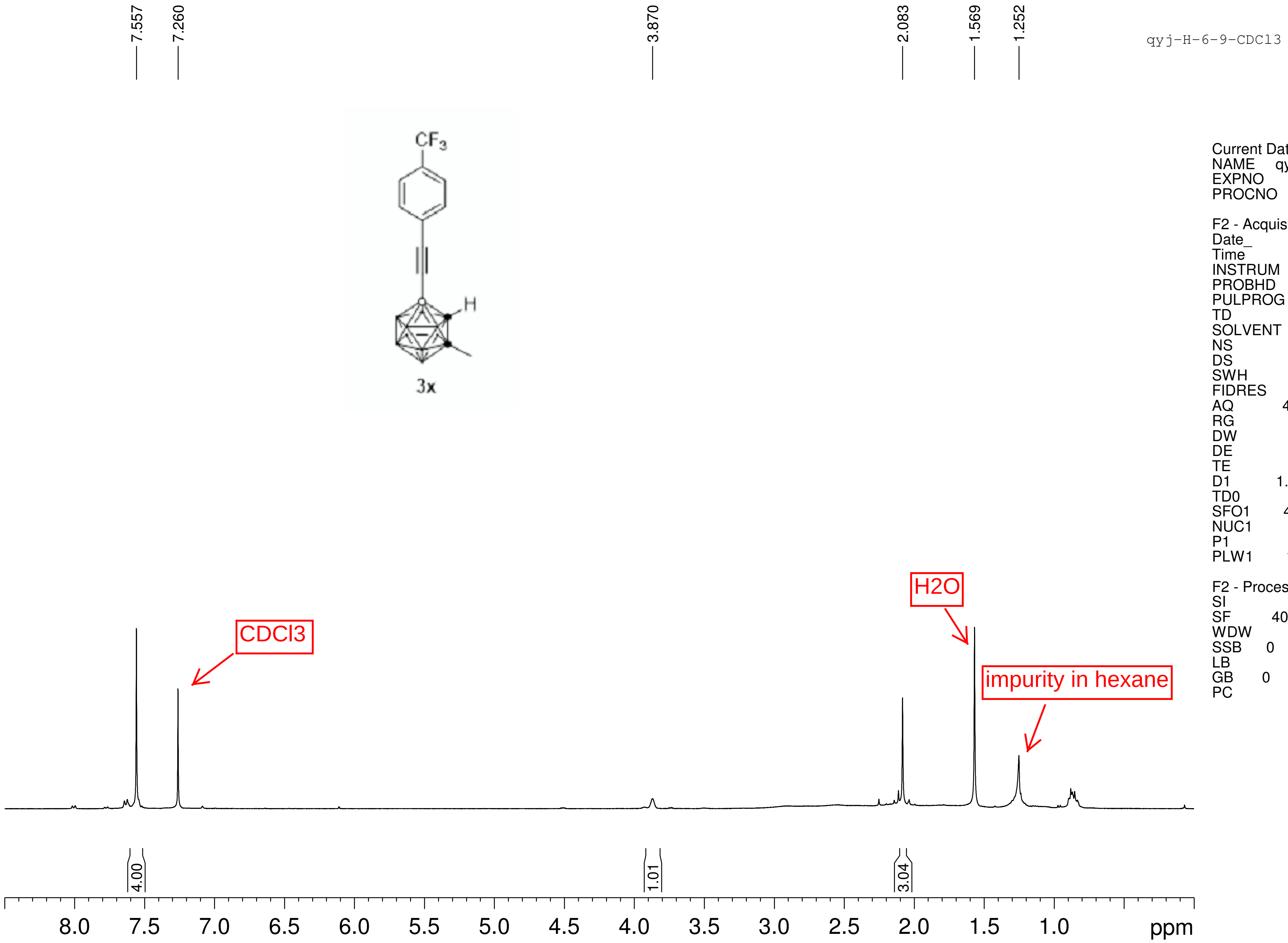

Current Data Parameters
NAME qyj-H-6-9-CDCl3
EXPNO 1
PROCNO 1

F2 - Acquisition Parameters
Date_ 20160420
Time 16.48 h
INSTRUM spect
PROBHD Z108618_0257 (
PULPROG zg30
TD 65536
SOLVENT CDCl3
NS 12
DS 2
SWH 8012.820 Hz
FIDRES 0.122266 Hz
AQ 4.0894465 sec
RG 128
DW 62.400 usec
DE 6.50 usec
TE 294.4 K
D1 1.00000000 sec
TD0 1
SFO1 400.2324714 MHz
NUC1 1H
P1 12.80 usec
PLW1 13.56000042 W

F2 - Processing parameters
SI 65536
SF 400.2300103 MHz
WDW EM
SSB 0
LB 0.30 Hz
GB 0
PC 1.00

qyj-C-6-9-CDCl3

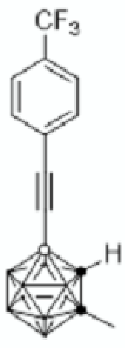

3x

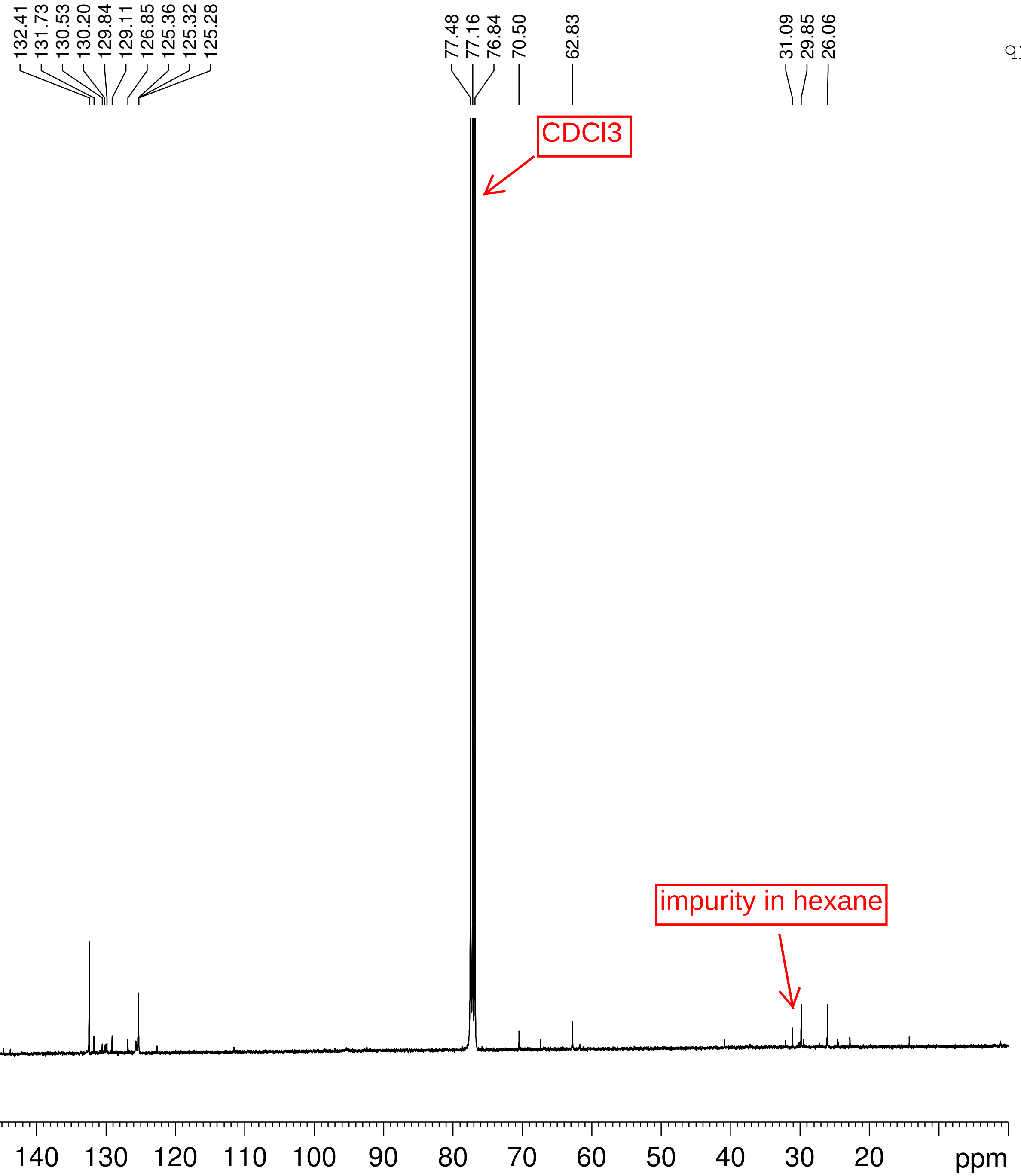


```
Current Data Parameters
NAME      qyj-C-6-9-CDCl3
EXPNO                   1
PROCNO                  1

F2 - Acquisition Parameters
Date_            20160422
Time                22.22 h
INSTRUM             spect
PROBHD    Z108618_0257 (
PULPROG            zgpg30
TD                  65536
SOLVENT             CDCl3
NS                  15472
DS                      4
SWH             29761.904 Hz
FIDRES           0.454131 Hz
AQ              1.1010048 sec
RG                    128
DW                 16.800 usec
DE                   6.50 usec
TE                  295.4 K
D1             2.00000000 sec
D11            0.03000000 sec
TD0                     1
SFO1          100.6479773 MHz
NUC1                  13C
P1                   9.50 usec
PLW1          55.34000015 W
SFO2          400.2316009 MHz
NUC2                   1H
CPDPRG[2          waltz16
PCPD2               90.00 usec
PLW2          13.56000042 W
PLW12          0.27428001 W
PLW13          0.13796000 W

F2 - Processing parameters
SI                  32768
SF            100.6379006 MHz
WDW                    EM
SSB      0
LB                   1.00 Hz
GB       0
PC                   1.40
```

qyj-B-6-9-CDCl3

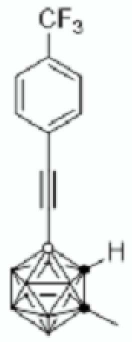

3x

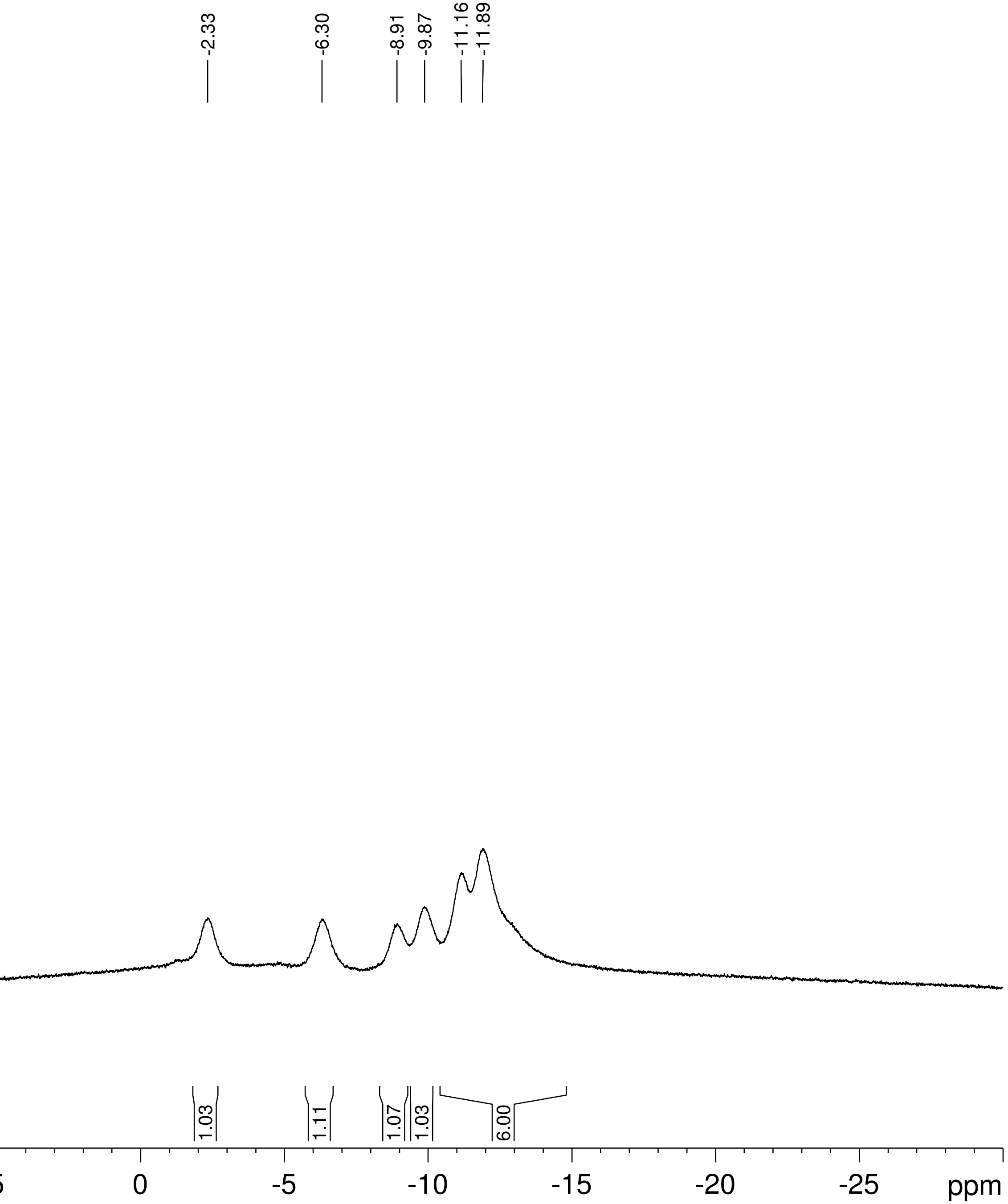

Current Data Parameters
NAME qyj-B-6-9-CDCl3
EXPNO 1
PROCNO 1

F2 - Acquisition Parameters
Date_ 20160420
Time 16.38 h
INSTRUM spect
PROBHD Z108618_0257 (
PULPROG zgdc
TD 65536
SOLVENT CDCl3
NS 48
DS 4
SWH 24038.461 Hz
FIDRES 0.366798 Hz
AQ 1.3631488 sec
RG 512
DW 20.800 usec
DE 6.50 usec
TE 294.8 K
D1 2.00000000 sec
D11 0.03000000 sec
TD0 1
SFO1 128.4096890 MHz
NUC1 11B
P1 7.50 usec
PLW1 55.09999847 W
SFO2 400.2316009 MHz
NUC2 1H
CPDPRG[2 waltz16
PCPD2 90.00 usec
PLW2 13.56000042 W
PLW12 0.27428001 W

F2 - Processing parameters
SI 32768
SF 128.4097615 MHz
WDW EM
SSB 0
LB 1.00 Hz
GB 0
PC 1.40

qyj-B-6-9-CDCl3(c)

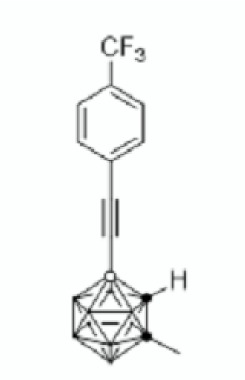

3x

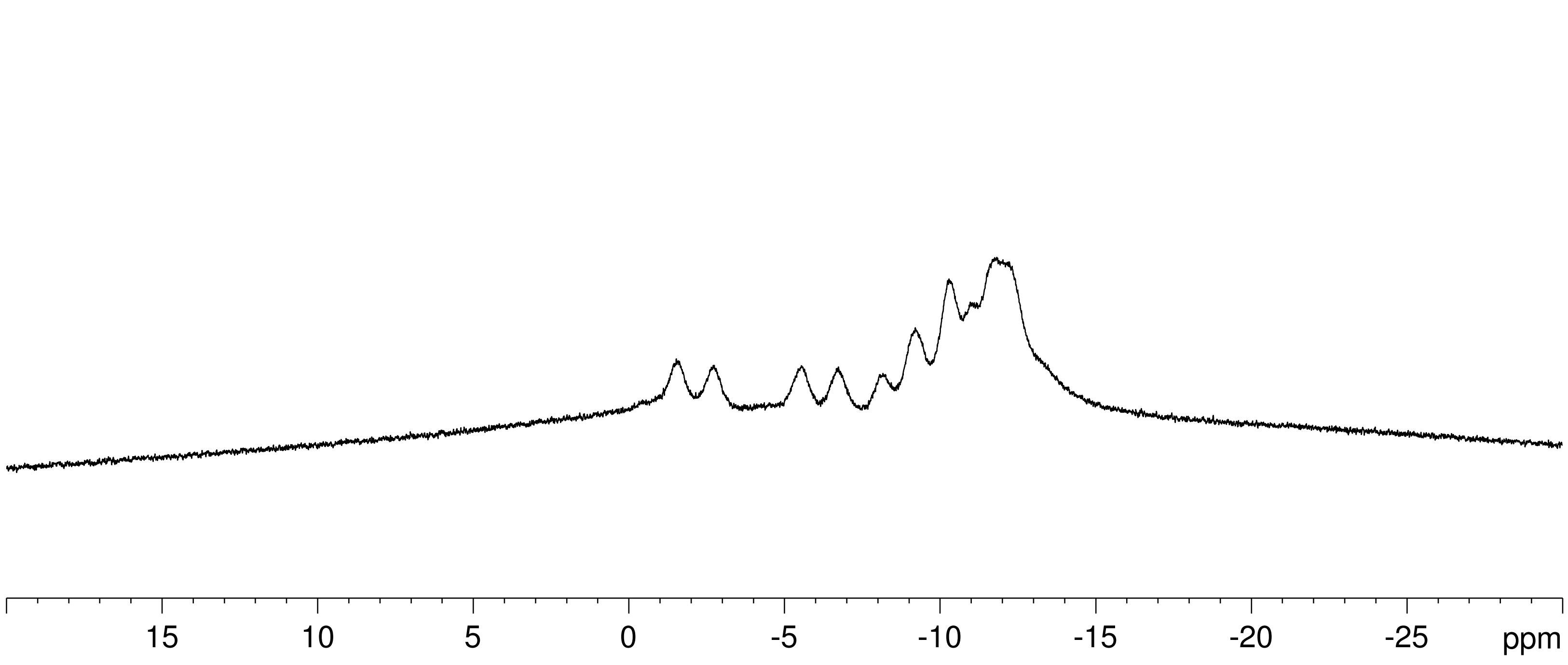

Current Data Parameters
NAME qyj-B-6-9-CDCl3(c)
EXPNO 1
PROCNO 1

F2 - Acquisition Parameters
Date_ 20160420
Time 16.42 h
INSTRUM spect
PROBHD Z108618_0257 (
PULPROG zg
TD 65536
SOLVENT CDCl3
NS 40
DS 2
SWH 24038.461 Hz
FIDRES 0.366798 Hz
AQ 1.3631488 sec
RG 287
DW 20.800 usec
DE 6.50 usec
TE 294.5 K
D1 2.00000000 sec
TD0 1
SFO1 128.4096890 MHz
NUC1 11B
P1 7.50 usec
PLW1 55.09999847 W

F2 - Processing parameters
SI 32768
SF 128.4097430 MHz
WDW EM
SSB 0
LB 1.00 Hz
GB 0
PC 1.40

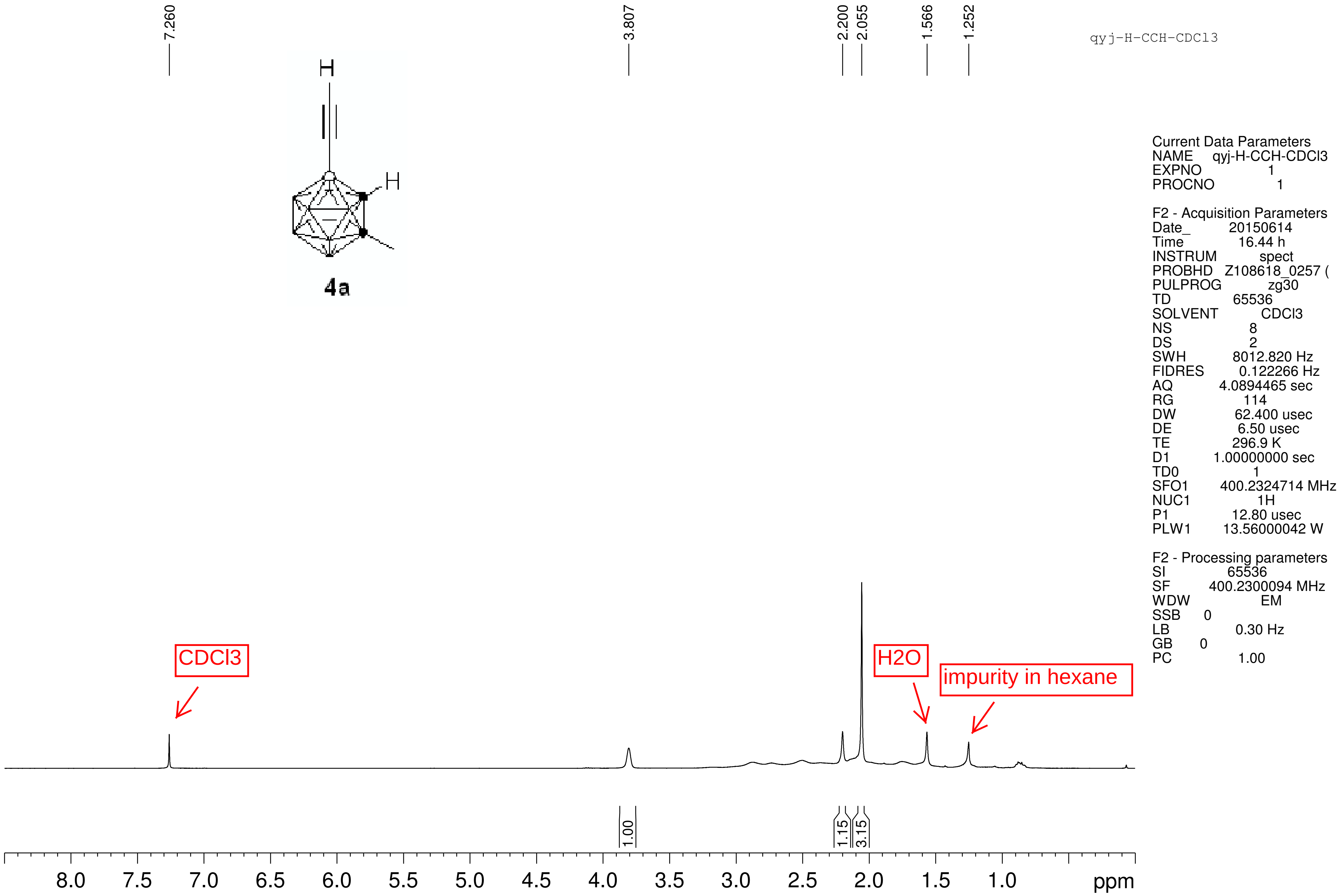

7.260
3.807
2.200
2.055
1.566
1.252
qyj-H-CCH-CDCl3
H
H
4a
CDCl3
H2O
impurity in hexane
1.00
1.15
3.15
8.0
7.5
7.0
6.5
6.0
5.5
5.0
4.5
4.0
3.5
3.0
2.5
2.0
1.5
1.0
ppm
Current Data Parameters
NAME qyj-H-CCH-CDCl3
EXPNO 1
PROCNO 1
F2 - Acquisition Parameters
Date_ 20150614
Time 16.44 h
INSTRUM spect
PROBHD Z108618_0257 (
PULPROG zg30
TD 65536
SOLVENT CDCl3
NS 8
DS 2
SWH 8012.820 Hz
FIDRES 0.122266 Hz
AQ 4.0894465 sec
RG 114
DW 62.400 usec
DE 6.50 usec
TE 296.9 K
D1 1.00000000 sec
TD0 1
SFO1 400.2324714 MHz
NUC1 1H
P1 12.80 usec
PLW1 13.56000042 W
F2 - Processing parameters
SI 65536
SF 400.2300094 MHz
WDW EM
SSB 0
LB 0.30 Hz
GB 0
PC 1.00

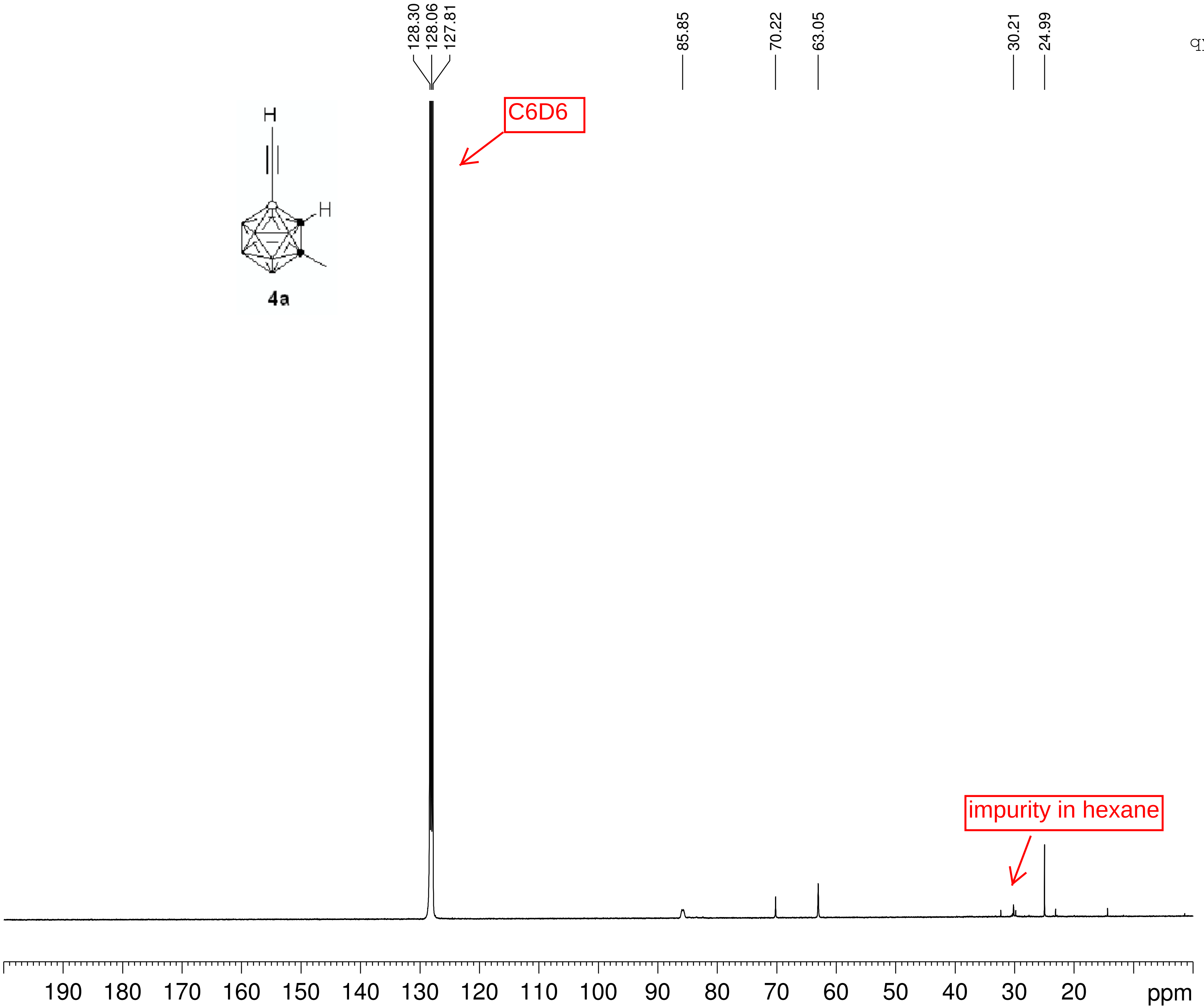

qyj-C-6-4a-C6D6

```
Current Data Parameters
NAME      qyj-C-6-4a-C6D6
EXPNO                   1
PROCNO                  1

F2 - Acquisition Parameters
Date_            20160408
Time                23.07 h
INSTRUM             spect
PROBHD    Z108618_0257 (
PULPROG            zgpg30
TD                  65536
SOLVENT              C6D6
NS                  11533
DS                      4
SWH             29761.904 Hz
FIDRES           0.454131 Hz
AQ              1.1010048 sec
RG                    128
DW                 16.800 usec
DE                   6.50 usec
TE                  294.6 K
D1             2.00000000 sec
D11            0.03000000 sec
TD0                     1
SFO1          100.6479773 MHz
NUC1                  13C
P1                   9.50 usec
PLW1          55.34000015 W
SFO2          400.2316009 MHz
NUC2                   1H
CPDPRG[2          waltz16
PCPD2               90.00 usec
PLW2          13.56000042 W
PLW12          0.27428001 W
PLW13          0.13796000 W

F2 - Processing parameters
SI                  32768
SF            100.6378780 MHz
WDW                    EM
SSB       0
LB                   1.00 Hz
GB        0
PC                   1.40
```

qyj-B-CBCCH-CDCl3

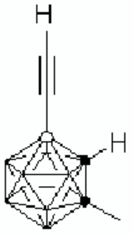

4a

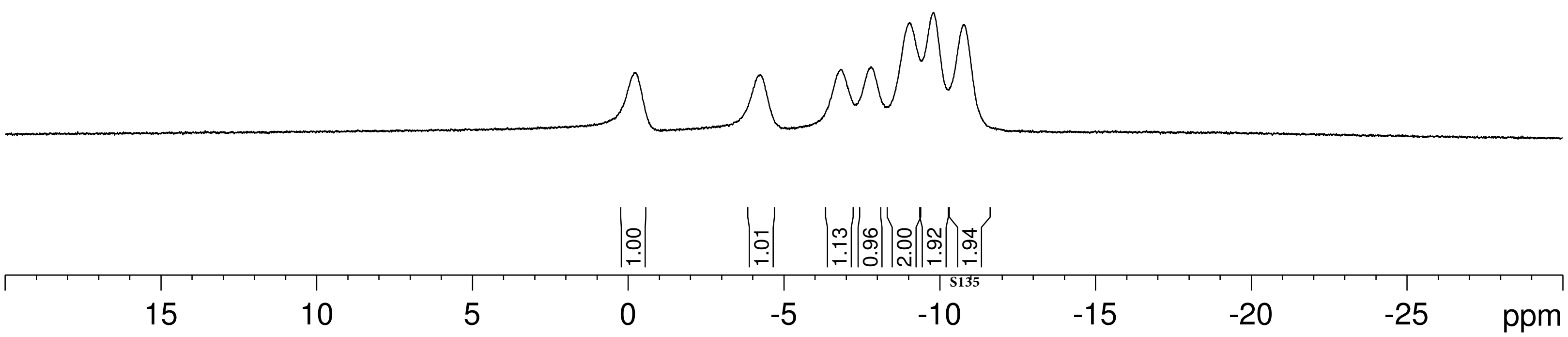


```
Current Data Parameters
NAME      qyj-B-CBCCH-CDCl3
EXPNO                     1
PROCNO                    1

F2 - Acquisition Parameters
Date_          20150629
Time              18.44 h
INSTRUM           spect
PROBHD    Z108618_0257 (
PULPROG          zgpg30
TD                65536
SOLVENT           CDCl3
NS                   12
DS                    4
SWH           29761.904 Hz
FIDRES         0.454131 Hz
AQ            1.1010048 sec
RG                  456
DW               16.800 usec
DE                 6.50 usec
TE                295.5 K
D1           2.00000000 sec
D11          0.03000000 sec
TD0                   1
SFO1        128.4096890 MHz
NUC1                11B
P1                 7.50 usec
PLW1        55.09999847 W
SFO2        400.2316008 MHz
NUC2                 1H
CPDPRG[2        waltz16
PCPD2             90.00 usec
PLW2        13.56000042 W
PLW12        0.27428001 W
PLW13        0.13796000 W

F2 - Processing parameters
SI                32768
SF          128.4095347 MHz
WDW                  EM
SSB      0
LB                 1.00 Hz
GB       0
PC                 1.40
```

qyj-B-CBCCH-CDCl3(C)

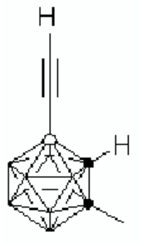

4a

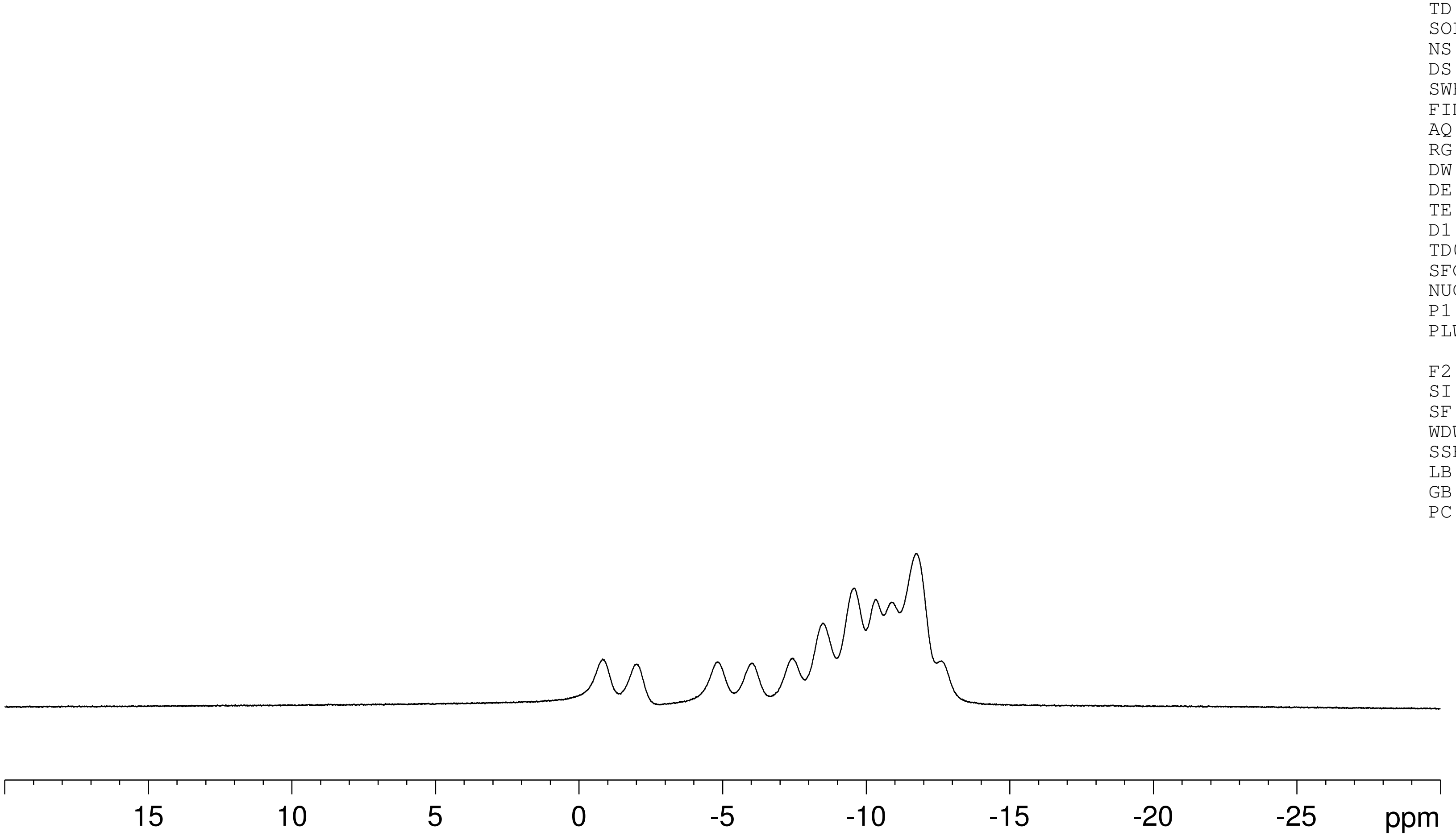


```
Current Data Parameters
NAME     qyj-B-CBCCH-CDCl3(C)
EXPNO                   1
PROCNO                  1

F2 - Acquisition Parameters
Date_            20150629
Time                18.46 h
INSTRUM             spect
PROBHD   Z108618_0257 (
PULPROG                zg
TD                  65536
SOLVENT             CDCl3
NS                     20
DS                      4
SWH             25510.203 Hz
FIDRES           0.389255 Hz
AQ              1.2845056 sec
RG                    256
DW                 19.600 usec
DE                   6.50 usec
TE                  295.4 K
D1             1.00000000 sec
TD0                     1
SFO1          128.4096891 MHz
NUC1                  11B
P1                   7.50 usec
PLW1          55.09999847 W

F2 - Processing parameters
SI                  32768
SF            128.4096891 MHz
WDW                    EM
SSB      0
LB                   1.00 Hz
GB       0
PC                   1.40
```

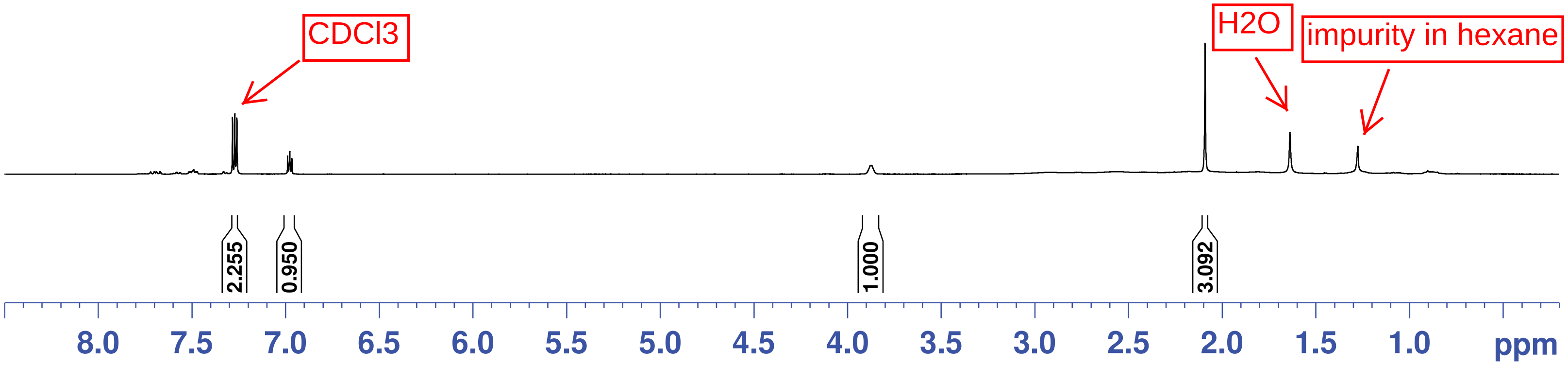

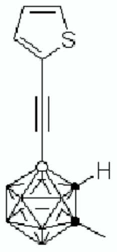

5a

qyj-H-6-1-CDCl3

```
Current Data Parameters
NAME      qyj-H-6-1-CDCl3
EXPNO                   1
PROCNO                  1

F2 - Acquisition Parameters
Date_            20150614
Time                11.37 h
INSTRUM             spect
PROBHD   Z824601_0021 (
PULPROG              zg30
TD                  65536
SOLVENT             CDCl3
NS                     16
DS                      2
SWH              8012.820 Hz
FIDRES           0.122266 Hz
AQ              4.0894465 sec
RG                    203
DW                 62.400 usec
DE                   6.50 usec
TE                  294.7 K
D1             1.00000000 sec
TD0                     1
SFO1          400.1324708 MHz
NUC1                   1H
P1                  15.00 usec
PLW1           8.31000042 W

F2 - Processing parameters
SI                  65536
SF            400.1300006 MHz
WDW                    EM
SSB      0
LB                   0.30 Hz
GB       0
PC                   1.00
```

qyj-C-6-1-CDCl3

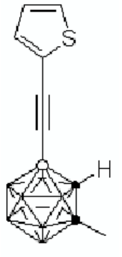

5a

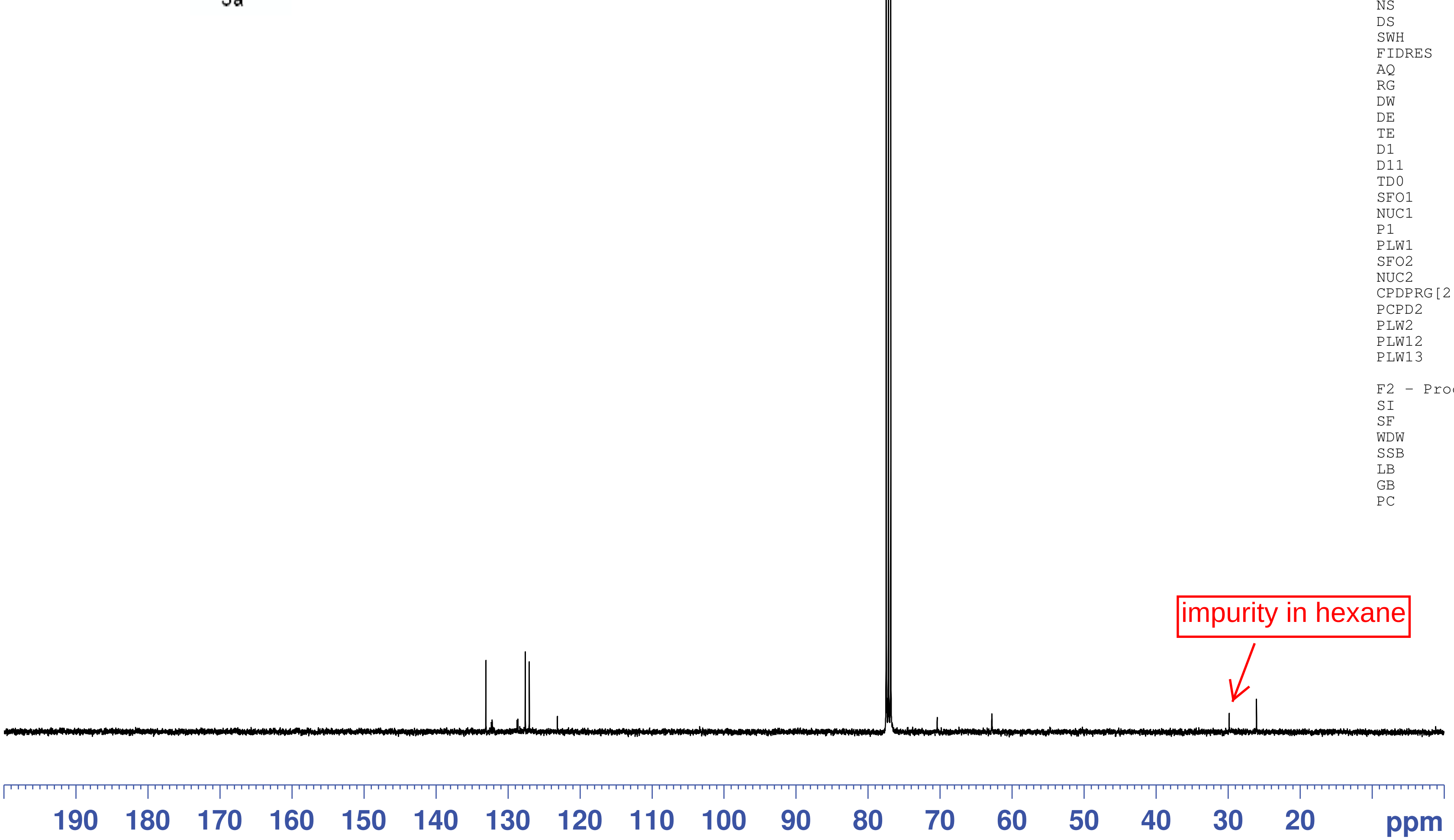

Current Data Parameters
NAME qyj-C-6-1-CDCl3
EXPNO 1
PROCNO 1

F2 - Acquisition Parameters
Date_ 20150614
Time 13.50 h
INSTRUM spect
PROBHD Z824601_0021 (
PULPROG zgpg30
TD 65536
SOLVENT CDCl3
NS 1074
DS 4
SWH 24038.461 Hz
FIDRES 0.366798 Hz
AQ 1.3631488 sec
RG 203
DW 20.800 usec
DE 6.50 usec
TE 295.1 K
D1 2.00000000 sec
D11 0.03000000 sec
TD0 1
SFO1 100.6228298 MHz
NUC1 13C
P1 9.50 usec
PLW1 41.25000000 W
SFO2 400.1316005 MHz
NUC2 1H
CPDPRG[2 waltz16
PCPD2 90.00 usec
PLW2 8.31000042 W
PLW12 0.23083000 W
PLW13 0.11611000 W

F2 - Processing parameters
SI 32768
SF 100.6127557 MHz
WDW EM
SSB 0
LB 1.00 Hz
GB 0
PC 1.40

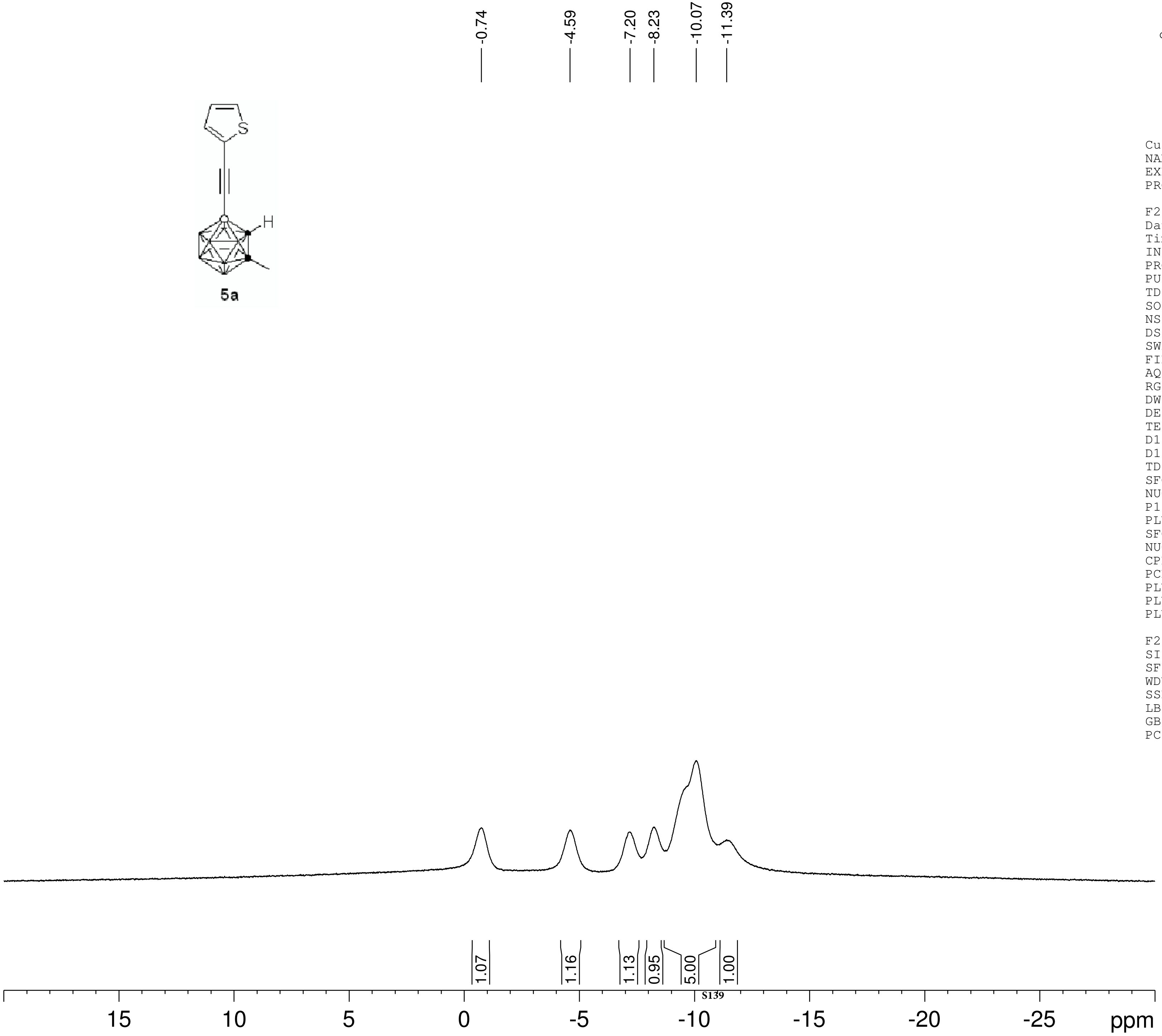

qyj-B-6-1-CDCl3

```
Current Data Parameters
NAME     qyj-B-6-1-CDCl3
EXPNO                  1
PROCNO                 1

F2 - Acquisition Parameters
Date_           20150622
Time               10.19 h
INSTRUM            spect
PROBHD   Z108618_0257 (
PULPROG           zgpg30
TD                 65536
SOLVENT             C6D6
NS                    40
DS                     4
SWH            29761.904 Hz
FIDRES          0.454131 Hz
AQ             1.1010048 sec
RG                   512
DW                16.800 usec
DE                  6.50 usec
TE                 296.9 K
D1            2.00000000 sec
D11           0.03000000 sec
TD0                    1
SFO1         128.4096890 MHz
NUC1                 11B
P1                  7.50 usec
PLW1         55.09999847 W
SFO2         400.2316008 MHz
NUC2                  1H
CPDPRG[2         waltz16
PCPD2              90.00 usec
PLW2         13.56000042 W
PLW12         0.27428001 W
PLW13         0.13796000 W

F2 - Processing parameters
SI                 32768
SF           128.4095347 MHz
WDW                   EM
SSB      0
LB                  1.00 Hz
GB       0
PC                  1.40
```

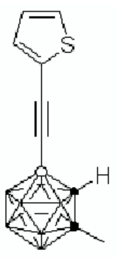

5a

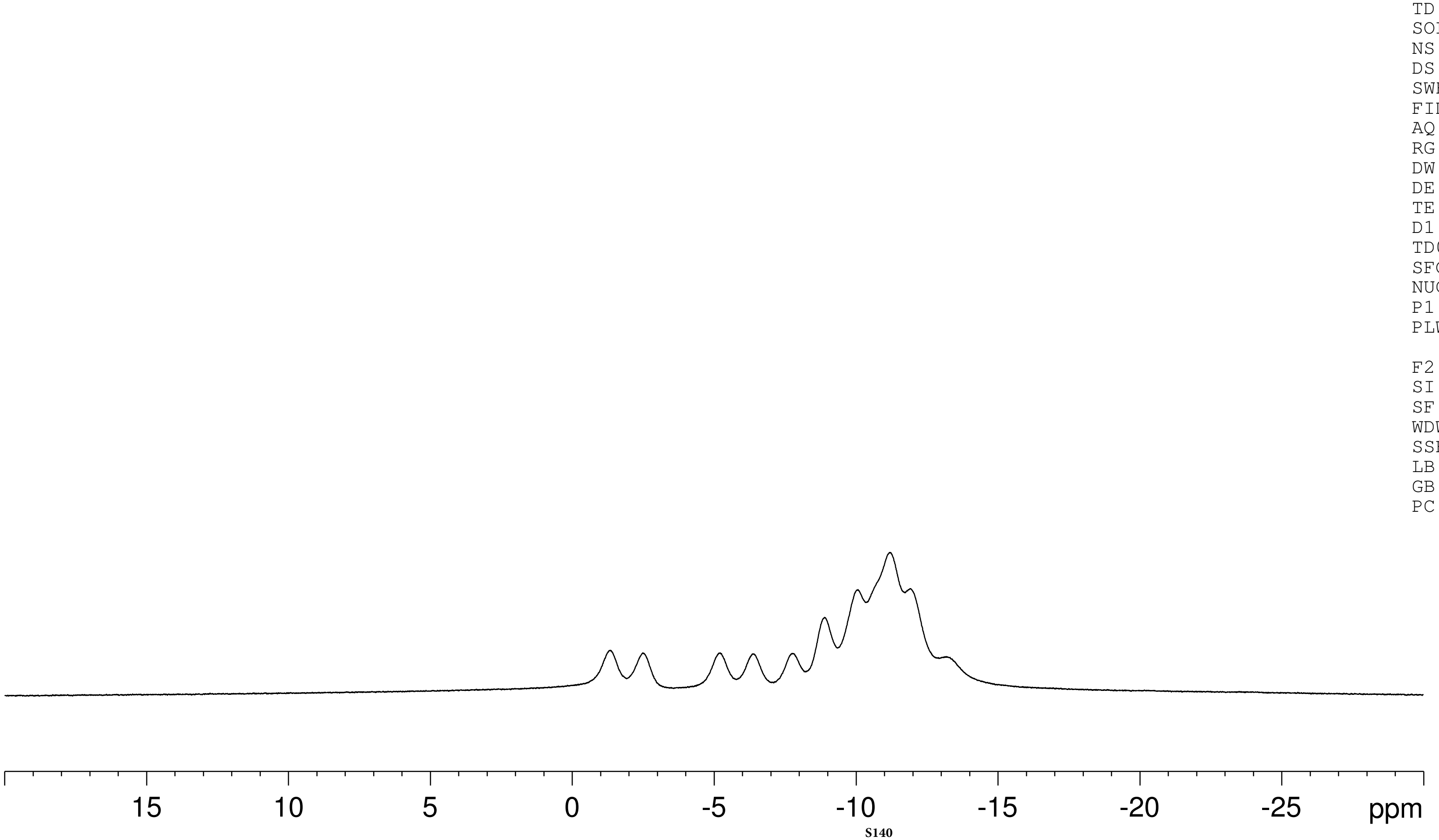

qyj-B-6-1-CDCl3(C)

Current Data Parameters
NAME qyj-B-6-1-CDCl3(C)
EXPNO 1
PROCNO 1

F2 - Acquisition Parameters
Date_ 20150622
Time 10.23 h
INSTRUM spect
PROBHD Z108618_0257 (
PULPROG zg
TD 65536
SOLVENT C6D6
NS 48
DS 4
SWH 25510.203 Hz
FIDRES 0.389255 Hz
AQ 1.2845056 sec
RG 322
DW 19.600 usec
DE 6.50 usec
TE 296.5 K
D1 1.00000000 sec
TD0 1
SFO1 128.4096891 MHz
NUC1 11B
P1 7.50 usec
PLW1 55.09999847 W

F2 - Processing parameters
SI 32768
SF 128.4096891 MHz
WDW EM
SSB 0
LB 1.00 Hz
GB 0
PC 1.40

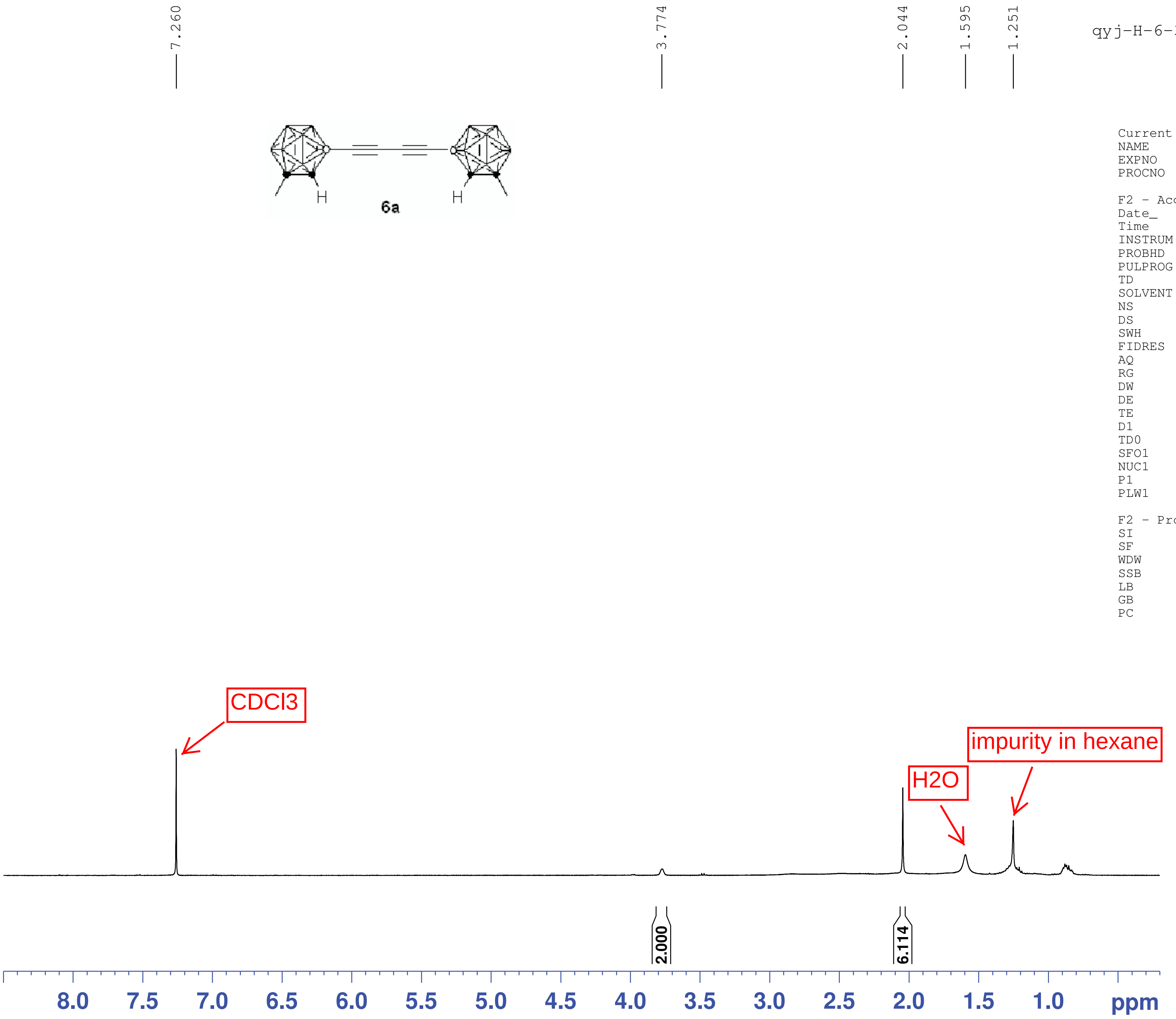

qyj-H-6-3-CDCl3

```
Current Data Parameters
NAME       qyj-H-6-3-CDCl3
EXPNO                    1
PROCNO                   1

F2 - Acquisition Parameters
Date_             20150614
Time                 15.38 h
INSTRUM              spect
PROBHD    Z824601_0021 (
PULPROG               zg30
TD                   65536
SOLVENT              CDCl3
NS                       8
DS                       2
SWH               8012.820 Hz
FIDRES            0.122266 Hz
AQ               4.0894465 sec
RG                     203
DW                  62.400 usec
DE                    6.50 usec
TE                   295.0 K
D1              1.00000000 sec
TD0                      1
SFO1           400.1324708 MHz
NUC1                    1H
P1                   15.00 usec
PLW1            8.31000042 W

F2 - Processing parameters
SI                   65536
SF             400.1300099 MHz
WDW                     EM
SSB      0
LB                    0.30 Hz
GB       0
PC                    1.00
```

qyj-C-6-3-CDCl3

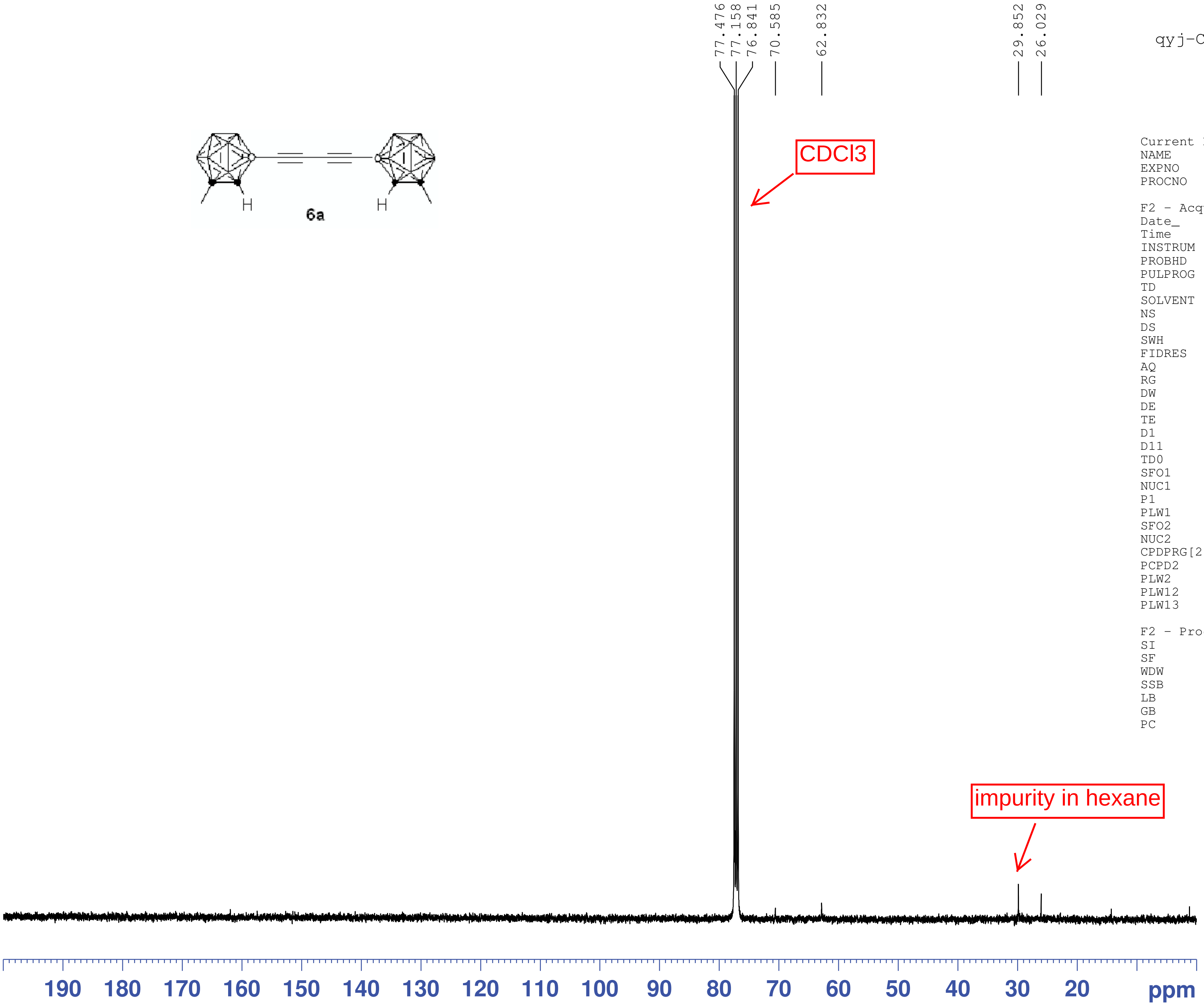


```
Current Data Parameters
NAME      qyj-C-6-3-CDCl3
EXPNO                   1
PROCNO                  1

F2 - Acquisition Parameters
Date_            20150614
Time                15.41 h
INSTRUM             spect
PROBHD   Z824601_0021 (
PULPROG           zgpg30
TD                 65536
SOLVENT            CDCl3
NS                  4421
DS                     4
SWH            24038.461 Hz
FIDRES          0.366798 Hz
AQ             1.3631488 sec
RG                   203
DW                20.800 usec
DE                  6.50 usec
TE                 295.1 K
D1            2.00000000 sec
D11           0.03000000 sec
TD0                    1
SFO1         100.6228298 MHz
NUC1                 13C
P1                  9.50 usec
PLW1         41.25000000 W
SFO2         400.1316005 MHz
NUC2                  1H
CPDPRG[2         waltz16
PCPD2              90.00 usec
PLW2          8.31000042 W
PLW12         0.23083000 W
PLW13         0.11611000 W

F2 - Processing parameters
SI                 32768
SF           100.6127549 MHz
WDW                   EM
SSB      0
LB                  1.00 Hz
GB       0
PC                  1.40
```

qyj-B-6-3-CDCl3

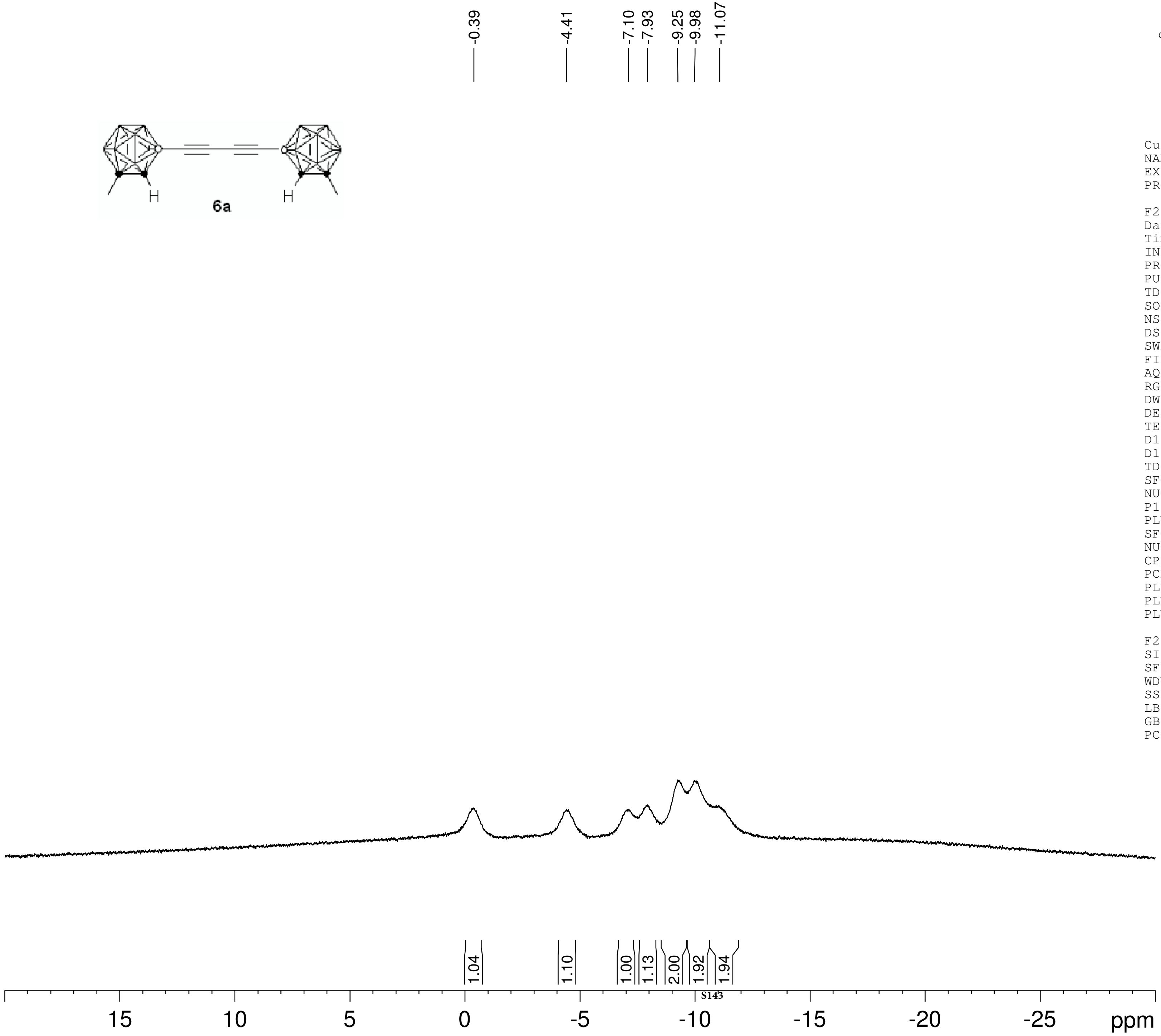

Current Data Parameters
NAME qyj-B-6-3-CDCl3
EXPNO 1
PROCNO 1

F2 - Acquisition Parameters
Date_ 20150622
Time 10.31 h
INSTRUM spect
PROBHD Z108618_0257 (
PULPROG zgpg30
TD 65536
SOLVENT C6D6
NS 80
DS 4
SWH 29761.904 Hz
FIDRES 0.454131 Hz
AQ 1.1010048 sec
RG 512
DW 16.800 usec
DE 6.50 usec
TE 296.7 K
D1 2.00000000 sec
D11 0.03000000 sec
TD0 1
SFO1 128.4096890 MHz
NUC1 11B
P1 7.50 usec
PLW1 55.09999847 W
SFO2 400.2316008 MHz
NUC2 1H
CPDPRG[2 waltz16
PCPD2 90.00 usec
PLW2 13.56000042 W
PLW12 0.27428001 W
PLW13 0.13796000 W

F2 - Processing parameters
SI 32768
SF 128.4095347 MHz
WDW EM
SSB 0
LB 1.00 Hz
GB 0
PC 1.40

qyj-B-6-3-CDCl3(C)

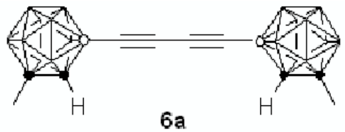

6a

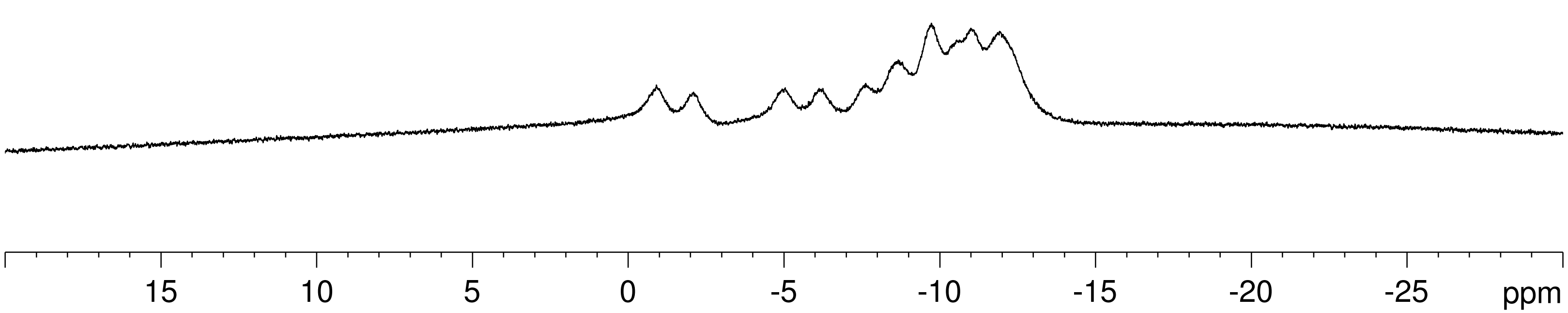

Current Data Parameters
NAME qyj-B-6-3-CDCl3(C)
EXPNO 1
PROCNO 1

F2 - Acquisition Parameters
Date_ 20150622
Time 10.35 h
INSTRUM spect
PROBHD Z108618_0257 (
PULPROG zg
TD 65536
SOLVENT C6D6
NS 40
DS 4
SWH 25510.203 Hz
FIDRES 0.389255 Hz
AQ 1.2845056 sec
RG 322
DW 19.600 usec
DE 6.50 usec
TE 296.2 K
D1 1.00000000 sec
TD0 1
SFO1 128.4096891 MHz
NUC1 11B
P1 7.50 usec
PLW1 55.09999847 W

F2 - Processing parameters
SI 32768
SF 128.4096891 MHz
WDW EM
SSB 0
LB 1.00 Hz
GB 0
PC 1.40

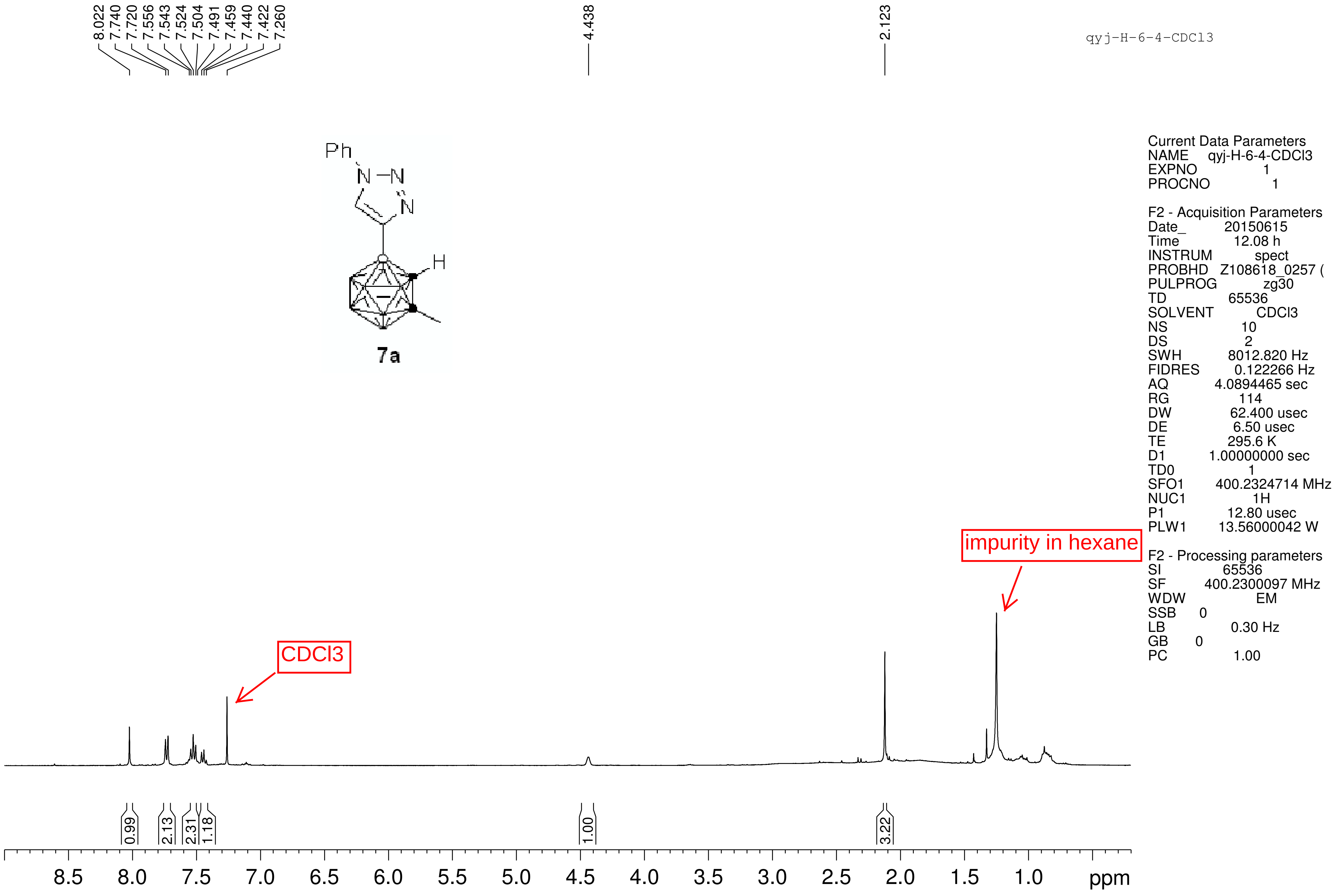
8.022
7.740
7.720
7.556
7.543
7.524
7.504
7.491
7.459
7.440
7.422
7.260
4.438
2.123
qyj-H-6-4-CDCl3
Ph
N
N
N
H
7a
CDCl3
impurity in hexane
0.99
2.13
2.31
1.18
1.00
3.22
8.5
8.0
7.5
7.0
6.5
6.0
5.5
5.0
4.5
4.0
3.5
3.0
2.5
2.0
1.5
1.0
ppm
Current Data Parameters
NAME qyj-H-6-4-CDCl3
EXPNO 1
PROCNO 1
F2 - Acquisition Parameters
Date_ 20150615
Time 12.08 h
INSTRUM spect
PROBHD Z108618_0257 (
PULPROG zg30
TD 65536
SOLVENT CDCl3
NS 10
DS 2
SWH 8012.820 Hz
FIDRES 0.122266 Hz
AQ 4.0894465 sec
RG 114
DW 62.400 usec
DE 6.50 usec
TE 295.6 K
D1 1.00000000 sec
TD0 1
SFO1 400.2324714 MHz
NUC1 1H
P1 12.80 usec
PLW1 13.56000042 W
F2 - Processing parameters
SI 65536
SF 400.2300097 MHz
WDW EM
SSB 0
LB 0.30 Hz
GB 0
PC 1.00

qyj-C-6-4-CDCl3

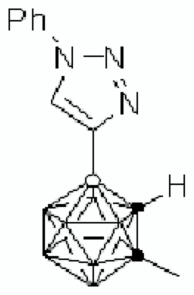

7a

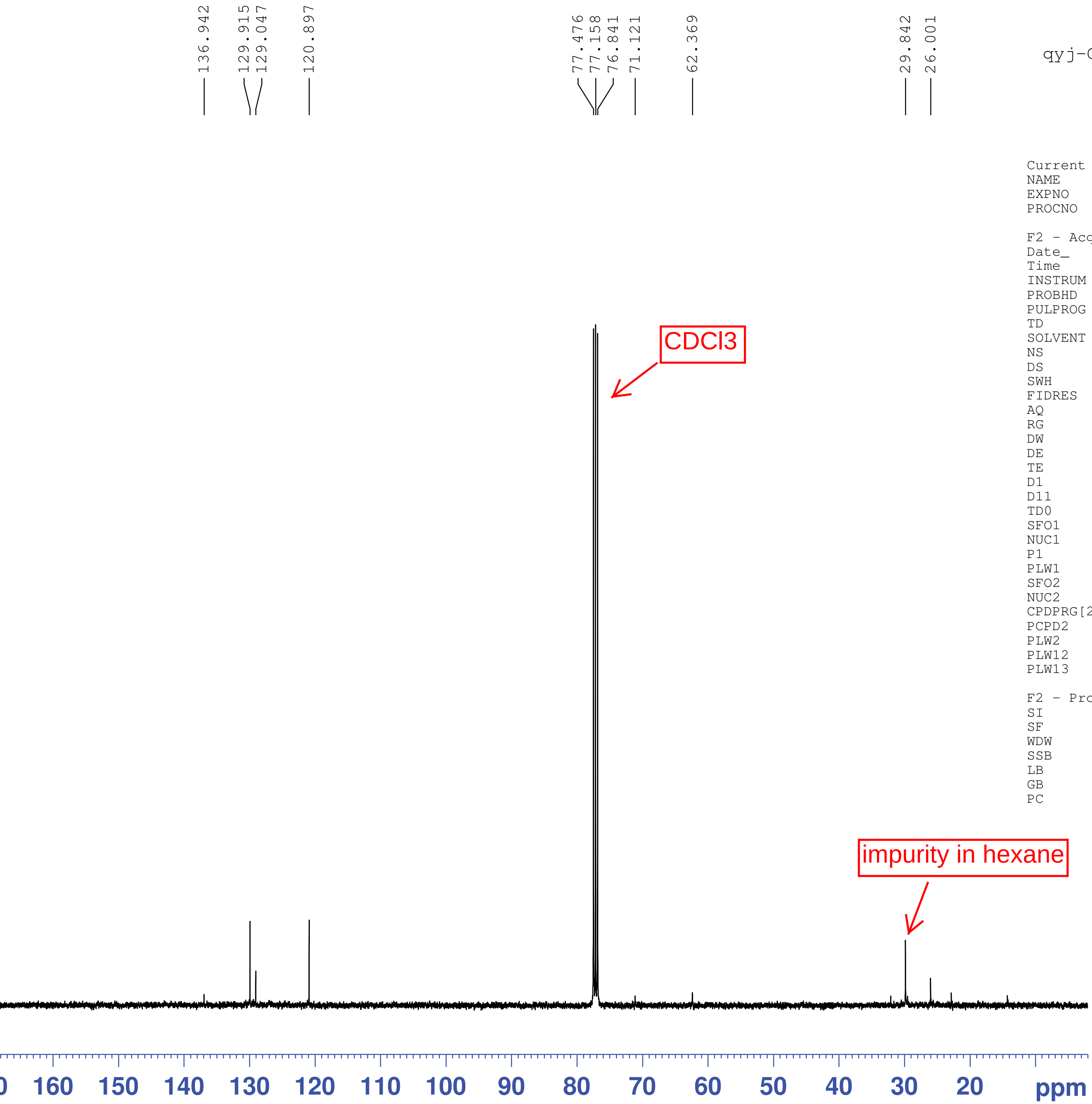

Current Data Parameters
NAME qyj-C-6-4-CDCl3
EXPNO 1
PROCNO 1

F2 - Acquisition Parameters
Date_ 20150615
Time 14.58 h
INSTRUM spect
PROBHD Z824601_0021 (
PULPROG zgpg30
TD 65536
SOLVENT CDCl3
NS 888
DS 4
SWH 24038.461 Hz
FIDRES 0.366798 Hz
AQ 1.3631488 sec
RG 203
DW 20.800 usec
DE 6.50 usec
TE 295.2 K
D1 2.00000000 sec
D11 0.03000000 sec
TD0 1
SFO1 100.6228298 MHz
NUC1 13C
P1 9.50 usec
PLW1 41.25000000 W
SFO2 400.1316005 MHz
NUC2 1H
CPDPRG[2 waltz16
PCPD2 90.00 usec
PLW2 8.31000042 W
PLW12 0.23083000 W
PLW13 0.11611000 W

F2 - Processing parameters
SI 32768
SF 100.6127557 MHz
WDW EM
SSB 0
LB 1.00 Hz
GB 0
PC 1.40

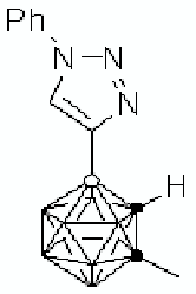

7a

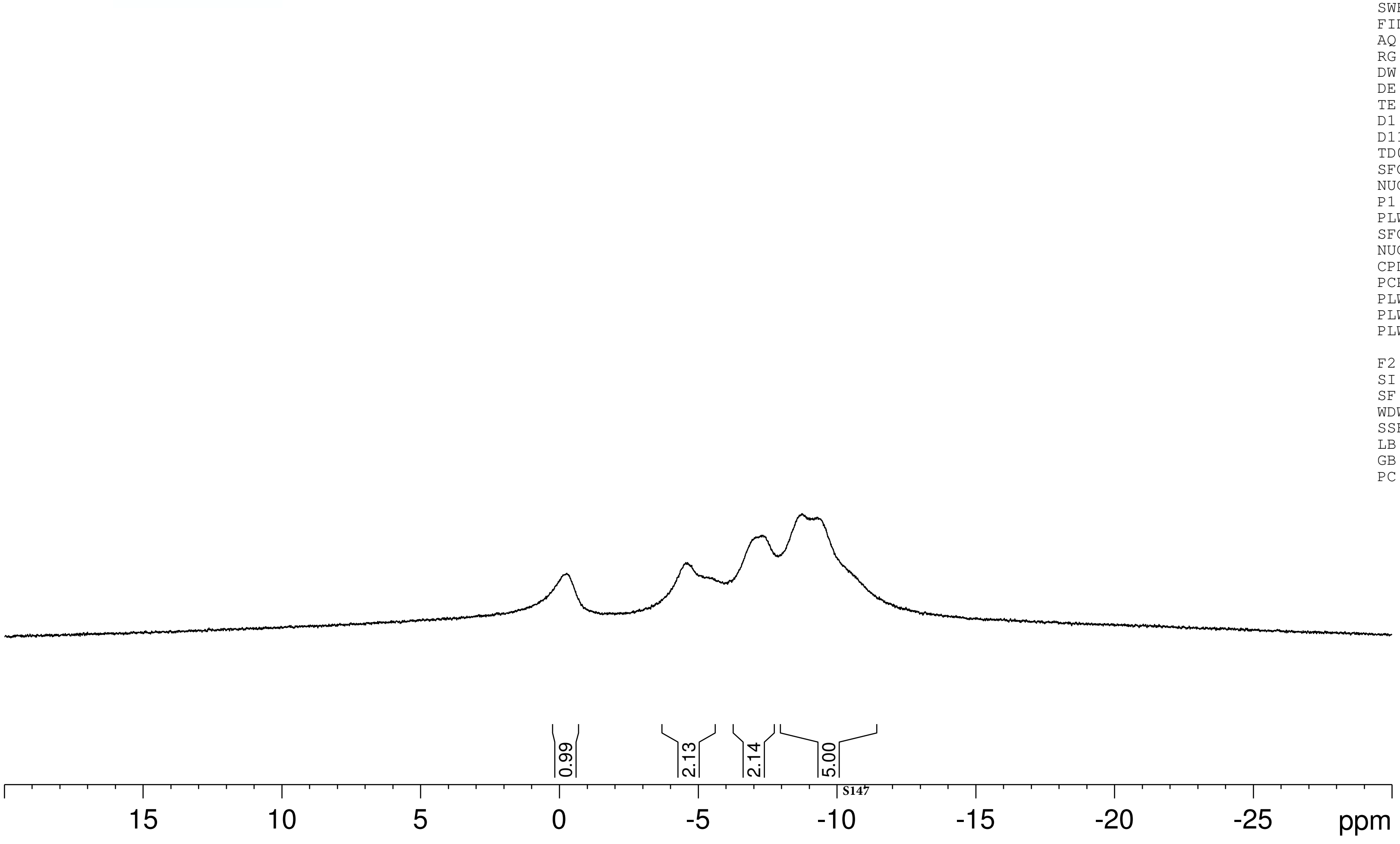

qyj-B-6-4-CDCl3

```
Current Data Parameters
NAME      qyj-B-6-4-CDCl3
EXPNO                    1
PROCNO                   1

F2 - Acquisition Parameters
Date_             20150622
Time                 10.37 h
INSTRUM              spect
PROBHD    Z108618_0257 (
PULPROG             zgpg30
TD                   65536
SOLVENT               C6D6
NS                      64
DS                       4
SWH              29761.904 Hz
FIDRES            0.454131 Hz
AQ               1.1010048 sec
RG                     512
DW                  16.800 usec
DE                    6.50 usec
TE                   296.4 K
D1              2.00000000 sec
D11             0.03000000 sec
TD0                      1
SFO1           128.4096890 MHz
NUC1                   11B
P1                    7.50 usec
PLW1           55.09999847 W
SFO2           400.2316008 MHz
NUC2                    1H
CPDPRG[2           waltz16
PCPD2                90.00 usec
PLW2           13.56000042 W
PLW12           0.27428001 W
PLW13           0.13796000 W

F2 - Processing parameters
SI                   32768
SF             128.4095347 MHz
WDW                     EM
SSB       0
LB                    1.00 Hz
GB        0
PC                    1.40
```

qyj-B-6-4-CDCl3(C)

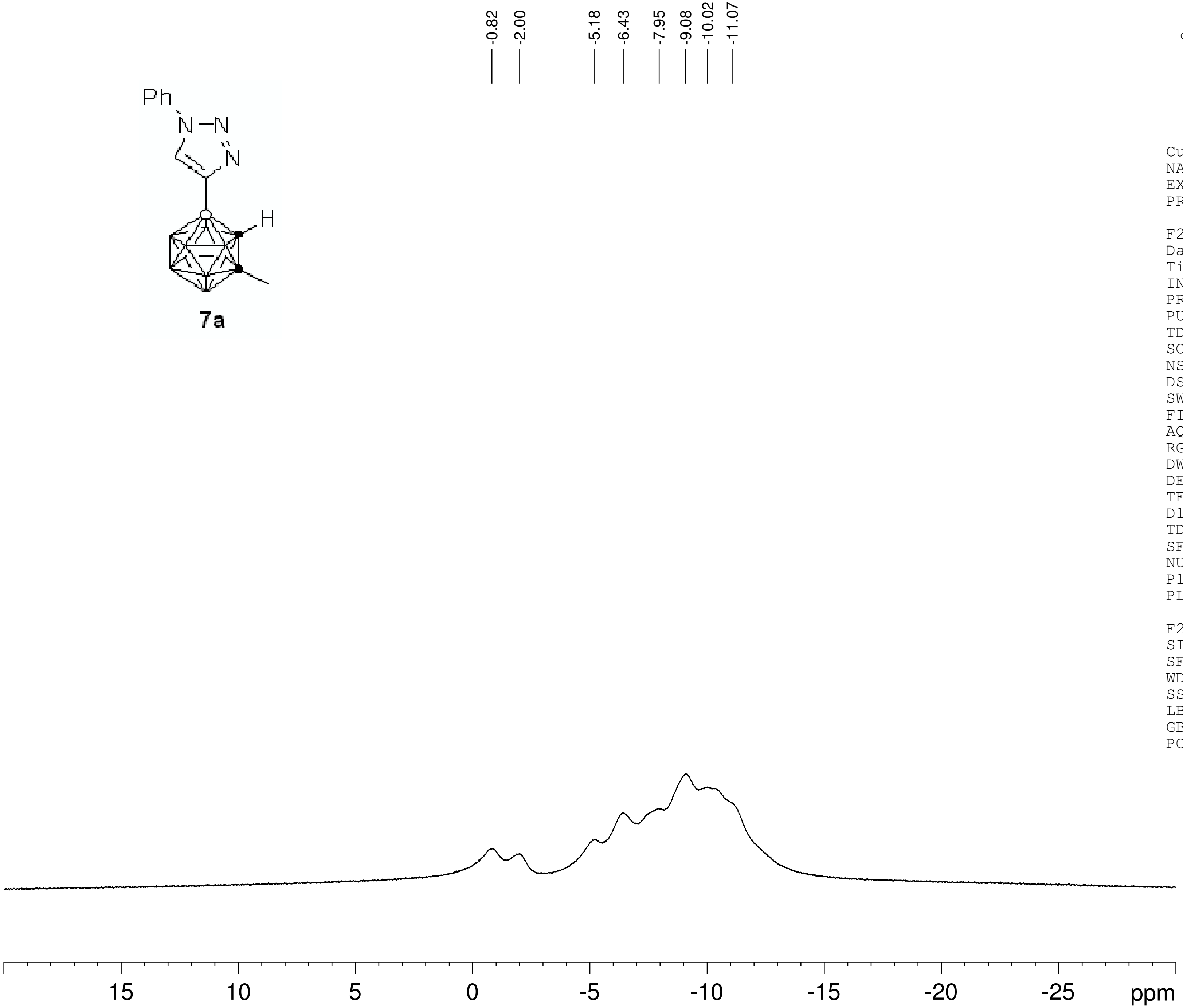

Current Data Parameters
NAME qyj-B-6-4-CDCl3(C)
EXPNO 1
PROCNO 1

F2 - Acquisition Parameters
Date_ 20150622
Time 10.42 h
INSTRUM spect
PROBHD Z108618_0257 (
PULPROG zg
TD 65536
SOLVENT C6D6
NS 56
DS 4
SWH 25510.203 Hz
FIDRES 0.389255 Hz
AQ 1.2845056 sec
RG 322
DW 19.600 usec
DE 6.50 usec
TE 296.2 K
D1 1.00000000 sec
TD0 1
SFO1 128.4096891 MHz
NUC1 11B
P1 7.50 usec
PLW1 55.09999847 W

F2 - Processing parameters
SI 32768
SF 128.4096891 MHz
WDW EM
SSB 0
LB 1.00 Hz
GB 0
PC 1.40
